# Assessing the causal relationship between circulating immune cells and abdominal aortic aneurysm by bi-directional Mendelian randomization analysis

**DOI:** 10.1038/s41598-024-64789-9

**Published:** 2024-06-14

**Authors:** Weiqiang Ruan, Xiaoqin Zhou, Ting Wang, Huizhen Liu, Guiying Zhang, Jiaoyan Sun, Ke Lin

**Affiliations:** 1 Department of Cardiovascular Surgery, West China Hospital, Sichuan University, No. 37, Guoxue Xiang, Chengdu, 610041 Sichuan People’s Republic of China; 2 Department of Vascular Surgery, West China Hospital, Sichuan University, Chengdu, People’s Republic of China; 3 Research Center of Clinical Epidemiology and Evidence-Based Medicine, West China Hospital, Sichuan University, Chengdu, People’s Republic of China; 4 Center of Biostatistics, Design, Measurement and Evaluation (CBDME), Department of Clinical Research Management, West China Hospital, Sichuan University, Chengdu, People’s Republic of China; 5 West China School of Public Health, Sichuan University, Chengdu, People’s Republic of China

**Keywords:** Abdominal aortic aneurysm, Immune cells, Genome-wide association study, Mendelian randomization, Cardiology, Risk factors, Vascular diseases

## Abstract

Although there is an association between abdominal aortic aneurysm (AAA) and circulating immune cell phenotypes, the exact causal relationship remains unclear. This study aimed to explore the causal relationships between immune cell phenotypes and AAA risk using a bidirectional two-sample Mendelian randomization approach. Data from genome-wide association studies pertaining to 731 immune cell traits and AAA were systematically analyzed. Using strict selection criteria, we identified 339 immune traits that are associated with at least 3 single nucleotide polymorphisms. A comprehensive MR analysis was conducted using several methods including Inverse Variance Weighted, Weighted Median Estimator, MR-Egger regression, Weighted Mode, and Simple Median methods. CD24 on switched memory cells (OR = 0.922, 95% CI 0.914–0.929, *P* = 2.62e−79) at the median fluorescence intensities level, and SSC-A on HLA-DR + natural killer cells (OR = 0.873, 95% CI 0.861–0.885, *P* = 8.96e−81) at the morphological parameter level, exhibited the strongest causal associations with AAA. In the reverse analysis, no significant causal effects of AAA on immune traits were found. The study elucidates the causal involvement of multiple circulating immune cell phenotypes in AAA development, signifying their potential as diagnostic markers or therapeutic targets. These identified immune traits may be crucial in modulating AAA-related inflammatory pathways.

## Introduction

Abdominal aortic aneurysms (AAA) are characterized by localized, permanent dilation of the abdominal aorta exceeding 50% of normal diameter or 3 cm in width^1,2^. This chronic degenerative condition results from progressive structural deterioration and weakening of the aortic wall. Known risk factors for AAA include advanced age, male sex, white race, smoking, dyslipidemia, and atherosclerosis^3,4^. If AAA dilation remains undetected and untreated, continued expansion can eventually precipitate life-threatening aortic rupture associated with high mortality. AAA affects roughly 2–5% of men and 0.5–1.5% of women over 60 years old in developed nations, representing a major source of cardiovascular morbidity and mortality^5^.

The pathogenesis of AAA is driven by chronic inflammation, extracellular matrix proteolysis, and smooth muscle cell apoptosis within the aortic wall^2,3^. AAA tissues exhibit infiltration of various leukocytes including macrophages, T cells, B cells, and mast cells^6–9^. Based on this evidence, the circulating immune cells are suspected to play a central role in AAA pathogenesis. However, the causal influence of systemic immune cell populations on AAA risk remains uncertain. While numerous observational studies have reported associations between circulating immune cells and presence, progression, or complications of AAA, residual confounding and reverse causation limit robust causal inference^10–13^.

Mendelian randomization (MR) leverages genetic variants as instrumental variables to strengthen causal conclusions from observational data. Since alleles are randomly allocated during meiosis independently of confounders, MR studies are less prone to confounding biases^14,15^. Additionally, the unidirectional flow of information from germline DNA sequence to downstream molecular traits minimizes reverse causation. Here, we performed bidirectional MR analyses to definitively investigate for causal relationships between circulating immune cell phenotypes and AAA risk. Elucidating the causal role of peripheral immune cells and inflammatory pathways in AAA may uncover potential drug targets or biomarkers to guide therapeutic monitoring and early intervention.

## Methods

### Study design and the assumption of MR

**Figure 1:**
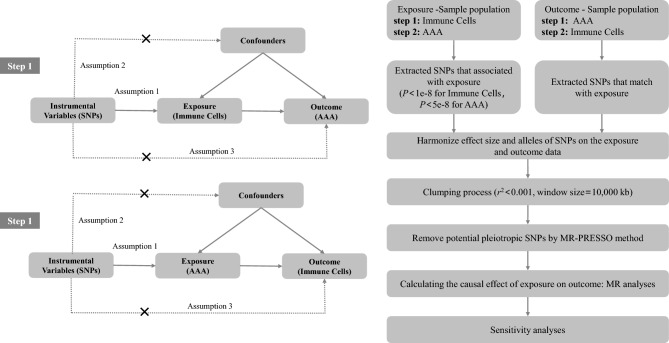
Schematic Representation of the Mendelian Randomization (MR) Analysis. The flowchart of the study based on three assumptions: (1) the instrumental variables (IVs) selected from datasets were associated with exposure; (2) the IVs were not associated with confounders; and (3) the IVs influences the outcome only through exposure. *AAA*, Abdominal Aortic Aneurysm, *SNP* single nucleotide polymorphism.

We utilized publicly available large-scale Genome-Wide Association Study (GWAS) data to conduct a two-sample MR analysis. This enabled us to explore the genetic associations circulating immune cell phenotypes and AAA. Concurrently, we reversed the exposure and outcome variables to perform a complementary inverse MR analysis, adding robustness to our findings. The schematic flow of our study methodology is graphically depicted in Fig. 1.

### Data sources

To provide a more robust and comprehensive understanding of the causal links between immune indicators and AAA, we leveraged the largest GWAS available for immunophenotyping of peripheral blood. This GWAS comprehensively characterized 731 distinct immune cell phenotypes in a cohort of 3757 individuals from Sardinia^16^. The study captured a wide array of immune cell types, providing detailed measurements that included 118 absolute cell (AC) counts, 389 median fluorescence intensities (MFIs) reflecting surface antigen expression levels, 32 morphological parameters (MPs) (captured through forward scatter (FSC) and side scatter analyses (SSC), proportional to cell size and complexity), and 192 relative cell (RC) counts. Approximately 22 million Single Nucleotide Polymorphisms (SNPs) were genotyped using high-density arrays or were imputed based on a Sardinian-specific reference panel. All analyses were rigorously adjusted for sex and age confounding variables. The summary statistics for each of the 731 phenotypic GWAS outcomes are publicly accessible through the GWAS Catalog (accession numbers GCST0001391 to GCST0002121).

GWAS summary statistics for AAA were sourced from a comprehensive meta-analysis of 17 individual GWAS spanning 14 distinct discovery cohorts, as reported in Roychowdhury et al.^17^. This all-encompassing meta-analysis, the most extensive of its kind to date, incorporated data from 39,221 AAA cases—37,214 of European descent and 2007 of African descent—and 1,086,107 controls derived from diverse population-based studies and biobanks. Prominent contributors to the dataset include illustrious studies like the Million Veteran Program, UK Biobank, and deCODE genetics, among others (a detailed breakdown is provided in Supplemental Table 1). Genotyping in the discovery cohorts was performed using various high-density SNP arrays, and genotype imputation was carried out using population-specific reference panels. After quality control and meta-analysis, summary statistics for 33.4 million SNPs were generated. These data have been made publicly available (https://csg.sph.umich.edu/willer/public/AAAgen2023/).

### Instrumental variable selection

Consistent with prior MR analyses, we adopted the same cut-off value of *P* < 5 × 10^–8^ to identify significant SNPs for each immune trait with the same criteria in our MR analysis^18^. When conducting a reverse MR analysis with AAA as the exposure, we set the same threshold for selecting SNPs.

The following three assumptions were satisfied for the two sample MR: (1) the instrumental variables (IVs) selected from datasets were associated with exposure. (2) the IVs were not associated with confounders. (3) the IVs influences the outcome only through exposure^14^. Second, the clump program in PLINK software was adopted to exclude the dependent instrumental variable of *r*^2^ < 0.001 (clumping window size = 10,000 kb), which was obtained using the 1000 Genome Projects reference panel in Europe^19^. Third, SNPs with minor allele frequency (MAF) < 0.01 were removed. Fourth, an important step in MR is to ensure that the effects of the SNPs on the exposure correspond to the same allele as the effects on the outcome. To avoid distortion of strand orientation or allele coding, we deleted palindromic SNPs (e.g. with A/T or G/C alleles).

The *F*-statistic of the IVs was calculated to detect whether or not there was a weak IV bias, an F-value less than 10 indicated a weak instrument and was excluded^20^. Through the PhenoScanner website (http://www.phenoscanner.medschl.cam.ac.uk) and the GWAS catalog (https://www.ebi.ac.uk/gwas/), we removed SNPs associated with "smoke", "high density lipoprotein", "low density lipoprotein", "Triglycerides", "total cholesterol", "apolipoprotein A1", "apolipoprotein B", "Hypertension", "Coronary artery disease", in order to limit the influence of confounding factors^21^. Then we utilized the remaining IVs for MR analysis. We further only included immune trait with at least three SNPs as IVs in downstream analyses.

### Statistical analysis for MR

We conducted bidirectional MR analysis to test both whether immune cell traits causally influence AAA and vice versa. Independent SNPs associated with each immune trait and AAA at genome-wide significance were used as instrumental variables. To test for causal effects of immune cells on AAA, we applied inverse variance weighted (IVW)^22,23^, weighted median (WME)^24^, weighted mode (WMo)^25^, MR-Egger^26^, and Simple Mode (SM) methods. We evaluated reverse direction effects of AAA on immune cells similarly. Multiple testing was adjusted using the FDR approach.

### Heterogeneity and pleiotropy analyses

We assessed heterogeneity through the application of Cochran's Q statistics, considering a *P* < 0.05 as indicative of significant heterogeneity^27^. To evaluate the direct relationship between the chosen IVs and the outcome, horizontal pleiotropy was tested using MR-Egger regression and MR-PRESSO global tests^28,29^. Any outliers of significance identified via the MR-PRESSO analysis were excluded to minimize the impact of horizontal pleiotropy. Additionally, we conducted a leave-one-out analysis to validate the robustness of the conclusions^27^.

### Analysis software

All statistical analyses were conducted using R version 4.2.3 (R Foundation for Statistical Computing, Vienna, Austria, https://cran.r-project.org/src/base/R-4/R-4.2.3.tar.gz). For MR analyses, we utilized the TwosampleMR (Version 0.5.7, https://github.com/MRCIEU/TwoSampleMR) and MR-PRESSO (Version 1.0, https://github.com/rondolab/MR-PRESSO)^29^ R packages.

## Results

### Exploration of the causal effect of immunophenotypes on AAA

In our two-sample MR study, we explored a comprehensive array of 731 circulating immune cell traits to discern their potential causal relationship with AAA. A stringent series of selection criteria narrowed this to 339 immune traits associated with at least three SNPs, as detailed in Supplemental Table 2.

**Figure 2:**
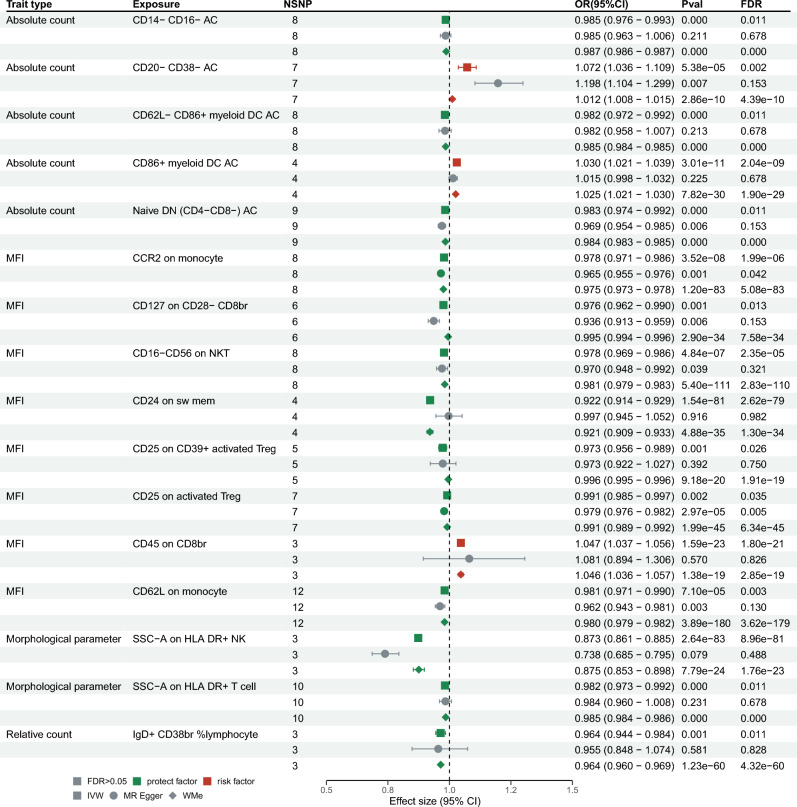
Causal Effects of immunophenotypes on Abdominal Aortic Aneurysm. *IVW* inverse variance weighted, *WMe* weighted median, *MFI* median fluorescence intensitie.

**Figure 3:**
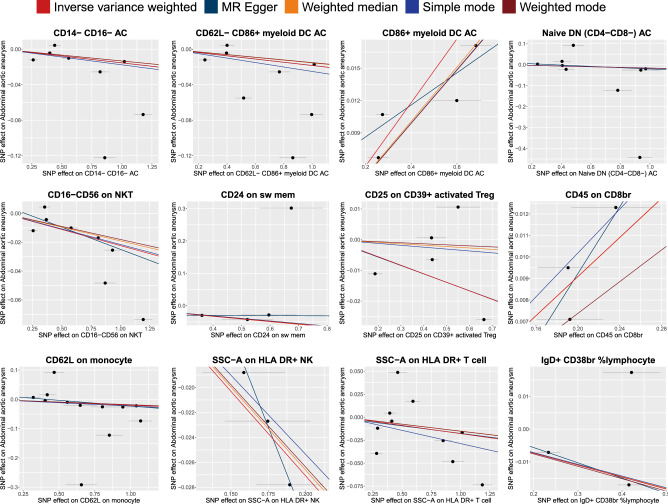
Scatter plots of the effect of the immunophenotypes on Abdominal Aortic Aneurysm.

Through the application of the IVW approach in MR, we identified 46 immune traits significantly associated with AAA risk. Importantly, 34 of these traits had consistent odds ratio (OR) directions across all employed methodologies—including IVW, WME, MR-Egger regression, WMo, and SM methods. After adjusting for multiple comparisons using the False Discovery Rate (FDR) method and performing additional sensitivity analyses, 16 immune trait-AAA pairs remained statistically significant (FDR-adjusted *P* < 0.05). These pairs included 8 MFIs, 5 ACs, 1 RCs, and 2 MPs, details in Supplemental Table 3 and Figs. 2, 3.

For traits measured at the Absolute Count level, we found strong evidence for causal relationships with AAA risk. Specifically, CD86 + myeloid DC AC (OR = 1.03, 95% confidence interval (CI) 1.021–1.039, *P* = 2.04e − 09) and CD20- CD38- AC (OR = 1.072, 95% CI 1.036–1.109, *P* = 2.28e − 03) demonstrated a significant positive causal effect on AAA risk. On the other hand, Naive DN (CD4-CD8-) AC (OR = 0.983, 95% CI 0.974–0.992, *P* = 1.05e − 02), CD14- CD16- AC (OR = 0.985, 95% CI 0.976–0.993, *P* = 1.05e − 02), and CD62L- CD86 + myeloid DC AC (OR = 0.982, 95% CI 0.972–0.992, *P* = 1.05e − 02) showed a significant negative causal effect on AAA risk.

When scrutinizing traits gauged through Mean Fluorescence Intensities, our MR analyses revealed significant causal relationships with AAA risk. CD24 on sw mem (OR = 0.922, 95% CI 0.914–0.929, *P* = 2.62e − 79) and CCR2 on monocyte (OR = 0.978, 95% CI 0.971–0.986, *P* = 1.99e − 06) demonstrated a significant negative causal effect on AAA risk. Similarly, CD16-CD56 on NKT (OR = 0.978, 95% CI 0.969–0.986, *P* = 2.35e − 05), CD62L on monocyte (OR = 0.981, 95% CI 0.971–0.990, *P* = 2.67e − 03), CD127 on CD28- CD8br (OR = 0.976, 95% CI 0.962–0.990, *P* = 1.32e − 02), CD25 on CD39 + activated Treg (OR = 0.973, 95% CI 0.956–0.989, *P* = 2.59e − 02), and CD25 on activated Treg (OR = 0.991, 95% CI 0.985–0.997, *P* = 3.45e − 02) also indicated a significant negative causal impact on AAA risk. In contrast, CD45 on CD8br (OR = 1.047, 95% CI 1.037–1.056, *P* = 1.80e − 21) showed a significant positive causal effect on AAA risk.

In terms of Morphological Parameters, SSC-A on HLA DR + NK (OR = 0.873, 95% CI 0.861–0.885, P = 8.96e − 81) and SSC-A on HLA DR + T cell (OR = 0.982, 95% CI 0.973–0.992, P = 1.07e − 02) indicated a significant negative causal effect on AAA risk.

Regarding traits characterized by Relative Counts, IgD + CD38br %lymphocyte (OR = 0.964, 95% CI 0.944–0.984, *P* = 1.15e−02) exhibited a significant negative causal impact on AAA risk.

**Figure 4:**
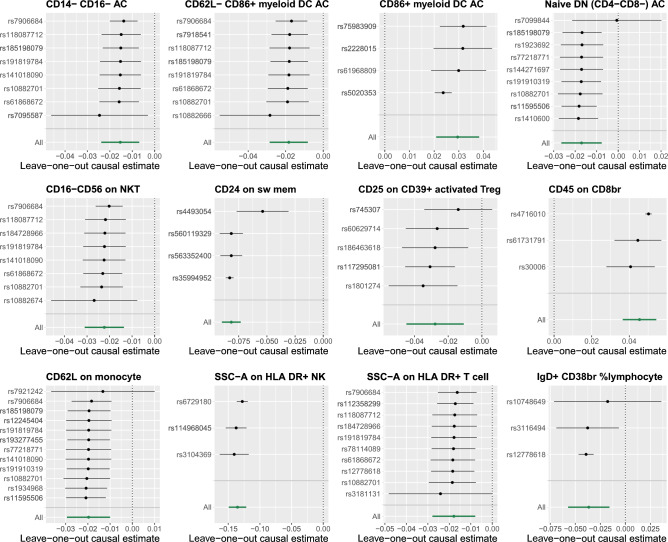
Leave-one-out plots of the effect of immunophenotypes on Abdominal Aortic Aneurysm.

In the MR-Egger regression analysis, four immune cell phenotypes demonstrated horizontal pleiotropy and were consequently excluded. Specifically, these included: Absolute count of CD20- CD38- AC with a *P-value* of 0.039, Mean Fluorescence Intensity (MFI) of CCR2 on monocytes with a *P-value* of 0.034, MFI of CD127 on CD28- CD8br with a *P-value* of 0.025, and MFI of CD25 on activated Treg with a *P-value* of 0. In addition, evidence of heterogeneity was uncovered by both Cochran's Q statistics and the MR-PRESSO global test. Consequently, we relied on the results from the IVW random effects model as our final outcomes. Notably, these findings remained robust after outlier correction, except for MFI of CCR2 on monocytes (Outlier-Corrected *P* = 0.138), as detailed in Supplemental Table 4. Moreover, a leave-one-out sensitivity analysis confirmed that no individual SNP disproportionately influenced the causal estimates associated with AAA, as illustrated in Fig. 4. Ultimately, we identified 12 immune cell phenotypes exerting a causal effect on AAA risk. Most notably, CD24 on switched memory cells (OR = 0.922, 95% CI 0.914–0.929, *P* = 2.62e−79) at the MFI level, and SSC-A on HLA-DR + natural killer cells (OR = 0.873, 95% CI 0.861–0.885, *P* = 8.96e−81) at the morphological parameter level, exhibited the strongest causal associations with AAA.

### Exploration of the causal effect of AAA onset on immunophenotypes

In our comprehensive analysis focused on the causal impact of AAA onset on 731 immunophenotypes, we employed the IVW method. This revealed that AAA was significantly associated with 18 immunophenotypes. Notably, 10 of these immunophenotypes displayed consistent OR directions across all employed analytical techniques. However, after adjusting for multiple comparisons via the FDR methodology and conducting additional sensitivity tests, AAA was found to have no significant causal effect on any of the investigated immunophenotypes (Supplemental Tables 5, 6).

## Discussion

Our bidirectional MR study offers a comprehensive analysis of the causal links between circulating immune cell phenotypes and the development of AAA. Notably, 10 immune traits remained statistically significant (FDR-adjusted *P* < 0.05) after rigorous statistical adjustments, offering potential therapeutic targets. However, our results do not suggest a significant causal effect of AAA onset on any of the investigated immune traits. Herein, we categorize our discussion of the Mechanisms of Association into four parts according to immune phenotype types. It's important to note that the potential mechanisms discussed below are based on hypotheses from previous literature and require further studies for validation.

### Absolute count

Higher counts of CD86 + myeloid dendritic cells were associated with increased AAA risk. CD86 is an immune co-stimulatory molecule predominantly expressed on antigen-presenting cells like dendritic cells and macrophages. It serves as a crucial facilitator for both innate and adaptive immune responses^30,31^. Elevated levels of circulating CD86 + myeloid DCs may potentiate the risk of AAA by amplifying proinflammatory T-cell activities.

CD86 interacts with CD28 on naïve T cells, providing the necessary co-stimulatory signals for T-cell activation^32^. This interaction effectively lowers the threshold for T-cell receptor-mediated responses. Once activated, T cells may contribute to the inflammatory cascade by releasing metalloproteinases (MMPs) which degrade the aortic wall's extracellular matrix^30,33^.

### Relative count

IgD + CD38br B cells Relative count displayed inverse association with AAA risk. IgD + CD38br B cells represent an activated B cell subset that can rapidly secrete IgM antibodies and participate in early immune responses^34,35^. Elevated levels of these activated B cells have been reported in some autoimmune diseases^36^. However, their specific role in AAA pathophysiology remains unclear and warrants further investigation.

### Mean fluorescence intensities

Our MR result indicates a negative association between CD24 and AAA risk, suggesting a potential protective role. As a cell adhesion molecule, CD24 is ubiquitously expressed but shows high prevalence in switched memory B cells. This negative correlation could potentially be linked to two primary mechanisms. First, reduced levels of CD24 are likely to limit leukocyte migration to the aortic wall, consequently mitigating the local inflammatory response, a well-established driver in AAA pathogenesis. Second, CD24 serves as an inhibitory modulator of immune responses through its interaction with specific siglec receptors^37,38^. These phosphatases inhibit downstream signaling pathways that would otherwise facilitate B cell activation and autoantibody production, processes known to exacerbate AAA^39^. Previous research corroborates CD24's immune inhibitory functions, showing that its deficiency augments humoral immune responses and raises susceptibility to autoimmune diseases^40^. Thus, elevated CD24 expression, particularly on switched memory B cells, could potentially serve as a protective mechanism by suppressing pro-inflammatory immune activities. These insights collectively contribute to the understanding that AAA development is modulated by immune-mediated mechanisms, and they also underscore the potential role of CD24 as a biomarker or therapeutic target in AAA.

In addition, higher expression of CD45 on CD8 bright T cells was causally associated with increased AAA risk. The CD45 antigen is a protein tyrosine phosphatase involved in regulation of T cell receptor signaling. The tyrosine phosphatase activity of CD45 is integral for the regulation of multiple signaling pathways, not only those associated with the T cell receptor but also with integrin and cytokine receptors^41,42^. This multi-faceted role suggests that elevated levels of CD45 could potentially have a broader impact, affecting various immune cells that are involved in the inflammatory milieu characteristic of AAA. Elevated CD45 levels may promote autoreactive T cell responses that drive AAA development. Further research is required to elucidate the precise mechanisms by which CD45 influences AAA risk.

### Morphological parameter

Our findings demonstrate that genetically predicted elevations in SSC-A on HLA-DR + NK cells and HLA-DR + T cells correspond to a reduced risk of AAA, highlighting the potential protective role of these cells.

HLA-DR is a Major Histocompatibility Complex (MHC) class II molecule that plays an integral role in antigen presentation and activation of CD4 + T cells. HLA-DR is constitutively expressed on professional antigen presenting cells like dendritic cells and B cells. However, HLA-DR can also be inducibly expressed on other cell types including NK cells and CD4 + T cells upon activation^43–45^. Recent studies have shown that a subset of NK cells expand and express HLA-DR during cytomegalovirus (CMV) infection. These HLA-DR + NK cells demonstrate enhanced cytokine production and ability to present antigens to CD4 + T cells compared to conventional NK cells^46,47^. Additionally, HLA-DR + NK cells have been found to process and present antigens through the HLA-DR pathway, suggesting they may help regulate antigen-specific CD4 + T cell responses^47^.

This association between HLA-DR expression on NK cells and a reduced AAA risk indicates that a robust presence or activity of this specific NK subset might regulate CD4 + T cell responses against AAA-associated antigens more effectively. This could potentially lead to the amplified activation and proliferation of CD4 + T cells, potentially mitigating the immune-mediated damage seen in AAA progression. Additionally, CD4 + T cells, when activated, can express HLA-DR, intensifying immune responses through direct antigen presentation. The increased expression of HLA-DR on these cells could be instrumental in modulating immune reactions related to AAA.

Our MR study provides evidence that multiple circulating immune cell phenotypes are causally implicated in AAA pathogenesis. These findings have a twofold clinical relevance. Firstly, the immune traits identified here may serve as novel therapeutic targets. Secondly, these causally associated immune traits could be examined as potential biomarkers for AAA.

### Study limitations

Our study has some limitations. Firstly, the majority of patients included in the GWAS summary data used in our research were of European descent. This discrepancy could potentially introduce biases in the estimates, thereby affecting the universal applicability of our findings. The results of this study may not be extrapolated to other racial or ethnic groups. Secondly, while our study identifies certain immune cell phenotypes as potential causal factors in AAA, the specific mechanisms by which they influence AAA risk remain unclear. Further experimental studies focusing on individual immune cell phenotypes are required to elucidate these mechanisms. Thirdly, we utilized publicly available GWAS summary statistics, which precluded conducting individual level analyses. We were unable to explore gene-environment interactions or subgroup effects. Finally, although we performed sensitivity analyses to assess and mitigate potential pleiotropy, we cannot completely rule out residual pleiotropy that may bias the causal estimates.

## Conclusions

Our MR study identifies multiple circulating immune cell phenotypes as playing causal roles in AAA development. Elucidating causal immune pathways provides vital clues to guide biomarker discovery, risk stratification, and immunomodulatory treatment innovations to improve AAA prevention and management.

### Supplementary Information

Supplementary Information.

## Data Availability

The Abdominal Aortic Aneurysm summary statistics of GWAS used in current study is publicly available (https://csg.sph.umich.edu/willer/public/AAAgen2023/). The summary statistics for each of the 731 phenotypic GWAS outcomes are publicly accessible through the GWAS Catalog (accession numbers GCST0001391 to GCST0002121).
