# Supplementary material for: Assessing the causal relationship between circulating immune cells and abdominal aortic aneurysm by bi-directional Mendelian randomization analysis
Source: Sci Rep. 2024 Jun 14;14:13733. doi: 10.1038/s41598-024-64789-9 (PMC11178833; doi:10.1038/s41598-024-64789-9)
Supplement: Supplementary file 1 — Supplementary Information. [file 41598_2024_64789_MOESM1_ESM.docx]

**Supplemental Tables**

**Supplemental Table 1.** Cohorts Contributing to the Discovery GWAS for Abdominal Aortic Aneurysm.
**Supplemental Table 2.** Details of Instrumental Variables Used for MR Analysis on the Effect Estimates of the Immunophenotypes on Abdominal Aortic Aneurysm.

**Supplemental Table 3.** Effect Estimates of the Immunophenotypes on Abdominal Aortic Aneurysm in the MR Analyses.
**Supplemental Table 4.** Test Results for Pleiotropy and Heterogeneity - Causal Effects of the Immunophenotypes on Abdominal Aortic Aneurysm.

**Supplemental Table 5.** Details of Instrumental Variables Used for MR Analysis on the Effect Estimates of Abdominal Aortic Aneurysm on the Immunophenotypes.
**Supplemental Table 6.** Effect Estimates of Abdominal Aortic Aneurysm on the Immunophenotypes in the MR Analyses.

**Supplemental Table 1.** Cohorts Contributing to the Discovery GWAS for Abdominal Aortic Aneurysm.

| **Cohort** | **Cases** | **Controls** | **Ancestry** | **Imputation panel** | **Association analysis** | **Number of variants** |
| --- | --- | --- | --- | --- | --- | --- |
| ARIC | 408 | 8554 | EUR | 1KG | SNPTEST | 20407887 |
| CHB-CVDC+DBDS | 3079 | 180236 | EUR | 1kG+HRC | SAIGE | 7489769 |
| CHIP+MGI | 1283 | 12202 | EUR | HRC | SAIGE | 20699674 |
| deCODE | 1656 | 265410 | EUR | deCODE | deCODE | 14771657 |
| DiscovEHR | 2238 | 105433 | EUR | HRC | REGENIE | 8767326 |
| eMERGE | 3092 | 15025 | EUR | HRC | PLINK | 7633402 |
| eMERGE | 119 | 603 | AFR | HRC | PLINK | 13196240 |
| HUNT | 734 | 68901 | EUR | HRC | SAIGE | 23134895 |
| Mayo VDB | 771 | 4913 | EUR | HRC | SAIGE | 15730490 |
| MVP | 17672 | 303695 | EUR | 1KG+AGR | PLINK2 | 26319140 |
| MVP | 1888 | 87728 | AFR | 1KG+AGR | PLINK2 | 38934642 |
| NZ1 | 608 | 612 | EUR | 1KG | PLINK | 7315095 |
| NZ2 | 397 | 384 | EUR | 1KG | PLINK | 7302959 |
| PMBB | 388 | 9879 | EUR | 1KG | PLINK | 6512545 |
| TABS | 52 | 483 | EUR | HRC | SAIGE | 9443602 |
| UKAGS+VIVA | 3595 | 15773 | EUR | 1KG | PLINK | 3586143 |
| UKBB | 1241 | 6276 | EUR | HRC | SNPTEST | 9884304 |

**Supplemental Table 2.** Details of Instrumental Variables Used for MR Analysis on the Effect Estimates of the Immunophenotypes on Abdominal Aortic Aneurysm.

| **Trait type** | **Exposure** | **SNP** | **Other allele** | **Effect allele** | **Beta** | **SE** | ***P* value** | **R square** | ***F*** |
| --- | --- | --- | --- | --- | --- | --- | --- | --- | --- |
| Absolute count | Activated & resting Treg AC | rs111797167 | A | G | 0.401 | 0.071 | 1.37e-08 | 0.009 | 32.35 |
| Absolute count | Activated & resting Treg AC | rs144398648 | C | T | 0.669 | 0.119 | 1.83e-08 | 0.009 | 31.78 |
| Absolute count | Activated & resting Treg AC | rs1569590 | A | G | 0.208 | 0.037 | 1.84e-08 | 0.009 | 31.78 |
| Absolute count | Activated & resting Treg AC | rs55809481 | T | C | -0.212 | 0.029 | 1.60e-13 | 0.015 | 54.79 |
| Absolute count | Activated & secreting Treg AC | rs11888944 | G | A | 0.16 | 0.028 | 9.87e-09 | 0.011 | 33.04 |
| Absolute count | Activated & secreting Treg AC | rs34873616 | C | T | 0.256 | 0.027 | 5.64e-21 | 0.030 | 89.58 |
| Absolute count | Activated & secreting Treg AC | rs61141933 | C | T | 0.258 | 0.041 | 5.39e-10 | 0.013 | 38.78 |
| Absolute count | CD11c+ monocyte AC | rs71632989 | T | G | -0.685 | 0.028 | 1.30e-124 | 0.145 | 609.96 |
| Absolute count | CD11c+ monocyte AC | rs7180804 | G | A | -0.252 | 0.03 | 8.74e-17 | 0.019 | 69.91 |
| Absolute count | CD11c+ monocyte AC | rs74341264 | A | G | -0.333 | 0.039 | 1.13e-17 | 0.020 | 74.02 |
| Absolute count | CD11c+ monocyte AC | rs865239 | A | G | -0.137 | 0.025 | 4.97e-08 | 0.008 | 29.84 |
| Absolute count | CD11c+ monocyte AC | rs9916257 | G | T | 0.146 | 0.025 | 6.72e-09 | 0.009 | 33.78 |
| Absolute count | CD14- CD16+ monocyte AC | rs2018404 | C | T | -0.271 | 0.037 | 4.69e-13 | 0.014 | 52.68 |
| Absolute count | CD14- CD16+ monocyte AC | rs2857373 | G | A | -0.349 | 0.057 | 1.12e-09 | 0.010 | 37.27 |
| Absolute count | CD14- CD16+ monocyte AC | rs28669075 | C | T | -0.166 | 0.025 | 2.44e-11 | 0.012 | 44.87 |
| Absolute count | CD14- CD16+ monocyte AC | rs4721574 | G | A | 0.26 | 0.028 | 6.98e-21 | 0.024 | 88.94 |
| Absolute count | CD14- CD16+ monocyte AC | rs709589 | C | T | -0.7 | 0.029 | 2.05e-117 | 0.135 | 571.11 |
| Absolute count | CD14- CD16+ monocyte AC | rs73367236 | G | T | 0.554 | 0.089 | 5.92e-10 | 0.010 | 38.53 |
| Absolute count | CD14- CD16- AC | rs10882701 | A | C | -0.4 | 0.024 | 4.54e-61 | 0.076 | 282.59 |
| Absolute count | CD14- CD16- AC | rs118087712 | G | A | 0.262 | 0.039 | 1.26e-11 | 0.013 | 46.18 |
| Absolute count | CD14- CD16- AC | rs141018090 | G | T | 0.557 | 0.053 | 2.14e-25 | 0.031 | 110.13 |
| Absolute count | CD14- CD16- AC | rs185198079 | C | T | 0.858 | 0.091 | 5.16e-21 | 0.025 | 89.57 |
| Absolute count | CD14- CD16- AC | rs191819784 | G | A | 0.819 | 0.073 | 8.22e-29 | 0.035 | 126.28 |
| Absolute count | CD14- CD16- AC | rs61868672 | G | A | -0.441 | 0.049 | 1.70e-19 | 0.023 | 82.49 |
| Absolute count | CD14- CD16- AC | rs7095587 | T | C | -1.021 | 0.018 | 1.00e-200 | 0.493 | 3348.13 |
| Absolute count | CD14- CD16- AC | rs7906684 | G | A | 1.177 | 0.076 | 4.08e-52 | 0.065 | 238.76 |
| Absolute count | CD20- AC | rs2596459 | G | T | 0.15 | 0.026 | 1.65e-08 | 0.009 | 32 |
| Absolute count | CD20- AC | rs34873616 | C | T | 0.234 | 0.022 | 9.09e-26 | 0.032 | 111.93 |
| Absolute count | CD20- AC | rs6707421 | A | G | 0.159 | 0.024 | 8.10e-11 | 0.012 | 42.48 |
| Absolute count | CD20- CD38- AC | rs13210358 | C | T | 0.471 | 0.071 | 4.24e-11 | 0.013 | 43.75 |
| Absolute count | CD20- CD38- AC | rs150386792 | G | A | 0.413 | 0.073 | 1.64e-08 | 0.009 | 32.01 |
| Absolute count | CD20- CD38- AC | rs181878688 | G | A | 0.524 | 0.066 | 3.88e-15 | 0.018 | 62.3 |
| Absolute count | CD20- CD38- AC | rs182904923 | T | G | 0.408 | 0.073 | 2.87e-08 | 0.009 | 30.92 |
| Absolute count | CD20- CD38- AC | rs185858489 | G | A | 0.471 | 0.069 | 1.22e-11 | 0.013 | 46.23 |
| Absolute count | CD20- CD38- AC | rs186978368 | C | T | 0.436 | 0.071 | 8.01e-10 | 0.011 | 37.96 |
| Absolute count | CD20- CD38- AC | rs34546581 | G | A | 0.202 | 0.031 | 5.06e-11 | 0.013 | 43.42 |
| Absolute count | CD25hi AC | rs11136335 | G | A | -0.165 | 0.028 | 2.20e-09 | 0.012 | 35.97 |
| Absolute count | CD25hi AC | rs1152928 | A | G | -0.823 | 0.105 | 7.27e-15 | 0.021 | 61.1 |
| Absolute count | CD25hi AC | rs11554159 | G | A | -0.374 | 0.029 | 3.14e-36 | 0.054 | 162.37 |
| Absolute count | CD25hi AC | rs139834534 | T | C | -0.794 | 0.144 | 4.18e-08 | 0.010 | 30.21 |
| Absolute count | CD25hi AC | rs145992681 | C | T | -0.745 | 0.117 | 2.73e-10 | 0.014 | 40.12 |
| Absolute count | CD25hi AC | rs148116066 | C | T | -0.486 | 0.083 | 4.69e-09 | 0.012 | 34.5 |
| Absolute count | CD25hi AC | rs148395375 | G | A | -0.858 | 0.148 | 8.60e-09 | 0.012 | 33.32 |
| Absolute count | CD25hi AC | rs1800973 | C | A | -1.218 | 0.045 | 1.72e-145 | 0.207 | 741.62 |
| Absolute count | CD25hi AC | rs192415250 | C | T | -0.812 | 0.131 | 6.73e-10 | 0.013 | 38.32 |
| Absolute count | CD25hi AC | rs2870817 | G | A | -0.629 | 0.105 | 2.06e-09 | 0.013 | 36.1 |
| Absolute count | CD25hi AC | rs445 | C | T | 0.209 | 0.034 | 1.45e-09 | 0.013 | 36.79 |
| Absolute count | CD25hi AC | rs540090564 | T | C | -0.785 | 0.123 | 2.11e-10 | 0.014 | 40.64 |
| Absolute count | CD25hi AC | rs74792658 | T | C | -0.928 | 0.09 | 2.90e-24 | 0.036 | 105.14 |
| Absolute count | CD25hi CD45RA- CD4 not Treg AC | rs3130685 | C | T | 0.189 | 0.03 | 2.56e-10 | 0.012 | 40.18 |
| Absolute count | CD25hi CD45RA- CD4 not Treg AC | rs36058358 | G | A | -0.22 | 0.038 | 6.68e-09 | 0.010 | 33.78 |
| Absolute count | CD25hi CD45RA- CD4 not Treg AC | rs4796089 | T | C | -0.21 | 0.037 | 1.90e-08 | 0.009 | 31.72 |
| Absolute count | CD28+ CD45RA- CD8br AC | rs10882701 | A | C | -0.384 | 0.027 | 1.78e-43 | 0.063 | 197.41 |
| Absolute count | CD28+ CD45RA- CD8br AC | rs1410600 | C | T | -0.233 | 0.028 | 2.19e-16 | 0.023 | 68.21 |
| Absolute count | CD28+ CD45RA- CD8br AC | rs191910319 | G | A | 0.461 | 0.077 | 1.92e-09 | 0.012 | 36.25 |
| Absolute count | CD28+ CD45RA- CD8br AC | rs2901833 | G | A | 0.27 | 0.028 | 3.43e-21 | 0.030 | 90.59 |
| Absolute count | CD28+ CD45RA- CD8br AC | rs6584027 | G | A | 1.026 | 0.021 | 1.00e-200 | 0.455 | 2433.82 |
| Absolute count | CD28+ CD45RA- CD8dim AC | rs2275848 | G | T | -0.219 | 0.04 | 4.81e-08 | 0.016 | 30.01 |
| Absolute count | CD28+ CD45RA- CD8dim AC | rs41294937 | T | C | 0.491 | 0.055 | 1.67e-18 | 0.041 | 78.63 |
| Absolute count | CD28+ CD45RA- CD8dim AC | rs709589 | C | T | -0.248 | 0.044 | 1.84e-08 | 0.017 | 31.89 |
| Absolute count | CD28- CD127- CD25++ CD8br AC | rs12921174 | T | C | -0.204 | 0.034 | 1.49e-09 | 0.012 | 36.74 |
| Absolute count | CD28- CD127- CD25++ CD8br AC | rs142607615 | C | T | -0.61 | 0.095 | 1.77e-10 | 0.014 | 40.97 |
| Absolute count | CD28- CD127- CD25++ CD8br AC | rs2949661 | C | T | 0.644 | 0.025 | 1.66e-133 | 0.187 | 671.36 |
| Absolute count | CD28- CD127- CD25++ CD8br AC | rs4657649 | T | C | 0.539 | 0.061 | 1.51e-18 | 0.026 | 78.24 |
| Absolute count | CD28- CD127- CD25++ CD8br AC | rs61814872 | C | T | 0.313 | 0.044 | 1.98e-12 | 0.017 | 49.91 |
| Absolute count | CD28- CD127- CD25++ CD8br AC | rs79179998 | G | A | 0.23 | 0.034 | 1.23e-11 | 0.016 | 46.27 |
| Absolute count | CD28- DN (CD4-CD8-) AC | rs112266065 | C | T | -0.942 | 0.115 | 3.63e-16 | 0.018 | 67.03 |
| Absolute count | CD28- DN (CD4-CD8-) AC | rs114919524 | A | C | -1.13 | 0.106 | 5.16e-26 | 0.030 | 112.94 |
| Absolute count | CD28- DN (CD4-CD8-) AC | rs117119882 | C | A | -0.861 | 0.115 | 1.04e-13 | 0.015 | 55.71 |
| Absolute count | CD28- DN (CD4-CD8-) AC | rs144365928 | A | G | -0.713 | 0.077 | 4.51e-20 | 0.023 | 85.12 |
| Absolute count | CD28- DN (CD4-CD8-) AC | rs148516965 | C | T | -0.541 | 0.056 | 6.82e-22 | 0.025 | 93.61 |
| Absolute count | CD28- DN (CD4-CD8-) AC | rs149806587 | A | G | -0.539 | 0.057 | 6.70e-21 | 0.024 | 88.99 |
| Absolute count | CD28- DN (CD4-CD8-) AC | rs184205740 | C | T | -1.191 | 0.116 | 1.79e-24 | 0.028 | 105.72 |
| Absolute count | CD28- DN (CD4-CD8-) AC | rs185430947 | A | G | -1.001 | 0.108 | 2.91e-20 | 0.023 | 86.02 |
| Absolute count | CD28- DN (CD4-CD8-) AC | rs188216171 | G | A | -0.858 | 0.109 | 5.48e-15 | 0.017 | 61.57 |
| Absolute count | CD28- DN (CD4-CD8-) AC | rs189799494 | G | T | -0.655 | 0.05 | 4.51e-38 | 0.044 | 170.19 |
| Absolute count | CD28- DN (CD4-CD8-) AC | rs191363479 | C | T | -1 | 0.109 | 7.14e-20 | 0.022 | 84.12 |
| Absolute count | CD28- DN (CD4-CD8-) AC | rs193039847 | G | A | -1.325 | 0.086 | 1.84e-51 | 0.060 | 235 |
| Absolute count | CD28- DN (CD4-CD8-) AC | rs4657041 | T | C | -0.15 | 0.027 | 2.29e-08 | 0.009 | 31.34 |
| Absolute count | CD28- DN (CD4-CD8-) AC | rs532866318 | T | G | -1.355 | 0.119 | 1.96e-29 | 0.034 | 129.15 |
| Absolute count | CD28- DN (CD4-CD8-) AC | rs535059510 | C | T | -0.916 | 0.117 | 7.92e-15 | 0.016 | 60.82 |
| Absolute count | CD28- DN (CD4-CD8-) AC | rs539241708 | C | T | -1.161 | 0.085 | 1.26e-41 | 0.049 | 187.39 |
| Absolute count | CD28- DN (CD4-CD8-) AC | rs556260593 | A | C | -1.004 | 0.105 | 2.55e-21 | 0.024 | 90.86 |
| Absolute count | CD28- DN (CD4-CD8-) AC | rs557577522 | G | A | -0.758 | 0.071 | 1.53e-26 | 0.031 | 115.41 |
| Absolute count | CD28- DN (CD4-CD8-) AC | rs568374507 | C | T | -0.566 | 0.058 | 6.71e-22 | 0.025 | 93.65 |
| Absolute count | CD28- DN (CD4-CD8-) AC | rs5758649 | C | T | -0.578 | 0.045 | 1.63e-37 | 0.044 | 167.5 |
| Absolute count | CD28- DN (CD4-CD8-) AC | rs73165126 | C | T | -1.239 | 0.034 | 1.00e-200 | 0.271 | 1357.41 |
| Absolute count | CD28- DN (CD4-CD8-) AC | rs73888140 | G | A | -0.573 | 0.06 | 1.37e-21 | 0.025 | 92.19 |
| Absolute count | CD28- DN (CD4-CD8-) AC | rs78806227 | C | T | -0.967 | 0.12 | 9.87e-16 | 0.017 | 64.98 |
| Absolute count | CD28- DN (CD4-CD8-) AC | rs79085286 | T | C | -0.386 | 0.053 | 4.54e-13 | 0.014 | 52.76 |
| Absolute count | CD33- HLA DR- AC | rs3087456 | G | A | -0.452 | 0.027 | 9.23e-61 | 0.072 | 280.72 |
| Absolute count | CD33- HLA DR- AC | rs72779785 | A | G | -0.193 | 0.032 | 2.31e-09 | 0.010 | 35.85 |
| Absolute count | CD33- HLA DR- AC | rs9378213 | G | T | -0.216 | 0.03 | 1.11e-12 | 0.014 | 50.99 |
| Absolute count | CD33br HLA DR+ CD14dim AC | rs11657759 | A | C | -0.135 | 0.024 | 1.81e-08 | 0.009 | 31.8 |
| Absolute count | CD33br HLA DR+ CD14dim AC | rs12874404 | A | G | 0.211 | 0.026 | 8.09e-16 | 0.018 | 65.37 |
| Absolute count | CD33br HLA DR+ CD14dim AC | rs139370814 | C | T | 0.566 | 0.086 | 6.24e-11 | 0.012 | 42.97 |
| Absolute count | CD33dim HLA DR+ CD11b- AC | rs11625742 | C | T | -5.386 | 0.963 | 2.41e-08 | 0.009 | 31.26 |
| Absolute count | CD33dim HLA DR+ CD11b- AC | rs117522565 | G | A | -29.76 | 4.659 | 1.93e-10 | 0.012 | 40.78 |
| Absolute count | CD33dim HLA DR+ CD11b- AC | rs1199676 | T | C | -38.78 | 4.679 | 1.63e-16 | 0.020 | 68.65 |
| Absolute count | CD33dim HLA DR+ CD11b- AC | rs12668413 | A | G | -13.58 | 2.416 | 2.07e-08 | 0.009 | 31.58 |
| Absolute count | CD33dim HLA DR+ CD11b- AC | rs130071 | G | A | 1.086 | 0.169 | 1.37e-10 | 0.012 | 41.47 |
| Absolute count | CD33dim HLA DR+ CD11b- AC | rs13087457 | A | G | -29.76 | 4.659 | 1.93e-10 | 0.012 | 40.78 |
| Absolute count | CD33dim HLA DR+ CD11b- AC | rs144448823 | A | C | -29.76 | 4.659 | 1.93e-10 | 0.012 | 40.78 |
| Absolute count | CD33dim HLA DR+ CD11b- AC | rs146686061 | T | C | -20.98 | 3.305 | 2.47e-10 | 0.012 | 40.27 |
| Absolute count | CD33dim HLA DR+ CD11b- AC | rs147133204 | A | G | -28.55 | 3.431 | 1.23e-16 | 0.020 | 69.2 |
| Absolute count | CD33dim HLA DR+ CD11b- AC | rs150498189 | C | T | -29.76 | 4.659 | 1.93e-10 | 0.012 | 40.78 |
| Absolute count | CD33dim HLA DR+ CD11b- AC | rs184982628 | G | T | -13.76 | 2.426 | 1.54e-08 | 0.009 | 32.15 |
| Absolute count | CD33dim HLA DR+ CD11b- AC | rs192667895 | T | C | -26.3 | 4.684 | 2.12e-08 | 0.009 | 31.51 |
| Absolute count | CD33dim HLA DR+ CD11b- AC | rs2615702 | C | T | -11.3 | 2.015 | 2.21e-08 | 0.009 | 31.43 |
| Absolute count | CD33dim HLA DR+ CD11b- AC | rs4854532 | A | C | -22.52 | 4.022 | 2.32e-08 | 0.009 | 31.33 |
| Absolute count | CD33dim HLA DR+ CD11b- AC | rs56124337 | T | C | -29.76 | 4.659 | 1.93e-10 | 0.012 | 40.78 |
| Absolute count | CD33dim HLA DR+ CD11b- AC | rs58905133 | A | G | 1.152 | 0.131 | 2.08e-18 | 0.022 | 77.41 |
| Absolute count | CD33dim HLA DR+ CD11b- AC | rs61871299 | A | C | -16.36 | 2.789 | 4.84e-09 | 0.010 | 34.39 |
| Absolute count | CD33dim HLA DR+ CD11b- AC | rs6769348 | T | C | -25.41 | 2.761 | 5.88e-20 | 0.024 | 84.65 |
| Absolute count | CD33dim HLA DR+ CD11b- AC | rs74960590 | A | C | -29.76 | 4.659 | 1.93e-10 | 0.012 | 40.78 |
| Absolute count | CD33dim HLA DR+ CD11b- AC | rs78871360 | C | T | -29.76 | 4.659 | 1.93e-10 | 0.012 | 40.78 |
| Absolute count | CD33dim HLA DR+ CD11b- AC | rs9882785 | T | C | -18.32 | 3.03 | 1.64e-09 | 0.011 | 36.54 |
| Absolute count | CD39+ activated Treg AC | rs532635130 | C | T | 0.369 | 0.065 | 1.72e-08 | 0.009 | 31.92 |
| Absolute count | CD39+ activated Treg AC | rs71632979 | A | G | -0.712 | 0.027 | 9.97e-142 | 0.164 | 703.06 |
| Absolute count | CD39+ activated Treg AC | rs71639910 | G | A | -0.275 | 0.05 | 3.27e-08 | 0.008 | 30.67 |
| Absolute count | CD39+ activated Treg AC | rs7180804 | G | A | -0.236 | 0.03 | 2.07e-15 | 0.017 | 63.51 |
| Absolute count | CD39+ activated Treg AC | rs74341264 | A | G | -0.342 | 0.038 | 2.53e-19 | 0.022 | 81.66 |
| Absolute count | CD39+ secreting Treg AC | rs10882701 | A | C | -0.359 | 0.024 | 1.08e-47 | 0.059 | 217.07 |
| Absolute count | CD39+ secreting Treg AC | rs11589644 | C | T | 0.656 | 0.115 | 1.39e-08 | 0.009 | 32.31 |
| Absolute count | CD39+ secreting Treg AC | rs11890666 | G | A | -0.161 | 0.028 | 7.94e-09 | 0.010 | 33.44 |
| Absolute count | CD39+ secreting Treg AC | rs12712610 | A | G | 0.201 | 0.027 | 1.83e-13 | 0.016 | 54.62 |
| Absolute count | CD39+ secreting Treg AC | rs146265359 | T | C | 0.313 | 0.046 | 1.82e-11 | 0.013 | 45.44 |
| Absolute count | CD39+ secreting Treg AC | rs185198079 | C | T | 0.745 | 0.092 | 6.49e-16 | 0.019 | 65.88 |
| Absolute count | CD39+ secreting Treg AC | rs191819784 | G | A | 0.84 | 0.074 | 1.37e-29 | 0.036 | 129.94 |
| Absolute count | CD39+ secreting Treg AC | rs1923692 | T | G | -0.55 | 0.053 | 6.37e-25 | 0.030 | 107.92 |
| Absolute count | CD39+ secreting Treg AC | rs35103389 | C | T | 0.248 | 0.045 | 3.07e-08 | 0.009 | 30.79 |
| Absolute count | CD39+ secreting Treg AC | rs61868672 | G | A | -0.324 | 0.05 | 7.94e-11 | 0.012 | 42.52 |
| Absolute count | CD39+ secreting Treg AC | rs7094382 | G | A | 0.767 | 0.021 | 1.00e-200 | 0.274 | 1297.27 |
| Absolute count | CD39+ secreting Treg AC | rs77745909 | A | G | 0.82 | 0.115 | 1.15e-12 | 0.015 | 50.95 |
| Absolute count | CD39+ secreting Treg AC | rs7906684 | G | A | 1.108 | 0.077 | 1.77e-45 | 0.057 | 206.08 |
| Absolute count | CD4 Treg AC | rs12598451 | G | A | -0.378 | 0.031 | 9.38e-33 | 0.047 | 145.47 |
| Absolute count | CD4 Treg AC | rs139174173 | C | A | 0.498 | 0.079 | 3.62e-10 | 0.013 | 39.54 |
| Absolute count | CD4 Treg AC | rs56199187 | T | C | 0.476 | 0.031 | 1.90e-51 | 0.074 | 236.58 |
| Absolute count | CD4 Treg AC | rs805288 | C | T | -0.18 | 0.029 | 8.44e-10 | 0.013 | 37.86 |
| Absolute count | CD4 Treg AC | rs885947 | G | A | 0.261 | 0.047 | 4.17e-08 | 0.010 | 30.2 |
| Absolute count | CD4 Treg AC | rs9268628 | A | C | -0.3 | 0.03 | 1.72e-23 | 0.033 | 101.42 |
| Absolute count | CD45RA+ CD28- CD8br AC | rs11687597 | A | G | 0.164 | 0.03 | 4.86e-08 | 0.008 | 29.87 |
| Absolute count | CD45RA+ CD28- CD8br AC | rs17405194 | C | T | 0.586 | 0.102 | 1.15e-08 | 0.009 | 32.69 |
| Absolute count | CD45RA+ CD28- CD8br AC | rs7840395 | A | G | -0.141 | 0.024 | 8.25e-09 | 0.009 | 33.33 |
| Absolute count | CD45RA- CD28- CD8br AC | rs12199079 | T | G | 0.2 | 0.028 | 1.17e-12 | 0.017 | 50.98 |
| Absolute count | CD45RA- CD28- CD8br AC | rs139795227 | A | C | -0.573 | 0.064 | 8.24e-19 | 0.027 | 79.47 |
| Absolute count | CD45RA- CD28- CD8br AC | rs6787493 | T | C | 0.406 | 0.045 | 4.79e-19 | 0.027 | 80.57 |
| Absolute count | CD45RA- CD28- CD8br AC | rs71632979 | A | G | 0.332 | 0.031 | 1.06e-25 | 0.038 | 111.92 |
| Absolute count | CD45RA- CD4+ AC | rs117147408 | A | G | 0.506 | 0.081 | 4.93e-10 | 0.012 | 38.94 |
| Absolute count | CD45RA- CD4+ AC | rs144837535 | T | C | 0.476 | 0.077 | 6.25e-10 | 0.012 | 38.45 |
| Absolute count | CD45RA- CD4+ AC | rs150386792 | G | A | 0.443 | 0.08 | 3.67e-08 | 0.010 | 30.45 |
| Absolute count | CD45RA- CD4+ AC | rs181878688 | G | A | 0.537 | 0.073 | 2.76e-13 | 0.017 | 53.8 |
| Absolute count | CD45RA- CD4+ AC | rs186978368 | C | T | 0.446 | 0.077 | 9.55e-09 | 0.011 | 33.09 |
| Absolute count | CD62L- CD86+ myeloid DC AC | rs10882666 | G | A | 1.015 | 0.019 | 1.00e-200 | 0.456 | 2882.41 |
| Absolute count | CD62L- CD86+ myeloid DC AC | rs10882701 | A | C | -0.396 | 0.025 | 8.03e-56 | 0.070 | 256.73 |
| Absolute count | CD62L- CD86+ myeloid DC AC | rs118087712 | G | A | 0.244 | 0.04 | 1.18e-09 | 0.011 | 37.18 |
| Absolute count | CD62L- CD86+ myeloid DC AC | rs185198079 | C | T | 0.864 | 0.094 | 5.62e-20 | 0.024 | 84.76 |
| Absolute count | CD62L- CD86+ myeloid DC AC | rs191819784 | G | A | 0.769 | 0.076 | 5.97e-24 | 0.029 | 103.33 |
| Absolute count | CD62L- CD86+ myeloid DC AC | rs61868672 | G | A | -0.4 | 0.05 | 2.97e-15 | 0.018 | 62.84 |
| Absolute count | CD62L- CD86+ myeloid DC AC | rs7906684 | G | A | 0.997 | 0.08 | 3.92e-35 | 0.044 | 156.37 |
| Absolute count | CD62L- CD86+ myeloid DC AC | rs7918541 | A | G | -0.517 | 0.055 | 1.53e-20 | 0.025 | 87.39 |
| Absolute count | CD62L- HLA DR++ monocyte AC | rs138030460 | C | T | -0.304 | 0.055 | 2.90e-08 | 0.008 | 30.88 |
| Absolute count | CD62L- HLA DR++ monocyte AC | rs140684757 | C | T | 0.464 | 0.073 | 2.78e-10 | 0.011 | 40.04 |
| Absolute count | CD62L- HLA DR++ monocyte AC | rs147487472 | C | A | 0.426 | 0.061 | 4.00e-12 | 0.013 | 48.42 |
| Absolute count | CD62L- HLA DR++ monocyte AC | rs3793662 | C | T | -0.19 | 0.029 | 1.11e-10 | 0.011 | 41.82 |
| Absolute count | CD62L- HLA DR++ monocyte AC | rs62626323 | C | A | 0.585 | 0.04 | 1.32e-47 | 0.056 | 216.14 |
| Absolute count | CD62L- HLA DR++ monocyte AC | rs709589 | C | T | -0.183 | 0.031 | 5.98e-09 | 0.009 | 33.99 |
| Absolute count | CD62L- monocyte AC | rs12712610 | A | G | -0.182 | 0.024 | 1.23e-14 | 0.017 | 60.02 |
| Absolute count | CD62L- monocyte AC | rs2463509 | C | T | 1.154 | 0.203 | 1.46e-08 | 0.009 | 32.23 |
| Absolute count | CD62L- monocyte AC | rs34873616 | C | T | -0.241 | 0.021 | 2.06e-29 | 0.036 | 129.12 |
| Absolute count | CD62L- monocyte AC | rs77634072 | C | T | -0.418 | 0.076 | 4.64e-08 | 0.009 | 29.98 |
| Absolute count | CD62L- myeloid DC AC | rs13191523 | A | G | 0.277 | 0.046 | 1.73e-09 | 0.012 | 36.45 |
| Absolute count | CD62L- myeloid DC AC | rs3129765 | A | G | -0.216 | 0.033 | 1.02e-10 | 0.013 | 42.02 |
| Absolute count | CD62L- myeloid DC AC | rs7745305 | T | C | 0.206 | 0.038 | 4.51e-08 | 0.010 | 30.06 |
| Absolute count | CD66b++ myeloid cell AC | rs139249541 | A | G | 0.415 | 0.07 | 2.69e-09 | 0.010 | 35.55 |
| Absolute count | CD66b++ myeloid cell AC | rs144837535 | T | C | 0.425 | 0.068 | 5.80e-10 | 0.010 | 38.57 |
| Absolute count | CD66b++ myeloid cell AC | rs150386792 | G | A | 0.411 | 0.072 | 1.17e-08 | 0.009 | 32.68 |
| Absolute count | CD66b++ myeloid cell AC | rs181878688 | G | A | 0.476 | 0.066 | 4.99e-13 | 0.014 | 52.55 |
| Absolute count | CD66b++ myeloid cell AC | rs186978368 | C | T | 0.411 | 0.07 | 5.05e-09 | 0.009 | 34.32 |
| Absolute count | CD66b++ myeloid cell AC | rs7569868 | C | T | 0.233 | 0.03 | 2.49e-14 | 0.016 | 58.56 |
| Absolute count | CD66b++ myeloid cell AC | rs9896155 | A | G | 0.141 | 0.025 | 2.39e-08 | 0.008 | 31.28 |
| Absolute count | CD86+ myeloid DC AC | rs2228015 | T | C | 0.683 | 0.068 | 3.72e-23 | 0.033 | 99.87 |
| Absolute count | CD86+ myeloid DC AC | rs5020353 | A | G | -0.274 | 0.029 | 1.65e-21 | 0.031 | 92.12 |
| Absolute count | CD86+ myeloid DC AC | rs61968809 | G | A | 0.256 | 0.047 | 4.36e-08 | 0.010 | 30.13 |
| Absolute count | CD86+ myeloid DC AC | rs75983909 | C | T | 0.599 | 0.107 | 2.09e-08 | 0.011 | 31.58 |
| Absolute count | CD8br AC | rs11158593 | G | A | 0.198 | 0.027 | 6.89e-13 | 0.018 | 52.01 |
| Absolute count | CD8br AC | rs12298133 | C | T | 0.248 | 0.036 | 1.11e-11 | 0.016 | 46.45 |
| Absolute count | CD8br AC | rs17205661 | G | A | -0.263 | 0.044 | 2.78e-09 | 0.012 | 35.52 |
| Absolute count | CD8br AC | rs2844608 | C | T | 0.199 | 0.032 | 3.90e-10 | 0.013 | 39.4 |
| Absolute count | CD8br AC | rs79335220 | T | G | -0.521 | 0.09 | 9.23e-09 | 0.011 | 33.16 |
| Absolute count | CD8dim NKT AC | rs111559749 | C | T | -0.267 | 0.047 | 1.27e-08 | 0.009 | 32.52 |
| Absolute count | CD8dim NKT AC | rs12478924 | T | C | 0.204 | 0.028 | 7.21e-13 | 0.015 | 51.86 |
| Absolute count | CD8dim NKT AC | rs4959028 | A | G | 0.27 | 0.032 | 9.05e-17 | 0.020 | 69.88 |
| Absolute count | CD8dim NKT AC | rs9271376 | G | A | -0.267 | 0.033 | 1.17e-15 | 0.019 | 64.7 |
| Absolute count | CD8dim NKT AC | rs9501550 | A | C | 0.303 | 0.047 | 2.06e-10 | 0.012 | 40.63 |
| Absolute count | CM CD4+ AC | rs10905719 | G | A | 0.184 | 0.032 | 7.06e-09 | 0.010 | 33.67 |
| Absolute count | CM CD4+ AC | rs11124653 | C | A | -0.214 | 0.026 | 5.23e-16 | 0.019 | 66.35 |
| Absolute count | CM CD4+ AC | rs112505169 | C | T | 0.796 | 0.116 | 8.13e-12 | 0.014 | 47.01 |
| Absolute count | CM CD4+ AC | rs11589644 | C | T | 0.692 | 0.119 | 6.88e-09 | 0.010 | 33.75 |
| Absolute count | CM CD4+ AC | rs12712610 | A | G | 0.261 | 0.028 | 1.53e-20 | 0.025 | 87.4 |
| Absolute count | CM CD8br AC | rs12219630 | C | T | -0.189 | 0.025 | 3.18e-14 | 0.016 | 58.04 |
| Absolute count | CM CD8br AC | rs1887027 | C | T | -0.424 | 0.027 | 8.06e-53 | 0.062 | 241.54 |
| Absolute count | CM CD8br AC | rs3793662 | C | T | -0.159 | 0.029 | 3.75e-08 | 0.008 | 30.4 |
| Absolute count | DN (CD4-CD8-) AC | rs1801274 | A | G | -0.239 | 0.026 | 6.57e-20 | 0.023 | 84.37 |
| Absolute count | DN (CD4-CD8-) AC | rs184012084 | G | A | 0.711 | 0.089 | 1.61e-15 | 0.018 | 64.04 |
| Absolute count | DN (CD4-CD8-) AC | rs41314914 | G | A | 0.232 | 0.04 | 7.69e-09 | 0.009 | 33.49 |
| Absolute count | EM DN (CD4-CD8-) AC | rs12212931 | T | G | 0.223 | 0.032 | 2.33e-12 | 0.013 | 49.5 |
| Absolute count | EM DN (CD4-CD8-) AC | rs144398648 | C | T | 0.698 | 0.119 | 5.31e-09 | 0.009 | 34.22 |
| Absolute count | EM DN (CD4-CD8-) AC | rs30003 | T | C | -0.179 | 0.029 | 1.42e-09 | 0.010 | 36.81 |
| Absolute count | EM DN (CD4-CD8-) AC | rs4939384 | A | G | -0.201 | 0.025 | 7.90e-16 | 0.018 | 65.45 |
| Absolute count | EM DN (CD4-CD8-) AC | rs62405562 | A | G | 0.192 | 0.029 | 2.46e-11 | 0.012 | 44.84 |
| Absolute count | EM DN (CD4-CD8-) AC | rs72836542 | C | T | 0.152 | 0.027 | 1.64e-08 | 0.009 | 32 |
| Absolute count | EM DN (CD4-CD8-) AC | rs9270560 | T | C | -0.179 | 0.03 | 1.55e-09 | 0.010 | 36.62 |
| Absolute count | HLA DR+ NK AC | rs143032729 | A | G | 0.414 | 0.075 | 3.97e-08 | 0.009 | 30.28 |
| Absolute count | HLA DR+ NK AC | rs56199187 | T | C | -0.755 | 0.028 | 2.11e-144 | 0.175 | 721.61 |
| Absolute count | HLA DR+ NK AC | rs6537835 | G | A | -0.207 | 0.037 | 2.54e-08 | 0.009 | 31.16 |
| Absolute count | HLA DR+ NK AC | rs74341264 | A | G | 0.383 | 0.04 | 1.43e-21 | 0.026 | 92.18 |
| Absolute count | HLA DR+ T cell AC | rs11961777 | T | C | 0.445 | 0.056 | 2.31e-15 | 0.017 | 63.29 |
| Absolute count | HLA DR+ T cell AC | rs1800973 | C | A | -0.315 | 0.046 | 7.74e-12 | 0.013 | 47.13 |
| Absolute count | HLA DR+ T cell AC | rs9270585 | C | T | -0.587 | 0.028 | 5.56e-92 | 0.108 | 438.16 |
| Absolute count | HLA DR+ T cell AC | rs9469245 | C | T | -0.261 | 0.028 | 2.56e-20 | 0.023 | 86.33 |
| Absolute count | HSC AC | rs111534725 | G | T | 0.731 | 0.065 | 4.66e-29 | 0.036 | 127.43 |
| Absolute count | HSC AC | rs141715056 | G | T | -0.439 | 0.076 | 7.34e-09 | 0.010 | 33.59 |
| Absolute count | HSC AC | rs151158664 | C | T | 1.144 | 0.088 | 8.90e-38 | 0.047 | 169.09 |
| Absolute count | HSC AC | rs17111341 | T | C | 0.624 | 0.052 | 9.80e-33 | 0.040 | 144.89 |
| Absolute count | HSC AC | rs185198079 | C | T | 0.778 | 0.094 | 2.55e-16 | 0.019 | 67.75 |
| Absolute count | HSC AC | rs190891675 | C | T | 1.09 | 0.113 | 6.87e-22 | 0.027 | 93.65 |
| Absolute count | HSC AC | rs1924699 | G | T | -0.319 | 0.057 | 2.11e-08 | 0.009 | 31.52 |
| Absolute count | HSC AC | rs35302565 | T | C | 0.266 | 0.043 | 4.88e-10 | 0.011 | 38.91 |
| Absolute count | HSC AC | rs546695501 | G | T | 0.696 | 0.122 | 1.30e-08 | 0.009 | 32.48 |
| Absolute count | HSC AC | rs7067762 | T | C | 0.807 | 0.022 | 1.00e-200 | 0.279 | 1331.43 |
| Absolute count | HSC AC | rs72812685 | G | A | 0.646 | 0.059 | 1.13e-27 | 0.034 | 120.88 |
| Absolute count | HSC AC | rs7906684 | G | A | 1.051 | 0.08 | 1.20e-38 | 0.048 | 173.06 |
| Absolute count | IgD+ AC | rs12930660 | G | A | 0.298 | 0.028 | 1.93e-26 | 0.039 | 115.42 |
| Absolute count | IgD+ AC | rs1801274 | A | G | 0.276 | 0.029 | 1.20e-20 | 0.030 | 88.11 |
| Absolute count | IgD+ AC | rs9932817 | T | G | -0.168 | 0.028 | 3.73e-09 | 0.012 | 34.97 |
| Absolute count | IgD+ CD24- AC | rs139249541 | A | G | 0.481 | 0.07 | 8.63e-12 | 0.013 | 46.9 |
| Absolute count | IgD+ CD24- AC | rs144837535 | T | C | 0.481 | 0.069 | 3.90e-12 | 0.013 | 48.47 |
| Absolute count | IgD+ CD24- AC | rs150386792 | G | A | 0.468 | 0.072 | 1.25e-10 | 0.011 | 41.59 |
| Absolute count | IgD+ CD24- AC | rs181878688 | G | A | 0.554 | 0.066 | 7.70e-17 | 0.019 | 70.12 |
| Absolute count | IgD+ CD24- AC | rs182904923 | T | G | 0.461 | 0.072 | 1.96e-10 | 0.011 | 40.71 |
| Absolute count | IgD+ CD24- AC | rs186978368 | C | T | 0.428 | 0.071 | 1.60e-09 | 0.010 | 36.58 |
| Absolute count | IgD+ CD24- AC | rs62432266 | G | T | 0.328 | 0.052 | 2.41e-10 | 0.011 | 40.29 |
| Absolute count | IgD+ CD24- AC | rs9916629 | T | C | 0.134 | 0.024 | 2.41e-08 | 0.008 | 31.24 |
| Absolute count | IgD+ CD38- AC | rs71632989 | T | G | -0.698 | 0.027 | 2.05e-136 | 0.159 | 674.19 |
| Absolute count | IgD+ CD38- AC | rs71639910 | G | A | -0.302 | 0.049 | 1.15e-09 | 0.010 | 37.22 |
| Absolute count | IgD+ CD38- AC | rs7180804 | G | A | -0.224 | 0.03 | 5.28e-14 | 0.016 | 57.08 |
| Absolute count | IgD+ CD38- AC | rs74341264 | A | G | -0.339 | 0.038 | 5.43e-19 | 0.022 | 80.11 |
| Absolute count | IgD+ CD38br AC | rs10748726 | A | G | 0.168 | 0.03 | 3.54e-08 | 0.009 | 30.51 |
| Absolute count | IgD+ CD38br AC | rs10882573 | G | A | 0.444 | 0.04 | 4.50e-28 | 0.034 | 122.76 |
| Absolute count | IgD+ CD38br AC | rs113594239 | C | T | 0.815 | 0.063 | 4.73e-37 | 0.046 | 165.54 |
| Absolute count | IgD+ CD38br AC | rs141715056 | G | T | -0.408 | 0.073 | 2.45e-08 | 0.009 | 31.23 |
| Absolute count | IgD+ CD38br AC | rs145557266 | A | G | 0.675 | 0.086 | 7.61e-15 | 0.017 | 60.94 |
| Absolute count | IgD+ CD38br AC | rs145997882 | G | A | -0.444 | 0.076 | 4.89e-09 | 0.010 | 34.4 |
| Absolute count | IgD+ CD38br AC | rs188572647 | T | C | 1.131 | 0.076 | 3.14e-48 | 0.060 | 219.42 |
| Absolute count | IgD+ CD38br AC | rs191459120 | G | T | 0.456 | 0.067 | 8.56e-12 | 0.013 | 46.93 |
| Absolute count | IgD+ CD38br AC | rs191819784 | G | A | 0.765 | 0.073 | 2.78e-25 | 0.031 | 109.61 |
| Absolute count | IgD+ CD38br AC | rs4918973 | A | G | 0.783 | 0.022 | 1.00e-200 | 0.275 | 1301.02 |
| Absolute count | IgD+ CD38br AC | rs79595377 | T | C | 0.506 | 0.082 | 9.28e-10 | 0.011 | 37.66 |
| Absolute count | IgD+ CD38dim AC | rs10882701 | A | C | -0.454 | 0.026 | 4.05e-64 | 0.093 | 300.1 |
| Absolute count | IgD+ CD38dim AC | rs12778797 | T | C | 0.305 | 0.028 | 1.54e-27 | 0.040 | 120.62 |
| Absolute count | IgD+ CD38dim AC | rs1410600 | C | T | -0.264 | 0.028 | 1.82e-21 | 0.031 | 91.9 |
| Absolute count | IgD+ CD38dim AC | rs145557266 | A | G | 0.569 | 0.096 | 3.84e-09 | 0.012 | 34.9 |
| Absolute count | IgD+ CD38dim AC | rs180781725 | A | C | 0.609 | 0.103 | 3.38e-09 | 0.012 | 35.12 |
| Absolute count | IgD+ CD38dim AC | rs186687550 | T | C | -0.446 | 0.081 | 3.40e-08 | 0.010 | 30.6 |
| Absolute count | IgD+ CD38dim AC | rs1923692 | T | G | -0.441 | 0.059 | 1.06e-13 | 0.019 | 55.76 |
| Absolute count | IgD+ CD38dim AC | rs7086986 | A | G | 1.177 | 0.017 | 1.00e-200 | 0.632 | 5017.83 |
| Absolute count | IgD- CD24- AC | rs112431762 | G | A | 0.65 | 0.073 | 1.75e-18 | 0.046 | 78.74 |
| Absolute count | IgD- CD24- AC | rs11882720 | T | G | -0.448 | 0.068 | 7.43e-11 | 0.026 | 42.91 |
| Absolute count | IgD- CD24- AC | rs140923781 | C | T | -0.677 | 0.102 | 4.88e-11 | 0.026 | 43.79 |
| Absolute count | IgD- CD24- AC | rs1801274 | A | G | -0.258 | 0.039 | 3.92e-11 | 0.026 | 44.22 |
| Absolute count | IgD- CD24- AC | rs2459145 | T | C | 1.44 | 0.207 | 5.16e-12 | 0.029 | 48.24 |
| Absolute count | IgD- CD24- AC | rs3865444 | C | A | -1.139 | 0.034 | 2.38e-191 | 0.413 | 1149.09 |
| Absolute count | IgD- CD24- AC | rs558625031 | G | A | -0.675 | 0.081 | 2.01e-16 | 0.041 | 68.95 |
| Absolute count | IgD- CD24- AC | rs7351079 | A | G | -0.39 | 0.04 | 8.18e-22 | 0.055 | 94.7 |
| Absolute count | IgD- CD24- AC | rs73932869 | G | A | -0.856 | 0.152 | 1.89e-08 | 0.019 | 31.9 |
| Absolute count | IgD- CD24- AC | rs75773078 | G | A | -0.469 | 0.067 | 3.89e-12 | 0.029 | 48.86 |
| Absolute count | IgD- CD24- AC | rs79976869 | A | G | -1.003 | 0.143 | 3.22e-12 | 0.029 | 49.2 |
| Absolute count | IgD- CD27- AC | rs10919058 | G | T | 0.207 | 0.037 | 3.72e-08 | 0.009 | 30.4 |
| Absolute count | IgD- CD27- AC | rs115901521 | G | A | 0.258 | 0.043 | 1.88e-09 | 0.011 | 36.26 |
| Absolute count | IgD- CD27- AC | rs3845548 | C | T | 0.908 | 0.027 | 1.00e-200 | 0.243 | 1094.65 |
| Absolute count | IgD- CD27- AC | rs532635130 | C | T | -0.374 | 0.068 | 4.52e-08 | 0.009 | 30.03 |
| Absolute count | IgD- CD27- AC | rs74341264 | A | G | 0.45 | 0.04 | 2.65e-29 | 0.036 | 128.63 |
| Absolute count | IgD- CD27- AC | rs79918451 | T | C | -0.376 | 0.065 | 6.27e-09 | 0.010 | 33.89 |
| Absolute count | IgD- CD38- AC | rs4796089 | T | C | -0.203 | 0.033 | 1.35e-09 | 0.011 | 36.93 |
| Absolute count | IgD- CD38- AC | rs6904670 | A | G | -0.146 | 0.026 | 3.34e-08 | 0.009 | 30.62 |
| Absolute count | IgD- CD38- AC | rs7728865 | G | A | -0.217 | 0.038 | 9.95e-09 | 0.010 | 33.01 |
| Absolute count | IgD- CD38br AC | rs117147408 | A | G | 0.652 | 0.082 | 3.03e-15 | 0.020 | 62.84 |
| Absolute count | IgD- CD38br AC | rs150386792 | G | A | 0.779 | 0.081 | 1.32e-21 | 0.029 | 92.48 |
| Absolute count | IgD- CD38br AC | rs181878688 | G | A | 0.915 | 0.073 | 7.58e-35 | 0.048 | 155.33 |
| Absolute count | IgD- CD38br AC | rs185858489 | G | A | 0.702 | 0.078 | 2.90e-19 | 0.026 | 81.5 |
| Absolute count | IgD- CD38br AC | rs186978368 | C | T | 0.673 | 0.078 | 1.50e-17 | 0.023 | 73.53 |
| Absolute count | IgD- CD38br AC | rs188738981 | C | T | -0.514 | 0.088 | 6.34e-09 | 0.011 | 33.89 |
| Absolute count | IgD- CD38br AC | rs557204100 | A | G | 0.569 | 0.096 | 2.82e-09 | 0.011 | 35.49 |
| Absolute count | IgD- CD38br AC | rs57245661 | A | C | 0.724 | 0.101 | 1.04e-12 | 0.016 | 51.21 |
| Absolute count | IgD- CD38br AC | rs62432266 | G | T | 0.387 | 0.058 | 2.12e-11 | 0.014 | 45.17 |
| Absolute count | IgD- CD38br AC | rs72956154 | A | G | 0.347 | 0.062 | 2.52e-08 | 0.010 | 31.19 |
| Absolute count | IgD- CD38br AC | rs77738700 | G | A | -0.748 | 0.041 | 9.39e-71 | 0.097 | 332.77 |
| Absolute count | Im MDSC AC | rs113013837 | A | G | 0.722 | 0.103 | 3.14e-12 | 0.013 | 48.93 |
| Absolute count | Im MDSC AC | rs139732336 | C | T | 0.545 | 0.091 | 2.10e-09 | 0.010 | 36.04 |
| Absolute count | Im MDSC AC | rs28739016 | T | G | 0.85 | 0.102 | 1.39e-16 | 0.019 | 68.92 |
| Absolute count | Im MDSC AC | rs55712453 | A | G | 0.574 | 0.09 | 2.02e-10 | 0.011 | 40.66 |
| Absolute count | Lymphocyte AC | rs12138291 | G | A | -0.277 | 0.039 | 7.97e-13 | 0.017 | 51.73 |
| Absolute count | Lymphocyte AC | rs142607615 | C | T | -0.607 | 0.096 | 2.47e-10 | 0.014 | 40.32 |
| Absolute count | Lymphocyte AC | rs16848876 | A | G | -0.542 | 0.094 | 9.54e-09 | 0.011 | 33.11 |
| Absolute count | Lymphocyte AC | rs2949661 | C | T | 0.644 | 0.025 | 3.75e-132 | 0.185 | 663.65 |
| Absolute count | Lymphocyte AC | rs72703432 | A | G | -0.481 | 0.086 | 2.53e-08 | 0.011 | 31.2 |
| Absolute count | Memory B cell AC | rs115780495 | T | G | 0.34 | 0.054 | 4.35e-10 | 0.013 | 39.19 |
| Absolute count | Memory B cell AC | rs139529340 | A | G | 0.202 | 0.033 | 1.68e-09 | 0.013 | 36.54 |
| Absolute count | Memory B cell AC | rs139947059 | G | A | 0.468 | 0.084 | 2.89e-08 | 0.011 | 30.93 |
| Absolute count | Memory B cell AC | rs1800973 | C | A | -0.349 | 0.051 | 6.15e-12 | 0.016 | 47.65 |
| Absolute count | Memory B cell AC | rs186878842 | A | C | -0.351 | 0.053 | 3.67e-11 | 0.015 | 44.1 |
| Absolute count | Memory B cell AC | rs188161802 | A | G | -0.507 | 0.079 | 1.50e-10 | 0.014 | 41.29 |
| Absolute count | Memory B cell AC | rs4910742 | G | A | 0.38 | 0.054 | 2.39e-12 | 0.017 | 49.52 |
| Absolute count | NK AC | rs116798506 | T | C | -0.586 | 0.086 | 1.12e-11 | 0.013 | 46.39 |
| Absolute count | NK AC | rs145567900 | C | A | -1.023 | 0.119 | 1.06e-17 | 0.020 | 74.11 |
| Absolute count | NK AC | rs16891187 | C | T | -0.363 | 0.066 | 3.36e-08 | 0.008 | 30.6 |
| Absolute count | NK AC | rs548982606 | C | T | -0.696 | 0.089 | 8.72e-15 | 0.016 | 60.65 |
| Absolute count | NK AC | rs574746346 | G | A | -1.315 | 0.082 | 7.11e-56 | 0.066 | 256.34 |
| Absolute count | NK AC | rs807784 | G | T | 0.367 | 0.052 | 2.16e-12 | 0.013 | 49.66 |
| Absolute count | Naive CD8br AC | rs139847598 | G | T | 0.199 | 0.035 | 1.13e-08 | 0.009 | 32.72 |
| Absolute count | Naive CD8br AC | rs144398648 | C | T | 0.803 | 0.118 | 1.45e-11 | 0.012 | 45.89 |
| Absolute count | Naive CD8br AC | rs1569590 | A | G | 0.226 | 0.037 | 1.10e-09 | 0.010 | 37.33 |
| Absolute count | Naive CD8br AC | rs2070770 | C | T | -0.272 | 0.042 | 8.13e-11 | 0.011 | 42.45 |
| Absolute count | Naive CD8br AC | rs30003 | T | C | -0.272 | 0.029 | 1.71e-20 | 0.023 | 87.07 |
| Absolute count | Naive CD8br AC | rs7772894 | C | T | 0.296 | 0.05 | 2.61e-09 | 0.010 | 35.61 |
| Absolute count | Naive DN (CD4-CD8-) AC | rs10882701 | A | C | -0.407 | 0.025 | 3.19e-56 | 0.071 | 258.92 |
| Absolute count | Naive DN (CD4-CD8-) AC | rs11595506 | C | T | -0.405 | 0.06 | 2.01e-11 | 0.013 | 45.26 |
| Absolute count | Naive DN (CD4-CD8-) AC | rs1410600 | C | T | -0.239 | 0.027 | 4.74e-19 | 0.023 | 80.42 |
| Absolute count | Naive DN (CD4-CD8-) AC | rs144271697 | T | C | 0.931 | 0.08 | 9.26e-31 | 0.038 | 135.54 |
| Absolute count | Naive DN (CD4-CD8-) AC | rs185198079 | C | T | 0.782 | 0.096 | 6.75e-16 | 0.019 | 65.79 |
| Absolute count | Naive DN (CD4-CD8-) AC | rs191910319 | G | A | 0.481 | 0.071 | 1.36e-11 | 0.013 | 46.02 |
| Absolute count | Naive DN (CD4-CD8-) AC | rs1923692 | T | G | -0.432 | 0.056 | 1.13e-14 | 0.017 | 60.14 |
| Absolute count | Naive DN (CD4-CD8-) AC | rs7099844 | T | G | 0.971 | 0.021 | 1.00e-200 | 0.392 | 2198.18 |
| Absolute count | Naive DN (CD4-CD8-) AC | rs77218771 | T | C | 0.938 | 0.13 | 6.39e-13 | 0.015 | 52.1 |
| Absolute count | Naive-mature B cell AC | rs181878688 | G | A | 0.399 | 0.07 | 1.07e-08 | 0.010 | 32.86 |
| Absolute count | Naive-mature B cell AC | rs185858489 | G | A | 0.424 | 0.072 | 5.38e-09 | 0.010 | 34.19 |
| Absolute count | Naive-mature B cell AC | rs6710361 | G | A | 0.192 | 0.032 | 1.45e-09 | 0.011 | 36.79 |
| Absolute count | Naive-mature B cell AC | rs9896155 | A | G | 0.186 | 0.027 | 5.64e-12 | 0.014 | 47.77 |
| Absolute count | PB/PC AC | rs1801274 | A | G | -0.41 | 0.026 | 4.45e-54 | 0.065 | 247.96 |
| Absolute count | PB/PC AC | rs184012084 | G | A | 0.726 | 0.09 | 1.10e-15 | 0.018 | 64.8 |
| Absolute count | PB/PC AC | rs28655219 | T | C | -0.395 | 0.036 | 1.49e-27 | 0.032 | 120.21 |
| Absolute count | Plasmacytoid DC AC | rs112062469 | G | A | -0.4 | 0.058 | 6.90e-12 | 0.013 | 47.35 |
| Absolute count | Plasmacytoid DC AC | rs112266065 | C | T | -0.853 | 0.116 | 2.53e-13 | 0.015 | 53.93 |
| Absolute count | Plasmacytoid DC AC | rs114919524 | A | C | -1.106 | 0.107 | 1.29e-24 | 0.028 | 106.39 |
| Absolute count | Plasmacytoid DC AC | rs117705111 | G | A | -0.673 | 0.066 | 2.04e-24 | 0.028 | 105.45 |
| Absolute count | Plasmacytoid DC AC | rs144365928 | A | G | -0.666 | 0.078 | 1.90e-17 | 0.020 | 72.93 |
| Absolute count | Plasmacytoid DC AC | rs146434619 | T | C | -0.521 | 0.045 | 3.65e-30 | 0.035 | 132.58 |
| Absolute count | Plasmacytoid DC AC | rs149806587 | A | G | -0.477 | 0.058 | 1.73e-16 | 0.018 | 68.5 |
| Absolute count | Plasmacytoid DC AC | rs184205740 | C | T | -1.193 | 0.117 | 3.20e-24 | 0.028 | 104.45 |
| Absolute count | Plasmacytoid DC AC | rs191363479 | C | T | -0.918 | 0.11 | 9.76e-17 | 0.019 | 69.62 |
| Absolute count | Plasmacytoid DC AC | rs191925297 | A | G | -0.382 | 0.068 | 2.65e-08 | 0.008 | 31.07 |
| Absolute count | Plasmacytoid DC AC | rs193039847 | G | A | -1.271 | 0.087 | 8.50e-47 | 0.055 | 212.24 |
| Absolute count | Plasmacytoid DC AC | rs539241708 | C | T | -1.092 | 0.086 | 1.86e-36 | 0.043 | 162.54 |
| Absolute count | Plasmacytoid DC AC | rs556260593 | A | C | -0.92 | 0.106 | 7.29e-18 | 0.020 | 74.83 |
| Absolute count | Plasmacytoid DC AC | rs557577522 | G | A | -0.718 | 0.071 | 1.21e-23 | 0.027 | 101.81 |
| Absolute count | Plasmacytoid DC AC | rs558720004 | A | C | -1.232 | 0.11 | 1.24e-28 | 0.033 | 125.37 |
| Absolute count | Plasmacytoid DC AC | rs5759079 | G | A | -0.405 | 0.036 | 1.32e-28 | 0.033 | 125.13 |
| Absolute count | Plasmacytoid DC AC | rs6002527 | T | C | -1.213 | 0.034 | 1.00e-200 | 0.254 | 1244.87 |
| Absolute count | Resting Treg AC | rs11589644 | C | T | -0.764 | 0.118 | 1.00e-10 | 0.012 | 42.07 |
| Absolute count | Resting Treg AC | rs41306231 | A | G | -0.705 | 0.122 | 8.99e-09 | 0.010 | 33.18 |
| Absolute count | Resting Treg AC | rs60220946 | C | T | -0.515 | 0.087 | 3.04e-09 | 0.010 | 35.32 |
| Absolute count | Resting Treg AC | rs77745909 | A | G | -0.802 | 0.118 | 1.35e-11 | 0.013 | 46.05 |
| Absolute count | Secreting Treg AC | rs1573734 | C | T | -0.182 | 0.029 | 2.20e-10 | 0.014 | 40.52 |
| Absolute count | Secreting Treg AC | rs28680958 | G | A | 0.186 | 0.032 | 8.94e-09 | 0.011 | 33.23 |
| Absolute count | Secreting Treg AC | rs6888699 | T | C | -0.284 | 0.043 | 4.67e-11 | 0.015 | 43.62 |
| Absolute count | T cell AC | rs113013837 | A | G | 0.696 | 0.103 | 1.87e-11 | 0.012 | 45.34 |
| Absolute count | T cell AC | rs28739016 | T | G | 0.833 | 0.102 | 5.49e-16 | 0.018 | 66.22 |
| Absolute count | T cell AC | rs7189927 | T | C | 0.158 | 0.025 | 2.70e-10 | 0.011 | 40.07 |
| Absolute count | T cell AC | rs78760135 | A | G | 0.547 | 0.089 | 9.28e-10 | 0.010 | 37.66 |
| Absolute count | TCRgd AC | rs116499452 | G | A | 0.424 | 0.056 | 6.40e-14 | 0.019 | 56.77 |
| Absolute count | TCRgd AC | rs139642648 | C | T | 0.77 | 0.12 | 1.84e-10 | 0.014 | 40.89 |
| Absolute count | TCRgd AC | rs142500247 | T | C | 0.98 | 0.079 | 9.78e-35 | 0.051 | 155.03 |
| Absolute count | TCRgd AC | rs147667223 | C | T | 0.726 | 0.118 | 9.35e-10 | 0.013 | 37.7 |
| Absolute count | TCRgd AC | rs17607399 | C | T | 0.254 | 0.042 | 1.23e-09 | 0.013 | 37.14 |
| Absolute count | TCRgd AC | rs181956108 | C | T | 0.535 | 0.084 | 2.44e-10 | 0.014 | 40.34 |
| Absolute count | TCRgd AC | rs186119640 | T | C | 0.66 | 0.071 | 2.69e-20 | 0.029 | 86.42 |
| Absolute count | TCRgd AC | rs186616415 | T | C | 0.618 | 0.092 | 2.83e-11 | 0.015 | 44.61 |
| Absolute count | TCRgd AC | rs186694440 | A | G | 0.792 | 0.118 | 2.09e-11 | 0.015 | 45.24 |
| Absolute count | TCRgd AC | rs34558770 | C | T | 0.496 | 0.032 | 5.75e-51 | 0.075 | 234.33 |
| Absolute count | TCRgd AC | rs535516159 | T | C | 0.668 | 0.1 | 3.06e-11 | 0.015 | 44.44 |
| Absolute count | TCRgd AC | rs568833722 | C | T | 0.559 | 0.094 | 3.29e-09 | 0.012 | 35.2 |
| Absolute count | TCRgd AC | rs7565287 | G | A | -0.362 | 0.053 | 1.17e-11 | 0.016 | 46.37 |
| Absolute count | TCRgd AC | rs76156778 | G | A | 0.625 | 0.102 | 1.15e-09 | 0.013 | 37.25 |
| Absolute count | TCRgd AC | rs917116 | T | G | 0.176 | 0.03 | 4.26e-09 | 0.012 | 34.7 |
| Absolute count | TD CD8br AC | rs146866569 | C | T | 0.582 | 0.086 | 1.29e-11 | 0.013 | 46.1 |
| Absolute count | TD CD8br AC | rs71632979 | A | G | -0.701 | 0.027 | 1.40e-134 | 0.155 | 663.43 |
| Absolute count | TD CD8br AC | rs71639910 | G | A | -0.286 | 0.049 | 4.58e-09 | 0.009 | 34.51 |
| Absolute count | TD CD8br AC | rs74341264 | A | G | -0.238 | 0.038 | 4.60e-10 | 0.011 | 39.03 |
| Absolute count | TD DN (CD4-CD8-) AC | rs113013837 | A | G | 0.65 | 0.104 | 4.26e-10 | 0.011 | 39.17 |
| Absolute count | TD DN (CD4-CD8-) AC | rs28739016 | T | G | 0.76 | 0.103 | 1.67e-13 | 0.015 | 54.68 |
| Absolute count | TD DN (CD4-CD8-) AC | rs3934748 | G | T | 0.495 | 0.09 | 3.38e-08 | 0.008 | 30.6 |
| Absolute count | TD DN (CD4-CD8-) AC | rs7189927 | T | C | 0.167 | 0.025 | 2.98e-11 | 0.012 | 44.46 |
| MFI | BAFF-R on CD20- CD38- | rs2181036 | T | C | -0.145 | 0.026 | 3.17e-08 | 0.009 | 30.72 |
| MFI | BAFF-R on CD20- CD38- | rs3087456 | G | A | -0.429 | 0.028 | 5.57e-52 | 0.062 | 237.56 |
| MFI | BAFF-R on CD20- CD38- | rs72779785 | A | G | -0.212 | 0.033 | 1.36e-10 | 0.011 | 41.43 |
| MFI | BAFF-R on CD20- CD38- | rs9378213 | G | T | -0.243 | 0.031 | 5.16e-15 | 0.017 | 61.69 |
| MFI | BAFF-R on IgD+ CD38- | rs56199187 | T | C | 0.589 | 0.028 | 1.96e-92 | 0.115 | 442.11 |
| MFI | BAFF-R on IgD+ CD38- | rs74341264 | A | G | -0.25 | 0.039 | 1.12e-10 | 0.012 | 41.86 |
| MFI | BAFF-R on IgD+ CD38- | rs876036 | T | C | -0.184 | 0.029 | 2.25e-10 | 0.012 | 40.43 |
| MFI | BAFF-R on IgD+ CD38br | rs112133087 | G | T | -0.385 | 0.07 | 3.59e-08 | 0.009 | 30.49 |
| MFI | BAFF-R on IgD+ CD38br | rs532635130 | C | T | -0.4 | 0.069 | 5.77e-09 | 0.010 | 34.07 |
| MFI | BAFF-R on IgD+ CD38br | rs71632989 | T | G | 0.919 | 0.027 | 1.00e-200 | 0.255 | 1161.27 |
| MFI | BAFF-R on IgD+ CD38br | rs71639910 | G | A | 0.425 | 0.052 | 3.96e-16 | 0.019 | 66.89 |
| MFI | BAFF-R on IgD+ CD38br | rs74341264 | A | G | 0.451 | 0.04 | 3.71e-29 | 0.036 | 127.95 |
| MFI | BAFF-R on IgD+ CD38dim | rs111534725 | G | T | 0.772 | 0.064 | 3.06e-33 | 0.042 | 147.35 |
| MFI | BAFF-R on IgD+ CD38dim | rs112505169 | C | T | 0.797 | 0.112 | 1.51e-12 | 0.015 | 50.39 |
| MFI | BAFF-R on IgD+ CD38dim | rs117245428 | G | A | 0.506 | 0.085 | 2.64e-09 | 0.010 | 35.61 |
| MFI | BAFF-R on IgD+ CD38dim | rs11890666 | G | A | -0.157 | 0.028 | 2.88e-08 | 0.009 | 30.93 |
| MFI | BAFF-R on IgD+ CD38dim | rs12712610 | A | G | 0.191 | 0.027 | 4.17e-12 | 0.014 | 48.35 |
| MFI | BAFF-R on IgD+ CD38dim | rs1360005 | T | C | -0.222 | 0.026 | 7.80e-18 | 0.022 | 74.78 |
| MFI | BAFF-R on IgD+ CD38dim | rs17111341 | T | C | 0.653 | 0.051 | 4.41e-37 | 0.046 | 165.68 |
| MFI | BAFF-R on IgD+ CD38dim | rs185198079 | C | T | 0.696 | 0.093 | 8.96e-14 | 0.016 | 56.02 |
| MFI | BAFF-R on IgD+ CD38dim | rs3176881 | A | G | 0.805 | 0.021 | 1.00e-200 | 0.293 | 1407.35 |
| MFI | BAFF-R on IgD+ CD38dim | rs77520132 | G | A | 0.583 | 0.059 | 1.03e-22 | 0.028 | 97.55 |
| MFI | BAFF-R on IgD+ CD38dim | rs7906684 | G | A | 1.072 | 0.078 | 1.24e-41 | 0.052 | 187.48 |
| MFI | BAFF-R on IgD- CD38br | rs71632979 | A | G | 0.809 | 0.028 | 9.71e-166 | 0.199 | 842.9 |
| MFI | BAFF-R on IgD- CD38br | rs71639910 | G | A | 0.367 | 0.052 | 2.25e-12 | 0.014 | 49.6 |
| MFI | BAFF-R on IgD- CD38br | rs74341264 | A | G | 0.355 | 0.04 | 1.25e-18 | 0.023 | 78.48 |
| MFI | CCR2 on CD14- CD16+ monocyte | rs12219630 | C | T | -0.185 | 0.025 | 1.18e-13 | 0.015 | 55.45 |
| MFI | CCR2 on CD14- CD16+ monocyte | rs1887027 | C | T | -0.42 | 0.027 | 1.16e-51 | 0.061 | 235.86 |
| MFI | CCR2 on CD14- CD16+ monocyte | rs3793662 | C | T | -0.163 | 0.029 | 2.05e-08 | 0.009 | 31.55 |
| MFI | CCR2 on CD62L+ myeloid DC | rs113013837 | A | G | 0.876 | 0.104 | 4.82e-17 | 0.019 | 71.03 |
| MFI | CCR2 on CD62L+ myeloid DC | rs139732336 | C | T | 0.548 | 0.092 | 2.48e-09 | 0.010 | 35.71 |
| MFI | CCR2 on CD62L+ myeloid DC | rs150816467 | T | C | 0.68 | 0.093 | 2.50e-13 | 0.015 | 53.95 |
| MFI | CCR2 on CD62L+ myeloid DC | rs28739016 | T | G | 0.989 | 0.103 | 1.42e-21 | 0.025 | 92.05 |
| MFI | CCR2 on CD62L+ myeloid DC | rs575687159 | C | T | 0.53 | 0.088 | 1.52e-09 | 0.010 | 36.68 |
| MFI | CCR2 on CD62L+ myeloid DC | rs62037424 | C | T | -0.605 | 0.106 | 1.05e-08 | 0.009 | 32.87 |
| MFI | CCR2 on CD62L+ plasmacytoid DC | rs111902980 | G | A | 0.513 | 0.092 | 2.65e-08 | 0.009 | 31.08 |
| MFI | CCR2 on CD62L+ plasmacytoid DC | rs116877116 | G | A | 0.234 | 0.042 | 3.32e-08 | 0.009 | 30.63 |
| MFI | CCR2 on CD62L+ plasmacytoid DC | rs6729180 | C | T | -0.203 | 0.026 | 1.09e-14 | 0.017 | 60.23 |
| MFI | CCR2 on granulocyte | rs113075410 | A | G | 0.311 | 0.055 | 1.74e-08 | 0.009 | 31.89 |
| MFI | CCR2 on granulocyte | rs139249541 | A | G | 0.566 | 0.071 | 2.99e-15 | 0.017 | 62.77 |
| MFI | CCR2 on granulocyte | rs144837535 | T | C | 0.557 | 0.07 | 3.16e-15 | 0.017 | 62.67 |
| MFI | CCR2 on granulocyte | rs150386792 | G | A | 0.53 | 0.074 | 8.76e-13 | 0.014 | 51.44 |
| MFI | CCR2 on granulocyte | rs181878688 | G | A | 0.663 | 0.067 | 1.23e-22 | 0.026 | 97.09 |
| MFI | CCR2 on granulocyte | rs182904923 | T | G | 0.541 | 0.074 | 2.56e-13 | 0.014 | 53.89 |
| MFI | CCR2 on granulocyte | rs186978368 | C | T | 0.496 | 0.072 | 7.32e-12 | 0.013 | 47.22 |
| MFI | CCR2 on granulocyte | rs62432266 | G | T | 0.36 | 0.052 | 7.36e-12 | 0.013 | 47.2 |
| MFI | CCR2 on monocyte | rs111534725 | G | T | 0.565 | 0.066 | 1.77e-17 | 0.021 | 73.11 |
| MFI | CCR2 on monocyte | rs11188757 | A | C | -0.249 | 0.031 | 3.06e-15 | 0.018 | 62.81 |
| MFI | CCR2 on monocyte | rs11596351 | G | A | 0.607 | 0.111 | 4.43e-08 | 0.009 | 30.09 |
| MFI | CCR2 on monocyte | rs1360005 | T | C | -0.25 | 0.026 | 3.64e-21 | 0.026 | 90.29 |
| MFI | CCR2 on monocyte | rs144271697 | T | C | 0.771 | 0.08 | 6.68e-22 | 0.027 | 93.76 |
| MFI | CCR2 on monocyte | rs17111341 | T | C | 0.544 | 0.053 | 1.04e-24 | 0.030 | 106.94 |
| MFI | CCR2 on monocyte | rs181392469 | G | A | -0.705 | 0.126 | 2.64e-08 | 0.009 | 31.06 |
| MFI | CCR2 on monocyte | rs4918969 | G | A | 0.876 | 0.022 | 1.00e-200 | 0.327 | 1656.03 |
| MFI | CCR2 on myeloid DC | rs12712610 | A | G | -0.163 | 0.026 | 7.83e-10 | 0.011 | 38.02 |
| MFI | CCR2 on myeloid DC | rs150386792 | G | A | -0.402 | 0.072 | 2.90e-08 | 0.009 | 30.91 |
| MFI | CCR2 on myeloid DC | rs181878688 | G | A | -0.405 | 0.066 | 9.09e-10 | 0.011 | 37.7 |
| MFI | CCR7 on naive CD8br | rs142186496 | A | G | 0.458 | 0.077 | 3.34e-09 | 0.022 | 35.34 |
| MFI | CCR7 on naive CD8br | rs9270588 | C | T | -0.43 | 0.04 | 7.98e-26 | 0.068 | 114.26 |
| MFI | CCR7 on naive CD8br | rs9469245 | C | T | -0.22 | 0.04 | 3.45e-08 | 0.019 | 30.7 |
| MFI | CD11b on CD33dim HLA DR- | rs112505169 | C | T | 0.853 | 0.105 | 7.03e-16 | 0.019 | 65.71 |
| MFI | CD11b on CD33dim HLA DR- | rs11589644 | C | T | 0.644 | 0.109 | 3.69e-09 | 0.010 | 34.94 |
| MFI | CD11b on CD33dim HLA DR- | rs142500247 | T | C | 0.636 | 0.073 | 6.43e-18 | 0.021 | 75.17 |
| MFI | CD11b on CD33dim HLA DR- | rs408686 | C | A | -0.266 | 0.03 | 4.22e-18 | 0.022 | 76.03 |
| MFI | CD11b on CD33dim HLA DR- | rs6751481 | T | C | 0.276 | 0.023 | 4.78e-32 | 0.040 | 141.65 |
| MFI | CD11b on CD66b++ myeloid cell | rs11615628 | G | A | -0.237 | 0.031 | 7.48e-14 | 0.019 | 56.46 |
| MFI | CD11b on CD66b++ myeloid cell | rs12298133 | C | T | 0.198 | 0.036 | 4.63e-08 | 0.010 | 30.02 |
| MFI | CD11b on CD66b++ myeloid cell | rs9264532 | C | T | 0.169 | 0.03 | 2.29e-08 | 0.011 | 31.37 |
| MFI | CD11b on Mo MDSC | rs1801274 | A | G | 0.551 | 0.034 | 2.77e-54 | 0.122 | 256.82 |
| MFI | CD11b on Mo MDSC | rs3820097 | G | A | -0.431 | 0.074 | 7.11e-09 | 0.018 | 33.79 |
| MFI | CD11b on Mo MDSC | rs3826007 | C | T | 0.361 | 0.04 | 7.44e-19 | 0.041 | 80.28 |
| MFI | CD11b on basophil | rs112266065 | C | T | -0.977 | 0.115 | 3.10e-17 | 0.019 | 71.95 |
| MFI | CD11b on basophil | rs114919524 | A | C | -1.126 | 0.106 | 9.33e-26 | 0.030 | 111.72 |
| MFI | CD11b on basophil | rs117119882 | C | A | -0.877 | 0.115 | 3.71e-14 | 0.016 | 57.79 |
| MFI | CD11b on basophil | rs141059303 | G | A | -0.646 | 0.117 | 3.34e-08 | 0.008 | 30.61 |
| MFI | CD11b on basophil | rs144365928 | A | G | -0.694 | 0.078 | 7.21e-19 | 0.021 | 79.54 |
| MFI | CD11b on basophil | rs148516965 | C | T | -0.565 | 0.056 | 1.22e-23 | 0.027 | 101.81 |
| MFI | CD11b on basophil | rs149167072 | A | G | 0.307 | 0.053 | 9.39e-09 | 0.009 | 33.09 |
| MFI | CD11b on basophil | rs149806587 | A | G | -0.535 | 0.057 | 1.61e-20 | 0.023 | 87.2 |
| MFI | CD11b on basophil | rs184205740 | C | T | -1.211 | 0.116 | 3.27e-25 | 0.029 | 109.11 |
| MFI | CD11b on basophil | rs185098316 | G | A | -0.289 | 0.052 | 3.61e-08 | 0.008 | 30.47 |
| MFI | CD11b on basophil | rs185430947 | A | G | -1.023 | 0.109 | 1.00e-20 | 0.024 | 88.2 |
| MFI | CD11b on basophil | rs188216171 | G | A | -0.861 | 0.11 | 4.66e-15 | 0.017 | 61.84 |
| MFI | CD11b on basophil | rs189799494 | G | T | -0.638 | 0.051 | 9.06e-36 | 0.042 | 159.16 |
| MFI | CD11b on basophil | rs191363479 | C | T | -1.024 | 0.11 | 2.18e-20 | 0.023 | 86.61 |
| MFI | CD11b on basophil | rs193039847 | G | A | -1.359 | 0.087 | 6.83e-54 | 0.063 | 246.64 |
| MFI | CD11b on basophil | rs532866318 | T | G | -1.378 | 0.119 | 2.48e-30 | 0.035 | 133.35 |
| MFI | CD11b on basophil | rs535059510 | C | T | -0.922 | 0.118 | 5.83e-15 | 0.017 | 61.39 |
| MFI | CD11b on basophil | rs539241708 | C | T | -1.201 | 0.085 | 2.80e-44 | 0.052 | 200.14 |
| MFI | CD11b on basophil | rs556260593 | A | C | -1.053 | 0.106 | 5.67e-23 | 0.026 | 98.63 |
| MFI | CD11b on basophil | rs557577522 | G | A | -0.77 | 0.071 | 3.12e-27 | 0.032 | 118.66 |
| MFI | CD11b on basophil | rs568374507 | C | T | -0.531 | 0.059 | 2.63e-19 | 0.022 | 81.56 |
| MFI | CD11b on basophil | rs5758649 | C | T | -0.553 | 0.045 | 3.93e-34 | 0.040 | 151.42 |
| MFI | CD11b on basophil | rs73165129 | C | T | -1.496 | 0.034 | 1.00e-200 | 0.350 | 1960.22 |
| MFI | CD11b on basophil | rs73888140 | G | A | -0.535 | 0.06 | 6.34e-19 | 0.021 | 79.77 |
| MFI | CD11b on basophil | rs78806227 | C | T | -0.941 | 0.12 | 5.92e-15 | 0.017 | 61.47 |
| MFI | CD11b on basophil | rs79085286 | T | C | -0.342 | 0.054 | 1.93e-10 | 0.011 | 40.75 |
| MFI | CD11c on granulocyte | rs112062469 | G | A | -0.459 | 0.058 | 2.92e-15 | 0.017 | 62.83 |
| MFI | CD11c on granulocyte | rs112266065 | C | T | -0.938 | 0.116 | 6.56e-16 | 0.018 | 65.86 |
| MFI | CD11c on granulocyte | rs114919524 | A | C | -1.131 | 0.107 | 8.48e-26 | 0.030 | 111.87 |
| MFI | CD11c on granulocyte | rs117705111 | G | A | -0.741 | 0.065 | 1.97e-29 | 0.034 | 129.03 |
| MFI | CD11c on granulocyte | rs141059303 | G | A | -0.656 | 0.117 | 2.33e-08 | 0.008 | 31.33 |
| MFI | CD11c on granulocyte | rs144712270 | G | A | -0.651 | 0.11 | 4.04e-09 | 0.009 | 34.78 |
| MFI | CD11c on granulocyte | rs146434619 | T | C | -0.583 | 0.045 | 2.02e-37 | 0.044 | 167.06 |
| MFI | CD11c on granulocyte | rs149806587 | A | G | -0.534 | 0.057 | 2.50e-20 | 0.023 | 86.32 |
| MFI | CD11c on granulocyte | rs1801274 | A | G | -0.172 | 0.027 | 1.86e-10 | 0.011 | 40.81 |
| MFI | CD11c on granulocyte | rs181447960 | G | A | 0.282 | 0.051 | 3.89e-08 | 0.008 | 30.31 |
| MFI | CD11c on granulocyte | rs184205740 | C | T | -1.208 | 0.116 | 6.47e-25 | 0.029 | 107.64 |
| MFI | CD11c on granulocyte | rs191363479 | C | T | -1.044 | 0.11 | 2.56e-21 | 0.024 | 90.85 |
| MFI | CD11c on granulocyte | rs193039847 | G | A | -1.351 | 0.087 | 6.33e-53 | 0.062 | 242.07 |
| MFI | CD11c on granulocyte | rs539241708 | C | T | -1.18 | 0.085 | 1.53e-42 | 0.050 | 191.67 |
| MFI | CD11c on granulocyte | rs556260593 | A | C | -1.04 | 0.106 | 1.52e-22 | 0.026 | 96.76 |
| MFI | CD11c on granulocyte | rs557577522 | G | A | -0.763 | 0.071 | 1.36e-26 | 0.031 | 115.66 |
| MFI | CD11c on granulocyte | rs558720004 | A | C | -1.267 | 0.11 | 2.31e-30 | 0.035 | 133.57 |
| MFI | CD11c on granulocyte | rs5759079 | G | A | -0.444 | 0.036 | 3.75e-34 | 0.040 | 151.52 |
| MFI | CD11c on granulocyte | rs6002524 | T | G | -1.38 | 0.035 | 1.00e-200 | 0.294 | 1523.15 |
| MFI | CD11c on granulocyte | rs77552099 | T | C | -0.307 | 0.049 | 4.17e-10 | 0.011 | 39.23 |
| MFI | CD11c on monocyte | rs10512469 | C | T | 0.249 | 0.031 | 1.98e-15 | 0.017 | 63.58 |
| MFI | CD11c on monocyte | rs183053322 | C | T | -0.408 | 0.064 | 2.51e-10 | 0.011 | 40.23 |
| MFI | CD11c on monocyte | rs9916257 | G | T | -0.232 | 0.023 | 1.51e-23 | 0.027 | 101.42 |
| MFI | CD11c on myeloid DC | rs11657118 | T | C | -0.143 | 0.024 | 3.70e-09 | 0.009 | 34.92 |
| MFI | CD11c on myeloid DC | rs12874404 | A | G | 0.234 | 0.026 | 1.09e-18 | 0.021 | 78.66 |
| MFI | CD11c on myeloid DC | rs139370814 | C | T | 0.539 | 0.087 | 7.29e-10 | 0.010 | 38.13 |
| MFI | CD123 on CD62L+ plasmacytoid DC | rs139370814 | C | T | -0.493 | 0.088 | 2.30e-08 | 0.009 | 31.34 |
| MFI | CD123 on CD62L+ plasmacytoid DC | rs72635970 | T | G | -0.34 | 0.061 | 3.01e-08 | 0.008 | 30.82 |
| MFI | CD123 on CD62L+ plasmacytoid DC | rs74053426 | G | A | 0.534 | 0.095 | 2.20e-08 | 0.009 | 31.43 |
| MFI | CD127 on CD28+ CD4+ | rs142456232 | C | T | -0.334 | 0.059 | 1.81e-08 | 0.011 | 31.84 |
| MFI | CD127 on CD28+ CD4+ | rs149728699 | G | A | -0.469 | 0.077 | 1.24e-09 | 0.013 | 37.11 |
| MFI | CD127 on CD28+ CD4+ | rs17552904 | G | T | -0.185 | 0.032 | 1.19e-08 | 0.011 | 32.68 |
| MFI | CD127 on CD28+ CD4+ | rs4566648 | T | G | -0.219 | 0.029 | 4.41e-14 | 0.020 | 57.55 |
| MFI | CD127 on CD28+ CD4+ | rs722926 | C | T | 0.164 | 0.028 | 3.49e-09 | 0.012 | 35.06 |
| MFI | CD127 on CD28+ CD4+ | rs76053862 | T | C | -0.388 | 0.059 | 5.61e-11 | 0.015 | 43.26 |
| MFI | CD127 on CD28+ CD4+ | rs78736654 | T | G | -0.404 | 0.074 | 4.41e-08 | 0.010 | 30.11 |
| MFI | CD127 on CD28+ CD45RA+ CD8br | rs139249541 | A | G | -0.44 | 0.08 | 3.78e-08 | 0.010 | 30.4 |
| MFI | CD127 on CD28+ CD45RA+ CD8br | rs144837535 | T | C | -0.633 | 0.078 | 1.03e-15 | 0.022 | 65.06 |
| MFI | CD127 on CD28+ CD45RA+ CD8br | rs181878688 | G | A | -0.778 | 0.075 | 8.33e-25 | 0.036 | 107.62 |
| MFI | CD127 on CD28+ CD45RA+ CD8br | rs182904923 | T | G | -0.517 | 0.082 | 3.86e-10 | 0.013 | 39.42 |
| MFI | CD127 on CD28+ CD45RA+ CD8br | rs186978368 | C | T | -0.651 | 0.079 | 2.60e-16 | 0.023 | 67.82 |
| MFI | CD127 on CD28+ CD45RA+ CD8br | rs533013936 | A | G | -0.602 | 0.079 | 2.51e-14 | 0.020 | 58.65 |
| MFI | CD127 on CD28+ CD45RA+ CD8br | rs62184019 | C | T | -0.435 | 0.034 | 2.31e-36 | 0.053 | 162.87 |
| MFI | CD127 on CD28+ CD45RA+ CD8br | rs62432266 | G | T | -0.374 | 0.058 | 1.57e-10 | 0.014 | 41.2 |
| MFI | CD127 on CD28- CD8br | rs113063605 | A | G | 0.218 | 0.04 | 3.83e-08 | 0.008 | 30.36 |
| MFI | CD127 on CD28- CD8br | rs137933840 | G | A | 0.421 | 0.071 | 3.01e-09 | 0.010 | 35.32 |
| MFI | CD127 on CD28- CD8br | rs1801274 | A | G | 0.509 | 0.026 | 7.00e-84 | 0.099 | 396.81 |
| MFI | CD127 on CD28- CD8br | rs186463618 | C | A | 0.493 | 0.066 | 9.60e-14 | 0.015 | 55.85 |
| MFI | CD127 on CD28- CD8br | rs34658867 | A | C | 0.366 | 0.061 | 1.58e-09 | 0.010 | 36.6 |
| MFI | CD127 on CD28- CD8br | rs745307 | G | A | 0.715 | 0.033 | 7.30e-96 | 0.112 | 458.02 |
| MFI | CD127 on T cell | rs1800973 | C | A | -0.256 | 0.046 | 2.51e-08 | 0.009 | 31.18 |
| MFI | CD127 on T cell | rs62395272 | C | T | -0.255 | 0.033 | 1.61e-14 | 0.016 | 59.42 |
| MFI | CD127 on T cell | rs9270588 | C | T | -0.539 | 0.028 | 3.21e-78 | 0.092 | 367.98 |
| MFI | CD127 on T cell | rs9469245 | C | T | -0.262 | 0.028 | 8.23e-21 | 0.024 | 88.57 |
| MFI | CD127 on granulocyte | rs2236073 | G | A | 0.179 | 0.025 | 6.38e-13 | 0.014 | 52.09 |
| MFI | CD127 on granulocyte | rs7246669 | A | G | 0.151 | 0.028 | 4.04e-08 | 0.008 | 30.25 |
| MFI | CD127 on granulocyte | rs72948545 | A | G | -0.858 | 0.051 | 2.16e-61 | 0.072 | 283.65 |
| MFI | CD14 on CD14+ CD16+ monocyte | rs111993863 | G | A | -0.858 | 0.067 | 3.90e-37 | 0.043 | 165.72 |
| MFI | CD14 on CD14+ CD16+ monocyte | rs112266065 | C | T | -0.92 | 0.116 | 2.59e-15 | 0.017 | 63.03 |
| MFI | CD14 on CD14+ CD16+ monocyte | rs112644855 | G | A | 0.318 | 0.056 | 2.01e-08 | 0.009 | 31.62 |
| MFI | CD14 on CD14+ CD16+ monocyte | rs117458132 | C | T | -0.327 | 0.05 | 5.10e-11 | 0.012 | 43.36 |
| MFI | CD14 on CD14+ CD16+ monocyte | rs118166233 | C | T | -0.652 | 0.104 | 4.56e-10 | 0.011 | 39.08 |
| MFI | CD14 on CD14+ CD16+ monocyte | rs143591969 | G | A | -1.276 | 0.039 | 1.00e-200 | 0.227 | 1071.53 |
| MFI | CD14 on CD14+ CD16+ monocyte | rs146434619 | T | C | -0.542 | 0.045 | 1.36e-32 | 0.038 | 144.07 |
| MFI | CD14 on CD14+ CD16+ monocyte | rs149806587 | A | G | -0.483 | 0.058 | 7.25e-17 | 0.019 | 70.23 |
| MFI | CD14 on CD14+ CD16+ monocyte | rs150288192 | C | T | -1.217 | 0.117 | 5.50e-25 | 0.029 | 108.14 |
| MFI | CD14 on CD14+ CD16+ monocyte | rs191363479 | C | T | -0.924 | 0.11 | 5.89e-17 | 0.019 | 70.65 |
| MFI | CD14 on CD14+ CD16+ monocyte | rs4657041 | T | C | -0.152 | 0.027 | 1.93e-08 | 0.009 | 31.71 |
| MFI | CD14 on CD14+ CD16+ monocyte | rs539130256 | T | C | -0.596 | 0.106 | 1.72e-08 | 0.009 | 31.92 |
| MFI | CD14 on CD14+ CD16+ monocyte | rs539241708 | C | T | -1.167 | 0.085 | 1.35e-41 | 0.049 | 187.03 |
| MFI | CD14 on CD14+ CD16+ monocyte | rs556260593 | A | C | -0.933 | 0.106 | 2.27e-18 | 0.021 | 77.28 |
| MFI | CD14 on CD14+ CD16+ monocyte | rs558720004 | A | C | -1.259 | 0.11 | 6.53e-30 | 0.035 | 131.4 |
| MFI | CD14 on CD14+ CD16+ monocyte | rs561787258 | G | A | -1.183 | 0.084 | 1.58e-43 | 0.051 | 196.54 |
| MFI | CD14 on CD14+ CD16+ monocyte | rs570148043 | G | A | -0.924 | 0.109 | 3.08e-17 | 0.019 | 71.95 |
| MFI | CD14 on CD14+ CD16+ monocyte | rs5759079 | G | A | -0.428 | 0.036 | 6.31e-32 | 0.037 | 140.91 |
| MFI | CD14 on CD14+ CD16+ monocyte | rs576126454 | T | G | -1.33 | 0.086 | 4.20e-52 | 0.061 | 238.1 |
| MFI | CD14 on CD14+ CD16+ monocyte | rs73888140 | G | A | -0.485 | 0.06 | 9.88e-16 | 0.017 | 65.01 |
| MFI | CD14 on CD14+ CD16+ monocyte | rs77552099 | T | C | -0.276 | 0.049 | 1.91e-08 | 0.009 | 31.7 |
| MFI | CD16 on CD14- CD16+ monocyte | rs139249541 | A | G | 0.457 | 0.07 | 9.72e-11 | 0.011 | 42.1 |
| MFI | CD16 on CD14- CD16+ monocyte | rs144837535 | T | C | 0.454 | 0.069 | 6.19e-11 | 0.012 | 42.99 |
| MFI | CD16 on CD14- CD16+ monocyte | rs150386792 | G | A | 0.423 | 0.073 | 6.64e-09 | 0.009 | 33.78 |
| MFI | CD16 on CD14- CD16+ monocyte | rs181878688 | G | A | 0.515 | 0.066 | 1.12e-14 | 0.016 | 60.14 |
| MFI | CD16 on CD14- CD16+ monocyte | rs186978368 | C | T | 0.439 | 0.071 | 6.73e-10 | 0.010 | 38.29 |
| MFI | CD16 on CD14- CD16+ monocyte | rs7569868 | C | T | 0.246 | 0.031 | 1.70e-15 | 0.017 | 63.92 |
| MFI | CD16-CD56 on NKT | rs10882674 | T | C | 0.811 | 0.021 | 1.00e-200 | 0.305 | 1509.52 |
| MFI | CD16-CD56 on NKT | rs10882701 | A | C | -0.368 | 0.024 | 6.66e-50 | 0.062 | 227.83 |
| MFI | CD16-CD56 on NKT | rs118087712 | G | A | 0.256 | 0.039 | 7.77e-11 | 0.012 | 42.56 |
| MFI | CD16-CD56 on NKT | rs141018090 | G | T | 0.579 | 0.054 | 1.79e-26 | 0.032 | 115.19 |
| MFI | CD16-CD56 on NKT | rs184728966 | C | T | 0.87 | 0.093 | 1.24e-20 | 0.025 | 87.81 |
| MFI | CD16-CD56 on NKT | rs191819784 | G | A | 0.933 | 0.073 | 3.72e-36 | 0.045 | 161.23 |
| MFI | CD16-CD56 on NKT | rs61868672 | G | A | -0.354 | 0.05 | 1.24e-12 | 0.015 | 50.77 |
| MFI | CD16-CD56 on NKT | rs7906684 | G | A | 1.195 | 0.077 | 1.13e-52 | 0.066 | 241.4 |
| MFI | CD19 on B cell | rs12196019 | C | A | 0.308 | 0.046 | 1.82e-11 | 0.013 | 45.41 |
| MFI | CD19 on B cell | rs12478924 | T | C | 0.204 | 0.028 | 6.27e-13 | 0.015 | 52.1 |
| MFI | CD19 on B cell | rs2105899 | T | C | -0.279 | 0.033 | 3.81e-17 | 0.020 | 71.6 |
| MFI | CD19 on B cell | rs9272014 | G | A | -0.168 | 0.029 | 6.75e-09 | 0.010 | 33.76 |
| MFI | CD19 on CD20- | rs11615628 | G | A | -0.248 | 0.031 | 3.36e-15 | 0.021 | 62.65 |
| MFI | CD19 on CD20- | rs12298133 | C | T | 0.219 | 0.036 | 1.13e-09 | 0.013 | 37.29 |
| MFI | CD19 on CD20- | rs9264532 | C | T | 0.188 | 0.03 | 3.87e-10 | 0.013 | 39.4 |
| MFI | CD19 on CD20- | rs9266064 | C | T | -0.175 | 0.03 | 3.97e-09 | 0.012 | 34.85 |
| MFI | CD19 on CD20- CD38- | rs36102158 | G | A | 0.274 | 0.049 | 1.89e-08 | 0.019 | 31.89 |
| MFI | CD19 on CD20- CD38- | rs4406271 | G | A | -0.259 | 0.036 | 1.01e-12 | 0.031 | 51.58 |
| MFI | CD19 on CD20- CD38- | rs6688357 | C | T | 0.635 | 0.058 | 6.76e-27 | 0.068 | 119.38 |
| MFI | CD19 on CD24+ CD27+ | rs114672530 | G | A | -0.678 | 0.105 | 1.29e-10 | 0.014 | 41.63 |
| MFI | CD19 on CD24+ CD27+ | rs12138291 | G | A | -0.263 | 0.039 | 1.14e-11 | 0.016 | 46.41 |
| MFI | CD19 on CD24+ CD27+ | rs2949661 | C | T | 0.615 | 0.025 | 4.33e-119 | 0.168 | 590.76 |
| MFI | CD19 on CD24+ CD27+ | rs4657649 | T | C | 0.353 | 0.062 | 1.33e-08 | 0.011 | 32.45 |
| MFI | CD19 on IgD+ CD24+ | rs10205992 | C | T | -0.296 | 0.027 | 3.00e-28 | 0.041 | 123.98 |
| MFI | CD19 on IgD+ CD24+ | rs1264570 | C | T | 0.198 | 0.033 | 1.42e-09 | 0.012 | 36.88 |
| MFI | CD19 on IgD+ CD24+ | rs12660712 | C | A | -0.28 | 0.049 | 1.15e-08 | 0.011 | 32.73 |
| MFI | CD19 on IgD+ CD24+ | rs130065 | G | A | -0.313 | 0.04 | 1.42e-14 | 0.020 | 59.78 |
| MFI | CD19 on IgD+ CD24+ | rs1356625 | T | C | 0.192 | 0.027 | 1.37e-12 | 0.017 | 50.68 |
| MFI | CD19 on IgD+ CD24+ | rs4832277 | G | A | 0.159 | 0.028 | 1.08e-08 | 0.011 | 32.87 |
| MFI | CD19 on IgD+ CD24+ | rs6903896 | G | A | -0.341 | 0.046 | 1.49e-13 | 0.019 | 55.07 |
| MFI | CD19 on IgD+ CD24+ | rs938487 | A | G | -0.517 | 0.03 | 2.91e-65 | 0.095 | 305.86 |
| MFI | CD19 on IgD+ CD38- | rs12138291 | G | A | -0.271 | 0.038 | 1.93e-12 | 0.017 | 49.94 |
| MFI | CD19 on IgD+ CD38- | rs2995089 | G | A | 0.602 | 0.025 | 1.10e-115 | 0.164 | 572.29 |
| MFI | CD19 on IgD+ CD38- | rs4657649 | T | C | 0.447 | 0.061 | 3.64e-13 | 0.018 | 53.28 |
| MFI | CD19 on IgD+ CD38- | rs7519927 | G | A | -0.19 | 0.028 | 7.71e-12 | 0.016 | 47.17 |
| MFI | CD19 on IgD+ CD38- unsw mem | rs16848876 | A | G | -0.52 | 0.094 | 3.10e-08 | 0.010 | 30.79 |
| MFI | CD19 on IgD+ CD38- unsw mem | rs2949661 | C | T | 0.548 | 0.026 | 3.54e-94 | 0.135 | 455.99 |
| MFI | CD19 on IgD+ CD38- unsw mem | rs61814872 | C | T | 0.278 | 0.044 | 5.01e-10 | 0.013 | 38.92 |
| MFI | CD19 on IgD+ CD38- unsw mem | rs76540213 | C | T | 0.526 | 0.096 | 4.79e-08 | 0.010 | 29.94 |
| MFI | CD19 on IgD+ CD38br | rs3793662 | C | T | -0.175 | 0.029 | 2.38e-09 | 0.010 | 35.78 |
| MFI | CD19 on IgD+ CD38br | rs41295093 | G | A | 0.424 | 0.04 | 1.11e-25 | 0.030 | 111.39 |
| MFI | CD19 on IgD+ CD38br | rs7072398 | G | A | -0.156 | 0.026 | 1.52e-09 | 0.010 | 36.68 |
| MFI | CD19 on IgD- CD24- | rs12675296 | G | A | -0.682 | 0.085 | 1.76e-15 | 0.022 | 64 |
| MFI | CD19 on IgD- CD24- | rs1886654 | T | C | -0.208 | 0.038 | 3.36e-08 | 0.011 | 30.64 |
| MFI | CD19 on IgD- CD24- | rs2981398 | A | G | -0.223 | 0.029 | 1.77e-14 | 0.020 | 59.35 |
| MFI | CD19 on IgD- CD27- | rs12212931 | T | G | 0.223 | 0.032 | 2.56e-12 | 0.013 | 49.29 |
| MFI | CD19 on IgD- CD27- | rs144398648 | C | T | 0.742 | 0.119 | 5.09e-10 | 0.011 | 38.85 |
| MFI | CD19 on IgD- CD27- | rs1941018 | A | G | -0.196 | 0.025 | 3.16e-15 | 0.017 | 62.7 |
| MFI | CD19 on IgD- CD27- | rs30003 | T | C | -0.167 | 0.029 | 1.67e-08 | 0.009 | 31.96 |
| MFI | CD19 on IgD- CD27- | rs388354 | A | C | -0.134 | 0.024 | 3.93e-08 | 0.008 | 30.28 |
| MFI | CD19 on IgD- CD27- | rs62405562 | A | G | 0.185 | 0.028 | 9.18e-11 | 0.011 | 42.2 |
| MFI | CD19 on IgD- CD27- | rs72836542 | C | T | 0.157 | 0.027 | 5.42e-09 | 0.009 | 34.18 |
| MFI | CD19 on IgD- CD27- | rs9270560 | T | C | -0.171 | 0.029 | 6.52e-09 | 0.009 | 33.82 |
| MFI | CD19 on IgD- CD38- | rs2236947 | C | A | 0.181 | 0.028 | 2.24e-10 | 0.013 | 40.49 |
| MFI | CD19 on IgD- CD38- | rs2978898 | T | C | -0.156 | 0.028 | 1.80e-08 | 0.010 | 31.87 |
| MFI | CD19 on IgD- CD38- | rs445 | C | T | 0.437 | 0.032 | 3.84e-40 | 0.055 | 180.88 |
| MFI | CD19 on IgD- CD38- | rs7825750 | T | C | 0.234 | 0.031 | 6.74e-14 | 0.018 | 56.63 |
| MFI | CD19 on IgD- CD38dim | rs12459503 | T | C | 0.27 | 0.042 | 1.74e-10 | 0.027 | 41.29 |
| MFI | CD19 on IgD- CD38dim | rs2459145 | T | C | 1.3 | 0.22 | 4.23e-09 | 0.023 | 34.9 |
| MFI | CD19 on IgD- CD38dim | rs3865444 | C | A | -0.95 | 0.039 | 2.39e-108 | 0.284 | 580.21 |
| MFI | CD19 on IgD- CD38dim | rs558625031 | G | A | -0.606 | 0.087 | 4.02e-12 | 0.032 | 48.87 |
| MFI | CD19 on IgD- CD38dim | rs7351079 | A | G | -0.31 | 0.043 | 1.04e-12 | 0.034 | 51.62 |
| MFI | CD19 on IgD- CD38dim | rs78949787 | C | T | 0.268 | 0.038 | 2.62e-12 | 0.033 | 49.73 |
| MFI | CD19 on sw mem | rs1081003 | G | A | -0.52 | 0.06 | 8.86e-18 | 0.039 | 75.21 |
| MFI | CD19 on sw mem | rs117705111 | G | A | -0.713 | 0.092 | 1.25e-14 | 0.032 | 60.39 |
| MFI | CD19 on sw mem | rs144365928 | A | G | -0.743 | 0.11 | 1.89e-11 | 0.024 | 45.62 |
| MFI | CD19 on sw mem | rs149806587 | A | G | -0.505 | 0.08 | 3.18e-10 | 0.021 | 39.97 |
| MFI | CD19 on sw mem | rs185409600 | A | G | -0.466 | 0.08 | 7.30e-09 | 0.018 | 33.74 |
| MFI | CD19 on sw mem | rs1894588 | C | T | -0.607 | 0.07 | 9.72e-18 | 0.039 | 75 |
| MFI | CD19 on sw mem | rs191363479 | C | T | -1.084 | 0.157 | 6.57e-12 | 0.025 | 47.74 |
| MFI | CD19 on sw mem | rs193039847 | G | A | -1.349 | 0.117 | 9.16e-30 | 0.067 | 132.79 |
| MFI | CD19 on sw mem | rs2018340 | G | A | -0.472 | 0.05 | 7.45e-21 | 0.047 | 89.81 |
| MFI | CD19 on sw mem | rs539241708 | C | T | -1.149 | 0.114 | 3.14e-23 | 0.052 | 101.12 |
| MFI | CD19 on sw mem | rs556260593 | A | C | -1.203 | 0.145 | 2.13e-16 | 0.036 | 68.66 |
| MFI | CD19 on sw mem | rs557577522 | G | A | -0.645 | 0.095 | 1.32e-11 | 0.025 | 46.33 |
| MFI | CD19 on sw mem | rs73165129 | C | T | -1.524 | 0.047 | 1.14e-183 | 0.366 | 1057.46 |
| MFI | CD19 on transitional | rs10882701 | A | C | -0.194 | 0.027 | 1.87e-12 | 0.017 | 50 |
| MFI | CD19 on transitional | rs11188449 | G | A | -0.502 | 0.025 | 7.23e-82 | 0.118 | 391.42 |
| MFI | CD19 on transitional | rs2901833 | G | A | 0.184 | 0.028 | 3.22e-11 | 0.015 | 44.33 |
| MFI | CD19 on unsw mem | rs11731825 | A | G | -0.202 | 0.032 | 2.09e-10 | 0.011 | 40.61 |
| MFI | CD19 on unsw mem | rs145567900 | C | A | -0.919 | 0.116 | 2.51e-15 | 0.017 | 63.12 |
| MFI | CD19 on unsw mem | rs2203043 | G | A | 0.296 | 0.05 | 4.72e-09 | 0.009 | 34.45 |
| MFI | CD19 on unsw mem | rs548982606 | C | T | -0.665 | 0.087 | 2.32e-14 | 0.016 | 58.67 |
| MFI | CD19 on unsw mem | rs574746346 | G | A | -1.143 | 0.08 | 7.68e-45 | 0.053 | 202.85 |
| MFI | CD20 on CD20- CD38- | rs11074934 | C | T | -0.413 | 0.041 | 2.14e-23 | 0.059 | 102.34 |
| MFI | CD20 on CD20- CD38- | rs6934244 | C | A | 0.378 | 0.053 | 1.76e-12 | 0.030 | 50.44 |
| MFI | CD20 on CD20- CD38- | rs9268628 | A | C | -0.386 | 0.039 | 1.32e-22 | 0.057 | 98.5 |
| MFI | CD20 on CD20- CD38- | rs9274623 | G | T | -0.402 | 0.043 | 4.95e-20 | 0.050 | 86.13 |
| MFI | CD20 on IgD+ | rs114672530 | G | A | -0.618 | 0.105 | 3.99e-09 | 0.012 | 34.85 |
| MFI | CD20 on IgD+ | rs12138291 | G | A | -0.28 | 0.038 | 3.82e-13 | 0.018 | 53.21 |
| MFI | CD20 on IgD+ | rs16848876 | A | G | -0.551 | 0.094 | 5.09e-09 | 0.012 | 34.34 |
| MFI | CD20 on IgD+ | rs2949661 | C | T | 0.618 | 0.025 | 7.26e-122 | 0.172 | 606.11 |
| MFI | CD20 on IgD+ | rs35055340 | C | T | -0.493 | 0.077 | 1.50e-10 | 0.014 | 41.3 |
| MFI | CD20 on IgD+ | rs72703432 | A | G | -0.504 | 0.086 | 4.70e-09 | 0.012 | 34.5 |
| MFI | CD20 on IgD+ CD24+ | rs10305689 | C | T | 0.464 | 0.083 | 2.42e-08 | 0.009 | 31.24 |
| MFI | CD20 on IgD+ CD24+ | rs112755578 | A | G | -0.32 | 0.049 | 7.78e-11 | 0.012 | 42.55 |
| MFI | CD20 on IgD+ CD24+ | rs116275309 | G | A | 0.68 | 0.109 | 5.10e-10 | 0.011 | 38.85 |
| MFI | CD20 on IgD+ CD24+ | rs146963227 | G | A | -0.573 | 0.101 | 1.35e-08 | 0.009 | 32.4 |
| MFI | CD20 on IgD+ CD24+ | rs150145799 | T | C | 0.592 | 0.036 | 6.58e-58 | 0.069 | 266.62 |
| MFI | CD20 on IgD+ CD24+ | rs1801274 | A | G | 0.383 | 0.026 | 1.86e-48 | 0.058 | 220.37 |
| MFI | CD20 on IgD+ CD24+ | rs188268415 | C | T | -0.468 | 0.059 | 2.30e-15 | 0.017 | 63.31 |
| MFI | CD20 on IgD+ CD24+ | rs189981470 | T | C | 0.592 | 0.104 | 1.53e-08 | 0.009 | 32.15 |
| MFI | CD20 on IgD+ CD24+ | rs193176459 | A | G | 0.729 | 0.112 | 1.05e-10 | 0.011 | 41.97 |
| MFI | CD20 on IgD+ CD24+ | rs587655195 | T | C | -0.681 | 0.113 | 2.17e-09 | 0.010 | 36 |
| MFI | CD20 on IgD+ CD24+ | rs679692 | G | T | 0.486 | 0.08 | 1.16e-09 | 0.010 | 37.21 |
| MFI | CD20 on IgD+ CD38- naive | rs112421400 | C | T | -0.198 | 0.035 | 1.69e-08 | 0.009 | 31.96 |
| MFI | CD20 on IgD+ CD38- naive | rs139084170 | C | A | 0.359 | 0.058 | 5.51e-10 | 0.010 | 38.67 |
| MFI | CD20 on IgD+ CD38- naive | rs1794511 | T | G | -0.244 | 0.034 | 6.87e-13 | 0.014 | 51.95 |
| MFI | CD20 on IgD+ CD38- naive | rs709589 | C | T | -0.612 | 0.03 | 4.43e-87 | 0.101 | 412.76 |
| MFI | CD20 on IgD+ CD38- naive | rs73367236 | G | T | 0.549 | 0.09 | 1.17e-09 | 0.010 | 37.19 |
| MFI | CD20 on IgD+ CD38- naive | rs77578883 | A | G | -0.283 | 0.052 | 4.21e-08 | 0.008 | 30.17 |
| MFI | CD20 on IgD+ CD38- naive | rs9265515 | T | C | -0.221 | 0.032 | 1.20e-11 | 0.012 | 46.24 |
| MFI | CD20 on IgD+ CD38- unsw mem | rs13401874 | G | A | -0.531 | 0.096 | 2.89e-08 | 0.009 | 30.9 |
| MFI | CD20 on IgD+ CD38- unsw mem | rs62395272 | C | T | -0.23 | 0.037 | 3.91e-10 | 0.011 | 39.36 |
| MFI | CD20 on IgD+ CD38- unsw mem | rs9271296 | A | G | -0.303 | 0.03 | 4.43e-23 | 0.027 | 99.22 |
| MFI | CD20 on IgD+ CD38- unsw mem | rs9296042 | C | T | -0.192 | 0.029 | 5.86e-11 | 0.012 | 43.11 |
| MFI | CD20 on IgD- CD24- | rs142500247 | T | C | 0.469 | 0.084 | 2.16e-08 | 0.011 | 31.5 |
| MFI | CD20 on IgD- CD24- | rs34460712 | G | A | 0.2 | 0.036 | 3.17e-08 | 0.010 | 30.76 |
| MFI | CD20 on IgD- CD24- | rs3757247 | C | T | 0.153 | 0.027 | 2.34e-08 | 0.011 | 31.34 |
| MFI | CD20 on IgD- CD24- | rs62183989 | C | T | -0.416 | 0.034 | 3.31e-34 | 0.050 | 152.53 |
| MFI | CD20 on IgD- CD38- | rs10786281 | T | C | 0.174 | 0.029 | 2.18e-09 | 0.012 | 36.03 |
| MFI | CD20 on IgD- CD38- | rs117458836 | G | A | 0.898 | 0.105 | 2.34e-17 | 0.024 | 72.66 |
| MFI | CD20 on IgD- CD38- | rs12778618 | T | C | 0.591 | 0.067 | 1.47e-18 | 0.026 | 78.29 |
| MFI | CD20 on IgD- CD38- | rs4293064 | C | A | 0.268 | 0.049 | 4.45e-08 | 0.010 | 30.09 |
| MFI | CD20 on IgD- CD38- | rs7084635 | A | G | 0.181 | 0.031 | 6.90e-09 | 0.011 | 33.72 |
| MFI | CD20 on IgD- CD38- | rs7086986 | A | G | 0.491 | 0.026 | 5.68e-74 | 0.107 | 350.54 |
| MFI | CD20 on IgD- CD38- | rs9264532 | C | T | 0.185 | 0.031 | 1.61e-09 | 0.012 | 36.6 |
| MFI | CD20 on IgD- CD38dim | rs112266065 | C | T | -0.911 | 0.116 | 4.27e-15 | 0.017 | 62.07 |
| MFI | CD20 on IgD- CD38dim | rs114919524 | A | C | -1.14 | 0.107 | 3.08e-26 | 0.030 | 113.88 |
| MFI | CD20 on IgD- CD38dim | rs117630508 | G | A | -0.829 | 0.068 | 1.44e-33 | 0.039 | 148.67 |
| MFI | CD20 on IgD- CD38dim | rs141059303 | G | A | -0.672 | 0.117 | 1.04e-08 | 0.009 | 32.87 |
| MFI | CD20 on IgD- CD38dim | rs144365928 | A | G | -0.708 | 0.078 | 1.13e-19 | 0.022 | 83.27 |
| MFI | CD20 on IgD- CD38dim | rs144886236 | T | C | -1.508 | 0.034 | 1.00e-200 | 0.354 | 2004.84 |
| MFI | CD20 on IgD- CD38dim | rs148516965 | C | T | -0.57 | 0.056 | 5.32e-24 | 0.028 | 103.47 |
| MFI | CD20 on IgD- CD38dim | rs149167072 | A | G | 0.311 | 0.053 | 6.40e-09 | 0.009 | 33.85 |
| MFI | CD20 on IgD- CD38dim | rs1801274 | A | G | -0.169 | 0.027 | 3.64e-10 | 0.011 | 39.52 |
| MFI | CD20 on IgD- CD38dim | rs181079441 | C | T | -0.375 | 0.045 | 7.56e-17 | 0.019 | 70.18 |
| MFI | CD20 on IgD- CD38dim | rs184205740 | C | T | -1.227 | 0.116 | 1.11e-25 | 0.030 | 111.44 |
| MFI | CD20 on IgD- CD38dim | rs185098316 | G | A | -0.291 | 0.052 | 2.79e-08 | 0.008 | 30.97 |
| MFI | CD20 on IgD- CD38dim | rs185430947 | A | G | -1.041 | 0.108 | 1.24e-21 | 0.025 | 92.34 |
| MFI | CD20 on IgD- CD38dim | rs187065953 | T | C | -0.789 | 0.096 | 2.10e-16 | 0.018 | 68.1 |
| MFI | CD20 on IgD- CD38dim | rs188216171 | G | A | -0.803 | 0.11 | 3.29e-13 | 0.014 | 53.4 |
| MFI | CD20 on IgD- CD38dim | rs189799494 | G | T | -0.606 | 0.051 | 1.65e-32 | 0.038 | 143.64 |
| MFI | CD20 on IgD- CD38dim | rs191363479 | C | T | -1.046 | 0.109 | 2.00e-21 | 0.024 | 91.53 |
| MFI | CD20 on IgD- CD38dim | rs193039847 | G | A | -1.37 | 0.087 | 1.52e-54 | 0.064 | 250.07 |
| MFI | CD20 on IgD- CD38dim | rs535059510 | C | T | -0.864 | 0.118 | 3.08e-13 | 0.014 | 53.55 |
| MFI | CD20 on IgD- CD38dim | rs539130256 | T | C | -0.589 | 0.105 | 2.41e-08 | 0.008 | 31.25 |
| MFI | CD20 on IgD- CD38dim | rs539241708 | C | T | -1.201 | 0.085 | 4.07e-44 | 0.052 | 199.44 |
| MFI | CD20 on IgD- CD38dim | rs556260593 | A | C | -1.045 | 0.106 | 9.08e-23 | 0.026 | 97.87 |
| MFI | CD20 on IgD- CD38dim | rs557577522 | G | A | -0.768 | 0.071 | 5.22e-27 | 0.031 | 117.63 |
| MFI | CD20 on IgD- CD38dim | rs558720004 | A | C | -1.281 | 0.11 | 4.33e-31 | 0.036 | 136.78 |
| MFI | CD20 on IgD- CD38dim | rs568374507 | C | T | -0.492 | 0.059 | 7.92e-17 | 0.019 | 70.06 |
| MFI | CD20 on IgD- CD38dim | rs73888140 | G | A | -0.496 | 0.06 | 1.69e-16 | 0.018 | 68.52 |
| MFI | CD20 on IgD- CD38dim | rs78806227 | C | T | -0.987 | 0.12 | 3.42e-16 | 0.018 | 67.1 |
| MFI | CD20 on IgD- CD38dim | rs79085286 | T | C | -0.32 | 0.053 | 2.43e-09 | 0.010 | 35.75 |
| MFI | CD20 on naive-mature B cell | rs10905719 | G | A | 0.173 | 0.032 | 4.88e-08 | 0.009 | 29.87 |
| MFI | CD20 on naive-mature B cell | rs11124653 | C | A | -0.195 | 0.026 | 1.36e-13 | 0.016 | 55.18 |
| MFI | CD20 on naive-mature B cell | rs11589644 | C | T | 0.675 | 0.119 | 1.53e-08 | 0.009 | 32.15 |
| MFI | CD20 on naive-mature B cell | rs12712610 | A | G | 0.235 | 0.028 | 6.67e-17 | 0.020 | 70.47 |
| MFI | CD20 on naive-mature B cell | rs77634072 | C | T | 0.603 | 0.091 | 3.35e-11 | 0.013 | 44.22 |
| MFI | CD20 on transitional | rs111993863 | G | A | -0.913 | 0.066 | 3.90e-42 | 0.049 | 189.71 |
| MFI | CD20 on transitional | rs112266065 | C | T | -0.913 | 0.116 | 3.76e-15 | 0.017 | 62.32 |
| MFI | CD20 on transitional | rs117458132 | C | T | -0.322 | 0.05 | 9.97e-11 | 0.011 | 42.06 |
| MFI | CD20 on transitional | rs141059303 | G | A | -0.704 | 0.117 | 2.06e-09 | 0.010 | 36.07 |
| MFI | CD20 on transitional | rs143591969 | G | A | -1.337 | 0.038 | 1.00e-200 | 0.250 | 1217.95 |
| MFI | CD20 on transitional | rs144712270 | G | A | -0.629 | 0.11 | 1.28e-08 | 0.009 | 32.47 |
| MFI | CD20 on transitional | rs146434619 | T | C | -0.552 | 0.045 | 1.01e-33 | 0.039 | 149.43 |
| MFI | CD20 on transitional | rs149806587 | A | G | -0.522 | 0.057 | 1.44e-19 | 0.022 | 82.78 |
| MFI | CD20 on transitional | rs1801274 | A | G | -0.176 | 0.027 | 6.47e-11 | 0.012 | 42.9 |
| MFI | CD20 on transitional | rs184205740 | C | T | -1.234 | 0.116 | 5.69e-26 | 0.030 | 112.71 |
| MFI | CD20 on transitional | rs185098316 | G | A | -0.292 | 0.052 | 2.68e-08 | 0.008 | 31.05 |
| MFI | CD20 on transitional | rs191363479 | C | T | -1.021 | 0.109 | 1.79e-20 | 0.023 | 87.05 |
| MFI | CD20 on transitional | rs539130256 | T | C | -0.61 | 0.105 | 7.66e-09 | 0.009 | 33.49 |
| MFI | CD20 on transitional | rs539241708 | C | T | -1.207 | 0.085 | 1.48e-44 | 0.052 | 201.48 |
| MFI | CD20 on transitional | rs556260593 | A | C | -1.038 | 0.106 | 1.62e-22 | 0.026 | 96.38 |
| MFI | CD20 on transitional | rs558720004 | A | C | -1.296 | 0.109 | 8.55e-32 | 0.037 | 140.26 |
| MFI | CD20 on transitional | rs561787258 | G | A | -1.214 | 0.084 | 5.97e-46 | 0.054 | 208.26 |
| MFI | CD20 on transitional | rs570148043 | G | A | -1.018 | 0.108 | 1.04e-20 | 0.024 | 88.15 |
| MFI | CD20 on transitional | rs5759079 | G | A | -0.436 | 0.036 | 4.86e-33 | 0.038 | 146.17 |
| MFI | CD20 on transitional | rs576126454 | T | G | -1.384 | 0.086 | 1.18e-56 | 0.066 | 260.17 |
| MFI | CD20 on transitional | rs73888140 | G | A | -0.492 | 0.06 | 3.59e-16 | 0.018 | 67.02 |
| MFI | CD20 on transitional | rs79085286 | T | C | -0.323 | 0.053 | 1.72e-09 | 0.010 | 36.42 |
| MFI | CD24 on CD24+ CD27+ | rs139249541 | A | G | 0.462 | 0.073 | 2.44e-10 | 0.011 | 40.28 |
| MFI | CD24 on CD24+ CD27+ | rs144837535 | T | C | 0.526 | 0.072 | 2.36e-13 | 0.015 | 54.06 |
| MFI | CD24 on CD24+ CD27+ | rs150386792 | G | A | 0.568 | 0.075 | 4.62e-14 | 0.015 | 57.31 |
| MFI | CD24 on CD24+ CD27+ | rs181878688 | G | A | 0.651 | 0.068 | 3.33e-21 | 0.024 | 90.4 |
| MFI | CD24 on CD24+ CD27+ | rs186978368 | C | T | 0.525 | 0.073 | 9.20e-13 | 0.014 | 51.35 |
| MFI | CD24 on CD24+ CD27+ | rs557204100 | A | G | 0.49 | 0.089 | 3.89e-08 | 0.008 | 30.32 |
| MFI | CD24 on CD24+ CD27+ | rs56360439 | T | C | 0.17 | 0.031 | 3.43e-08 | 0.008 | 30.57 |
| MFI | CD24 on CD24+ CD27+ | rs57245661 | A | C | 0.543 | 0.094 | 8.47e-09 | 0.009 | 33.3 |
| MFI | CD24 on CD24+ CD27+ | rs62432266 | G | T | 0.365 | 0.053 | 8.75e-12 | 0.013 | 46.86 |
| MFI | CD24 on CD24+ CD27+ | rs75494211 | G | A | 0.471 | 0.07 | 2.22e-11 | 0.012 | 45.03 |
| MFI | CD24 on CD24+ CD27+ | rs78268116 | C | T | -0.409 | 0.039 | 2.79e-25 | 0.029 | 109.48 |
| MFI | CD24 on CD24+ CD27+ | rs9916629 | T | C | 0.146 | 0.025 | 4.13e-09 | 0.009 | 34.72 |
| MFI | CD24 on IgD+ CD38- unsw mem | rs12986962 | A | G | -0.157 | 0.028 | 3.58e-08 | 0.009 | 30.46 |
| MFI | CD24 on IgD+ CD38- unsw mem | rs144837535 | T | C | 0.446 | 0.073 | 1.46e-09 | 0.011 | 36.76 |
| MFI | CD24 on IgD+ CD38- unsw mem | rs150386792 | G | A | 0.472 | 0.077 | 1.02e-09 | 0.011 | 37.48 |
| MFI | CD24 on IgD+ CD38- unsw mem | rs181878688 | G | A | 0.509 | 0.07 | 5.84e-13 | 0.015 | 52.27 |
| MFI | CD24 on IgD+ CD38- unsw mem | rs186978368 | C | T | 0.424 | 0.075 | 1.89e-08 | 0.009 | 31.75 |
| MFI | CD24 on IgD+ CD38- unsw mem | rs58745116 | G | A | 0.166 | 0.026 | 1.05e-10 | 0.012 | 41.96 |
| MFI | CD24 on sw mem | rs35994952 | A | C | 0.596 | 0.096 | 5.76e-10 | 0.013 | 38.65 |
| MFI | CD24 on sw mem | rs4493054 | G | A | 0.361 | 0.03 | 1.22e-32 | 0.048 | 144.97 |
| MFI | CD24 on sw mem | rs560119329 | C | T | 0.52 | 0.089 | 6.32e-09 | 0.012 | 33.91 |
| MFI | CD24 on sw mem | rs563352400 | G | A | 0.674 | 0.11 | 9.87e-10 | 0.013 | 37.56 |
| MFI | CD24 on transitional | rs34256565 | T | C | -0.384 | 0.03 | 6.05e-36 | 0.042 | 159.91 |
| MFI | CD24 on transitional | rs9270657 | T | G | -0.172 | 0.03 | 6.22e-09 | 0.009 | 33.91 |
| MFI | CD24 on transitional | rs9461744 | A | G | 0.283 | 0.034 | 1.38e-16 | 0.018 | 68.97 |
| MFI | CD25 on CD45RA- CD4 not Treg | rs112431762 | G | A | 0.643 | 0.074 | 1.01e-17 | 0.046 | 75.18 |
| MFI | CD25 on CD45RA- CD4 not Treg | rs138712525 | G | A | 0.343 | 0.046 | 1.22e-13 | 0.035 | 55.91 |
| MFI | CD25 on CD45RA- CD4 not Treg | rs139663572 | C | A | -0.721 | 0.109 | 5.28e-11 | 0.027 | 43.63 |
| MFI | CD25 on CD45RA- CD4 not Treg | rs140923781 | C | T | -0.659 | 0.113 | 6.19e-09 | 0.021 | 34.08 |
| MFI | CD25 on CD45RA- CD4 not Treg | rs2459145 | T | C | 1.359 | 0.244 | 3.05e-08 | 0.020 | 30.96 |
| MFI | CD25 on CD45RA- CD4 not Treg | rs3865444 | C | A | -1.138 | 0.04 | 2.60e-145 | 0.345 | 818.98 |
| MFI | CD25 on CD45RA- CD4 not Treg | rs56043070 | G | A | 0.501 | 0.078 | 2.26e-10 | 0.026 | 40.72 |
| MFI | CD25 on CD45RA- CD4 not Treg | rs7351079 | A | G | -0.364 | 0.042 | 1.15e-17 | 0.046 | 74.9 |
| MFI | CD25 on CD45RA- CD4 not Treg | rs73932869 | G | A | -0.899 | 0.16 | 2.45e-08 | 0.020 | 31.4 |
| MFI | CD25 on CD45RA- CD4 not Treg | rs75773078 | G | A | -0.492 | 0.07 | 4.12e-12 | 0.030 | 48.77 |
| MFI | CD25 on B cell | rs13390252 | A | G | -0.48 | 0.034 | 5.58e-45 | 0.066 | 204.85 |
| MFI | CD25 on B cell | rs59639671 | C | T | 0.196 | 0.036 | 4.70e-08 | 0.010 | 29.96 |
| MFI | CD25 on B cell | rs72923091 | G | A | 0.176 | 0.029 | 2.09e-09 | 0.012 | 36.1 |
| MFI | CD25 on CD20- CD38- | rs10882701 | A | C | -0.354 | 0.025 | 1.32e-44 | 0.056 | 201.94 |
| MFI | CD25 on CD20- CD38- | rs11596570 | C | T | -0.312 | 0.056 | 2.57e-08 | 0.009 | 31.14 |
| MFI | CD25 on CD20- CD38- | rs141018090 | G | T | 0.446 | 0.055 | 1.08e-15 | 0.019 | 64.84 |
| MFI | CD25 on CD20- CD38- | rs145557266 | A | G | 0.607 | 0.089 | 1.37e-11 | 0.013 | 46.01 |
| MFI | CD25 on CD20- CD38- | rs191819784 | G | A | 0.564 | 0.076 | 1.60e-13 | 0.016 | 54.85 |
| MFI | CD25 on CD20- CD38- | rs202211780 | A | G | 0.442 | 0.045 | 2.46e-22 | 0.027 | 95.78 |
| MFI | CD25 on CD20- CD38- | rs7094382 | G | A | 0.914 | 0.02 | 1.00e-200 | 0.370 | 2021.39 |
| MFI | CD25 on CD39+ CD4 Treg | rs181878688 | G | A | -0.434 | 0.068 | 1.99e-10 | 0.012 | 40.71 |
| MFI | CD25 on CD39+ CD4 Treg | rs2367376 | T | C | 0.172 | 0.029 | 2.85e-09 | 0.010 | 35.46 |
| MFI | CD25 on CD39+ CD4 Treg | rs7559619 | A | G | -0.179 | 0.026 | 1.27e-11 | 0.013 | 46.15 |
| MFI | CD25 on CD39+ CD4 Treg | rs77745909 | A | G | 0.694 | 0.116 | 2.17e-09 | 0.010 | 35.99 |
| MFI | CD25 on CD39+ activated Treg | rs117295081 | A | G | 0.55 | 0.097 | 1.29e-08 | 0.009 | 32.48 |
| MFI | CD25 on CD39+ activated Treg | rs1801274 | A | G | 0.437 | 0.026 | 1.62e-62 | 0.074 | 289.33 |
| MFI | CD25 on CD39+ activated Treg | rs186463618 | C | A | 0.434 | 0.065 | 3.94e-11 | 0.012 | 43.89 |
| MFI | CD25 on CD39+ activated Treg | rs60629714 | T | C | 0.186 | 0.032 | 4.55e-09 | 0.009 | 34.51 |
| MFI | CD25 on CD39+ activated Treg | rs745307 | G | A | 0.664 | 0.033 | 2.07e-83 | 0.098 | 394.29 |
| MFI | CD25 on CD39+ secreting Treg | rs1801274 | A | G | -0.552 | 0.034 | 2.53e-54 | 0.122 | 257.08 |
| MFI | CD25 on CD39+ secreting Treg | rs3820097 | G | A | 0.429 | 0.074 | 8.69e-09 | 0.018 | 33.39 |
| MFI | CD25 on CD39+ secreting Treg | rs3826007 | C | T | -0.354 | 0.04 | 3.71e-18 | 0.040 | 76.97 |
| MFI | CD25 on CD45RA+ CD4 not Treg | rs11124653 | C | A | -0.186 | 0.027 | 3.74e-12 | 0.014 | 48.55 |
| MFI | CD25 on CD45RA+ CD4 not Treg | rs12712610 | A | G | 0.235 | 0.028 | 2.15e-16 | 0.020 | 68.11 |
| MFI | CD25 on CD45RA+ CD4 not Treg | rs77634072 | C | T | 0.637 | 0.092 | 6.39e-12 | 0.014 | 47.51 |
| MFI | CD25 on IgD+ | rs3130913 | G | A | 0.184 | 0.03 | 5.83e-10 | 0.011 | 38.56 |
| MFI | CD25 on IgD+ | rs6808893 | C | T | -0.204 | 0.025 | 3.27e-16 | 0.018 | 67.23 |
| MFI | CD25 on IgD+ | rs6934244 | C | A | 0.328 | 0.039 | 9.31e-17 | 0.019 | 69.77 |
| MFI | CD25 on IgD+ | rs73499473 | C | T | 0.38 | 0.029 | 1.91e-38 | 0.045 | 171.93 |
| MFI | CD25 on IgD+ | rs9268628 | A | C | -0.408 | 0.028 | 1.52e-45 | 0.054 | 206.16 |
| MFI | CD25 on IgD+ | rs9274623 | G | T | -0.373 | 0.032 | 5.30e-31 | 0.036 | 136.52 |
| MFI | CD25 on IgD+ | rs9927195 | A | G | 0.148 | 0.026 | 2.33e-08 | 0.009 | 31.32 |
| MFI | CD25 on IgD+ CD24+ | rs112755578 | A | G | -0.446 | 0.049 | 3.16e-19 | 0.022 | 81.2 |
| MFI | CD25 on IgD+ CD24+ | rs116275309 | G | A | 0.823 | 0.11 | 1.15e-13 | 0.015 | 55.53 |
| MFI | CD25 on IgD+ CD24+ | rs145828126 | G | A | -0.323 | 0.059 | 4.73e-08 | 0.008 | 29.94 |
| MFI | CD25 on IgD+ CD24+ | rs149020192 | C | T | 0.575 | 0.084 | 8.94e-12 | 0.013 | 46.84 |
| MFI | CD25 on IgD+ CD24+ | rs150145799 | T | C | 0.718 | 0.036 | 2.76e-83 | 0.098 | 393.51 |
| MFI | CD25 on IgD+ CD24+ | rs1801274 | A | G | 0.394 | 0.026 | 8.17e-50 | 0.059 | 227 |
| MFI | CD25 on IgD+ CD24+ | rs181465742 | G | T | 0.582 | 0.1 | 6.74e-09 | 0.009 | 33.73 |
| MFI | CD25 on IgD+ CD24+ | rs188268415 | C | T | -0.557 | 0.059 | 1.25e-20 | 0.024 | 87.74 |
| MFI | CD25 on IgD+ CD24+ | rs189981470 | T | C | 0.668 | 0.105 | 2.43e-10 | 0.011 | 40.26 |
| MFI | CD25 on IgD+ CD24+ | rs190362224 | C | T | -0.669 | 0.111 | 1.78e-09 | 0.010 | 36.34 |
| MFI | CD25 on IgD+ CD24+ | rs193176459 | A | G | 0.913 | 0.114 | 1.45e-15 | 0.017 | 64.22 |
| MFI | CD25 on IgD+ CD24+ | rs587760652 | A | G | 0.735 | 0.128 | 1.06e-08 | 0.009 | 32.89 |
| MFI | CD25 on IgD+ CD24+ | rs604983 | G | A | 0.606 | 0.08 | 6.04e-14 | 0.015 | 56.77 |
| MFI | CD25 on IgD+ CD24+ | rs80178684 | A | G | -0.649 | 0.118 | 3.65e-08 | 0.008 | 30.46 |
| MFI | CD25 on IgD+ CD38- | rs112266065 | C | T | -1.035 | 0.115 | 4.17e-19 | 0.022 | 80.53 |
| MFI | CD25 on IgD+ CD38- | rs114919524 | A | C | -1.124 | 0.107 | 1.56e-25 | 0.029 | 110.7 |
| MFI | CD25 on IgD+ CD38- | rs117119882 | C | A | -0.932 | 0.116 | 1.05e-15 | 0.017 | 64.92 |
| MFI | CD25 on IgD+ CD38- | rs141059303 | G | A | -0.643 | 0.117 | 4.16e-08 | 0.008 | 30.18 |
| MFI | CD25 on IgD+ CD38- | rs144365928 | A | G | -0.683 | 0.078 | 2.29e-18 | 0.021 | 77.19 |
| MFI | CD25 on IgD+ CD38- | rs148516965 | C | T | -0.562 | 0.056 | 2.27e-23 | 0.027 | 100.55 |
| MFI | CD25 on IgD+ CD38- | rs149167072 | A | G | 0.347 | 0.053 | 9.52e-11 | 0.011 | 42.13 |
| MFI | CD25 on IgD+ CD38- | rs149806587 | A | G | -0.526 | 0.057 | 8.41e-20 | 0.022 | 83.86 |
| MFI | CD25 on IgD+ CD38- | rs150288192 | C | T | -1.224 | 0.117 | 2.08e-25 | 0.029 | 110.14 |
| MFI | CD25 on IgD+ CD38- | rs185098316 | G | A | -0.308 | 0.052 | 4.25e-09 | 0.009 | 34.66 |
| MFI | CD25 on IgD+ CD38- | rs185430947 | A | G | -0.998 | 0.108 | 5.45e-20 | 0.023 | 84.72 |
| MFI | CD25 on IgD+ CD38- | rs188216171 | G | A | -0.921 | 0.11 | 5.91e-17 | 0.019 | 70.65 |
| MFI | CD25 on IgD+ CD38- | rs189799494 | G | T | -0.656 | 0.05 | 9.55e-38 | 0.044 | 168.65 |
| MFI | CD25 on IgD+ CD38- | rs191363479 | C | T | -1.002 | 0.109 | 9.03e-20 | 0.022 | 83.84 |
| MFI | CD25 on IgD+ CD38- | rs193039847 | G | A | -1.396 | 0.086 | 1.22e-56 | 0.066 | 260.32 |
| MFI | CD25 on IgD+ CD38- | rs535059510 | C | T | -0.983 | 0.118 | 9.47e-17 | 0.019 | 69.71 |
| MFI | CD25 on IgD+ CD38- | rs539130256 | T | C | -0.611 | 0.105 | 7.31e-09 | 0.009 | 33.59 |
| MFI | CD25 on IgD+ CD38- | rs539241708 | C | T | -1.243 | 0.085 | 3.09e-47 | 0.055 | 214.49 |
| MFI | CD25 on IgD+ CD38- | rs556260593 | A | C | -1.02 | 0.106 | 8.35e-22 | 0.025 | 93.07 |
| MFI | CD25 on IgD+ CD38- | rs557577522 | G | A | -0.805 | 0.071 | 1.43e-29 | 0.034 | 129.71 |
| MFI | CD25 on IgD+ CD38- | rs566910078 | G | A | -1.261 | 0.11 | 3.53e-30 | 0.035 | 132.55 |
| MFI | CD25 on IgD+ CD38- | rs568374507 | C | T | -0.571 | 0.059 | 4.09e-22 | 0.025 | 94.64 |
| MFI | CD25 on IgD+ CD38- | rs5758649 | C | T | -0.602 | 0.045 | 3.19e-40 | 0.047 | 180.49 |
| MFI | CD25 on IgD+ CD38- | rs73165139 | A | G | -1.396 | 0.034 | 1.00e-200 | 0.316 | 1688.88 |
| MFI | CD25 on IgD+ CD38- | rs73888140 | G | A | -0.574 | 0.06 | 1.67e-21 | 0.024 | 91.78 |
| MFI | CD25 on IgD+ CD38- | rs78806227 | C | T | -0.963 | 0.12 | 1.62e-15 | 0.017 | 64.04 |
| MFI | CD25 on IgD+ CD38- | rs79085286 | T | C | -0.368 | 0.053 | 6.88e-12 | 0.013 | 47.35 |
| MFI | CD25 on IgD+ CD38- unsw mem | rs116798506 | T | C | -0.611 | 0.086 | 1.14e-12 | 0.014 | 50.91 |
| MFI | CD25 on IgD+ CD38- unsw mem | rs145567900 | C | A | -0.834 | 0.118 | 2.31e-12 | 0.013 | 49.52 |
| MFI | CD25 on IgD+ CD38- unsw mem | rs548982606 | C | T | -0.549 | 0.089 | 6.08e-10 | 0.010 | 38.49 |
| MFI | CD25 on IgD+ CD38- unsw mem | rs574746346 | G | A | -1.017 | 0.082 | 1.35e-34 | 0.040 | 153.51 |
| MFI | CD25 on IgD+ CD38- unsw mem | rs807784 | G | T | 0.317 | 0.052 | 8.42e-10 | 0.010 | 37.85 |
| MFI | CD25 on IgD- CD24- | rs111695695 | C | A | -0.545 | 0.068 | 2.04e-15 | 0.040 | 64.27 |
| MFI | CD25 on IgD- CD24- | rs191091405 | C | T | 0.573 | 0.066 | 9.45e-18 | 0.046 | 75.3 |
| MFI | CD25 on IgD- CD24- | rs2459145 | T | C | 1.404 | 0.252 | 2.94e-08 | 0.020 | 31.05 |
| MFI | CD25 on IgD- CD24- | rs56043070 | G | A | 0.488 | 0.078 | 6.30e-10 | 0.024 | 38.66 |
| MFI | CD25 on IgD- CD24- | rs7245846 | G | A | -1.131 | 0.039 | 1.81e-150 | 0.356 | 856.97 |
| MFI | CD25 on IgD- CD24- | rs7351079 | A | G | -0.364 | 0.042 | 1.40e-17 | 0.046 | 74.49 |
| MFI | CD25 on IgD- CD24- | rs73932869 | G | A | -0.923 | 0.164 | 2.31e-08 | 0.020 | 31.5 |
| MFI | CD25 on IgD- CD24- | rs75773078 | G | A | -0.492 | 0.071 | 4.53e-12 | 0.030 | 48.59 |
| MFI | CD25 on IgD- CD24- | rs79377141 | G | A | 0.342 | 0.046 | 1.26e-13 | 0.035 | 55.82 |
| MFI | CD25 on IgD- CD38- | rs28669075 | C | T | -0.211 | 0.025 | 5.47e-17 | 0.019 | 70.81 |
| MFI | CD25 on IgD- CD38- | rs28723652 | C | A | 0.414 | 0.074 | 1.89e-08 | 0.009 | 31.72 |
| MFI | CD25 on IgD- CD38- | rs35501848 | G | T | -0.165 | 0.025 | 5.73e-11 | 0.012 | 43.17 |
| MFI | CD25 on IgD- CD38- | rs709589 | C | T | -0.624 | 0.03 | 9.25e-90 | 0.105 | 426.7 |
| MFI | CD25 on IgD- CD38br | rs112266065 | C | T | -0.891 | 0.116 | 1.83e-14 | 0.016 | 59.14 |
| MFI | CD25 on IgD- CD38br | rs114919524 | A | C | -1.133 | 0.107 | 7.68e-26 | 0.030 | 112.27 |
| MFI | CD25 on IgD- CD38br | rs117630508 | G | A | -0.824 | 0.068 | 4.84e-33 | 0.038 | 146.17 |
| MFI | CD25 on IgD- CD38br | rs144365928 | A | G | -0.724 | 0.078 | 1.90e-20 | 0.023 | 86.87 |
| MFI | CD25 on IgD- CD38br | rs148516965 | C | T | -0.574 | 0.056 | 3.10e-24 | 0.028 | 104.56 |
| MFI | CD25 on IgD- CD38br | rs149167072 | A | G | 0.307 | 0.054 | 1.11e-08 | 0.009 | 32.77 |
| MFI | CD25 on IgD- CD38br | rs1801274 | A | G | -0.187 | 0.027 | 5.25e-12 | 0.013 | 47.88 |
| MFI | CD25 on IgD- CD38br | rs181079441 | C | T | -0.372 | 0.045 | 1.79e-16 | 0.018 | 68.42 |
| MFI | CD25 on IgD- CD38br | rs184205740 | C | T | -1.196 | 0.116 | 2.10e-24 | 0.028 | 105.34 |
| MFI | CD25 on IgD- CD38br | rs185430947 | A | G | -1.03 | 0.108 | 4.01e-21 | 0.024 | 90.07 |
| MFI | CD25 on IgD- CD38br | rs187065953 | T | C | -0.788 | 0.096 | 2.62e-16 | 0.018 | 67.66 |
| MFI | CD25 on IgD- CD38br | rs188216171 | G | A | -0.787 | 0.11 | 1.05e-12 | 0.014 | 51.05 |
| MFI | CD25 on IgD- CD38br | rs189799494 | G | T | -0.601 | 0.051 | 7.61e-32 | 0.037 | 140.46 |
| MFI | CD25 on IgD- CD38br | rs191363479 | C | T | -1.032 | 0.11 | 7.76e-21 | 0.024 | 88.61 |
| MFI | CD25 on IgD- CD38br | rs193039847 | G | A | -1.367 | 0.087 | 3.88e-54 | 0.064 | 248.06 |
| MFI | CD25 on IgD- CD38br | rs535059510 | C | T | -0.851 | 0.118 | 7.33e-13 | 0.014 | 51.79 |
| MFI | CD25 on IgD- CD38br | rs539130256 | T | C | -0.608 | 0.106 | 9.28e-09 | 0.009 | 33.1 |
| MFI | CD25 on IgD- CD38br | rs539241708 | C | T | -1.188 | 0.085 | 4.49e-43 | 0.050 | 194.27 |
| MFI | CD25 on IgD- CD38br | rs556260593 | A | C | -1.044 | 0.106 | 1.07e-22 | 0.026 | 97.32 |
| MFI | CD25 on IgD- CD38br | rs557577522 | G | A | -0.765 | 0.071 | 1.02e-26 | 0.031 | 116.26 |
| MFI | CD25 on IgD- CD38br | rs558720004 | A | C | -1.258 | 0.11 | 6.34e-30 | 0.035 | 131.43 |
| MFI | CD25 on IgD- CD38br | rs568374507 | C | T | -0.485 | 0.059 | 2.65e-16 | 0.018 | 67.63 |
| MFI | CD25 on IgD- CD38br | rs73888140 | G | A | -0.49 | 0.06 | 5.17e-16 | 0.018 | 66.31 |
| MFI | CD25 on IgD- CD38br | rs76140449 | T | C | -1.322 | 0.033 | 1.00e-200 | 0.301 | 1575.2 |
| MFI | CD25 on IgD- CD38br | rs78806227 | C | T | -0.97 | 0.12 | 1.14e-15 | 0.017 | 64.76 |
| MFI | CD25 on IgD- CD38br | rs79085286 | T | C | -0.319 | 0.054 | 2.93e-09 | 0.010 | 35.39 |
| MFI | CD25 on IgD- CD38dim | rs12901644 | C | T | -0.168 | 0.031 | 3.80e-08 | 0.009 | 30.38 |
| MFI | CD25 on IgD- CD38dim | rs3131627 | A | G | 0.126 | 0.021 | 2.26e-09 | 0.010 | 35.92 |
| MFI | CD25 on IgD- CD38dim | rs6729180 | C | T | 0.1 | 0.018 | 3.85e-08 | 0.009 | 30.37 |
| MFI | CD25 on activated & secreting Treg | rs112505169 | C | T | 0.868 | 0.108 | 1.54e-15 | 0.018 | 64.15 |
| MFI | CD25 on activated & secreting Treg | rs11589644 | C | T | 0.834 | 0.112 | 1.06e-13 | 0.016 | 55.63 |
| MFI | CD25 on activated & secreting Treg | rs17607399 | C | T | 0.218 | 0.039 | 2.77e-08 | 0.009 | 30.99 |
| MFI | CD25 on activated & secreting Treg | rs183949931 | T | C | 0.691 | 0.076 | 1.68e-19 | 0.023 | 82.52 |
| MFI | CD25 on activated & secreting Treg | rs28570998 | C | T | 0.415 | 0.076 | 4.94e-08 | 0.009 | 29.85 |
| MFI | CD25 on activated & secreting Treg | rs413431 | A | G | -0.294 | 0.031 | 9.54e-21 | 0.025 | 88.33 |
| MFI | CD25 on activated & secreting Treg | rs6751481 | T | C | 0.289 | 0.024 | 5.49e-33 | 0.041 | 146.09 |
| MFI | CD25 on activated Treg | rs11882720 | T | G | -0.476 | 0.068 | 3.79e-12 | 0.029 | 48.89 |
| MFI | CD25 on activated Treg | rs150952234 | G | A | 0.473 | 0.07 | 1.89e-11 | 0.027 | 45.67 |
| MFI | CD25 on activated Treg | rs2459145 | T | C | 1.52 | 0.207 | 3.12e-13 | 0.032 | 53.96 |
| MFI | CD25 on activated Treg | rs3865444 | C | A | -1.143 | 0.034 | 1.05e-190 | 0.412 | 1143.51 |
| MFI | CD25 on activated Treg | rs558625031 | G | A | -0.634 | 0.082 | 1.67e-14 | 0.035 | 59.91 |
| MFI | CD25 on activated Treg | rs7351079 | A | G | -0.401 | 0.04 | 7.98e-23 | 0.058 | 99.58 |
| MFI | CD25 on activated Treg | rs78949787 | C | T | 0.283 | 0.036 | 6.32e-15 | 0.037 | 61.87 |
| MFI | CD25 on memory B cell | rs2066399 | G | A | 0.335 | 0.032 | 1.13e-25 | 0.030 | 111.33 |
| MFI | CD25 on memory B cell | rs34486765 | G | A | 0.168 | 0.026 | 1.75e-10 | 0.011 | 40.95 |
| MFI | CD25 on memory B cell | rs542874068 | G | A | -0.418 | 0.071 | 4.97e-09 | 0.009 | 34.35 |
| MFI | CD25 on memory B cell | rs72836542 | C | T | 0.149 | 0.027 | 2.45e-08 | 0.008 | 31.2 |
| MFI | CD25 on memory B cell | rs7768875 | C | T | -0.171 | 0.031 | 3.05e-08 | 0.008 | 30.8 |
| MFI | CD25 on memory B cell | rs7939177 | C | T | -0.178 | 0.025 | 6.73e-13 | 0.014 | 51.99 |
| MFI | CD25 on transitional | rs2169052 | T | C | 0.236 | 0.04 | 2.75e-09 | 0.012 | 35.56 |
| MFI | CD25 on transitional | rs35587265 | A | G | -0.422 | 0.059 | 7.75e-13 | 0.018 | 51.77 |
| MFI | CD25 on transitional | rs55971447 | C | T | -0.41 | 0.032 | 2.59e-37 | 0.055 | 167.56 |
| MFI | CD25 on unsw mem | rs114293843 | C | T | 0.435 | 0.077 | 2.04e-08 | 0.011 | 31.61 |
| MFI | CD25 on unsw mem | rs35994952 | A | C | 0.596 | 0.096 | 6.18e-10 | 0.013 | 38.49 |
| MFI | CD25 on unsw mem | rs560119329 | C | T | 0.554 | 0.09 | 7.09e-10 | 0.013 | 38.23 |
| MFI | CD25 on unsw mem | rs563352400 | G | A | 0.702 | 0.11 | 2.27e-10 | 0.014 | 40.52 |
| MFI | CD25 on unsw mem | rs61802329 | C | T | 0.167 | 0.027 | 9.48e-10 | 0.013 | 37.68 |
| MFI | CD25 on unsw mem | rs67898294 | C | T | 0.328 | 0.028 | 7.19e-31 | 0.045 | 136.5 |
| MFI | CD25 on unsw mem | rs75841521 | G | A | -0.442 | 0.081 | 4.65e-08 | 0.010 | 30 |
| MFI | CD25 on unsw mem | rs79298699 | C | T | -0.279 | 0.048 | 7.22e-09 | 0.012 | 33.66 |
| MFI | CD27 on CD20- | rs11882720 | T | G | -0.441 | 0.072 | 9.25e-10 | 0.023 | 37.89 |
| MFI | CD27 on CD20- | rs3865444 | C | A | -1.219 | 0.036 | 2.80e-187 | 0.417 | 1128.14 |
| MFI | CD27 on CD20- | rs558625031 | G | A | -0.71 | 0.085 | 1.33e-16 | 0.042 | 69.85 |
| MFI | CD27 on CD20- | rs7351079 | A | G | -0.337 | 0.042 | 2.54e-15 | 0.039 | 63.78 |
| MFI | CD27 on CD20- | rs78488761 | C | T | 0.402 | 0.071 | 2.16e-08 | 0.020 | 31.63 |
| MFI | CD27 on CD20- | rs78949787 | C | T | 0.294 | 0.037 | 3.54e-15 | 0.038 | 63.11 |
| MFI | CD27 on CD24+ CD27+ | rs144398648 | C | T | 0.659 | 0.119 | 3.48e-08 | 0.008 | 30.52 |
| MFI | CD27 on CD24+ CD27+ | rs2066399 | G | A | 0.226 | 0.032 | 2.95e-12 | 0.013 | 49.05 |
| MFI | CD27 on CD24+ CD27+ | rs4939384 | A | G | -0.205 | 0.025 | 2.29e-16 | 0.018 | 67.92 |
| MFI | CD27 on CD24+ CD27+ | rs62405562 | A | G | 0.194 | 0.029 | 1.15e-11 | 0.013 | 46.31 |
| MFI | CD27 on CD24+ CD27+ | rs72836542 | C | T | 0.164 | 0.027 | 9.87e-10 | 0.010 | 37.52 |
| MFI | CD27 on CD24+ CD27+ | rs9270560 | T | C | -0.168 | 0.03 | 1.31e-08 | 0.009 | 32.45 |
| MFI | CD27 on IgD+ CD24+ | rs113013837 | A | G | 0.783 | 0.103 | 3.47e-14 | 0.016 | 57.9 |
| MFI | CD27 on IgD+ CD24+ | rs137965091 | G | A | 0.385 | 0.068 | 1.32e-08 | 0.009 | 32.43 |
| MFI | CD27 on IgD+ CD24+ | rs139732336 | C | T | 0.563 | 0.091 | 5.58e-10 | 0.010 | 38.65 |
| MFI | CD27 on IgD+ CD24+ | rs150816467 | T | C | 0.579 | 0.092 | 3.00e-10 | 0.011 | 39.87 |
| MFI | CD27 on IgD+ CD24+ | rs28739016 | T | G | 0.876 | 0.102 | 1.34e-17 | 0.020 | 73.61 |
| MFI | CD27 on IgD- CD38br | rs1051488 | C | T | 0.24 | 0.033 | 8.82e-13 | 0.015 | 51.45 |
| MFI | CD27 on IgD- CD38br | rs2105899 | T | C | -0.236 | 0.035 | 1.47e-11 | 0.013 | 45.87 |
| MFI | CD27 on IgD- CD38br | rs7667122 | T | C | -0.15 | 0.027 | 4.33e-08 | 0.009 | 30.11 |
| MFI | CD27 on IgD- CD38br | rs77745909 | A | G | -0.823 | 0.117 | 2.25e-12 | 0.014 | 49.57 |
| MFI | CD27 on unsw mem | rs10228844 | A | G | -13.39 | 1.914 | 3.23e-12 | 0.014 | 48.91 |
| MFI | CD27 on unsw mem | rs1121709 | G | A | -35.11 | 5.793 | 1.50e-09 | 0.011 | 36.71 |
| MFI | CD27 on unsw mem | rs1150563 | T | C | -26.19 | 4.157 | 3.32e-10 | 0.011 | 39.67 |
| MFI | CD27 on unsw mem | rs116110730 | G | A | -53.46 | 5.651 | 5.53e-21 | 0.025 | 89.44 |
| MFI | CD27 on unsw mem | rs118120777 | A | G | 4.435 | 0.763 | 6.72e-09 | 0.010 | 33.77 |
| MFI | CD27 on unsw mem | rs143809079 | G | A | 4.024 | 0.732 | 4.10e-08 | 0.009 | 30.22 |
| MFI | CD27 on unsw mem | rs147004969 | A | G | -52.41 | 5.762 | 1.56e-19 | 0.024 | 82.69 |
| MFI | CD27 on unsw mem | rs150382715 | T | C | -29.2 | 3.702 | 4.09e-15 | 0.018 | 62.18 |
| MFI | CD27 on unsw mem | rs183791586 | A | G | 3.988 | 0.692 | 8.81e-09 | 0.010 | 33.23 |
| MFI | CD27 on unsw mem | rs188592444 | C | T | 3.913 | 0.706 | 3.14e-08 | 0.009 | 30.74 |
| MFI | CD27 on unsw mem | rs2054143 | A | G | -35.15 | 5.793 | 1.44e-09 | 0.011 | 36.8 |
| MFI | CD27 on unsw mem | rs237926 | T | C | -13.86 | 2.11 | 5.80e-11 | 0.012 | 43.12 |
| MFI | CD27 on unsw mem | rs334513 | A | G | -15.69 | 2.129 | 2.17e-13 | 0.016 | 54.28 |
| MFI | CD27 on unsw mem | rs4265908 | G | A | -54.48 | 5.136 | 6.94e-26 | 0.032 | 112.45 |
| MFI | CD27 on unsw mem | rs4712874 | C | T | -28.79 | 4.287 | 2.19e-11 | 0.013 | 45.07 |
| MFI | CD27 on unsw mem | rs4752660 | C | T | -28.86 | 4.285 | 1.90e-11 | 0.013 | 45.34 |
| MFI | CD27 on unsw mem | rs62636633 | C | T | -26.04 | 4.037 | 1.27e-10 | 0.012 | 41.58 |
| MFI | CD27 on unsw mem | rs6424838 | T | C | -52.41 | 5.762 | 1.56e-19 | 0.024 | 82.69 |
| MFI | CD27 on unsw mem | rs6457917 | A | C | -48.87 | 5.482 | 7.86e-19 | 0.023 | 79.42 |
| MFI | CD27 on unsw mem | rs72557975 | G | A | -49.1 | 5.705 | 1.14e-17 | 0.021 | 74.03 |
| MFI | CD27 on unsw mem | rs73311647 | C | T | -26.04 | 4.037 | 1.27e-10 | 0.012 | 41.58 |
| MFI | CD27 on unsw mem | rs73369750 | A | G | -22.8 | 3.48 | 6.51e-11 | 0.012 | 42.9 |
| MFI | CD27 on unsw mem | rs8099106 | A | G | -42 | 6.021 | 3.63e-12 | 0.014 | 48.63 |
| MFI | CD27 on unsw mem | rs947742 | A | G | -51.33 | 5.506 | 1.95e-20 | 0.025 | 86.86 |
| MFI | CD28 on CD28+ CD4+ | rs11961777 | T | C | 0.457 | 0.056 | 3.84e-16 | 0.018 | 66.89 |
| MFI | CD28 on CD28+ CD4+ | rs1800973 | C | A | -0.312 | 0.046 | 1.06e-11 | 0.013 | 46.49 |
| MFI | CD28 on CD28+ CD4+ | rs9270585 | C | T | -0.605 | 0.028 | 3.03e-98 | 0.115 | 470.58 |
| MFI | CD28 on CD28+ CD4+ | rs9469245 | C | T | -0.267 | 0.028 | 2.52e-21 | 0.024 | 90.95 |
| MFI | CD28 on CD28+ CD45RA+ CD8br | rs112062469 | G | A | -0.509 | 0.058 | 2.17e-18 | 0.021 | 77.28 |
| MFI | CD28 on CD28+ CD45RA+ CD8br | rs112266065 | C | T | -0.976 | 0.115 | 3.55e-17 | 0.019 | 71.67 |
| MFI | CD28 on CD28+ CD45RA+ CD8br | rs114919524 | A | C | -1.098 | 0.107 | 1.65e-24 | 0.028 | 105.84 |
| MFI | CD28 on CD28+ CD45RA+ CD8br | rs117119882 | C | A | -0.901 | 0.116 | 7.93e-15 | 0.016 | 60.81 |
| MFI | CD28 on CD28+ CD45RA+ CD8br | rs144365928 | A | G | -0.715 | 0.077 | 4.66e-20 | 0.023 | 85.06 |
| MFI | CD28 on CD28+ CD45RA+ CD8br | rs146434619 | T | C | -0.647 | 0.045 | 3.33e-46 | 0.054 | 209.34 |
| MFI | CD28 on CD28+ CD45RA+ CD8br | rs148516965 | C | T | -0.542 | 0.056 | 7.26e-22 | 0.025 | 93.49 |
| MFI | CD28 on CD28+ CD45RA+ CD8br | rs149167072 | A | G | 0.312 | 0.053 | 5.60e-09 | 0.009 | 34.1 |
| MFI | CD28 on CD28+ CD45RA+ CD8br | rs149806587 | A | G | -0.54 | 0.057 | 6.65e-21 | 0.024 | 89.02 |
| MFI | CD28 on CD28+ CD45RA+ CD8br | rs184205740 | C | T | -1.151 | 0.116 | 7.27e-23 | 0.026 | 98.06 |
| MFI | CD28 on CD28+ CD45RA+ CD8br | rs185430947 | A | G | -1.002 | 0.108 | 3.32e-20 | 0.023 | 85.71 |
| MFI | CD28 on CD28+ CD45RA+ CD8br | rs188216171 | G | A | -0.895 | 0.11 | 3.91e-16 | 0.018 | 66.8 |
| MFI | CD28 on CD28+ CD45RA+ CD8br | rs189799494 | G | T | -0.66 | 0.05 | 2.61e-38 | 0.045 | 171.32 |
| MFI | CD28 on CD28+ CD45RA+ CD8br | rs191363479 | C | T | -0.999 | 0.109 | 9.84e-20 | 0.022 | 83.48 |
| MFI | CD28 on CD28+ CD45RA+ CD8br | rs193039847 | G | A | -1.283 | 0.087 | 4.43e-48 | 0.056 | 218.31 |
| MFI | CD28 on CD28+ CD45RA+ CD8br | rs2229442 | A | G | -1.257 | 0.034 | 1.00e-200 | 0.274 | 1380.66 |
| MFI | CD28 on CD28+ CD45RA+ CD8br | rs4657041 | T | C | -0.148 | 0.027 | 4.18e-08 | 0.008 | 30.17 |
| MFI | CD28 on CD28+ CD45RA+ CD8br | rs532866318 | T | G | -1.314 | 0.12 | 1.25e-27 | 0.032 | 120.64 |
| MFI | CD28 on CD28+ CD45RA+ CD8br | rs535059510 | C | T | -0.958 | 0.117 | 4.99e-16 | 0.018 | 66.41 |
| MFI | CD28 on CD28+ CD45RA+ CD8br | rs539241708 | C | T | -1.113 | 0.085 | 3.62e-38 | 0.045 | 170.64 |
| MFI | CD28 on CD28+ CD45RA+ CD8br | rs556260593 | A | C | -1.004 | 0.106 | 3.11e-21 | 0.024 | 90.52 |
| MFI | CD28 on CD28+ CD45RA+ CD8br | rs557577522 | G | A | -0.746 | 0.071 | 1.24e-25 | 0.030 | 111.15 |
| MFI | CD28 on CD28+ CD45RA+ CD8br | rs568374507 | C | T | -0.575 | 0.059 | 1.86e-22 | 0.026 | 96.26 |
| MFI | CD28 on CD28+ CD45RA+ CD8br | rs78806227 | C | T | -0.931 | 0.12 | 1.16e-14 | 0.016 | 60.02 |
| MFI | CD28 on CD28+ CD45RA+ CD8br | rs79085286 | T | C | -0.398 | 0.053 | 1.02e-13 | 0.015 | 55.74 |
| MFI | CD28 on CD28+ CD45RA- CD8br | rs142607615 | C | T | -0.54 | 0.096 | 2.29e-08 | 0.011 | 31.38 |
| MFI | CD28 on CD28+ CD45RA- CD8br | rs2949661 | C | T | 0.567 | 0.026 | 3.45e-99 | 0.142 | 482.65 |
| MFI | CD28 on CD28+ CD45RA- CD8br | rs4657649 | T | C | 0.49 | 0.062 | 3.56e-15 | 0.021 | 62.57 |
| MFI | CD28 on CD28+ CD45RA- CD8br | rs57319220 | G | A | 0.202 | 0.037 | 3.70e-08 | 0.010 | 30.46 |
| MFI | CD28 on CD28+ CD45RA- CD8br | rs61814872 | C | T | 0.288 | 0.045 | 1.46e-10 | 0.014 | 41.37 |
| MFI | CD28 on CD39+ CD4+ | rs139795227 | A | C | -1.147 | 0.062 | 2.34e-72 | 0.107 | 342.56 |
| MFI | CD28 on CD39+ CD4+ | rs140205221 | A | G | -0.51 | 0.077 | 4.53e-11 | 0.015 | 43.67 |
| MFI | CD28 on CD39+ CD4+ | rs141264358 | T | C | -0.587 | 0.107 | 4.18e-08 | 0.011 | 30.22 |
| MFI | CD28 on CD39+ CD4+ | rs758800 | T | C | 0.662 | 0.045 | 5.12e-48 | 0.072 | 219.9 |
| MFI | CD28 on CD39+ CD4+ | rs76064946 | T | G | -0.35 | 0.042 | 2.61e-16 | 0.023 | 67.84 |
| MFI | CD28 on CD39+ CD4+ | rs79015439 | C | T | -0.791 | 0.121 | 6.94e-11 | 0.015 | 42.82 |
| MFI | CD28 on CD39+ activated Treg | rs11882720 | T | G | -0.44 | 0.072 | 1.16e-09 | 0.023 | 37.43 |
| MFI | CD28 on CD39+ activated Treg | rs3865444 | C | A | -1.214 | 0.037 | 7.84e-184 | 0.412 | 1101.82 |
| MFI | CD28 on CD39+ activated Treg | rs558625031 | G | A | -0.713 | 0.085 | 1.04e-16 | 0.043 | 70.35 |
| MFI | CD28 on CD39+ activated Treg | rs7351079 | A | G | -0.326 | 0.042 | 2.43e-14 | 0.036 | 59.21 |
| MFI | CD28 on CD39+ activated Treg | rs78949787 | C | T | 0.288 | 0.037 | 1.51e-14 | 0.037 | 60.15 |
| MFI | CD28 on CD45RA+ CD4+ | rs3793662 | C | T | -0.181 | 0.029 | 4.38e-10 | 0.011 | 39.12 |
| MFI | CD28 on CD45RA+ CD4+ | rs62626323 | C | A | 0.349 | 0.04 | 3.54e-18 | 0.020 | 76.31 |
| MFI | CD28 on CD45RA+ CD4+ | rs709589 | C | T | -0.249 | 0.031 | 1.19e-15 | 0.017 | 64.65 |
| MFI | CD28 on activated & secreting Treg | rs181878688 | G | A | -0.534 | 0.091 | 4.91e-09 | 0.018 | 34.52 |
| MFI | CD28 on activated & secreting Treg | rs548316 | G | A | -0.199 | 0.034 | 6.38e-09 | 0.018 | 34.02 |
| MFI | CD28 on activated & secreting Treg | rs574746346 | G | A | -0.753 | 0.12 | 4.83e-10 | 0.021 | 39.14 |
| MFI | CD28 on activated Treg | rs146313501 | C | T | -0.304 | 0.051 | 1.91e-09 | 0.012 | 36.27 |
| MFI | CD28 on activated Treg | rs35594332 | G | A | -0.494 | 0.042 | 1.62e-31 | 0.046 | 139.56 |
| MFI | CD28 on activated Treg | rs56262516 | G | A | 0.253 | 0.035 | 6.75e-13 | 0.018 | 52.02 |
| MFI | CD28 on activated Treg | rs9263601 | T | C | 0.2 | 0.031 | 8.61e-11 | 0.014 | 42.39 |
| MFI | CD28 on resting Treg | rs112266065 | C | T | -0.88 | 0.116 | 3.42e-14 | 0.016 | 57.89 |
| MFI | CD28 on resting Treg | rs114919524 | A | C | -1.14 | 0.107 | 2.81e-26 | 0.030 | 114.09 |
| MFI | CD28 on resting Treg | rs117630508 | G | A | -0.826 | 0.068 | 2.38e-33 | 0.039 | 147.64 |
| MFI | CD28 on resting Treg | rs117705111 | G | A | -0.763 | 0.065 | 2.97e-31 | 0.036 | 137.72 |
| MFI | CD28 on resting Treg | rs139592873 | G | T | -0.474 | 0.083 | 1.01e-08 | 0.009 | 32.96 |
| MFI | CD28 on resting Treg | rs141059303 | G | A | -0.66 | 0.117 | 1.87e-08 | 0.009 | 31.74 |
| MFI | CD28 on resting Treg | rs144365928 | A | G | -0.727 | 0.078 | 1.15e-20 | 0.023 | 87.88 |
| MFI | CD28 on resting Treg | rs148516965 | C | T | -0.578 | 0.056 | 1.16e-24 | 0.028 | 106.58 |
| MFI | CD28 on resting Treg | rs149167072 | A | G | 0.308 | 0.053 | 8.65e-09 | 0.009 | 33.25 |
| MFI | CD28 on resting Treg | rs1801274 | A | G | -0.18 | 0.027 | 2.29e-11 | 0.012 | 44.97 |
| MFI | CD28 on resting Treg | rs181079441 | C | T | -0.359 | 0.045 | 1.53e-15 | 0.017 | 64.11 |
| MFI | CD28 on resting Treg | rs184205740 | C | T | -1.21 | 0.116 | 4.48e-25 | 0.029 | 108.37 |
| MFI | CD28 on resting Treg | rs185430947 | A | G | -1.028 | 0.108 | 3.71e-21 | 0.024 | 90.05 |
| MFI | CD28 on resting Treg | rs191363479 | C | T | -1.031 | 0.109 | 6.78e-21 | 0.024 | 88.93 |
| MFI | CD28 on resting Treg | rs193039847 | G | A | -1.389 | 0.086 | 3.70e-56 | 0.066 | 257.89 |
| MFI | CD28 on resting Treg | rs539130256 | T | C | -0.616 | 0.105 | 5.47e-09 | 0.009 | 34.16 |
| MFI | CD28 on resting Treg | rs539241708 | C | T | -1.213 | 0.085 | 4.79e-45 | 0.053 | 203.92 |
| MFI | CD28 on resting Treg | rs556260593 | A | C | -1.046 | 0.106 | 7.71e-23 | 0.026 | 98.06 |
| MFI | CD28 on resting Treg | rs557577522 | G | A | -0.777 | 0.071 | 1.24e-27 | 0.032 | 120.55 |
| MFI | CD28 on resting Treg | rs558720004 | A | C | -1.269 | 0.109 | 1.42e-30 | 0.035 | 134.48 |
| MFI | CD28 on resting Treg | rs5758962 | A | C | -0.395 | 0.044 | 6.05e-19 | 0.021 | 79.9 |
| MFI | CD28 on resting Treg | rs62232129 | G | A | -0.305 | 0.053 | 7.41e-09 | 0.009 | 33.56 |
| MFI | CD28 on resting Treg | rs73165126 | C | T | -1.305 | 0.033 | 1.00e-200 | 0.298 | 1552.62 |
| MFI | CD28 on resting Treg | rs73888140 | G | A | -0.49 | 0.06 | 4.72e-16 | 0.018 | 66.47 |
| MFI | CD3 on CD28+ CD4+ | rs11615628 | G | A | -0.303 | 0.031 | 1.12e-21 | 0.031 | 92.88 |
| MFI | CD3 on CD28+ CD4+ | rs12298133 | C | T | 0.205 | 0.036 | 1.78e-08 | 0.011 | 31.87 |
| MFI | CD3 on CD28+ CD4+ | rs2744514 | G | A | -0.193 | 0.035 | 3.20e-08 | 0.010 | 30.74 |
| MFI | CD3 on CD28+ CD4+ | rs28584364 | C | T | 0.333 | 0.053 | 2.77e-10 | 0.014 | 40.09 |
| MFI | CD3 on CD28+ CD45RA+ CD8br | rs11074934 | C | T | -0.359 | 0.042 | 1.36e-17 | 0.044 | 74.45 |
| MFI | CD3 on CD28+ CD45RA+ CD8br | rs6808893 | C | T | -0.225 | 0.036 | 6.70e-10 | 0.023 | 38.52 |
| MFI | CD3 on CD28+ CD45RA+ CD8br | rs76918333 | G | A | 0.391 | 0.071 | 4.47e-08 | 0.018 | 30.18 |
| MFI | CD3 on CD28+ CD45RA+ CD8br | rs9269118 | G | A | 0.398 | 0.041 | 5.02e-22 | 0.055 | 95.7 |
| MFI | CD3 on CD28+ CD45RA+ CD8br | rs9270588 | C | T | -0.418 | 0.041 | 3.39e-24 | 0.061 | 106.18 |
| MFI | CD3 on CD28+ CD45RA+ CD8br | rs9469257 | G | A | -0.294 | 0.04 | 2.26e-13 | 0.032 | 54.64 |
| MFI | CD3 on CD28+ CD45RA- CD8br | rs113013837 | A | G | 0.743 | 0.104 | 1.09e-12 | 0.014 | 50.96 |
| MFI | CD3 on CD28+ CD45RA- CD8br | rs28739016 | T | G | 0.837 | 0.103 | 6.87e-16 | 0.018 | 65.73 |
| MFI | CD3 on CD28+ CD45RA- CD8br | rs55712453 | A | G | 0.558 | 0.091 | 8.33e-10 | 0.010 | 37.86 |
| MFI | CD3 on CD28+ DN (CD4-CD8-) | rs112505169 | C | T | -0.819 | 0.101 | 7.55e-16 | 0.019 | 65.55 |
| MFI | CD3 on CD28+ DN (CD4-CD8-) | rs11589644 | C | T | -0.732 | 0.105 | 3.23e-12 | 0.014 | 48.84 |
| MFI | CD3 on CD28+ DN (CD4-CD8-) | rs140308479 | T | G | -0.536 | 0.098 | 4.64e-08 | 0.009 | 29.98 |
| MFI | CD3 on CD28+ DN (CD4-CD8-) | rs141272483 | C | T | 0.39 | 0.07 | 3.07e-08 | 0.009 | 30.78 |
| MFI | CD3 on CD28+ DN (CD4-CD8-) | rs142500247 | T | C | -0.699 | 0.07 | 6.49e-23 | 0.028 | 98.47 |
| MFI | CD3 on CD28+ DN (CD4-CD8-) | rs17607399 | C | T | -0.208 | 0.037 | 1.70e-08 | 0.009 | 31.96 |
| MFI | CD3 on CD28+ DN (CD4-CD8-) | rs413431 | A | G | 0.275 | 0.029 | 1.16e-20 | 0.025 | 87.91 |
| MFI | CD3 on CD28+ DN (CD4-CD8-) | rs6751481 | T | C | -0.29 | 0.022 | 6.29e-38 | 0.047 | 169.82 |
| MFI | CD3 on CD28- CD8br | rs116007826 | A | G | 0.834 | 0.06 | 6.52e-42 | 0.062 | 189.9 |
| MFI | CD3 on CD28- CD8br | rs34039593 | T | G | 1.075 | 0.046 | 3.67e-110 | 0.159 | 542.68 |
| MFI | CD3 on CD28- CD8br | rs362522 | C | T | 0.322 | 0.041 | 3.42e-15 | 0.021 | 62.65 |
| MFI | CD3 on CD28- CD8br | rs55971447 | C | T | -0.342 | 0.032 | 2.91e-26 | 0.038 | 114.58 |
| MFI | CD3 on CD28- CD8br | rs7748976 | G | A | 0.266 | 0.041 | 1.44e-10 | 0.014 | 41.36 |
| MFI | CD3 on CD28- CD8br | rs9296042 | C | T | -0.319 | 0.03 | 3.03e-25 | 0.037 | 109.74 |
| MFI | CD3 on CD28- CD8br | rs9468543 | G | A | 0.636 | 0.083 | 3.01e-14 | 0.020 | 58.27 |
| MFI | CD3 on CD39+ resting Treg | rs111695695 | C | A | -0.513 | 0.068 | 1.06e-13 | 0.035 | 56.2 |
| MFI | CD3 on CD39+ resting Treg | rs143334311 | A | G | 0.937 | 0.157 | 3.10e-09 | 0.022 | 35.51 |
| MFI | CD3 on CD39+ resting Treg | rs191091405 | C | T | 0.543 | 0.066 | 5.01e-16 | 0.041 | 67.13 |
| MFI | CD3 on CD39+ resting Treg | rs7245846 | G | A | -1.115 | 0.039 | 6.08e-143 | 0.341 | 803.91 |
| MFI | CD3 on CD39+ resting Treg | rs7351079 | A | G | -0.335 | 0.042 | 5.07e-15 | 0.039 | 62.38 |
| MFI | CD3 on CD39+ resting Treg | rs75773078 | G | A | -0.525 | 0.071 | 1.64e-13 | 0.034 | 55.32 |
| MFI | CD3 on CD39+ resting Treg | rs79377141 | G | A | 0.338 | 0.046 | 3.07e-13 | 0.034 | 54.03 |
| MFI | CD3 on CD4+ | rs117067704 | A | G | 0.641 | 0.089 | 7.97e-13 | 0.018 | 51.72 |
| MFI | CD3 on CD4+ | rs144843975 | C | T | 0.368 | 0.062 | 3.33e-09 | 0.012 | 35.17 |
| MFI | CD3 on CD4+ | rs62045817 | C | T | -0.214 | 0.037 | 5.27e-09 | 0.012 | 34.26 |
| MFI | CD3 on CD4+ | rs722926 | C | T | 0.152 | 0.028 | 3.66e-08 | 0.011 | 30.46 |
| MFI | CD3 on CM CD8br | rs12921174 | T | C | -0.21 | 0.034 | 4.82e-10 | 0.013 | 38.98 |
| MFI | CD3 on CM CD8br | rs142607615 | C | T | -0.618 | 0.095 | 1.02e-10 | 0.014 | 42.06 |
| MFI | CD3 on CM CD8br | rs28607988 | A | C | 0.233 | 0.036 | 1.46e-10 | 0.014 | 41.34 |
| MFI | CD3 on CM CD8br | rs2949661 | C | T | 0.686 | 0.024 | 5.66e-154 | 0.214 | 789.52 |
| MFI | CD3 on CM CD8br | rs4657649 | T | C | 0.549 | 0.061 | 3.34e-19 | 0.027 | 81.29 |
| MFI | CD3 on CM CD8br | rs61814872 | C | T | 0.335 | 0.044 | 4.48e-14 | 0.019 | 57.49 |
| MFI | CD3 on EM CD4+ | rs11773763 | C | T | -0.186 | 0.03 | 7.29e-10 | 0.013 | 38.17 |
| MFI | CD3 on EM CD4+ | rs343817 | G | T | -0.208 | 0.032 | 6.96e-11 | 0.015 | 42.84 |
| MFI | CD3 on EM CD4+ | rs3917761 | G | T | -0.166 | 0.028 | 4.48e-09 | 0.012 | 34.59 |
| MFI | CD3 on EM CD4+ | rs4987358 | G | T | 0.326 | 0.029 | 4.42e-28 | 0.041 | 123.21 |
| MFI | CD3 on HLA DR+ CD4+ | rs10882701 | A | C | -0.448 | 0.024 | 2.50e-73 | 0.091 | 344.13 |
| MFI | CD3 on HLA DR+ CD4+ | rs11188449 | G | A | -1.091 | 0.017 | 1.00e-200 | 0.535 | 3956.38 |
| MFI | CD3 on HLA DR+ CD4+ | rs118087712 | G | A | 0.288 | 0.039 | 3.90e-13 | 0.015 | 53.07 |
| MFI | CD3 on HLA DR+ CD4+ | rs141018090 | G | T | 0.64 | 0.054 | 1.49e-31 | 0.039 | 139.31 |
| MFI | CD3 on HLA DR+ CD4+ | rs148430006 | G | T | 0.498 | 0.087 | 1.38e-08 | 0.009 | 32.35 |
| MFI | CD3 on HLA DR+ CD4+ | rs181677252 | C | T | 0.386 | 0.07 | 3.35e-08 | 0.009 | 30.63 |
| MFI | CD3 on HLA DR+ CD4+ | rs185198079 | C | T | 0.966 | 0.093 | 4.43e-25 | 0.031 | 108.64 |
| MFI | CD3 on HLA DR+ CD4+ | rs191819784 | G | A | 1.008 | 0.074 | 4.01e-41 | 0.051 | 185.09 |
| MFI | CD3 on HLA DR+ CD4+ | rs61868672 | G | A | -0.438 | 0.05 | 2.48e-18 | 0.022 | 77.11 |
| MFI | CD3 on HLA DR+ CD4+ | rs7906684 | G | A | 1.337 | 0.077 | 4.54e-64 | 0.080 | 297.68 |
| MFI | CD3 on NKT | rs111780443 | C | T | -0.366 | 0.066 | 3.25e-08 | 0.010 | 30.7 |
| MFI | CD3 on NKT | rs12921174 | T | C | -0.194 | 0.034 | 1.40e-08 | 0.011 | 32.36 |
| MFI | CD3 on NKT | rs2995089 | G | A | 0.619 | 0.025 | 6.62e-120 | 0.170 | 595.34 |
| MFI | CD3 on NKT | rs4657649 | T | C | 0.482 | 0.062 | 9.11e-15 | 0.020 | 60.69 |
| MFI | CD3 on NKT | rs4657706 | C | T | 0.192 | 0.033 | 8.64e-09 | 0.011 | 33.32 |
| MFI | CD3 on NKT | rs61814886 | G | A | 0.318 | 0.044 | 8.58e-13 | 0.017 | 51.58 |
| MFI | CD3 on NKT | rs78132817 | T | G | -0.499 | 0.081 | 1.00e-09 | 0.013 | 37.54 |
| MFI | CD3 on secreting Treg | rs11961777 | T | C | -0.47 | 0.072 | 9.55e-11 | 0.022 | 42.36 |
| MFI | CD3 on secreting Treg | rs1354106 | T | G | -0.431 | 0.038 | 1.72e-28 | 0.064 | 126.6 |
| MFI | CD3 on secreting Treg | rs2071473 | C | T | -0.24 | 0.038 | 2.30e-10 | 0.021 | 40.61 |
| MFI | CD3 on secreting Treg | rs9270591 | C | T | 0.416 | 0.039 | 5.46e-26 | 0.058 | 114.47 |
| MFI | CD33 on CD14+ monocyte | rs111902980 | G | A | 0.473 | 0.086 | 4.72e-08 | 0.009 | 29.95 |
| MFI | CD33 on CD14+ monocyte | rs139249541 | A | G | 0.398 | 0.071 | 1.85e-08 | 0.009 | 31.79 |
| MFI | CD33 on CD14+ monocyte | rs144837535 | T | C | 0.413 | 0.069 | 2.27e-09 | 0.010 | 35.9 |
| MFI | CD33 on CD14+ monocyte | rs181878688 | G | A | 0.448 | 0.066 | 1.46e-11 | 0.013 | 45.85 |
| MFI | CD33 on CD14+ monocyte | rs186978368 | C | T | 0.388 | 0.07 | 3.84e-08 | 0.009 | 30.34 |
| MFI | CD33 on CD14+ monocyte | rs6710361 | G | A | 0.177 | 0.03 | 5.45e-09 | 0.010 | 34.17 |
| MFI | CD33 on CD14+ monocyte | rs9896155 | A | G | 0.152 | 0.026 | 2.84e-09 | 0.010 | 35.44 |
| MFI | CD33 on CD33br HLA DR+ | rs115901521 | G | A | -0.27 | 0.039 | 6.78e-12 | 0.013 | 47.38 |
| MFI | CD33 on CD33br HLA DR+ | rs56199187 | T | C | 0.848 | 0.026 | 1.00e-200 | 0.228 | 1070.39 |
| MFI | CD33 on CD33br HLA DR+ | rs74341264 | A | G | -0.342 | 0.038 | 2.74e-19 | 0.022 | 81.49 |
| MFI | CD33 on CD33br HLA DR+ CD14- | rs1801274 | A | G | 0.465 | 0.026 | 2.06e-70 | 0.083 | 328.67 |
| MFI | CD33 on CD33br HLA DR+ CD14- | rs183974458 | C | T | 0.46 | 0.066 | 3.09e-12 | 0.013 | 48.94 |
| MFI | CD33 on CD33br HLA DR+ CD14- | rs2664523 | G | A | 0.36 | 0.063 | 1.03e-08 | 0.009 | 32.91 |
| MFI | CD33 on CD33br HLA DR+ CD14- | rs6687275 | T | G | 0.198 | 0.028 | 3.17e-12 | 0.013 | 48.89 |
| MFI | CD33 on CD33br HLA DR+ CD14- | rs745307 | G | A | 0.698 | 0.033 | 3.78e-92 | 0.108 | 439.13 |
| MFI | CD33 on CD33dim HLA DR+ CD11b- | rs115805162 | C | T | -0.226 | 0.032 | 2.75e-12 | 0.014 | 49.17 |
| MFI | CD33 on CD33dim HLA DR+ CD11b- | rs569911 | T | C | 0.153 | 0.026 | 5.43e-09 | 0.010 | 34.19 |
| MFI | CD33 on CD33dim HLA DR+ CD11b- | rs71632979 | A | G | 0.655 | 0.028 | 2.29e-112 | 0.139 | 547.41 |
| MFI | CD33 on Im MDSC | rs113346428 | G | A | 0.356 | 0.055 | 7.15e-11 | 0.012 | 42.69 |
| MFI | CD33 on Im MDSC | rs11544989 | A | G | 0.141 | 0.025 | 2.55e-08 | 0.008 | 31.13 |
| MFI | CD33 on Im MDSC | rs115536607 | C | T | 0.386 | 0.054 | 1.35e-12 | 0.014 | 50.58 |
| MFI | CD33 on Im MDSC | rs117147408 | A | G | 0.708 | 0.076 | 1.51e-20 | 0.023 | 87.33 |
| MFI | CD33 on Im MDSC | rs144837535 | T | C | 0.706 | 0.07 | 1.75e-23 | 0.027 | 101.06 |
| MFI | CD33 on Im MDSC | rs150386792 | G | A | 0.73 | 0.074 | 7.30e-23 | 0.026 | 98.16 |
| MFI | CD33 on Im MDSC | rs181878688 | G | A | 0.898 | 0.067 | 2.33e-40 | 0.047 | 181.15 |
| MFI | CD33 on Im MDSC | rs182663933 | T | C | 0.397 | 0.057 | 3.11e-12 | 0.013 | 48.91 |
| MFI | CD33 on Im MDSC | rs186978368 | C | T | 0.674 | 0.072 | 1.20e-20 | 0.023 | 87.81 |
| MFI | CD33 on Im MDSC | rs57245661 | A | C | 0.619 | 0.093 | 2.61e-11 | 0.012 | 44.7 |
| MFI | CD33 on Im MDSC | rs62432266 | G | T | 0.455 | 0.052 | 5.24e-18 | 0.020 | 75.52 |
| MFI | CD33 on Im MDSC | rs77738700 | G | A | -0.32 | 0.039 | 5.38e-16 | 0.018 | 66.24 |
| MFI | CD33 on Im MDSC | rs9916257 | G | T | 0.144 | 0.025 | 5.79e-09 | 0.009 | 34.02 |
| MFI | CD33 on Mo MDSC | rs10014683 | T | C | -3.777 | 0.668 | 1.72e-08 | 0.009 | 31.92 |
| MFI | CD33 on Mo MDSC | rs10930181 | T | C | -3.813 | 0.673 | 1.59e-08 | 0.009 | 32.08 |
| MFI | CD33 on Mo MDSC | rs113760892 | T | C | -3.783 | 0.668 | 1.63e-08 | 0.009 | 32.02 |
| MFI | CD33 on Mo MDSC | rs114971917 | T | C | -3.783 | 0.668 | 1.63e-08 | 0.009 | 32.02 |
| MFI | CD33 on Mo MDSC | rs116562707 | A | G | 0.402 | 0.068 | 3.97e-09 | 0.010 | 34.8 |
| MFI | CD33 on Mo MDSC | rs138636327 | T | C | -3.783 | 0.668 | 1.63e-08 | 0.009 | 32.02 |
| MFI | CD33 on Mo MDSC | rs140439680 | T | G | -3.783 | 0.668 | 1.63e-08 | 0.009 | 32.02 |
| MFI | CD33 on Mo MDSC | rs141862602 | A | G | -3.783 | 0.668 | 1.63e-08 | 0.009 | 32.02 |
| MFI | CD33 on Mo MDSC | rs143239155 | A | G | -3.783 | 0.668 | 1.63e-08 | 0.009 | 32.02 |
| MFI | CD33 on Mo MDSC | rs148464573 | A | G | -3.783 | 0.668 | 1.63e-08 | 0.009 | 32.02 |
| MFI | CD33 on Mo MDSC | rs149530842 | T | C | -3.783 | 0.668 | 1.63e-08 | 0.009 | 32.02 |
| MFI | CD33 on Mo MDSC | rs149628462 | G | A | -3.783 | 0.668 | 1.63e-08 | 0.009 | 32.02 |
| MFI | CD33 on Mo MDSC | rs180762782 | T | C | -3.783 | 0.668 | 1.63e-08 | 0.009 | 32.02 |
| MFI | CD33 on Mo MDSC | rs180923845 | A | G | -3.783 | 0.668 | 1.63e-08 | 0.009 | 32.02 |
| MFI | CD33 on Mo MDSC | rs185708993 | G | A | -3.783 | 0.668 | 1.63e-08 | 0.009 | 32.02 |
| MFI | CD33 on Mo MDSC | rs190639387 | T | G | -3.783 | 0.668 | 1.63e-08 | 0.009 | 32.02 |
| MFI | CD33 on Mo MDSC | rs193299790 | T | C | -3.783 | 0.668 | 1.63e-08 | 0.009 | 32.02 |
| MFI | CD33 on Mo MDSC | rs199934765 | A | G | -3.783 | 0.668 | 1.63e-08 | 0.009 | 32.02 |
| MFI | CD33 on Mo MDSC | rs201357007 | A | G | -3.783 | 0.668 | 1.63e-08 | 0.009 | 32.02 |
| MFI | CD33 on Mo MDSC | rs2400862 | A | G | -3.643 | 0.666 | 4.80e-08 | 0.009 | 29.91 |
| MFI | CD33 on Mo MDSC | rs34457782 | G | A | -0.25 | 0.045 | 3.48e-08 | 0.009 | 30.56 |
| MFI | CD33 on Mo MDSC | rs34873616 | C | T | 0.103 | 0.018 | 4.74e-09 | 0.010 | 34.44 |
| MFI | CD33 on Mo MDSC | rs349478 | A | G | -6.841 | 1.208 | 1.63e-08 | 0.009 | 32.05 |
| MFI | CD33 on Mo MDSC | rs61090010 | A | G | -3.783 | 0.668 | 1.63e-08 | 0.009 | 32.02 |
| MFI | CD33 on Mo MDSC | rs6467354 | C | T | -4.08 | 0.722 | 1.70e-08 | 0.009 | 31.95 |
| MFI | CD33 on Mo MDSC | rs6483391 | A | G | -3.86 | 0.652 | 3.53e-09 | 0.010 | 35.03 |
| MFI | CD33 on Mo MDSC | rs6748248 | G | T | -3.8 | 0.672 | 1.66e-08 | 0.009 | 32 |
| MFI | CD33 on Mo MDSC | rs7496070 | G | A | -3.764 | 0.668 | 1.90e-08 | 0.009 | 31.72 |
| MFI | CD33 on Mo MDSC | rs76969561 | C | T | -3.783 | 0.668 | 1.63e-08 | 0.009 | 32.02 |
| MFI | CD33 on Mo MDSC | rs943885 | A | C | -3.787 | 0.668 | 1.57e-08 | 0.009 | 32.09 |
| MFI | CD33 on Mo MDSC | rs9611143 | A | G | -3.783 | 0.668 | 1.63e-08 | 0.009 | 32.02 |
| MFI | CD34 on HSC | rs11615628 | G | A | -0.326 | 0.031 | 6.46e-25 | 0.036 | 108.19 |
| MFI | CD34 on HSC | rs2855450 | C | T | -0.209 | 0.034 | 1.04e-09 | 0.013 | 37.46 |
| MFI | CD34 on HSC | rs28584364 | C | T | 0.353 | 0.052 | 2.08e-11 | 0.015 | 45.21 |
| MFI | CD38 on CD3- CD19- | rs11051398 | C | T | -0.151 | 0.026 | 7.67e-09 | 0.009 | 33.49 |
| MFI | CD38 on CD3- CD19- | rs140684757 | C | T | 0.515 | 0.074 | 3.35e-12 | 0.013 | 48.77 |
| MFI | CD38 on CD3- CD19- | rs147487472 | C | A | 0.446 | 0.062 | 5.60e-13 | 0.014 | 52.34 |
| MFI | CD38 on CD3- CD19- | rs3793662 | C | T | -0.195 | 0.03 | 4.56e-11 | 0.012 | 43.63 |
| MFI | CD38 on CD3- CD19- | rs41294937 | T | C | 0.563 | 0.04 | 2.21e-43 | 0.051 | 195.66 |
| MFI | CD38 on IgD+ CD38dim | rs115383270 | G | A | -0.302 | 0.034 | 3.69e-19 | 0.022 | 80.9 |
| MFI | CD38 on IgD+ CD38dim | rs62395272 | C | T | -0.262 | 0.033 | 3.77e-15 | 0.017 | 62.35 |
| MFI | CD38 on IgD+ CD38dim | rs9275416 | C | T | 0.552 | 0.034 | 1.18e-57 | 0.068 | 265.39 |
| MFI | CD38 on IgD+ CD38dim | rs9276316 | A | G | -0.306 | 0.028 | 1.54e-26 | 0.031 | 115.44 |
| MFI | CD38 on IgD- CD38br | rs12752576 | A | G | -0.728 | 0.11 | 4.57e-11 | 0.015 | 43.68 |
| MFI | CD38 on IgD- CD38br | rs2995091 | T | C | 0.652 | 0.025 | 1.87e-136 | 0.191 | 688.56 |
| MFI | CD38 on IgD- CD38br | rs61814886 | G | A | 0.362 | 0.044 | 2.58e-16 | 0.023 | 67.85 |
| MFI | CD38 on IgD- CD38br | rs6668517 | A | C | -0.231 | 0.041 | 2.66e-08 | 0.011 | 31.08 |
| MFI | CD38 on IgD- CD38br | rs72703432 | A | G | -0.503 | 0.086 | 5.23e-09 | 0.012 | 34.28 |
| MFI | CD38 on IgD- CD38br | rs78132817 | T | G | -0.512 | 0.081 | 2.63e-10 | 0.014 | 40.19 |
| MFI | CD38 on IgD- CD38dim | rs113598618 | C | T | 0.466 | 0.069 | 2.72e-11 | 0.027 | 44.94 |
| MFI | CD38 on IgD- CD38dim | rs11882720 | T | G | -0.423 | 0.068 | 5.92e-10 | 0.023 | 38.76 |
| MFI | CD38 on IgD- CD38dim | rs1801274 | A | G | -0.261 | 0.039 | 1.84e-11 | 0.027 | 45.74 |
| MFI | CD38 on IgD- CD38dim | rs2459145 | T | C | 1.329 | 0.207 | 1.75e-10 | 0.025 | 41.25 |
| MFI | CD38 on IgD- CD38dim | rs3865444 | C | A | -1.119 | 0.034 | 2.40e-183 | 0.400 | 1086.97 |
| MFI | CD38 on IgD- CD38dim | rs558625031 | G | A | -0.696 | 0.081 | 1.96e-17 | 0.043 | 73.73 |
| MFI | CD38 on IgD- CD38dim | rs7351079 | A | G | -0.383 | 0.04 | 2.91e-21 | 0.053 | 92.03 |
| MFI | CD38 on IgD- CD38dim | rs78949787 | C | T | 0.298 | 0.036 | 1.33e-16 | 0.041 | 69.8 |
| MFI | CD38 on naive-mature B cell | rs11551421 | G | A | 0.34 | 0.044 | 7.41e-15 | 0.017 | 60.97 |
| MFI | CD38 on naive-mature B cell | rs180717063 | G | A | 0.72 | 0.094 | 1.98e-14 | 0.016 | 59 |
| MFI | CD38 on naive-mature B cell | rs3132462 | G | T | -0.366 | 0.041 | 8.68e-19 | 0.021 | 79.16 |
| MFI | CD38 on naive-mature B cell | rs35118762 | C | T | 0.812 | 0.041 | 1.54e-83 | 0.098 | 394.9 |
| MFI | CD38 on naive-mature B cell | rs474534 | A | G | -0.368 | 0.033 | 1.43e-28 | 0.033 | 125.01 |
| MFI | CD38 on naive-mature B cell | rs544612305 | G | A | -0.245 | 0.041 | 2.77e-09 | 0.010 | 35.5 |
| MFI | CD38 on naive-mature B cell | rs76886291 | G | A | 0.335 | 0.048 | 2.82e-12 | 0.013 | 49.13 |
| MFI | CD38 on naive-mature B cell | rs9501678 | C | T | 0.417 | 0.076 | 4.86e-08 | 0.008 | 29.89 |
| MFI | CD38 on transitional | rs150145799 | T | C | 0.288 | 0.036 | 3.92e-15 | 0.017 | 62.27 |
| MFI | CD38 on transitional | rs558721 | C | T | 0.333 | 0.059 | 1.70e-08 | 0.009 | 31.93 |
| MFI | CD38 on transitional | rs9597833 | T | G | -0.138 | 0.025 | 3.61e-08 | 0.008 | 30.47 |
| MFI | CD39 on CD39+ CD8br | rs114068468 | C | T | 0.367 | 0.066 | 2.51e-08 | 0.019 | 31.32 |
| MFI | CD39 on CD39+ CD8br | rs115799162 | T | C | 0.533 | 0.092 | 7.40e-09 | 0.020 | 33.74 |
| MFI | CD39 on CD39+ CD8br | rs180717063 | G | A | 0.847 | 0.129 | 7.89e-11 | 0.026 | 42.82 |
| MFI | CD39 on CD39+ CD8br | rs2760980 | G | A | 1.049 | 0.061 | 2.44e-60 | 0.152 | 291.81 |
| MFI | CD39 on CD39+ CD8br | rs61998168 | G | A | 0.593 | 0.105 | 1.73e-08 | 0.019 | 32.07 |
| MFI | CD39 on CD39+ CD8br | rs9268498 | T | C | -0.451 | 0.045 | 2.71e-23 | 0.059 | 101.81 |
| MFI | CD39 on CD39+ CD8br | rs9279789 | T | C | 0.711 | 0.112 | 2.87e-10 | 0.024 | 40.22 |
| MFI | CD39 on CD39+ activated Treg | rs140308479 | T | G | 0.556 | 0.097 | 1.03e-08 | 0.010 | 32.93 |
| MFI | CD39 on CD39+ activated Treg | rs61038907 | C | A | 0.14 | 0.025 | 2.72e-08 | 0.009 | 31.03 |
| MFI | CD39 on CD39+ activated Treg | rs6729180 | C | T | 0.184 | 0.023 | 1.20e-15 | 0.019 | 64.61 |
| MFI | CD39 on CD39+ activated Treg | rs77745909 | A | G | 0.654 | 0.103 | 2.80e-10 | 0.012 | 40.06 |
| MFI | CD39 on CD39+ secreting Treg | rs11731825 | A | G | -0.198 | 0.032 | 7.26e-10 | 0.010 | 38.13 |
| MFI | CD39 on CD39+ secreting Treg | rs145567900 | C | A | -1.013 | 0.118 | 1.16e-17 | 0.020 | 73.91 |
| MFI | CD39 on CD39+ secreting Treg | rs197429 | C | T | 0.167 | 0.029 | 1.35e-08 | 0.009 | 32.41 |
| MFI | CD39 on CD39+ secreting Treg | rs548982606 | C | T | -0.735 | 0.088 | 9.27e-17 | 0.019 | 69.75 |
| MFI | CD39 on CD39+ secreting Treg | rs572542 | C | T | -0.451 | 0.072 | 5.24e-10 | 0.010 | 38.77 |
| MFI | CD39 on CD39+ secreting Treg | rs574746346 | G | A | -1.273 | 0.081 | 6.52e-54 | 0.063 | 246.98 |
| MFI | CD39 on granulocyte | rs1403755 | C | T | 0.27 | 0.047 | 8.82e-09 | 0.011 | 33.25 |
| MFI | CD39 on granulocyte | rs2875404 | C | T | -0.154 | 0.028 | 3.16e-08 | 0.010 | 30.72 |
| MFI | CD39 on granulocyte | rs72915529 | G | A | -0.701 | 0.128 | 4.27e-08 | 0.010 | 30.14 |
| MFI | CD4 on CD28+ CD4+ | rs1007337 | C | T | -0.139 | 0.025 | 3.33e-08 | 0.008 | 30.63 |
| MFI | CD4 on CD28+ CD4+ | rs144398648 | C | T | 0.825 | 0.119 | 4.65e-12 | 0.013 | 48.08 |
| MFI | CD4 on CD28+ CD4+ | rs2066399 | G | A | 0.238 | 0.032 | 1.75e-13 | 0.015 | 54.61 |
| MFI | CD4 on CD28+ CD4+ | rs4939384 | A | G | -0.236 | 0.025 | 2.50e-21 | 0.024 | 90.95 |
| MFI | CD4 on CD28+ CD4+ | rs62405562 | A | G | 0.228 | 0.029 | 1.82e-15 | 0.017 | 63.75 |
| MFI | CD4 on CD28+ CD4+ | rs6509428 | C | T | -0.18 | 0.029 | 7.95e-10 | 0.010 | 37.97 |
| MFI | CD4 on CD28+ CD4+ | rs72836542 | C | T | 0.175 | 0.027 | 7.20e-11 | 0.012 | 42.71 |
| MFI | CD4 on CD28+ CD4+ | rs7404890 | G | A | 0.179 | 0.033 | 4.23e-08 | 0.008 | 30.14 |
| MFI | CD4 on CD28+ CD4+ | rs9270560 | T | C | -0.218 | 0.03 | 2.13e-13 | 0.015 | 54.25 |
| MFI | CD4 on CD39+ secreting Treg | rs182004736 | A | G | -0.245 | 0.042 | 6.46e-09 | 0.010 | 33.85 |
| MFI | CD4 on CD39+ secreting Treg | rs189561833 | A | G | 0.5 | 0.086 | 8.35e-09 | 0.010 | 33.34 |
| MFI | CD4 on CD39+ secreting Treg | rs61839660 | C | T | 0.611 | 0.055 | 6.70e-28 | 0.034 | 121.95 |
| MFI | CD4 on CD4 Treg | rs7245846 | G | A | -0.836 | 0.049 | 1.02e-58 | 0.189 | 290.12 |
| MFI | CD4 on CD4 Treg | rs7351079 | A | G | -0.282 | 0.048 | 5.38e-09 | 0.027 | 34.48 |
| MFI | CD4 on CD4 Treg | rs78949787 | C | T | 0.228 | 0.041 | 3.22e-08 | 0.024 | 30.93 |
| MFI | CD4 on CD4+ | rs13154973 | A | C | -0.292 | 0.043 | 8.14e-12 | 0.016 | 47.07 |
| MFI | CD4 on CD4+ | rs1573735 | C | T | -0.207 | 0.028 | 4.02e-13 | 0.018 | 53.08 |
| MFI | CD4 on CD4+ | rs3129891 | G | A | -0.237 | 0.034 | 5.88e-12 | 0.016 | 47.72 |
| MFI | CD4 on CD4+ | rs9271772 | G | A | 0.261 | 0.035 | 2.23e-13 | 0.018 | 54.24 |
| MFI | CD4 on CD45RA+ CD4+ | rs56262516 | G | A | 0.23 | 0.034 | 1.23e-11 | 0.016 | 46.25 |
| MFI | CD4 on CD45RA+ CD4+ | rs7425430 | A | G | -0.219 | 0.032 | 1.18e-11 | 0.016 | 46.34 |
| MFI | CD4 on CD45RA+ CD4+ | rs9263601 | T | C | 0.176 | 0.03 | 4.31e-09 | 0.012 | 34.65 |
| MFI | CD4 on activated Treg | rs10748643 | A | G | 1 | 0.019 | 1.00e-200 | 0.435 | 2650.1 |
| MFI | CD4 on activated Treg | rs111534725 | G | T | 0.767 | 0.065 | 7.27e-32 | 0.039 | 140.77 |
| MFI | CD4 on activated Treg | rs12778618 | T | C | 0.819 | 0.061 | 5.07e-40 | 0.050 | 179.81 |
| MFI | CD4 on activated Treg | rs149309642 | G | A | -0.625 | 0.1 | 4.83e-10 | 0.011 | 38.93 |
| MFI | CD4 on activated Treg | rs17111341 | T | C | 0.64 | 0.052 | 2.59e-34 | 0.042 | 152.42 |
| MFI | CD4 on activated Treg | rs185198079 | C | T | 0.769 | 0.095 | 6.90e-16 | 0.019 | 65.75 |
| MFI | CD4 on activated Treg | rs72812685 | G | A | 0.624 | 0.059 | 7.75e-26 | 0.032 | 112.21 |
| MFI | CD4 on activated Treg | rs76736637 | G | A | 0.539 | 0.086 | 4.91e-10 | 0.011 | 38.91 |
| MFI | CD4 on activated Treg | rs7906684 | G | A | 1.079 | 0.08 | 1.89e-40 | 0.050 | 181.9 |
| MFI | CD4 on resting Treg | rs114066913 | C | A | 0.657 | 0.077 | 2.98e-17 | 0.044 | 72.92 |
| MFI | CD4 on resting Treg | rs28772323 | A | C | -0.347 | 0.04 | 6.20e-18 | 0.046 | 76.16 |
| MFI | CD4 on resting Treg | rs9261798 | A | G | -0.293 | 0.047 | 6.59e-10 | 0.024 | 38.57 |
| MFI | CD4 on resting Treg | rs9270588 | C | T | -0.599 | 0.039 | 2.14e-49 | 0.129 | 233.95 |
| MFI | CD40 on CD14+ CD16+ monocyte | rs112505169 | C | T | -0.856 | 0.108 | 2.73e-15 | 0.018 | 62.97 |
| MFI | CD40 on CD14+ CD16+ monocyte | rs11589644 | C | T | -0.82 | 0.111 | 2.11e-13 | 0.016 | 54.31 |
| MFI | CD40 on CD14+ CD16+ monocyte | rs17607399 | C | T | -0.221 | 0.039 | 1.44e-08 | 0.009 | 32.28 |
| MFI | CD40 on CD14+ CD16+ monocyte | rs183949931 | T | C | -0.698 | 0.076 | 4.48e-20 | 0.024 | 85.19 |
| MFI | CD40 on CD14+ CD16+ monocyte | rs413431 | A | G | 0.296 | 0.031 | 3.31e-21 | 0.026 | 90.46 |
| MFI | CD40 on CD14+ CD16+ monocyte | rs6751481 | T | C | -0.288 | 0.024 | 3.53e-33 | 0.041 | 146.98 |
| MFI | CD45 on CD33- HLA DR- | rs10501383 | C | T | -0.299 | 0.041 | 3.61e-13 | 0.014 | 53.22 |
| MFI | CD45 on CD33- HLA DR- | rs11751198 | G | A | 0.218 | 0.037 | 4.52e-09 | 0.009 | 34.53 |
| MFI | CD45 on CD33- HLA DR- | rs144398648 | C | T | 0.903 | 0.119 | 4.80e-14 | 0.015 | 57.22 |
| MFI | CD45 on CD33- HLA DR- | rs1569590 | A | G | 0.207 | 0.037 | 2.88e-08 | 0.008 | 30.9 |
| MFI | CD45 on CD33- HLA DR- | rs35039517 | G | A | 0.189 | 0.032 | 2.00e-09 | 0.010 | 36.13 |
| MFI | CD45 on CD33- HLA DR- | rs55809481 | T | C | -0.322 | 0.029 | 9.31e-29 | 0.033 | 125.86 |
| MFI | CD45 on CD33- HLA DR- | rs9271146 | A | C | -0.173 | 0.031 | 1.77e-08 | 0.009 | 31.85 |
| MFI | CD45 on CD33br HLA DR+ | rs12874404 | A | G | 0.183 | 0.026 | 4.02e-12 | 0.013 | 48.42 |
| MFI | CD45 on CD33br HLA DR+ | rs139370814 | C | T | 0.569 | 0.087 | 5.94e-11 | 0.012 | 43.07 |
| MFI | CD45 on CD33br HLA DR+ CD14- | rs12874404 | A | G | -0.146 | 0.026 | 1.60e-08 | 0.009 | 32.03 |
| MFI | CD45 on CD33br HLA DR+ CD14- | rs183053322 | C | T | -0.416 | 0.066 | 3.35e-10 | 0.011 | 39.66 |
| MFI | CD45 on CD33br HLA DR+ CD14- | rs7218011 | A | G | 0.182 | 0.032 | 1.74e-08 | 0.009 | 31.9 |
| MFI | CD45 on CD33br HLA DR+ CD14- | rs9916257 | G | T | -0.191 | 0.024 | 1.21e-15 | 0.017 | 64.57 |
| MFI | CD45 on CD33dim HLA DR- | rs10882701 | A | C | -0.16 | 0.028 | 7.66e-09 | 0.011 | 33.51 |
| MFI | CD45 on CD33dim HLA DR- | rs117458836 | G | A | 0.693 | 0.104 | 3.45e-11 | 0.015 | 44.25 |
| MFI | CD45 on CD33dim HLA DR- | rs12778618 | T | C | 0.52 | 0.066 | 4.44e-15 | 0.021 | 62.11 |
| MFI | CD45 on CD33dim HLA DR- | rs1539433 | C | T | 0.544 | 0.025 | 5.03e-95 | 0.136 | 460.32 |
| MFI | CD45 on CD33dim HLA DR- | rs7084635 | A | G | 0.168 | 0.031 | 4.61e-08 | 0.010 | 30 |
| MFI | CD45 on CD33dim HLA DR- | rs7426056 | A | G | 0.3 | 0.028 | 1.67e-26 | 0.038 | 115.68 |
| MFI | CD45 on CD4+ | rs184018666 | C | T | -0.396 | 0.068 | 5.58e-09 | 0.010 | 34.13 |
| MFI | CD45 on CD4+ | rs71632979 | A | G | 0.809 | 0.028 | 9.85e-166 | 0.199 | 842.94 |
| MFI | CD45 on CD4+ | rs71639910 | G | A | 0.401 | 0.052 | 1.66e-14 | 0.017 | 59.39 |
| MFI | CD45 on CD4+ | rs74341264 | A | G | 0.407 | 0.04 | 5.49e-24 | 0.030 | 103.52 |
| MFI | CD45 on CD66b++ myelod cell | rs113013837 | A | G | 0.755 | 0.102 | 2.20e-13 | 0.015 | 54.2 |
| MFI | CD45 on CD66b++ myelod cell | rs137965091 | G | A | 0.384 | 0.067 | 1.27e-08 | 0.009 | 32.51 |
| MFI | CD45 on CD66b++ myelod cell | rs150239512 | A | G | 0.574 | 0.093 | 7.29e-10 | 0.010 | 38.12 |
| MFI | CD45 on CD66b++ myelod cell | rs150816467 | T | C | 0.583 | 0.091 | 1.88e-10 | 0.011 | 40.79 |
| MFI | CD45 on CD66b++ myelod cell | rs28739016 | T | G | 0.829 | 0.102 | 4.91e-16 | 0.018 | 66.46 |
| MFI | CD45 on CD8br | rs30006 | T | C | -0.191 | 0.029 | 8.55e-11 | 0.011 | 42.37 |
| MFI | CD45 on CD8br | rs4716010 | G | A | 0.192 | 0.032 | 2.24e-09 | 0.010 | 35.91 |
| MFI | CD45 on CD8br | rs61731791 | C | T | 0.236 | 0.042 | 2.51e-08 | 0.008 | 31.16 |
| MFI | CD45 on HLA DR+ CD4+ | rs10015947 | A | G | -289.6 | 31.26 | 3.34e-20 | 0.025 | 85.78 |
| MFI | CD45 on HLA DR+ CD4+ | rs10105261 | A | G | -254.2 | 38.99 | 8.12e-11 | 0.012 | 42.48 |
| MFI | CD45 on HLA DR+ CD4+ | rs10117573 | A | G | 84.89 | 14 | 1.48e-09 | 0.011 | 36.75 |
| MFI | CD45 on HLA DR+ CD4+ | rs10228844 | A | G | -415.6 | 45.02 | 4.57e-20 | 0.024 | 85.17 |
| MFI | CD45 on HLA DR+ CD4+ | rs10484068 | C | T | 118.5 | 17.59 | 1.86e-11 | 0.013 | 45.36 |
| MFI | CD45 on HLA DR+ CD4+ | rs10753659 | C | T | -410.8 | 52.74 | 8.92e-15 | 0.017 | 60.64 |
| MFI | CD45 on HLA DR+ CD4+ | rs10858195 | A | G | -542.2 | 68.73 | 4.06e-15 | 0.018 | 62.2 |
| MFI | CD45 on HLA DR+ CD4+ | rs1106305 | C | T | 95.49 | 13.66 | 3.28e-12 | 0.014 | 48.84 |
| MFI | CD45 on HLA DR+ CD4+ | rs11099578 | A | C | -204.6 | 34.24 | 2.55e-09 | 0.010 | 35.69 |
| MFI | CD45 on HLA DR+ CD4+ | rs11112760 | C | T | 82 | 13.96 | 4.67e-09 | 0.010 | 34.48 |
| MFI | CD45 on HLA DR+ CD4+ | rs111266370 | C | T | 72.35 | 12 | 1.83e-09 | 0.011 | 36.33 |
| MFI | CD45 on HLA DR+ CD4+ | rs111387857 | T | C | 101.7 | 17.12 | 3.13e-09 | 0.010 | 35.27 |
| MFI | CD45 on HLA DR+ CD4+ | rs111628737 | C | T | 96.59 | 17.01 | 1.46e-08 | 0.009 | 32.23 |
| MFI | CD45 on HLA DR+ CD4+ | rs11171776 | C | T | -303.8 | 51.37 | 3.67e-09 | 0.010 | 34.95 |
| MFI | CD45 on HLA DR+ CD4+ | rs1121709 | G | A | -1825 | 132.8 | 6.98e-42 | 0.053 | 188.74 |
| MFI | CD45 on HLA DR+ CD4+ | rs112517700 | G | A | 95.85 | 16.51 | 7.06e-09 | 0.010 | 33.68 |
| MFI | CD45 on HLA DR+ CD4+ | rs112532913 | T | C | 89.54 | 15.4 | 6.70e-09 | 0.010 | 33.79 |
| MFI | CD45 on HLA DR+ CD4+ | rs113370154 | C | A | 75.73 | 13.7 | 3.46e-08 | 0.009 | 30.54 |
| MFI | CD45 on HLA DR+ CD4+ | rs114988805 | T | G | 87.65 | 15.17 | 8.34e-09 | 0.010 | 33.36 |
| MFI | CD45 on HLA DR+ CD4+ | rs1150563 | T | C | -946.8 | 95.85 | 1.04e-22 | 0.028 | 97.52 |
| MFI | CD45 on HLA DR+ CD4+ | rs115565469 | T | C | 117.5 | 17.74 | 3.98e-11 | 0.013 | 43.84 |
| MFI | CD45 on HLA DR+ CD4+ | rs115574834 | A | G | 146.6 | 17.12 | 1.65e-17 | 0.021 | 73.28 |
| MFI | CD45 on HLA DR+ CD4+ | rs115647766 | C | T | 115.2 | 18.6 | 6.55e-10 | 0.011 | 38.34 |
| MFI | CD45 on HLA DR+ CD4+ | rs116110730 | G | A | -735.3 | 130.6 | 1.94e-08 | 0.009 | 31.68 |
| MFI | CD45 on HLA DR+ CD4+ | rs116451850 | A | G | 72.56 | 11.94 | 1.37e-09 | 0.011 | 36.91 |
| MFI | CD45 on HLA DR+ CD4+ | rs11661882 | G | A | -233.6 | 40.25 | 7.08e-09 | 0.010 | 33.66 |
| MFI | CD45 on HLA DR+ CD4+ | rs116938017 | A | G | 76.2 | 12.72 | 2.34e-09 | 0.010 | 35.87 |
| MFI | CD45 on HLA DR+ CD4+ | rs116971245 | G | A | 88.97 | 13.87 | 1.60e-10 | 0.012 | 41.12 |
| MFI | CD45 on HLA DR+ CD4+ | rs117150384 | A | G | 94.3 | 16.38 | 9.42e-09 | 0.010 | 33.12 |
| MFI | CD45 on HLA DR+ CD4+ | rs117638796 | G | A | 70.95 | 12.11 | 5.15e-09 | 0.010 | 34.31 |
| MFI | CD45 on HLA DR+ CD4+ | rs118120777 | A | G | 121.8 | 18.08 | 1.92e-11 | 0.013 | 45.36 |
| MFI | CD45 on HLA DR+ CD4+ | rs11859136 | A | G | 112.9 | 16.84 | 2.34e-11 | 0.013 | 44.92 |
| MFI | CD45 on HLA DR+ CD4+ | rs12036093 | G | A | 73.1 | 13 | 2.01e-08 | 0.009 | 31.6 |
| MFI | CD45 on HLA DR+ CD4+ | rs12130486 | T | C | 76.42 | 11.29 | 1.55e-11 | 0.013 | 45.79 |
| MFI | CD45 on HLA DR+ CD4+ | rs12435243 | A | C | -438.7 | 56.37 | 9.43e-15 | 0.017 | 60.53 |
| MFI | CD45 on HLA DR+ CD4+ | rs12711540 | A | G | -510.8 | 58.18 | 2.56e-18 | 0.022 | 77.04 |
| MFI | CD45 on HLA DR+ CD4+ | rs13010105 | A | G | 98.72 | 17.57 | 2.08e-08 | 0.009 | 31.55 |
| MFI | CD45 on HLA DR+ CD4+ | rs13104493 | A | G | 84.59 | 13.57 | 5.08e-10 | 0.011 | 38.84 |
| MFI | CD45 on HLA DR+ CD4+ | rs13337647 | T | C | 103.5 | 16.47 | 3.63e-10 | 0.011 | 39.47 |
| MFI | CD45 on HLA DR+ CD4+ | rs1335099 | T | C | -394.5 | 60.52 | 8.20e-11 | 0.012 | 42.47 |
| MFI | CD45 on HLA DR+ CD4+ | rs135065 | T | C | -91.04 | 15.63 | 6.20e-09 | 0.010 | 33.91 |
| MFI | CD45 on HLA DR+ CD4+ | rs137976632 | C | T | 76.4 | 11.98 | 2.03e-10 | 0.012 | 40.65 |
| MFI | CD45 on HLA DR+ CD4+ | rs138948609 | G | A | 59.98 | 10.31 | 6.47e-09 | 0.010 | 33.83 |
| MFI | CD45 on HLA DR+ CD4+ | rs139498809 | G | T | 88.88 | 14.91 | 2.76e-09 | 0.010 | 35.51 |
| MFI | CD45 on HLA DR+ CD4+ | rs139741196 | G | A | 100.8 | 17.73 | 1.44e-08 | 0.009 | 32.3 |
| MFI | CD45 on HLA DR+ CD4+ | rs140426322 | C | T | 95.5 | 15.71 | 1.33e-09 | 0.011 | 36.93 |
| MFI | CD45 on HLA DR+ CD4+ | rs140800343 | C | T | 69.9 | 12.06 | 7.48e-09 | 0.010 | 33.57 |
| MFI | CD45 on HLA DR+ CD4+ | rs140976870 | C | T | 110.2 | 16.85 | 6.99e-11 | 0.012 | 42.75 |
| MFI | CD45 on HLA DR+ CD4+ | rs141291585 | C | A | 88.95 | 15.51 | 1.07e-08 | 0.010 | 32.87 |
| MFI | CD45 on HLA DR+ CD4+ | rs143028194 | C | A | 112.5 | 17.54 | 1.60e-10 | 0.012 | 41.11 |
| MFI | CD45 on HLA DR+ CD4+ | rs143355478 | G | A | 97.4 | 17.6 | 3.37e-08 | 0.009 | 30.61 |
| MFI | CD45 on HLA DR+ CD4+ | rs143940797 | G | A | 43.94 | 7.987 | 4.03e-08 | 0.009 | 30.25 |
| MFI | CD45 on HLA DR+ CD4+ | rs144011128 | A | G | 97.53 | 17.06 | 1.17e-08 | 0.009 | 32.66 |
| MFI | CD45 on HLA DR+ CD4+ | rs146360329 | T | C | 73.8 | 13.01 | 1.52e-08 | 0.009 | 32.16 |
| MFI | CD45 on HLA DR+ CD4+ | rs146627868 | G | A | 90.03 | 14.57 | 7.31e-10 | 0.011 | 38.16 |
| MFI | CD45 on HLA DR+ CD4+ | rs146863207 | T | C | 75.62 | 13.68 | 3.50e-08 | 0.009 | 30.54 |
| MFI | CD45 on HLA DR+ CD4+ | rs147004969 | A | G | -2921 | 127.3 | 1.99e-108 | 0.134 | 526.2 |
| MFI | CD45 on HLA DR+ CD4+ | rs147026353 | G | T | 107.1 | 15.52 | 6.24e-12 | 0.014 | 47.59 |
| MFI | CD45 on HLA DR+ CD4+ | rs1481689 | T | G | -423.4 | 68.01 | 5.37e-10 | 0.011 | 38.73 |
| MFI | CD45 on HLA DR+ CD4+ | rs148259347 | A | G | -494.1 | 81.38 | 1.40e-09 | 0.011 | 36.84 |
| MFI | CD45 on HLA DR+ CD4+ | rs148402893 | T | C | 58.91 | 10.58 | 2.78e-08 | 0.009 | 30.99 |
| MFI | CD45 on HLA DR+ CD4+ | rs149668689 | G | A | 93.27 | 17.03 | 4.65e-08 | 0.009 | 29.98 |
| MFI | CD45 on HLA DR+ CD4+ | rs150374010 | T | C | 54.47 | 9.679 | 1.97e-08 | 0.009 | 31.65 |
| MFI | CD45 on HLA DR+ CD4+ | rs150382715 | T | C | -1321 | 88.86 | 1.72e-48 | 0.061 | 220.87 |
| MFI | CD45 on HLA DR+ CD4+ | rs150840857 | G | A | 85 | 15.4 | 3.67e-08 | 0.009 | 30.45 |
| MFI | CD45 on HLA DR+ CD4+ | rs1590822 | T | C | -140.1 | 23.49 | 2.76e-09 | 0.010 | 35.55 |
| MFI | CD45 on HLA DR+ CD4+ | rs1613817 | G | A | -410.3 | 68.05 | 1.82e-09 | 0.011 | 36.33 |
| MFI | CD45 on HLA DR+ CD4+ | rs17016743 | C | T | 105.6 | 17.67 | 2.56e-09 | 0.010 | 35.69 |
| MFI | CD45 on HLA DR+ CD4+ | rs180886600 | T | G | 86.2 | 15.49 | 2.81e-08 | 0.009 | 30.95 |
| MFI | CD45 on HLA DR+ CD4+ | rs182750448 | T | C | 101.3 | 17.85 | 1.50e-08 | 0.009 | 32.19 |
| MFI | CD45 on HLA DR+ CD4+ | rs183543370 | C | T | 89.14 | 14.04 | 2.44e-10 | 0.012 | 40.29 |
| MFI | CD45 on HLA DR+ CD4+ | rs183791586 | A | G | 104.3 | 16.31 | 1.82e-10 | 0.012 | 40.87 |
| MFI | CD45 on HLA DR+ CD4+ | rs184444615 | C | T | 60.29 | 10.42 | 7.79e-09 | 0.010 | 33.46 |
| MFI | CD45 on HLA DR+ CD4+ | rs184779498 | G | A | 115.2 | 16.69 | 6.11e-12 | 0.014 | 47.61 |
| MFI | CD45 on HLA DR+ CD4+ | rs187344708 | C | T | 122.7 | 17.03 | 7.13e-13 | 0.015 | 51.88 |
| MFI | CD45 on HLA DR+ CD4+ | rs188522 | A | G | -203 | 37.12 | 4.83e-08 | 0.009 | 29.89 |
| MFI | CD45 on HLA DR+ CD4+ | rs189004510 | C | A | 106.3 | 15.57 | 1.01e-11 | 0.013 | 46.58 |
| MFI | CD45 on HLA DR+ CD4+ | rs189420173 | T | G | 72.54 | 12.91 | 2.10e-08 | 0.009 | 31.55 |
| MFI | CD45 on HLA DR+ CD4+ | rs191576893 | G | A | 105.8 | 16.96 | 5.09e-10 | 0.011 | 38.89 |
| MFI | CD45 on HLA DR+ CD4+ | rs192481283 | G | A | 131.8 | 18.34 | 8.03e-13 | 0.015 | 51.62 |
| MFI | CD45 on HLA DR+ CD4+ | rs193099284 | G | A | 105.2 | 17.42 | 1.75e-09 | 0.011 | 36.45 |
| MFI | CD45 on HLA DR+ CD4+ | rs1948837 | G | T | -310.1 | 36.71 | 4.40e-17 | 0.021 | 71.31 |
| MFI | CD45 on HLA DR+ CD4+ | rs201243595 | A | G | -532.5 | 77.41 | 7.15e-12 | 0.014 | 47.29 |
| MFI | CD45 on HLA DR+ CD4+ | rs201988582 | C | A | -416.3 | 48.8 | 2.19e-17 | 0.021 | 72.73 |
| MFI | CD45 on HLA DR+ CD4+ | rs202141816 | C | T | -675.6 | 76.85 | 2.31e-18 | 0.022 | 77.24 |
| MFI | CD45 on HLA DR+ CD4+ | rs2054143 | A | G | -1827 | 132.8 | 6.06e-42 | 0.053 | 189.16 |
| MFI | CD45 on HLA DR+ CD4+ | rs2058165 | T | C | -532.8 | 58.87 | 2.33e-19 | 0.023 | 81.86 |
| MFI | CD45 on HLA DR+ CD4+ | rs2190238 | G | T | -623.1 | 65.23 | 2.36e-21 | 0.026 | 91.19 |
| MFI | CD45 on HLA DR+ CD4+ | rs220485 | G | A | -303.5 | 53.98 | 2.03e-08 | 0.009 | 31.59 |
| MFI | CD45 on HLA DR+ CD4+ | rs2244407 | T | C | -223.3 | 35.26 | 2.73e-10 | 0.012 | 40.08 |
| MFI | CD45 on HLA DR+ CD4+ | rs237926 | T | C | -478 | 49.45 | 7.96e-22 | 0.027 | 93.38 |
| MFI | CD45 on HLA DR+ CD4+ | rs2403905 | G | A | -414.2 | 54.58 | 4.15e-14 | 0.017 | 57.56 |
| MFI | CD45 on HLA DR+ CD4+ | rs2425025 | G | A | -204.9 | 34.06 | 1.98e-09 | 0.011 | 36.17 |
| MFI | CD45 on HLA DR+ CD4+ | rs2492054 | A | G | -222.2 | 35.02 | 2.51e-10 | 0.012 | 40.23 |
| MFI | CD45 on HLA DR+ CD4+ | rs2496150 | A | G | -388.2 | 50.03 | 1.11e-14 | 0.017 | 60.17 |
| MFI | CD45 on HLA DR+ CD4+ | rs260694 | G | T | -209.3 | 36.71 | 1.28e-08 | 0.009 | 32.49 |
| MFI | CD45 on HLA DR+ CD4+ | rs2659579 | A | G | -1289 | 104.1 | 1.77e-34 | 0.043 | 153.23 |
| MFI | CD45 on HLA DR+ CD4+ | rs2659584 | C | T | -514.5 | 56.53 | 1.48e-19 | 0.024 | 82.79 |
| MFI | CD45 on HLA DR+ CD4+ | rs2789440 | C | T | -138.1 | 22.61 | 1.12e-09 | 0.011 | 37.28 |
| MFI | CD45 on HLA DR+ CD4+ | rs278970 | C | T | -315.7 | 41.57 | 3.99e-14 | 0.017 | 57.64 |
| MFI | CD45 on HLA DR+ CD4+ | rs2815430 | A | G | -206.2 | 33.66 | 1.01e-09 | 0.011 | 37.51 |
| MFI | CD45 on HLA DR+ CD4+ | rs2820583 | T | C | -181.7 | 32.19 | 1.78e-08 | 0.009 | 31.84 |
| MFI | CD45 on HLA DR+ CD4+ | rs285103 | A | C | -263.3 | 31.34 | 6.40e-17 | 0.020 | 70.54 |
| MFI | CD45 on HLA DR+ CD4+ | rs2940682 | G | T | -211.1 | 36.75 | 1.00e-08 | 0.010 | 32.98 |
| MFI | CD45 on HLA DR+ CD4+ | rs307834 | G | A | -158.3 | 21.42 | 1.87e-13 | 0.016 | 54.58 |
| MFI | CD45 on HLA DR+ CD4+ | rs334513 | A | G | -505.2 | 49.92 | 9.74e-24 | 0.029 | 102.36 |
| MFI | CD45 on HLA DR+ CD4+ | rs35514807 | A | G | 118.7 | 17.88 | 3.66e-11 | 0.013 | 44.05 |
| MFI | CD45 on HLA DR+ CD4+ | rs421380 | C | T | -201.1 | 34.87 | 8.75e-09 | 0.010 | 33.24 |
| MFI | CD45 on HLA DR+ CD4+ | rs4253965 | T | C | -242.7 | 37.05 | 6.59e-11 | 0.012 | 42.89 |
| MFI | CD45 on HLA DR+ CD4+ | rs4265908 | G | A | -881.5 | 118.2 | 1.13e-13 | 0.016 | 55.58 |
| MFI | CD45 on HLA DR+ CD4+ | rs430434 | A | G | -234.4 | 41.93 | 2.45e-08 | 0.009 | 31.23 |
| MFI | CD45 on HLA DR+ CD4+ | rs4324550 | C | T | -340.6 | 45.69 | 1.14e-13 | 0.016 | 55.54 |
| MFI | CD45 on HLA DR+ CD4+ | rs4593123 | G | A | -190.5 | 34.08 | 2.44e-08 | 0.009 | 31.23 |
| MFI | CD45 on HLA DR+ CD4+ | rs4821845 | C | T | -659.9 | 84.5 | 7.60e-15 | 0.018 | 60.95 |
| MFI | CD45 on HLA DR+ CD4+ | rs533167188 | A | G | 83.33 | 14.57 | 1.17e-08 | 0.010 | 32.69 |
| MFI | CD45 on HLA DR+ CD4+ | rs536135674 | T | C | 95.49 | 17.1 | 2.54e-08 | 0.009 | 31.17 |
| MFI | CD45 on HLA DR+ CD4+ | rs541823325 | C | T | 114 | 19.49 | 5.42e-09 | 0.010 | 34.19 |
| MFI | CD45 on HLA DR+ CD4+ | rs547829064 | A | G | 98.15 | 17.88 | 4.30e-08 | 0.009 | 30.12 |
| MFI | CD45 on HLA DR+ CD4+ | rs547935704 | A | C | 75.58 | 13.24 | 1.24e-08 | 0.009 | 32.57 |
| MFI | CD45 on HLA DR+ CD4+ | rs554895660 | A | G | 98.79 | 15.31 | 1.27e-10 | 0.012 | 41.61 |
| MFI | CD45 on HLA DR+ CD4+ | rs555008713 | G | A | 98.73 | 14.85 | 3.43e-11 | 0.013 | 44.18 |
| MFI | CD45 on HLA DR+ CD4+ | rs558860878 | A | C | 100.8 | 17.61 | 1.13e-08 | 0.010 | 32.75 |
| MFI | CD45 on HLA DR+ CD4+ | rs561775102 | A | C | 86.55 | 13.71 | 3.09e-10 | 0.012 | 39.83 |
| MFI | CD45 on HLA DR+ CD4+ | rs56311462 | G | A | -659.6 | 64.97 | 7.01e-24 | 0.029 | 103.01 |
| MFI | CD45 on HLA DR+ CD4+ | rs564696 | A | G | -429.7 | 51.34 | 8.32e-17 | 0.020 | 70.01 |
| MFI | CD45 on HLA DR+ CD4+ | rs568554963 | G | A | 108.3 | 17.42 | 5.80e-10 | 0.011 | 38.63 |
| MFI | CD45 on HLA DR+ CD4+ | rs59615051 | T | C | 126 | 15.9 | 3.05e-15 | 0.018 | 62.76 |
| MFI | CD45 on HLA DR+ CD4+ | rs6026066 | A | C | -231.4 | 32.61 | 1.58e-12 | 0.015 | 50.32 |
| MFI | CD45 on HLA DR+ CD4+ | rs61632622 | G | A | 113.6 | 17.16 | 4.14e-11 | 0.013 | 43.8 |
| MFI | CD45 on HLA DR+ CD4+ | rs6424838 | T | C | -2921 | 127.3 | 1.99e-108 | 0.134 | 526.2 |
| MFI | CD45 on HLA DR+ CD4+ | rs6471765 | A | G | -159.5 | 28.14 | 1.57e-08 | 0.009 | 32.11 |
| MFI | CD45 on HLA DR+ CD4+ | rs6517382 | T | C | -548.4 | 57.56 | 2.95e-21 | 0.026 | 90.72 |
| MFI | CD45 on HLA DR+ CD4+ | rs6725974 | C | T | -479.8 | 68.18 | 2.37e-12 | 0.014 | 49.49 |
| MFI | CD45 on HLA DR+ CD4+ | rs6927181 | C | T | -146.2 | 20.86 | 2.85e-12 | 0.014 | 49.09 |
| MFI | CD45 on HLA DR+ CD4+ | rs7192419 | G | A | 115.1 | 17.5 | 5.49e-11 | 0.013 | 43.23 |
| MFI | CD45 on HLA DR+ CD4+ | rs72557975 | G | A | -1761 | 129.6 | 5.76e-41 | 0.051 | 184.52 |
| MFI | CD45 on HLA DR+ CD4+ | rs73206236 | G | A | 130.7 | 17.31 | 5.64e-14 | 0.016 | 56.98 |
| MFI | CD45 on HLA DR+ CD4+ | rs73369750 | A | G | -767.3 | 82.16 | 1.70e-20 | 0.025 | 87.17 |
| MFI | CD45 on HLA DR+ CD4+ | rs73815908 | G | A | -156.9 | 28.19 | 2.81e-08 | 0.009 | 30.96 |
| MFI | CD45 on HLA DR+ CD4+ | rs74540476 | G | A | 90.23 | 16.28 | 3.21e-08 | 0.009 | 30.7 |
| MFI | CD45 on HLA DR+ CD4+ | rs75054940 | C | T | 100.8 | 17.68 | 1.27e-08 | 0.009 | 32.49 |
| MFI | CD45 on HLA DR+ CD4+ | rs75177063 | T | C | 101.5 | 17.69 | 1.05e-08 | 0.010 | 32.9 |
| MFI | CD45 on HLA DR+ CD4+ | rs75287501 | G | A | 102.6 | 15.35 | 2.72e-11 | 0.013 | 44.65 |
| MFI | CD45 on HLA DR+ CD4+ | rs75907214 | C | T | 91.22 | 16.4 | 2.84e-08 | 0.009 | 30.92 |
| MFI | CD45 on HLA DR+ CD4+ | rs7597139 | G | A | -87.22 | 15.38 | 1.55e-08 | 0.009 | 32.14 |
| MFI | CD45 on HLA DR+ CD4+ | rs7613821 | A | G | -287.7 | 48.86 | 4.25e-09 | 0.010 | 34.65 |
| MFI | CD45 on HLA DR+ CD4+ | rs7664060 | G | A | -254.2 | 38.15 | 3.09e-11 | 0.013 | 44.37 |
| MFI | CD45 on HLA DR+ CD4+ | rs773483 | G | A | -138.9 | 24.38 | 1.34e-08 | 0.009 | 32.44 |
| MFI | CD45 on HLA DR+ CD4+ | rs7794931 | C | T | -101.4 | 16.35 | 6.19e-10 | 0.011 | 38.44 |
| MFI | CD45 on HLA DR+ CD4+ | rs78110305 | C | T | -860.9 | 130.2 | 4.44e-11 | 0.013 | 43.69 |
| MFI | CD45 on HLA DR+ CD4+ | rs78275221 | G | A | 101.4 | 17.52 | 7.68e-09 | 0.010 | 33.48 |
| MFI | CD45 on HLA DR+ CD4+ | rs78374377 | G | A | 110.8 | 15.16 | 3.34e-13 | 0.015 | 53.39 |
| MFI | CD45 on HLA DR+ CD4+ | rs78437522 | A | G | -580.3 | 60.69 | 2.16e-21 | 0.026 | 91.37 |
| MFI | CD45 on HLA DR+ CD4+ | rs78622624 | T | C | 97.68 | 15.15 | 1.30e-10 | 0.012 | 41.55 |
| MFI | CD45 on HLA DR+ CD4+ | rs78641098 | C | T | 88.05 | 15.87 | 3.10e-08 | 0.009 | 30.76 |
| MFI | CD45 on HLA DR+ CD4+ | rs7872296 | A | G | -159 | 26.49 | 2.13e-09 | 0.010 | 36.01 |
| MFI | CD45 on HLA DR+ CD4+ | rs7955573 | T | C | -305.9 | 46.07 | 3.62e-11 | 0.013 | 44.06 |
| MFI | CD45 on HLA DR+ CD4+ | rs79757812 | G | A | 99.34 | 16.98 | 5.33e-09 | 0.010 | 34.21 |
| MFI | CD45 on HLA DR+ CD4+ | rs79888370 | G | T | 47.59 | 8.691 | 4.66e-08 | 0.009 | 29.97 |
| MFI | CD45 on HLA DR+ CD4+ | rs8099106 | A | G | -1013 | 137.6 | 2.25e-13 | 0.016 | 54.17 |
| MFI | CD45 on HLA DR+ CD4+ | rs849576 | A | G | -173.5 | 31.09 | 2.56e-08 | 0.009 | 31.12 |
| MFI | CD45 on HLA DR+ CD4+ | rs914512 | G | A | -254.6 | 28.16 | 2.56e-19 | 0.023 | 81.7 |
| MFI | CD45 on HLA DR+ CD4+ | rs9433776 | T | C | -189.4 | 28.15 | 1.99e-11 | 0.013 | 45.24 |
| MFI | CD45 on HLA DR+ CD4+ | rs9551076 | C | T | -300 | 39.11 | 2.19e-14 | 0.017 | 58.8 |
| MFI | CD45 on HLA DR+ CD4+ | rs9886595 | C | A | -207.9 | 24.66 | 4.96e-17 | 0.020 | 71.03 |
| MFI | CD45 on HLA DR+ CD4+ | rs9989264 | G | A | -252.6 | 43.04 | 4.77e-09 | 0.010 | 34.42 |
| MFI | CD45 on Mo MDSC | rs112062469 | G | A | -0.537 | 0.058 | 3.09e-20 | 0.023 | 85.88 |
| MFI | CD45 on Mo MDSC | rs112266065 | C | T | -1.05 | 0.115 | 1.42e-19 | 0.022 | 82.74 |
| MFI | CD45 on Mo MDSC | rs114919524 | A | C | -1.135 | 0.107 | 5.50e-26 | 0.030 | 112.67 |
| MFI | CD45 on Mo MDSC | rs117119882 | C | A | -0.937 | 0.116 | 7.50e-16 | 0.018 | 65.59 |
| MFI | CD45 on Mo MDSC | rs141059303 | G | A | -0.646 | 0.117 | 3.87e-08 | 0.008 | 30.35 |
| MFI | CD45 on Mo MDSC | rs144365928 | A | G | -0.699 | 0.078 | 3.71e-19 | 0.022 | 80.87 |
| MFI | CD45 on Mo MDSC | rs146434619 | T | C | -0.657 | 0.045 | 2.19e-47 | 0.056 | 215.08 |
| MFI | CD45 on Mo MDSC | rs148516965 | C | T | -0.566 | 0.056 | 1.19e-23 | 0.027 | 101.84 |
| MFI | CD45 on Mo MDSC | rs149167072 | A | G | 0.351 | 0.053 | 6.05e-11 | 0.012 | 43.05 |
| MFI | CD45 on Mo MDSC | rs149806587 | A | G | -0.526 | 0.057 | 9.05e-20 | 0.022 | 83.71 |
| MFI | CD45 on Mo MDSC | rs150288192 | C | T | -1.22 | 0.117 | 3.16e-25 | 0.029 | 109.23 |
| MFI | CD45 on Mo MDSC | rs185098316 | G | A | -0.311 | 0.052 | 3.19e-09 | 0.010 | 35.21 |
| MFI | CD45 on Mo MDSC | rs185430947 | A | G | -0.997 | 0.108 | 6.63e-20 | 0.023 | 84.34 |
| MFI | CD45 on Mo MDSC | rs188216171 | G | A | -0.945 | 0.11 | 1.03e-17 | 0.020 | 74.12 |
| MFI | CD45 on Mo MDSC | rs189799494 | G | T | -0.668 | 0.05 | 3.75e-39 | 0.046 | 175.42 |
| MFI | CD45 on Mo MDSC | rs191363479 | C | T | -1 | 0.11 | 1.18e-19 | 0.022 | 83.14 |
| MFI | CD45 on Mo MDSC | rs193039847 | G | A | -1.39 | 0.087 | 4.79e-56 | 0.066 | 257.43 |
| MFI | CD45 on Mo MDSC | rs535059510 | C | T | -0.998 | 0.118 | 3.35e-17 | 0.019 | 71.79 |
| MFI | CD45 on Mo MDSC | rs539130256 | T | C | -0.602 | 0.106 | 1.23e-08 | 0.009 | 32.56 |
| MFI | CD45 on Mo MDSC | rs539241708 | C | T | -1.224 | 0.085 | 9.98e-46 | 0.054 | 207.2 |
| MFI | CD45 on Mo MDSC | rs556260593 | A | C | -1.007 | 0.106 | 3.17e-21 | 0.024 | 90.54 |
| MFI | CD45 on Mo MDSC | rs557577522 | G | A | -0.791 | 0.071 | 1.59e-28 | 0.033 | 124.75 |
| MFI | CD45 on Mo MDSC | rs558720004 | A | C | -1.265 | 0.11 | 2.81e-30 | 0.035 | 133.14 |
| MFI | CD45 on Mo MDSC | rs568374507 | C | T | -0.598 | 0.059 | 4.67e-24 | 0.028 | 103.76 |
| MFI | CD45 on Mo MDSC | rs6002539 | A | G | -1.249 | 0.033 | 1.00e-200 | 0.279 | 1411.12 |
| MFI | CD45 on Mo MDSC | rs78806227 | C | T | -0.972 | 0.12 | 9.66e-16 | 0.017 | 64.99 |
| MFI | CD45 on Mo MDSC | rs79085286 | T | C | -0.391 | 0.053 | 3.18e-13 | 0.014 | 53.45 |
| MFI | CD45 on NK | rs113861918 | C | T | -0.215 | 0.037 | 6.42e-09 | 0.012 | 33.89 |
| MFI | CD45 on NK | rs1801274 | A | G | 0.248 | 0.03 | 1.36e-16 | 0.024 | 69.12 |
| MFI | CD45 on NK | rs7185541 | A | G | -0.171 | 0.03 | 1.98e-08 | 0.011 | 31.69 |
| MFI | CD45 on NK | rs8046381 | T | C | 0.468 | 0.041 | 9.63e-30 | 0.044 | 131.19 |
| MFI | CD45 on basophil | rs1152928 | A | G | -0.752 | 0.102 | 2.48e-13 | 0.017 | 53.99 |
| MFI | CD45 on basophil | rs11554159 | G | A | -0.299 | 0.028 | 1.82e-25 | 0.034 | 110.62 |
| MFI | CD45 on basophil | rs117630441 | C | T | -0.843 | 0.088 | 2.63e-21 | 0.028 | 91.07 |
| MFI | CD45 on basophil | rs145992681 | C | T | -0.631 | 0.115 | 4.77e-08 | 0.010 | 29.93 |
| MFI | CD45 on basophil | rs1800973 | C | A | -1.048 | 0.044 | 3.29e-114 | 0.153 | 561.06 |
| MFI | CD45 on basophil | rs445 | C | T | 0.229 | 0.033 | 4.94e-12 | 0.015 | 48.07 |
| MFI | CD45 on basophil | rs75066377 | G | T | -0.245 | 0.041 | 1.74e-09 | 0.012 | 36.44 |
| MFI | CD45 on lymphocyte | rs2236073 | G | A | 0.176 | 0.025 | 2.96e-12 | 0.013 | 48.99 |
| MFI | CD45 on lymphocyte | rs72948545 | A | G | -0.744 | 0.052 | 2.52e-45 | 0.053 | 205.09 |
| MFI | CD45 on lymphocyte | rs783414 | A | G | -0.205 | 0.033 | 6.96e-10 | 0.010 | 38.23 |
| MFI | CD45 on lymphocyte | rs28634695 | G | A | -0.203 | 0.025 | 5.70e-16 | 0.018 | 66.13 |
| MFI | CD45 on lymphocyte | rs28723652 | C | A | 0.434 | 0.073 | 3.22e-09 | 0.010 | 35.2 |
| MFI | CD45 on lymphocyte | rs35501848 | G | T | -0.163 | 0.025 | 6.58e-11 | 0.012 | 42.85 |
| MFI | CD45 on lymphocyte | rs709589 | C | T | -0.663 | 0.03 | 4.57e-103 | 0.119 | 495.5 |
| MFI | CD4RA on TD CD4+ | rs11615628 | G | A | -0.206 | 0.031 | 5.49e-11 | 0.015 | 43.29 |
| MFI | CD4RA on TD CD4+ | rs2253487 | G | A | 0.221 | 0.03 | 1.42e-13 | 0.019 | 55.13 |
| MFI | CD4RA on TD CD4+ | rs9263475 | A | G | 0.203 | 0.03 | 9.61e-12 | 0.016 | 46.73 |
| MFI | CD62L on CD62L+ myeloid DC | rs2988277 | C | T | 0.463 | 0.026 | 9.75e-69 | 0.100 | 323.72 |
| MFI | CD62L on CD62L+ myeloid DC | rs41289614 | C | T | 0.188 | 0.034 | 3.94e-08 | 0.010 | 30.33 |
| MFI | CD62L on CD62L+ myeloid DC | rs4657649 | T | C | 0.433 | 0.06 | 8.31e-13 | 0.017 | 51.64 |
| MFI | CD62L on monocyte | rs10882701 | A | C | -0.403 | 0.025 | 2.04e-56 | 0.070 | 259.8 |
| MFI | CD62L on monocyte | rs11595506 | C | T | -0.418 | 0.059 | 2.57e-12 | 0.014 | 49.33 |
| MFI | CD62L on monocyte | rs12245404 | G | A | 0.648 | 0.115 | 2.06e-08 | 0.009 | 31.59 |
| MFI | CD62L on monocyte | rs141018090 | G | T | 0.558 | 0.056 | 3.17e-23 | 0.028 | 99.94 |
| MFI | CD62L on monocyte | rs185198079 | C | T | 0.852 | 0.095 | 5.27e-19 | 0.023 | 80.21 |
| MFI | CD62L on monocyte | rs191819784 | G | A | 0.803 | 0.077 | 2.19e-25 | 0.031 | 110.08 |
| MFI | CD62L on monocyte | rs191910319 | G | A | 0.466 | 0.07 | 3.38e-11 | 0.013 | 44.2 |
| MFI | CD62L on monocyte | rs193277455 | A | G | -0.656 | 0.109 | 1.85e-09 | 0.010 | 36.3 |
| MFI | CD62L on monocyte | rs1934968 | A | G | -0.32 | 0.053 | 1.46e-09 | 0.011 | 36.76 |
| MFI | CD62L on monocyte | rs77218771 | T | C | 0.946 | 0.129 | 2.74e-13 | 0.015 | 53.82 |
| MFI | CD62L on monocyte | rs7906684 | G | A | 1.07 | 0.08 | 2.10e-39 | 0.049 | 176.66 |
| MFI | CD62L on monocyte | rs7921242 | A | G | 1.04 | 0.019 | 1.00e-200 | 0.456 | 2878.1 |
| MFI | CD64 on CD14+ CD16- monocyte | rs1084559 | G | A | -0.147 | 0.025 | 3.32e-09 | 0.010 | 35.15 |
| MFI | CD64 on CD14+ CD16- monocyte | rs2181036 | T | C | -0.163 | 0.026 | 2.58e-10 | 0.011 | 40.17 |
| MFI | CD64 on CD14+ CD16- monocyte | rs3087456 | G | A | -0.436 | 0.027 | 3.62e-55 | 0.066 | 253.22 |
| MFI | CD64 on CD14+ CD16- monocyte | rs68174286 | C | T | 0.326 | 0.045 | 6.53e-13 | 0.014 | 52.04 |
| MFI | CD64 on CD14+ CD16- monocyte | rs72779785 | A | G | -0.202 | 0.033 | 6.46e-10 | 0.011 | 38.35 |
| MFI | CD64 on monocyte | rs10919544 | T | C | -0.256 | 0.027 | 2.40e-20 | 0.027 | 86.58 |
| MFI | CD64 on monocyte | rs148849191 | C | T | -0.782 | 0.121 | 1.11e-10 | 0.013 | 41.9 |
| MFI | CD64 on monocyte | rs17504675 | G | A | -0.47 | 0.076 | 8.59e-10 | 0.012 | 37.84 |
| MFI | CD64 on monocyte | rs186817752 | A | G | -0.526 | 0.084 | 4.83e-10 | 0.012 | 38.97 |
| MFI | CD64 on monocyte | rs188738981 | C | T | -0.697 | 0.088 | 4.41e-15 | 0.020 | 62.09 |
| MFI | CD64 on monocyte | rs191904494 | T | C | -0.718 | 0.095 | 4.40e-14 | 0.018 | 57.48 |
| MFI | CD64 on monocyte | rs45602031 | C | T | -0.341 | 0.05 | 1.30e-11 | 0.015 | 46.15 |
| MFI | CD64 on monocyte | rs77291736 | C | T | -1.076 | 0.038 | 6.93e-155 | 0.202 | 787.94 |
| MFI | CD64 on monocyte | rs79553258 | G | A | -0.734 | 0.111 | 3.88e-11 | 0.014 | 43.94 |
| MFI | CD66b on CD66b++ myeloid cell | rs113013837 | A | G | 0.716 | 0.102 | 3.43e-12 | 0.013 | 48.71 |
| MFI | CD66b on CD66b++ myeloid cell | rs137965091 | G | A | 0.39 | 0.067 | 7.36e-09 | 0.009 | 33.58 |
| MFI | CD66b on CD66b++ myeloid cell | rs139732336 | C | T | 0.518 | 0.09 | 1.02e-08 | 0.009 | 32.94 |
| MFI | CD66b on CD66b++ myeloid cell | rs150816467 | T | C | 0.546 | 0.091 | 2.44e-09 | 0.010 | 35.74 |
| MFI | CD66b on CD66b++ myeloid cell | rs28739016 | T | G | 0.779 | 0.102 | 2.44e-14 | 0.016 | 58.53 |
| MFI | CD66b on Gr MDSC | rs11086984 | C | T | 0.256 | 0.034 | 1.30e-13 | 0.015 | 55.22 |
| MFI | CD66b on Gr MDSC | rs147714617 | C | T | 0.258 | 0.037 | 5.22e-12 | 0.013 | 47.91 |
| MFI | CD66b on Gr MDSC | rs149803677 | A | C | 0.514 | 0.068 | 5.35e-14 | 0.015 | 57.02 |
| MFI | CD66b on Gr MDSC | rs162076 | G | A | -0.145 | 0.025 | 6.20e-09 | 0.009 | 33.92 |
| MFI | CD66b on Gr MDSC | rs1801274 | A | G | -0.221 | 0.026 | 7.07e-17 | 0.019 | 70.34 |
| MFI | CD66b on Gr MDSC | rs25680 | G | A | -0.262 | 0.033 | 2.86e-15 | 0.017 | 62.85 |
| MFI | CD66b on Gr MDSC | rs34101571 | G | A | -0.45 | 0.079 | 1.45e-08 | 0.009 | 32.24 |
| MFI | CD66b on Gr MDSC | rs35574803 | T | C | 0.759 | 0.052 | 3.63e-46 | 0.054 | 209.16 |
| MFI | CD66b on Gr MDSC | rs529513098 | T | G | 0.635 | 0.099 | 1.69e-10 | 0.011 | 41.01 |
| MFI | CD66b on Gr MDSC | rs551832918 | A | G | 0.764 | 0.104 | 2.67e-13 | 0.015 | 53.8 |
| MFI | CD66b on Gr MDSC | rs564347773 | G | A | 0.664 | 0.078 | 3.15e-17 | 0.019 | 71.93 |
| MFI | CD66b on Gr MDSC | rs569077190 | G | A | 0.368 | 0.055 | 1.83e-11 | 0.012 | 45.4 |
| MFI | CD66b on Gr MDSC | rs9989924 | T | C | 0.152 | 0.026 | 7.02e-09 | 0.009 | 33.68 |
| MFI | CD8 on CD8br | rs10882655 | G | A | -0.294 | 0.027 | 7.84e-28 | 0.040 | 122.03 |
| MFI | CD8 on CD8br | rs9263475 | A | G | 0.208 | 0.029 | 1.11e-12 | 0.017 | 51.03 |
| MFI | CD8 on CD8br | rs9265953 | A | G | -0.173 | 0.029 | 2.73e-09 | 0.012 | 35.56 |
| MFI | CD8 on CD28+ CD45RA- CD8br | rs114672530 | G | A | -0.668 | 0.104 | 1.46e-10 | 0.014 | 41.34 |
| MFI | CD8 on CD28+ CD45RA- CD8br | rs12138291 | G | A | -0.274 | 0.038 | 1.00e-12 | 0.017 | 51.28 |
| MFI | CD8 on CD28+ CD45RA- CD8br | rs16848876 | A | G | -0.556 | 0.093 | 2.89e-09 | 0.012 | 35.45 |
| MFI | CD8 on CD28+ CD45RA- CD8br | rs2949661 | C | T | 0.628 | 0.025 | 6.96e-128 | 0.180 | 639.75 |
| MFI | CD8 on CD28+ CD45RA- CD8br | rs35055340 | C | T | -0.484 | 0.076 | 2.49e-10 | 0.014 | 40.29 |
| MFI | CD8 on CD28+ CD45RA- CD8br | rs72703432 | A | G | -0.493 | 0.085 | 8.07e-09 | 0.011 | 33.44 |
| MFI | CD8 on CD39+ CD8br | rs11086984 | C | T | 0.274 | 0.034 | 1.59e-15 | 0.017 | 64.02 |
| MFI | CD8 on CD39+ CD8br | rs147714617 | C | T | 0.264 | 0.037 | 1.33e-12 | 0.014 | 50.64 |
| MFI | CD8 on CD39+ CD8br | rs162076 | G | A | -0.163 | 0.025 | 5.03e-11 | 0.012 | 43.42 |
| MFI | CD8 on CD39+ CD8br | rs1801274 | A | G | -0.204 | 0.026 | 1.14e-14 | 0.016 | 60.08 |
| MFI | CD8 on CD39+ CD8br | rs25680 | G | A | -0.212 | 0.033 | 1.29e-10 | 0.011 | 41.55 |
| MFI | CD8 on CD39+ CD8br | rs34101571 | G | A | -0.534 | 0.079 | 1.38e-11 | 0.012 | 45.97 |
| MFI | CD8 on CD39+ CD8br | rs35574803 | T | C | 0.658 | 0.053 | 3.42e-35 | 0.041 | 156.41 |
| MFI | CD8 on CD39+ CD8br | rs551832918 | A | G | 0.662 | 0.104 | 2.27e-10 | 0.011 | 40.41 |
| MFI | CD8 on CD39+ CD8br | rs564347773 | G | A | 0.526 | 0.078 | 1.92e-11 | 0.012 | 45.31 |
| MFI | CD8 on CD39+ CD8br | rs75918255 | G | A | 0.418 | 0.067 | 4.84e-10 | 0.011 | 38.93 |
| MFI | CD8 on CM CD8br | rs1006368 | C | T | 0.389 | 0.04 | 5.08e-22 | 0.029 | 94.37 |
| MFI | CD8 on CM CD8br | rs10919543 | A | G | -0.151 | 0.027 | 3.03e-08 | 0.010 | 30.83 |
| MFI | CD8 on CM CD8br | rs9912354 | C | T | 0.161 | 0.027 | 2.35e-09 | 0.011 | 35.86 |
| MFI | CD8 on TD CD8br | rs2394447 | C | T | 0.257 | 0.044 | 4.72e-09 | 0.021 | 34.62 |
| MFI | CD8 on TD CD8br | rs9270591 | C | T | -0.566 | 0.038 | 2.93e-48 | 0.122 | 227.37 |
| MFI | CD8 on naive CD8br | rs140308479 | T | G | 0.613 | 0.102 | 1.86e-09 | 0.010 | 36.26 |
| MFI | CD8 on naive CD8br | rs4832054 | G | A | 0.173 | 0.027 | 2.21e-10 | 0.012 | 40.48 |
| MFI | CD8 on naive CD8br | rs7559619 | A | G | -0.203 | 0.025 | 3.81e-16 | 0.019 | 66.9 |
| MFI | CD8 on naive CD8br | rs77745909 | A | G | 0.754 | 0.109 | 4.94e-12 | 0.014 | 48 |
| MFI | CD8 on naive CD8br | rs79815527 | G | A | 0.275 | 0.046 | 3.15e-09 | 0.010 | 35.26 |
| MFI | CD80 on CD62L+ plasmacytoid DC | rs111436326 | C | T | 0.412 | 0.069 | 2.63e-09 | 0.010 | 35.6 |
| MFI | CD80 on CD62L+ plasmacytoid DC | rs139376787 | G | A | -0.312 | 0.048 | 6.85e-11 | 0.012 | 42.81 |
| MFI | CD80 on CD62L+ plasmacytoid DC | rs148765873 | T | C | -0.35 | 0.063 | 2.69e-08 | 0.009 | 31.04 |
| MFI | CD80 on CD62L+ plasmacytoid DC | rs1801274 | A | G | 0.529 | 0.025 | 1.29e-90 | 0.106 | 431.12 |
| MFI | CD80 on CD62L+ plasmacytoid DC | rs183974458 | C | T | 0.549 | 0.066 | 1.22e-16 | 0.019 | 69.22 |
| MFI | CD80 on CD62L+ plasmacytoid DC | rs34658867 | A | C | 0.404 | 0.061 | 3.03e-11 | 0.012 | 44.41 |
| MFI | CD80 on CD62L+ plasmacytoid DC | rs6687275 | T | G | 0.339 | 0.028 | 7.56e-33 | 0.039 | 145.25 |
| MFI | CD80 on CD62L+ plasmacytoid DC | rs745307 | G | A | 0.709 | 0.033 | 5.03e-94 | 0.110 | 448.62 |
| MFI | CD80 on myeloid DC | rs115797102 | C | T | -0.709 | 0.12 | 3.17e-09 | 0.010 | 35.22 |
| MFI | CD80 on myeloid DC | rs12712610 | A | G | -0.182 | 0.028 | 5.43e-11 | 0.012 | 43.27 |
| MFI | CD80 on myeloid DC | rs74830391 | C | T | 0.2 | 0.036 | 4.14e-08 | 0.009 | 30.19 |
| MFI | CD80 on myeloid DC | rs77745909 | A | G | -0.716 | 0.117 | 1.02e-09 | 0.011 | 37.5 |
| MFI | CD80 on myeloid DC | rs7893324 | T | C | 0.282 | 0.035 | 1.37e-15 | 0.018 | 64.36 |
| MFI | CD80 on plasmacytoid DC | rs149192849 | T | C | -0.311 | 0.055 | 1.35e-08 | 0.009 | 32.39 |
| MFI | CD80 on plasmacytoid DC | rs4822134 | T | C | 0.147 | 0.025 | 6.78e-09 | 0.009 | 33.74 |
| MFI | CD80 on plasmacytoid DC | rs73165129 | C | T | -0.529 | 0.041 | 4.96e-38 | 0.044 | 169.97 |
| MFI | CD86 on granulocyte | rs11086984 | C | T | 0.276 | 0.035 | 2.23e-15 | 0.017 | 63.39 |
| MFI | CD86 on granulocyte | rs144642376 | A | G | 0.278 | 0.049 | 1.69e-08 | 0.009 | 31.93 |
| MFI | CD86 on granulocyte | rs147714617 | C | T | 0.299 | 0.037 | 1.93e-15 | 0.017 | 63.64 |
| MFI | CD86 on granulocyte | rs149803677 | A | C | 0.493 | 0.069 | 8.07e-13 | 0.014 | 51.59 |
| MFI | CD86 on granulocyte | rs162076 | G | A | -0.164 | 0.025 | 8.52e-11 | 0.011 | 42.34 |
| MFI | CD86 on granulocyte | rs1801274 | A | G | -0.293 | 0.026 | 2.48e-28 | 0.033 | 123.83 |
| MFI | CD86 on granulocyte | rs25680 | G | A | -0.315 | 0.033 | 3.76e-21 | 0.024 | 90.18 |
| MFI | CD86 on granulocyte | rs34101571 | G | A | -0.536 | 0.08 | 2.10e-11 | 0.012 | 45.14 |
| MFI | CD86 on granulocyte | rs35574803 | T | C | 0.7 | 0.053 | 1.02e-38 | 0.045 | 173.24 |
| MFI | CD86 on granulocyte | rs529513098 | T | G | 0.608 | 0.1 | 1.33e-09 | 0.010 | 36.96 |
| MFI | CD86 on granulocyte | rs551832918 | A | G | 0.731 | 0.105 | 4.27e-12 | 0.013 | 48.28 |
| MFI | CD86 on granulocyte | rs564347773 | G | A | 0.609 | 0.079 | 1.64e-14 | 0.016 | 59.38 |
| MFI | CD86 on granulocyte | rs569077190 | G | A | 0.319 | 0.055 | 7.71e-09 | 0.009 | 33.48 |
| MFI | CX3CR1 on CD14+ CD16+ monocyte | rs1723018 | A | G | 0.424 | 0.026 | 9.84e-58 | 0.084 | 268.01 |
| MFI | CX3CR1 on CD14+ CD16+ monocyte | rs61814886 | G | A | 0.254 | 0.043 | 4.21e-09 | 0.012 | 34.72 |
| MFI | CX3CR1 on CD14+ CD16+ monocyte | rs858545 | A | C | -0.168 | 0.027 | 5.71e-10 | 0.013 | 38.65 |
| MFI | CX3CR1 on CD14- CD16+ monocyte | rs17664049 | C | T | -0.661 | 0.12 | 3.96e-08 | 0.010 | 30.3 |
| MFI | CX3CR1 on CD14- CD16+ monocyte | rs2480679 | A | G | 0.229 | 0.041 | 2.08e-08 | 0.011 | 31.58 |
| MFI | CX3CR1 on CD14- CD16+ monocyte | rs2988279 | G | A | 0.337 | 0.027 | 8.20e-35 | 0.051 | 155.43 |
| MFI | CX3CR1 on CD14- CD16- | rs10501383 | C | T | -0.255 | 0.041 | 5.41e-10 | 0.010 | 38.7 |
| MFI | CX3CR1 on CD14- CD16- | rs11751198 | G | A | 0.2 | 0.036 | 4.32e-08 | 0.008 | 30.12 |
| MFI | CX3CR1 on CD14- CD16- | rs144398648 | C | T | 0.754 | 0.119 | 2.57e-10 | 0.011 | 40.18 |
| MFI | CX3CR1 on CD14- CD16- | rs55809481 | T | C | -0.242 | 0.029 | 5.44e-17 | 0.019 | 70.85 |
| MFI | HLA DR on HLA DR+ CD8br | rs35587265 | A | G | -0.473 | 0.059 | 1.64e-15 | 0.022 | 64.13 |
| MFI | HLA DR on HLA DR+ CD8br | rs61802333 | G | T | 0.312 | 0.028 | 1.13e-28 | 0.042 | 126.09 |
| MFI | HLA DR on HLA DR+ CD8br | rs6687275 | T | G | 0.233 | 0.031 | 1.56e-13 | 0.019 | 54.99 |
| MFI | HLA DR on HLA DR+ T cell | rs12138291 | G | A | -0.213 | 0.038 | 2.86e-08 | 0.011 | 30.96 |
| MFI | HLA DR on HLA DR+ T cell | rs1617988 | T | C | 0.428 | 0.026 | 1.39e-57 | 0.084 | 267.29 |
| MFI | HLA DR on HLA DR+ T cell | rs4657649 | T | C | 0.362 | 0.061 | 3.65e-09 | 0.012 | 34.99 |
| MFI | HLA DR on B cell | rs140684757 | C | T | 0.451 | 0.074 | 1.04e-09 | 0.010 | 37.43 |
| MFI | HLA DR on B cell | rs147487472 | C | A | 0.419 | 0.062 | 1.20e-11 | 0.012 | 46.23 |
| MFI | HLA DR on B cell | rs62626323 | C | A | 0.543 | 0.04 | 1.25e-40 | 0.048 | 182.46 |
| MFI | HLA DR on CD14+ monocyte | rs148451781 | A | C | -0.394 | 0.058 | 1.11e-11 | 0.013 | 46.41 |
| MFI | HLA DR on CD14+ monocyte | rs3793662 | C | T | -0.17 | 0.029 | 3.69e-09 | 0.009 | 34.96 |
| MFI | HLA DR on CD14+ monocyte | rs41294937 | T | C | 0.573 | 0.039 | 9.24e-48 | 0.056 | 216.82 |
| MFI | HLA DR on CD14+ monocyte | rs41295123 | G | A | -0.341 | 0.057 | 2.37e-09 | 0.010 | 35.8 |
| MFI | HLA DR on CD14+ monocyte | rs41295351 | G | A | -0.517 | 0.088 | 3.89e-09 | 0.009 | 34.83 |
| MFI | HLA DR on CD14+ monocyte | rs7078614 | G | T | 0.218 | 0.025 | 9.24e-18 | 0.020 | 74.42 |
| MFI | HLA DR on CD14- CD16- | rs130071 | G | A | 2.545 | 0.413 | 7.93e-10 | 0.011 | 37.97 |
| MFI | HLA DR on CD14- CD16- | rs17212021 | T | C | -2.715 | 0.473 | 1.03e-08 | 0.010 | 32.93 |
| MFI | HLA DR on CD14- CD16- | rs1736913 | T | C | 2.095 | 0.367 | 1.26e-08 | 0.009 | 32.53 |
| MFI | HLA DR on CD14- CD16- | rs450175 | A | C | -1.704 | 0.307 | 3.08e-08 | 0.009 | 30.77 |
| MFI | HLA DR on CD14- CD16- | rs58905133 | A | G | 3.541 | 0.318 | 2.91e-28 | 0.035 | 123.69 |
| MFI | HLA DR on CD33- HLA DR+ | rs111983490 | G | A | -0.217 | 0.037 | 3.74e-09 | 0.010 | 34.91 |
| MFI | HLA DR on CD33- HLA DR+ | rs28584364 | C | T | 0.312 | 0.051 | 1.43e-09 | 0.011 | 36.82 |
| MFI | HLA DR on CD33- HLA DR+ | rs6729180 | C | T | 0.202 | 0.026 | 1.78e-14 | 0.017 | 59.23 |
| MFI | HLA DR on CD33- HLA DR+ | rs6755452 | T | G | 0.19 | 0.032 | 4.75e-09 | 0.010 | 34.46 |
| MFI | HLA DR on CD33dim HLA DR+ CD11b+ | rs117147408 | A | G | 0.482 | 0.079 | 1.47e-09 | 0.011 | 36.76 |
| MFI | HLA DR on CD33dim HLA DR+ CD11b+ | rs12986962 | A | G | -0.16 | 0.028 | 1.54e-08 | 0.009 | 32.13 |
| MFI | HLA DR on CD33dim HLA DR+ CD11b+ | rs144837535 | T | C | 0.483 | 0.073 | 4.34e-11 | 0.013 | 43.72 |
| MFI | HLA DR on CD33dim HLA DR+ CD11b+ | rs150386792 | G | A | 0.502 | 0.077 | 6.77e-11 | 0.012 | 42.83 |
| MFI | HLA DR on CD33dim HLA DR+ CD11b+ | rs181878688 | G | A | 0.582 | 0.07 | 1.18e-16 | 0.020 | 69.31 |
| MFI | HLA DR on CD33dim HLA DR+ CD11b+ | rs186978368 | C | T | 0.46 | 0.075 | 7.74e-10 | 0.011 | 38.02 |
| MFI | HLA DR on CD33dim HLA DR+ CD11b+ | rs4796089 | T | C | -0.222 | 0.038 | 3.91e-09 | 0.010 | 34.81 |
| MFI | HLA DR on CD33dim HLA DR+ CD11b+ | rs62432266 | G | T | 0.309 | 0.054 | 1.25e-08 | 0.009 | 32.54 |
| MFI | HLA DR on CD33dim HLA DR+ CD11b- | rs112755578 | A | G | -0.511 | 0.049 | 3.75e-25 | 0.029 | 108.92 |
| MFI | HLA DR on CD33dim HLA DR+ CD11b- | rs116275309 | G | A | 0.843 | 0.11 | 2.16e-14 | 0.016 | 58.86 |
| MFI | HLA DR on CD33dim HLA DR+ CD11b- | rs145828126 | G | A | -0.336 | 0.059 | 1.09e-08 | 0.009 | 32.81 |
| MFI | HLA DR on CD33dim HLA DR+ CD11b- | rs149020192 | C | T | 0.604 | 0.083 | 5.07e-13 | 0.014 | 52.54 |
| MFI | HLA DR on CD33dim HLA DR+ CD11b- | rs150145799 | T | C | 0.775 | 0.036 | 8.90e-99 | 0.116 | 473.25 |
| MFI | HLA DR on CD33dim HLA DR+ CD11b- | rs1801274 | A | G | 0.429 | 0.026 | 1.45e-59 | 0.071 | 274.63 |
| MFI | HLA DR on CD33dim HLA DR+ CD11b- | rs181465742 | G | T | 0.594 | 0.1 | 2.69e-09 | 0.010 | 35.56 |
| MFI | HLA DR on CD33dim HLA DR+ CD11b- | rs188268415 | C | T | -0.601 | 0.059 | 4.30e-24 | 0.028 | 103.92 |
| MFI | HLA DR on CD33dim HLA DR+ CD11b- | rs189981470 | T | C | 0.674 | 0.105 | 1.39e-10 | 0.011 | 41.41 |
| MFI | HLA DR on CD33dim HLA DR+ CD11b- | rs190362224 | C | T | -0.722 | 0.11 | 5.89e-11 | 0.012 | 43.11 |
| MFI | HLA DR on CD33dim HLA DR+ CD11b- | rs193176459 | A | G | 0.935 | 0.113 | 2.00e-16 | 0.018 | 68.23 |
| MFI | HLA DR on CD33dim HLA DR+ CD11b- | rs587655195 | T | C | -0.725 | 0.114 | 2.51e-10 | 0.011 | 40.26 |
| MFI | HLA DR on CD33dim HLA DR+ CD11b- | rs587759315 | G | T | -0.712 | 0.118 | 2.13e-09 | 0.010 | 36.04 |
| MFI | HLA DR on CD33dim HLA DR+ CD11b- | rs587760652 | A | G | 0.725 | 0.127 | 1.36e-08 | 0.009 | 32.35 |
| MFI | HLA DR on CD33dim HLA DR+ CD11b- | rs604983 | G | A | 0.632 | 0.08 | 3.41e-15 | 0.017 | 62.53 |
| MFI | HLA DR on CD33dim HLA DR+ CD11b- | rs80178684 | A | G | -0.641 | 0.117 | 4.21e-08 | 0.008 | 30.14 |
| MFI | HLA DR on HSC | rs145567900 | C | A | -0.769 | 0.119 | 1.22e-10 | 0.011 | 41.66 |
| MFI | HLA DR on HSC | rs548982606 | C | T | -0.526 | 0.089 | 4.35e-09 | 0.009 | 34.6 |
| MFI | HLA DR on HSC | rs574746346 | G | A | -0.933 | 0.083 | 1.06e-28 | 0.033 | 125.59 |
| MFI | HVEM on CM CD8br | rs1375493 | G | A | 0.152 | 0.025 | 9.92e-10 | 0.010 | 37.52 |
| MFI | HVEM on CM CD8br | rs2418135 | G | A | -0.149 | 0.024 | 1.16e-09 | 0.010 | 37.18 |
| MFI | HVEM on CM CD8br | rs445 | C | T | -0.182 | 0.03 | 1.36e-09 | 0.010 | 36.89 |
| MFI | HVEM on T cell | rs112266065 | C | T | -1.037 | 0.115 | 3.87e-19 | 0.022 | 80.71 |
| MFI | HVEM on T cell | rs114919524 | A | C | -1.128 | 0.107 | 1.18e-25 | 0.030 | 111.28 |
| MFI | HVEM on T cell | rs117119882 | C | A | -0.923 | 0.116 | 1.98e-15 | 0.017 | 63.57 |
| MFI | HVEM on T cell | rs117193272 | T | C | -1.486 | 0.034 | 1.00e-200 | 0.344 | 1912.53 |
| MFI | HVEM on T cell | rs141059303 | G | A | -0.653 | 0.117 | 2.69e-08 | 0.008 | 31.05 |
| MFI | HVEM on T cell | rs144365928 | A | G | -0.696 | 0.078 | 5.38e-19 | 0.021 | 80.1 |
| MFI | HVEM on T cell | rs148516965 | C | T | -0.563 | 0.056 | 2.03e-23 | 0.027 | 100.73 |
| MFI | HVEM on T cell | rs149167072 | A | G | 0.338 | 0.053 | 2.93e-10 | 0.011 | 39.91 |
| MFI | HVEM on T cell | rs149806587 | A | G | -0.516 | 0.057 | 3.84e-19 | 0.022 | 80.78 |
| MFI | HVEM on T cell | rs150288192 | C | T | -1.235 | 0.117 | 8.11e-26 | 0.030 | 111.93 |
| MFI | HVEM on T cell | rs185098316 | G | A | -0.308 | 0.052 | 4.69e-09 | 0.009 | 34.46 |
| MFI | HVEM on T cell | rs185430947 | A | G | -1.005 | 0.108 | 3.27e-20 | 0.023 | 85.75 |
| MFI | HVEM on T cell | rs188216171 | G | A | -0.932 | 0.11 | 2.75e-17 | 0.019 | 72.17 |
| MFI | HVEM on T cell | rs189799494 | G | T | -0.648 | 0.051 | 6.32e-37 | 0.043 | 164.75 |
| MFI | HVEM on T cell | rs191363479 | C | T | -1.009 | 0.11 | 5.12e-20 | 0.023 | 84.86 |
| MFI | HVEM on T cell | rs193039847 | G | A | -1.381 | 0.087 | 2.55e-55 | 0.065 | 253.93 |
| MFI | HVEM on T cell | rs535059510 | C | T | -0.975 | 0.118 | 1.83e-16 | 0.018 | 68.44 |
| MFI | HVEM on T cell | rs539130256 | T | C | -0.612 | 0.106 | 7.09e-09 | 0.009 | 33.62 |
| MFI | HVEM on T cell | rs539241708 | C | T | -1.229 | 0.085 | 3.91e-46 | 0.054 | 209.04 |
| MFI | HVEM on T cell | rs556260593 | A | C | -1.029 | 0.106 | 4.22e-22 | 0.025 | 94.54 |
| MFI | HVEM on T cell | rs557577522 | G | A | -0.791 | 0.071 | 1.58e-28 | 0.033 | 124.78 |
| MFI | HVEM on T cell | rs566910078 | G | A | -1.271 | 0.11 | 1.41e-30 | 0.035 | 134.41 |
| MFI | HVEM on T cell | rs568374507 | C | T | -0.573 | 0.059 | 3.36e-22 | 0.025 | 95.07 |
| MFI | HVEM on T cell | rs5758649 | C | T | -0.59 | 0.045 | 1.09e-38 | 0.045 | 173.15 |
| MFI | HVEM on T cell | rs73888140 | G | A | -0.573 | 0.06 | 2.27e-21 | 0.024 | 91.18 |
| MFI | HVEM on T cell | rs78806227 | C | T | -0.949 | 0.12 | 4.62e-15 | 0.017 | 61.92 |
| MFI | HVEM on T cell | rs79085286 | T | C | -0.372 | 0.053 | 4.29e-12 | 0.013 | 48.26 |
| MFI | HVEM on TD CD8br | rs147487472 | C | A | 0.38 | 0.062 | 7.55e-10 | 0.010 | 38.06 |
| MFI | HVEM on TD CD8br | rs147829780 | C | T | 0.414 | 0.074 | 1.97e-08 | 0.009 | 31.66 |
| MFI | HVEM on TD CD8br | rs41294937 | T | C | 0.476 | 0.04 | 1.74e-31 | 0.037 | 138.81 |
| MFI | HVEM on naive CD4+ | rs11187166 | C | T | 0.176 | 0.032 | 2.97e-08 | 0.008 | 30.84 |
| MFI | HVEM on naive CD4+ | rs144398648 | C | T | 0.999 | 0.118 | 4.22e-17 | 0.019 | 71.33 |
| MFI | HVEM on naive CD4+ | rs192222877 | G | A | 0.629 | 0.109 | 8.82e-09 | 0.009 | 33.23 |
| MFI | HVEM on naive CD4+ | rs2070770 | C | T | -0.266 | 0.042 | 2.09e-10 | 0.011 | 40.58 |
| MFI | HVEM on naive CD4+ | rs55809481 | T | C | -0.319 | 0.028 | 9.01e-29 | 0.033 | 125.99 |
| MFI | HVEM on naive CD4+ | rs77008217 | C | T | 0.254 | 0.042 | 1.28e-09 | 0.010 | 37.02 |
| MFI | HVEM on naive CD4+ | rs909846 | T | G | 0.206 | 0.037 | 3.14e-08 | 0.008 | 30.74 |
| MFI | HVEM on naive CD4+ | rs9271146 | A | C | -0.189 | 0.03 | 6.09e-10 | 0.010 | 38.47 |
| MFI | HVEM on naive CD4+ | rs9469053 | A | G | 0.237 | 0.037 | 1.47e-10 | 0.011 | 41.28 |
| MFI | IgD on IgD+ CD38- unsw mem | rs147487472 | C | A | 0.424 | 0.061 | 6.12e-12 | 0.013 | 47.58 |
| MFI | IgD on IgD+ CD38- unsw mem | rs147829780 | C | T | 0.473 | 0.074 | 1.37e-10 | 0.011 | 41.41 |
| MFI | IgD on IgD+ CD38- unsw mem | rs3793662 | C | T | -0.165 | 0.029 | 2.19e-08 | 0.009 | 31.42 |
| MFI | IgD on IgD+ CD38- unsw mem | rs41294937 | T | C | 0.484 | 0.04 | 1.68e-32 | 0.038 | 143.61 |
| MFI | IgD on IgD+ CD38br | rs28424875 | A | C | -0.319 | 0.058 | 3.67e-08 | 0.011 | 30.46 |
| MFI | IgD on IgD+ CD38br | rs55971447 | C | T | -0.276 | 0.032 | 1.22e-17 | 0.025 | 74.02 |
| MFI | IgD on transitional | rs111758898 | T | C | 0.296 | 0.053 | 2.27e-08 | 0.009 | 31.36 |
| MFI | IgD on transitional | rs115536607 | C | T | 0.316 | 0.054 | 4.64e-09 | 0.009 | 34.49 |
| MFI | IgD on transitional | rs117147408 | A | G | 0.626 | 0.075 | 9.75e-17 | 0.019 | 69.64 |
| MFI | IgD on transitional | rs144837535 | T | C | 0.677 | 0.069 | 3.42e-22 | 0.025 | 95.03 |
| MFI | IgD on transitional | rs150386792 | G | A | 0.666 | 0.073 | 1.13e-19 | 0.022 | 83.27 |
| MFI | IgD on transitional | rs181878688 | G | A | 0.825 | 0.066 | 7.22e-35 | 0.041 | 154.89 |
| MFI | IgD on transitional | rs186978368 | C | T | 0.646 | 0.071 | 1.93e-19 | 0.022 | 82.2 |
| MFI | IgD on transitional | rs557204100 | A | G | 0.497 | 0.087 | 1.12e-08 | 0.009 | 32.76 |
| MFI | IgD on transitional | rs57245661 | A | C | 0.599 | 0.092 | 7.42e-11 | 0.012 | 42.63 |
| MFI | IgD on transitional | rs62432266 | G | T | 0.42 | 0.052 | 7.72e-16 | 0.018 | 65.5 |
| MFI | IgD on transitional | rs7569868 | C | T | 0.215 | 0.031 | 6.05e-12 | 0.013 | 47.6 |
| MFI | IgD on transitional | rs77291736 | C | T | -0.369 | 0.038 | 9.86e-22 | 0.025 | 92.87 |
| MFI | IgD on transitional | rs9916629 | T | C | 0.143 | 0.024 | 3.16e-09 | 0.010 | 35.23 |
| MFI | PDL-1 on CD14+ CD16- monocyte | rs11086984 | C | T | 0.199 | 0.035 | 1.25e-08 | 0.009 | 32.53 |
| MFI | PDL-1 on CD14+ CD16- monocyte | rs147714617 | C | T | 0.256 | 0.038 | 1.11e-11 | 0.013 | 46.41 |
| MFI | PDL-1 on CD14+ CD16- monocyte | rs25680 | G | A | -0.213 | 0.033 | 2.13e-10 | 0.011 | 40.55 |
| MFI | PDL-1 on CD14+ CD16- monocyte | rs35574803 | T | C | 0.671 | 0.053 | 1.49e-35 | 0.041 | 158.13 |
| MFI | PDL-1 on CD14+ CD16- monocyte | rs551832918 | A | G | 0.585 | 0.106 | 3.06e-08 | 0.008 | 30.75 |
| MFI | PDL-1 on CD14+ CD16- monocyte | rs56237381 | G | A | 0.429 | 0.077 | 2.95e-08 | 0.008 | 30.86 |
| MFI | PDL-1 on CD14+ CD16- monocyte | rs564347773 | G | A | 0.531 | 0.079 | 2.49e-11 | 0.012 | 44.78 |
| MFI | PDL-1 on CD14+ CD16- monocyte | rs72485816 | T | C | -0.409 | 0.072 | 1.77e-08 | 0.009 | 31.85 |
| MFI | PDL-1 on CD14+ CD16- monocyte | rs75918255 | G | A | 0.437 | 0.068 | 1.57e-10 | 0.011 | 41.17 |
| MFI | PDL-1 on CD14- CD16- | rs13191523 | A | G | 0.294 | 0.048 | 8.66e-10 | 0.012 | 37.82 |
| MFI | PDL-1 on CD14- CD16- | rs2105898 | T | G | -0.255 | 0.034 | 1.34e-13 | 0.017 | 55.25 |
| MFI | PDL-1 on CD14- CD16- | rs7745305 | T | C | 0.239 | 0.039 | 1.06e-09 | 0.012 | 37.43 |
| Morphological parameter | FSC-A on CD14+ monocyte | rs116844769 | G | A | 0.284 | 0.051 | 2.26e-08 | 0.009 | 31.39 |
| Morphological parameter | FSC-A on CD14+ monocyte | rs139370814 | C | T | 0.511 | 0.088 | 6.43e-09 | 0.009 | 33.84 |
| Morphological parameter | FSC-A on CD14+ monocyte | rs72635970 | T | G | 0.357 | 0.061 | 5.45e-09 | 0.009 | 34.17 |
| Morphological parameter | FSC-A on CD14+ monocyte | rs74053426 | G | A | -0.542 | 0.095 | 1.27e-08 | 0.009 | 32.5 |
| Morphological parameter | FSC-A on CD4+ | rs113013837 | A | G | 0.715 | 0.104 | 6.21e-12 | 0.013 | 47.54 |
| Morphological parameter | FSC-A on CD4+ | rs11643297 | A | G | 0.148 | 0.026 | 7.82e-09 | 0.009 | 33.48 |
| Morphological parameter | FSC-A on CD4+ | rs139732336 | C | T | 0.535 | 0.091 | 4.48e-09 | 0.009 | 34.56 |
| Morphological parameter | FSC-A on CD4+ | rs28739016 | T | G | 0.847 | 0.103 | 2.13e-16 | 0.018 | 68.03 |
| Morphological parameter | FSC-A on CD4+ | rs55712453 | A | G | 0.574 | 0.09 | 2.31e-10 | 0.011 | 40.39 |
| Morphological parameter | FSC-A on granulocyte | rs115251423 | G | A | 0.545 | 0.077 | 1.35e-12 | 0.016 | 50.64 |
| Morphological parameter | FSC-A on granulocyte | rs192633703 | C | T | 0.43 | 0.078 | 3.37e-08 | 0.010 | 30.61 |
| Morphological parameter | FSC-A on granulocyte | rs6721978 | T | C | 0.224 | 0.028 | 1.34e-15 | 0.020 | 64.48 |
| Morphological parameter | FSC-A on lymphocyte | rs11090614 | A | G | -0.305 | 0.039 | 8.45e-15 | 0.032 | 61.18 |
| Morphological parameter | FSC-A on lymphocyte | rs112266065 | C | T | -0.914 | 0.162 | 1.92e-08 | 0.017 | 31.83 |
| Morphological parameter | FSC-A on lymphocyte | rs114919524 | A | C | -1.226 | 0.164 | 1.28e-13 | 0.029 | 55.69 |
| Morphological parameter | FSC-A on lymphocyte | rs150612106 | G | A | -0.696 | 0.111 | 4.23e-10 | 0.021 | 39.36 |
| Morphological parameter | FSC-A on lymphocyte | rs185430947 | A | G | -1.042 | 0.157 | 4.05e-11 | 0.023 | 44.11 |
| Morphological parameter | FSC-A on lymphocyte | rs189799494 | G | T | -0.645 | 0.068 | 3.89e-21 | 0.047 | 91.15 |
| Morphological parameter | FSC-A on lymphocyte | rs193039847 | G | A | -1.367 | 0.117 | 1.88e-30 | 0.069 | 136.13 |
| Morphological parameter | FSC-A on lymphocyte | rs539241708 | C | T | -1.187 | 0.114 | 1.15e-24 | 0.056 | 108.11 |
| Morphological parameter | FSC-A on lymphocyte | rs556260593 | A | C | -1.188 | 0.145 | 5.33e-16 | 0.035 | 66.78 |
| Morphological parameter | FSC-A on lymphocyte | rs566910078 | G | A | -1.308 | 0.168 | 1.02e-14 | 0.032 | 60.77 |
| Morphological parameter | FSC-A on lymphocyte | rs568374507 | C | T | -0.598 | 0.078 | 2.95e-14 | 0.031 | 58.63 |
| Morphological parameter | FSC-A on lymphocyte | rs5758649 | C | T | -0.621 | 0.06 | 1.02e-24 | 0.056 | 108.34 |
| Morphological parameter | FSC-A on lymphocyte | rs73165129 | C | T | -1.495 | 0.047 | 9.85e-175 | 0.351 | 992.86 |
| Morphological parameter | FSC-A on lymphocyte | rs73888140 | G | A | -0.542 | 0.08 | 1.69e-11 | 0.024 | 45.83 |
| Morphological parameter | FSC-A on myeloid DC | rs12554596 | A | G | -0.156 | 0.025 | 8.43e-10 | 0.010 | 37.87 |
| Morphological parameter | FSC-A on myeloid DC | rs2066399 | G | A | 0.187 | 0.032 | 6.08e-09 | 0.009 | 33.94 |
| Morphological parameter | FSC-A on myeloid DC | rs2070770 | C | T | -0.257 | 0.042 | 7.49e-10 | 0.010 | 38.07 |
| Morphological parameter | FSC-A on myeloid DC | rs30003 | T | C | -0.195 | 0.029 | 3.22e-11 | 0.012 | 44.3 |
| Morphological parameter | SSC-A on B cell | rs112266065 | C | T | -1.054 | 0.115 | 1.07e-19 | 0.022 | 83.37 |
| Morphological parameter | SSC-A on B cell | rs114919524 | A | C | -1.122 | 0.107 | 2.41e-25 | 0.029 | 109.9 |
| Morphological parameter | SSC-A on B cell | rs117119882 | C | A | -0.958 | 0.116 | 1.82e-16 | 0.018 | 68.39 |
| Morphological parameter | SSC-A on B cell | rs144365928 | A | G | -0.69 | 0.078 | 1.19e-18 | 0.021 | 78.5 |
| Morphological parameter | SSC-A on B cell | rs148516965 | C | T | -0.554 | 0.056 | 1.10e-22 | 0.026 | 97.33 |
| Morphological parameter | SSC-A on B cell | rs149167072 | A | G | 0.353 | 0.053 | 4.83e-11 | 0.012 | 43.48 |
| Morphological parameter | SSC-A on B cell | rs149806587 | A | G | -0.525 | 0.058 | 1.02e-19 | 0.022 | 83.48 |
| Morphological parameter | SSC-A on B cell | rs184205740 | C | T | -1.186 | 0.116 | 4.97e-24 | 0.028 | 103.58 |
| Morphological parameter | SSC-A on B cell | rs185098316 | G | A | -0.298 | 0.052 | 1.55e-08 | 0.009 | 32.12 |
| Morphological parameter | SSC-A on B cell | rs185430947 | A | G | -0.967 | 0.109 | 9.11e-19 | 0.021 | 79.01 |
| Morphological parameter | SSC-A on B cell | rs188216171 | G | A | -0.955 | 0.11 | 4.81e-18 | 0.020 | 75.73 |
| Morphological parameter | SSC-A on B cell | rs189799494 | G | T | -0.687 | 0.05 | 3.33e-41 | 0.048 | 185.2 |
| Morphological parameter | SSC-A on B cell | rs191363479 | C | T | -0.967 | 0.11 | 1.94e-18 | 0.021 | 77.6 |
| Morphological parameter | SSC-A on B cell | rs193039847 | G | A | -1.352 | 0.087 | 5.84e-53 | 0.062 | 242.03 |
| Morphological parameter | SSC-A on B cell | rs4657041 | T | C | -0.152 | 0.027 | 1.93e-08 | 0.009 | 31.67 |
| Morphological parameter | SSC-A on B cell | rs532866318 | T | G | -1.358 | 0.12 | 3.05e-29 | 0.034 | 128.21 |
| Morphological parameter | SSC-A on B cell | rs535059510 | C | T | -1.017 | 0.118 | 9.25e-18 | 0.020 | 74.49 |
| Morphological parameter | SSC-A on B cell | rs539241708 | C | T | -1.176 | 0.085 | 3.20e-42 | 0.049 | 190.01 |
| Morphological parameter | SSC-A on B cell | rs556260593 | A | C | -0.997 | 0.106 | 8.63e-21 | 0.024 | 88.53 |
| Morphological parameter | SSC-A on B cell | rs557577522 | G | A | -0.772 | 0.071 | 3.39e-27 | 0.031 | 118.49 |
| Morphological parameter | SSC-A on B cell | rs568374507 | C | T | -0.622 | 0.059 | 7.39e-26 | 0.030 | 112.21 |
| Morphological parameter | SSC-A on B cell | rs5758649 | C | T | -0.64 | 0.045 | 4.41e-45 | 0.053 | 203.99 |
| Morphological parameter | SSC-A on B cell | rs73888140 | G | A | -0.622 | 0.06 | 7.72e-25 | 0.029 | 107.41 |
| Morphological parameter | SSC-A on B cell | rs74348413 | C | T | -1.363 | 0.034 | 1.00e-200 | 0.301 | 1573.62 |
| Morphological parameter | SSC-A on B cell | rs78806227 | C | T | -0.962 | 0.12 | 1.96e-15 | 0.017 | 63.66 |
| Morphological parameter | SSC-A on B cell | rs79085286 | T | C | -0.416 | 0.053 | 9.17e-15 | 0.016 | 60.54 |
| Morphological parameter | SSC-A on CD14+ monocyte | rs139249541 | A | G | 0.523 | 0.072 | 6.09e-13 | 0.014 | 52.16 |
| Morphological parameter | SSC-A on CD14+ monocyte | rs144837535 | T | C | 0.525 | 0.071 | 1.96e-13 | 0.015 | 54.41 |
| Morphological parameter | SSC-A on CD14+ monocyte | rs150386792 | G | A | 0.516 | 0.075 | 6.37e-12 | 0.013 | 47.49 |
| Morphological parameter | SSC-A on CD14+ monocyte | rs181878688 | G | A | 0.617 | 0.068 | 2.29e-19 | 0.022 | 81.85 |
| Morphological parameter | SSC-A on CD14+ monocyte | rs182904923 | T | G | 0.509 | 0.075 | 1.02e-11 | 0.013 | 46.59 |
| Morphological parameter | SSC-A on CD14+ monocyte | rs186978368 | C | T | 0.467 | 0.073 | 1.75e-10 | 0.011 | 40.94 |
| Morphological parameter | SSC-A on CD14+ monocyte | rs62432266 | G | T | 0.354 | 0.053 | 2.58e-11 | 0.012 | 44.73 |
| Morphological parameter | SSC-A on CD4+ | rs1264580 | G | A | 0.186 | 0.034 | 3.47e-08 | 0.008 | 30.55 |
| Morphological parameter | SSC-A on CD4+ | rs34256565 | T | C | -0.385 | 0.03 | 2.32e-36 | 0.042 | 161.94 |
| Morphological parameter | SSC-A on CD4+ | rs9270657 | T | G | -0.171 | 0.029 | 6.34e-09 | 0.009 | 33.88 |
| Morphological parameter | SSC-A on CD4+ | rs9461744 | A | G | 0.302 | 0.034 | 8.28e-19 | 0.021 | 79.24 |
| Morphological parameter | SSC-A on HLA DR+ NK | rs114968045 | T | C | -0.159 | 0.028 | 1.48e-08 | 0.009 | 32.23 |
| Morphological parameter | SSC-A on HLA DR+ NK | rs3104369 | T | C | 0.175 | 0.029 | 1.22e-09 | 0.011 | 37.13 |
| Morphological parameter | SSC-A on HLA DR+ NK | rs6729180 | C | T | 0.189 | 0.022 | 3.44e-17 | 0.021 | 71.77 |
| Morphological parameter | SSC-A on HLA DR+ T cell | rs10882701 | A | C | -0.412 | 0.025 | 3.85e-59 | 0.074 | 273.35 |
| Morphological parameter | SSC-A on HLA DR+ T cell | rs112358299 | G | A | 0.282 | 0.051 | 2.70e-08 | 0.009 | 31.05 |
| Morphological parameter | SSC-A on HLA DR+ T cell | rs118087712 | G | A | 0.29 | 0.04 | 7.54e-13 | 0.015 | 51.77 |
| Morphological parameter | SSC-A on HLA DR+ T cell | rs12778618 | T | C | 0.593 | 0.062 | 2.33e-21 | 0.026 | 91.22 |
| Morphological parameter | SSC-A on HLA DR+ T cell | rs184728966 | C | T | 0.933 | 0.096 | 6.44e-22 | 0.027 | 93.81 |
| Morphological parameter | SSC-A on HLA DR+ T cell | rs191819784 | G | A | 0.851 | 0.076 | 2.05e-28 | 0.035 | 124.39 |
| Morphological parameter | SSC-A on HLA DR+ T cell | rs3181131 | T | C | 1.014 | 0.019 | 1.00e-200 | 0.448 | 2764.44 |
| Morphological parameter | SSC-A on HLA DR+ T cell | rs61868672 | G | A | -0.394 | 0.051 | 1.54e-14 | 0.017 | 59.54 |
| Morphological parameter | SSC-A on HLA DR+ T cell | rs78114089 | G | A | -0.462 | 0.08 | 7.50e-09 | 0.010 | 33.55 |
| Morphological parameter | SSC-A on HLA DR+ T cell | rs7906684 | G | A | 1.186 | 0.08 | 4.30e-48 | 0.060 | 219.1 |
| Morphological parameter | SSC-A on NK | rs115536607 | C | T | 0.311 | 0.055 | 2.24e-08 | 0.008 | 31.4 |
| Morphological parameter | SSC-A on NK | rs117147408 | A | G | 0.569 | 0.078 | 3.23e-13 | 0.014 | 53.42 |
| Morphological parameter | SSC-A on NK | rs144837535 | T | C | 0.584 | 0.072 | 7.70e-16 | 0.018 | 65.48 |
| Morphological parameter | SSC-A on NK | rs150386792 | G | A | 0.635 | 0.076 | 6.84e-17 | 0.019 | 70.35 |
| Morphological parameter | SSC-A on NK | rs181878688 | G | A | 0.728 | 0.069 | 1.02e-25 | 0.030 | 111.52 |
| Morphological parameter | SSC-A on NK | rs186978368 | C | T | 0.58 | 0.074 | 5.32e-15 | 0.017 | 61.62 |
| Morphological parameter | SSC-A on NK | rs557204100 | A | G | 0.527 | 0.09 | 4.83e-09 | 0.009 | 34.4 |
| Morphological parameter | SSC-A on NK | rs57245661 | A | C | 0.574 | 0.095 | 1.61e-09 | 0.010 | 36.57 |
| Morphological parameter | SSC-A on NK | rs62432266 | G | T | 0.399 | 0.053 | 1.00e-13 | 0.015 | 55.74 |
| Morphological parameter | SSC-A on NK | rs7218011 | A | G | -0.187 | 0.034 | 3.82e-08 | 0.008 | 30.34 |
| Morphological parameter | SSC-A on NK | rs77738700 | G | A | -0.421 | 0.04 | 8.64e-26 | 0.030 | 111.87 |
| Morphological parameter | SSC-A on NK | rs9912354 | C | T | 0.144 | 0.025 | 9.10e-09 | 0.009 | 33.17 |
| Morphological parameter | SSC-A on plasmacytoid DC | rs10882675 | C | T | 0.817 | 0.023 | 1.00e-200 | 0.279 | 1314.74 |
| Morphological parameter | SSC-A on plasmacytoid DC | rs111534725 | G | T | 0.728 | 0.066 | 5.69e-28 | 0.035 | 122.29 |
| Morphological parameter | SSC-A on plasmacytoid DC | rs151158664 | C | T | 1.143 | 0.089 | 1.03e-36 | 0.046 | 164.03 |
| Morphological parameter | SSC-A on plasmacytoid DC | rs17111341 | T | C | 0.642 | 0.053 | 1.45e-33 | 0.042 | 148.9 |
| Morphological parameter | SSC-A on plasmacytoid DC | rs181391987 | G | A | -0.401 | 0.072 | 2.17e-08 | 0.009 | 31.46 |
| Morphological parameter | SSC-A on plasmacytoid DC | rs185198079 | C | T | 0.861 | 0.096 | 5.24e-19 | 0.023 | 80.24 |
| Morphological parameter | SSC-A on plasmacytoid DC | rs190891675 | C | T | 1.131 | 0.115 | 1.46e-22 | 0.028 | 96.83 |
| Morphological parameter | SSC-A on plasmacytoid DC | rs191459120 | G | T | 0.434 | 0.07 | 8.00e-10 | 0.011 | 37.96 |
| Morphological parameter | SSC-A on plasmacytoid DC | rs35302565 | T | C | 0.256 | 0.043 | 4.06e-09 | 0.010 | 34.76 |
| Morphological parameter | SSC-A on plasmacytoid DC | rs72812685 | G | A | 0.678 | 0.06 | 1.49e-29 | 0.037 | 129.79 |
| Morphological parameter | SSC-A on plasmacytoid DC | rs7906684 | G | A | 1.091 | 0.081 | 4.89e-40 | 0.050 | 179.89 |
| Relative count | Activated & resting Treg %CD4+ | rs11733310 | A | G | -0.429 | 0.074 | 8.26e-09 | 0.009 | 33.35 |
| Relative count | Activated & resting Treg %CD4+ | rs140380950 | T | C | -0.357 | 0.063 | 1.57e-08 | 0.009 | 32.1 |
| Relative count | Activated & resting Treg %CD4+ | rs80330977 | G | A | -0.258 | 0.044 | 6.16e-09 | 0.009 | 33.92 |
| Relative count | Activated & secreting Treg %CD4 Treg | rs112505169 | C | T | 0.685 | 0.106 | 1.22e-10 | 0.012 | 41.65 |
| Relative count | Activated & secreting Treg %CD4 Treg | rs12285327 | T | C | -0.228 | 0.042 | 4.61e-08 | 0.009 | 29.99 |
| Relative count | Activated & secreting Treg %CD4 Treg | rs142500247 | T | C | 0.508 | 0.075 | 1.13e-11 | 0.013 | 46.37 |
| Relative count | Activated & secreting Treg %CD4 Treg | rs408686 | C | A | -0.209 | 0.031 | 1.33e-11 | 0.013 | 46.03 |
| Relative count | Activated & secreting Treg %CD4 Treg | rs6751481 | T | C | 0.218 | 0.023 | 3.10e-20 | 0.025 | 85.91 |
| Relative count | Activated Treg %CD4 Treg | rs113075410 | A | G | 0.322 | 0.055 | 4.92e-09 | 0.009 | 34.36 |
| Relative count | Activated Treg %CD4 Treg | rs115536607 | C | T | 0.349 | 0.054 | 1.40e-10 | 0.011 | 41.38 |
| Relative count | Activated Treg %CD4 Treg | rs117147408 | A | G | 0.668 | 0.076 | 1.38e-18 | 0.021 | 78.22 |
| Relative count | Activated Treg %CD4 Treg | rs144837535 | T | C | 0.68 | 0.07 | 4.72e-22 | 0.025 | 94.37 |
| Relative count | Activated Treg %CD4 Treg | rs150386792 | G | A | 0.7 | 0.073 | 2.62e-21 | 0.024 | 90.88 |
| Relative count | Activated Treg %CD4 Treg | rs181878688 | G | A | 0.858 | 0.067 | 3.81e-37 | 0.043 | 165.76 |
| Relative count | Activated Treg %CD4 Treg | rs186978368 | C | T | 0.651 | 0.072 | 1.88e-19 | 0.022 | 82.25 |
| Relative count | Activated Treg %CD4 Treg | rs57245661 | A | C | 0.616 | 0.092 | 2.71e-11 | 0.012 | 44.63 |
| Relative count | Activated Treg %CD4 Treg | rs62432266 | G | T | 0.434 | 0.052 | 1.45e-16 | 0.019 | 68.85 |
| Relative count | Activated Treg %CD4 Treg | rs72956154 | A | G | 0.37 | 0.057 | 1.02e-10 | 0.011 | 42 |
| Relative count | Activated Treg %CD4 Treg | rs77738700 | G | A | -0.323 | 0.039 | 2.69e-16 | 0.018 | 67.59 |
| Relative count | Activated Treg %CD4 Treg | rs9916257 | G | T | 0.147 | 0.025 | 2.20e-09 | 0.010 | 35.93 |
| Relative count | B cell %lymphocyte | rs117519014 | C | A | 0.435 | 0.067 | 7.57e-11 | 0.014 | 42.66 |
| Relative count | B cell %lymphocyte | rs17190839 | G | A | -0.284 | 0.043 | 7.21e-11 | 0.014 | 42.74 |
| Relative count | B cell %lymphocyte | rs72847706 | T | C | -0.255 | 0.032 | 1.80e-15 | 0.021 | 63.95 |
| Relative count | B cell %lymphocyte | rs77488547 | T | C | -0.272 | 0.047 | 7.96e-09 | 0.011 | 33.47 |
| Relative count | B cell %lymphocyte | rs9357097 | C | T | 0.192 | 0.028 | 8.08e-12 | 0.016 | 47.1 |
| Relative count | CD11c+ monocyte %monocyte | rs11615628 | G | A | -0.322 | 0.031 | 1.21e-24 | 0.035 | 106.92 |
| Relative count | CD11c+ monocyte %monocyte | rs9272242 | T | C | 0.222 | 0.032 | 3.73e-12 | 0.016 | 48.64 |
| Relative count | CD11c+ monocyte %monocyte | rs9277770 | T | G | -0.208 | 0.032 | 1.44e-10 | 0.014 | 41.36 |
| Relative count | CD127- CD8br %CD8br | rs11882720 | T | G | -0.447 | 0.072 | 6.36e-10 | 0.024 | 38.64 |
| Relative count | CD127- CD8br %CD8br | rs3865444 | C | A | -1.223 | 0.036 | 9.19e-189 | 0.420 | 1139.95 |
| Relative count | CD127- CD8br %CD8br | rs558625031 | G | A | -0.721 | 0.085 | 4.46e-17 | 0.044 | 72.09 |
| Relative count | CD127- CD8br %CD8br | rs7351079 | A | G | -0.341 | 0.042 | 1.07e-15 | 0.040 | 65.54 |
| Relative count | CD127- CD8br %CD8br | rs78488761 | C | T | 0.411 | 0.071 | 9.88e-09 | 0.021 | 33.17 |
| Relative count | CD127- CD8br %CD8br | rs78949787 | C | T | 0.294 | 0.037 | 4.12e-15 | 0.038 | 62.81 |
| Relative count | CD14+ CD16- monocyte %monocyte | rs2949661 | C | T | 0.521 | 0.026 | 1.05e-84 | 0.123 | 406.25 |
| Relative count | CD14+ CD16- monocyte %monocyte | rs4657649 | T | C | 0.415 | 0.062 | 2.05e-11 | 0.015 | 45.26 |
| Relative count | CD14+ CD16- monocyte %monocyte | rs61814872 | C | T | 0.272 | 0.044 | 1.17e-09 | 0.013 | 37.23 |
| Relative count | CD14- CD16+ monocyte %monocyte | rs114672530 | G | A | -0.646 | 0.105 | 8.19e-10 | 0.013 | 37.92 |
| Relative count | CD14- CD16+ monocyte %monocyte | rs12138291 | G | A | -0.284 | 0.038 | 2.28e-13 | 0.018 | 54.22 |
| Relative count | CD14- CD16+ monocyte %monocyte | rs16848876 | A | G | -0.541 | 0.094 | 1.05e-08 | 0.011 | 32.92 |
| Relative count | CD14- CD16+ monocyte %monocyte | rs2949661 | C | T | 0.632 | 0.025 | 3.46e-127 | 0.179 | 635.88 |
| Relative count | CD14- CD16+ monocyte %monocyte | rs35055340 | C | T | -0.466 | 0.077 | 1.48e-09 | 0.012 | 36.77 |
| Relative count | CD14- CD16+ monocyte %monocyte | rs72703432 | A | G | -0.488 | 0.086 | 1.57e-08 | 0.011 | 32.12 |
| Relative count | CD16+ monocyte %monocyte | rs139249541 | A | G | 0.429 | 0.076 | 1.48e-08 | 0.009 | 32.22 |
| Relative count | CD16+ monocyte %monocyte | rs144837535 | T | C | 0.457 | 0.074 | 6.12e-10 | 0.011 | 38.49 |
| Relative count | CD16+ monocyte %monocyte | rs150386792 | G | A | 0.425 | 0.078 | 4.56e-08 | 0.009 | 30.02 |
| Relative count | CD16+ monocyte %monocyte | rs181878688 | G | A | 0.458 | 0.071 | 1.07e-10 | 0.012 | 41.92 |
| Relative count | CD16+ monocyte %monocyte | rs6755452 | T | G | 0.216 | 0.033 | 5.00e-11 | 0.012 | 43.43 |
| Relative count | CD16+ monocyte %monocyte | rs9896155 | A | G | 0.17 | 0.027 | 5.51e-10 | 0.011 | 38.71 |
| Relative count | CD20- %B cell | rs2250246 | A | G | 0.147 | 0.024 | 2.12e-09 | 0.010 | 36.02 |
| Relative count | CD20- %B cell | rs35574803 | T | C | -0.353 | 0.054 | 5.92e-11 | 0.012 | 43.07 |
| Relative count | CD25hi %CD4+ | rs115797102 | C | T | -0.657 | 0.119 | 3.45e-08 | 0.009 | 30.54 |
| Relative count | CD25hi %CD4+ | rs12712610 | A | G | -0.157 | 0.028 | 1.21e-08 | 0.009 | 32.61 |
| Relative count | CD25hi %CD4+ | rs61839660 | C | T | 0.41 | 0.055 | 1.38e-13 | 0.016 | 55.14 |
| Relative count | CD25hi %CD4+ | rs71479760 | C | A | -0.146 | 0.026 | 3.20e-08 | 0.009 | 30.69 |
| Relative count | CD25hi %CD4+ | rs74830391 | C | T | 0.199 | 0.036 | 3.87e-08 | 0.009 | 30.33 |
| Relative count | CD25hi %CD4+ | rs77745909 | A | G | -0.697 | 0.116 | 2.16e-09 | 0.010 | 36 |
| Relative count | CD25hi CD45RA+ CD4 not Treg %CD4+ | rs71632979 | A | G | 0.752 | 0.027 | 1.40e-150 | 0.183 | 756.36 |
| Relative count | CD25hi CD45RA+ CD4 not Treg %CD4+ | rs71639910 | G | A | 0.359 | 0.051 | 2.05e-12 | 0.014 | 49.78 |
| Relative count | CD25hi CD45RA+ CD4 not Treg %CD4+ | rs74341264 | A | G | 0.365 | 0.039 | 1.18e-20 | 0.025 | 87.9 |
| Relative count | CD25hi CD45RA+ CD4 not Treg %T cell | rs3865444 | C | A | -0.518 | 0.038 | 1.14e-40 | 0.092 | 187.01 |
| Relative count | CD25hi CD45RA+ CD4 not Treg %T cell | rs4499342 | C | T | 0.254 | 0.046 | 3.85e-08 | 0.016 | 30.45 |
| Relative count | CD25hi CD45RA+ CD4 not Treg %T cell | rs7351079 | A | G | -0.276 | 0.037 | 1.37e-13 | 0.029 | 55.54 |
| Relative count | CD28+ CD45RA- CD8dim %T cell | rs146491190 | A | G | -0.288 | 0.047 | 6.86e-10 | 0.011 | 38.26 |
| Relative count | CD28+ CD45RA- CD8dim %T cell | rs4082014 | G | A | -0.303 | 0.029 | 4.89e-25 | 0.031 | 108.43 |
| Relative count | CD28+ CD45RA- CD8dim %T cell | rs6535445 | T | C | -0.303 | 0.026 | 1.35e-31 | 0.039 | 139.48 |
| Relative count | CD28+ DN (CD4-CD8-) %DN | rs12212931 | T | G | 0.212 | 0.032 | 3.02e-11 | 0.012 | 44.41 |
| Relative count | CD28+ DN (CD4-CD8-) %DN | rs144398648 | C | T | 0.734 | 0.119 | 7.77e-10 | 0.010 | 38 |
| Relative count | CD28+ DN (CD4-CD8-) %DN | rs1941018 | A | G | -0.181 | 0.025 | 4.63e-13 | 0.014 | 52.74 |
| Relative count | CD28+ DN (CD4-CD8-) %DN | rs35139284 | C | T | 0.183 | 0.029 | 3.55e-10 | 0.011 | 39.55 |
| Relative count | CD28+ DN (CD4-CD8-) %DN | rs354026 | G | T | 0.2 | 0.029 | 1.12e-11 | 0.013 | 46.38 |
| Relative count | CD28- CD127- CD25++ CD8br %CD8br | rs149188558 | T | C | -0.469 | 0.081 | 6.81e-09 | 0.009 | 33.73 |
| Relative count | CD28- CD127- CD25++ CD8br %CD8br | rs189779608 | G | A | -0.575 | 0.102 | 1.80e-08 | 0.009 | 31.79 |
| Relative count | CD28- CD127- CD25++ CD8br %CD8br | rs4683184 | G | A | 0.245 | 0.026 | 1.17e-20 | 0.024 | 87.82 |
| Relative count | CD28- CD127- CD25++ CD8br %CD8br | rs79825014 | G | T | -0.416 | 0.068 | 1.02e-09 | 0.010 | 37.46 |
| Relative count | CD28- CD127- CD25++ CD8br %T cell | rs115383270 | G | A | 0.257 | 0.033 | 4.42e-15 | 0.017 | 62 |
| Relative count | CD28- CD127- CD25++ CD8br %T cell | rs1258266 | T | C | -2.317 | 0.424 | 4.85e-08 | 0.008 | 29.89 |
| Relative count | CD28- CD127- CD25++ CD8br %T cell | rs143052305 | C | T | 0.434 | 0.075 | 7.48e-09 | 0.009 | 33.55 |
| Relative count | CD28- CD127- CD25++ CD8br %T cell | rs7893879 | G | T | -2.162 | 0.389 | 3.02e-08 | 0.008 | 30.82 |
| Relative count | CD28- CD25++ CD8br %CD8br | rs10882659 | T | C | -0.223 | 0.027 | 3.43e-16 | 0.023 | 67.27 |
| Relative count | CD28- CD25++ CD8br %CD8br | rs12138291 | G | A | -0.259 | 0.038 | 1.41e-11 | 0.016 | 45.99 |
| Relative count | CD28- CD25++ CD8br %CD8br | rs142607615 | C | T | -0.582 | 0.094 | 8.21e-10 | 0.013 | 37.93 |
| Relative count | CD28- CD25++ CD8br %CD8br | rs16848876 | A | G | -0.518 | 0.093 | 2.91e-08 | 0.010 | 30.91 |
| Relative count | CD28- CD25++ CD8br %CD8br | rs2949661 | C | T | 0.58 | 0.025 | 5.30e-108 | 0.154 | 530.08 |
| Relative count | CD28- CD25++ CD8br %T cell | rs112266065 | C | T | -1.072 | 0.115 | 1.88e-20 | 0.023 | 87 |
| Relative count | CD28- CD25++ CD8br %T cell | rs114919524 | A | C | -1.136 | 0.107 | 3.92e-26 | 0.030 | 113.5 |
| Relative count | CD28- CD25++ CD8br %T cell | rs144365928 | A | G | -0.7 | 0.078 | 2.86e-19 | 0.022 | 81.4 |
| Relative count | CD28- CD25++ CD8br %T cell | rs148516965 | C | T | -0.565 | 0.056 | 1.14e-23 | 0.027 | 101.92 |
| Relative count | CD28- CD25++ CD8br %T cell | rs149167072 | A | G | 0.348 | 0.053 | 7.48e-11 | 0.012 | 42.62 |
| Relative count | CD28- CD25++ CD8br %T cell | rs184205740 | C | T | -1.185 | 0.116 | 3.96e-24 | 0.028 | 104.12 |
| Relative count | CD28- CD25++ CD8br %T cell | rs185098316 | G | A | -0.302 | 0.052 | 8.34e-09 | 0.009 | 33.34 |
| Relative count | CD28- CD25++ CD8br %T cell | rs185430947 | A | G | -0.998 | 0.108 | 4.98e-20 | 0.023 | 84.94 |
| Relative count | CD28- CD25++ CD8br %T cell | rs187065953 | T | C | -0.89 | 0.095 | 1.42e-20 | 0.023 | 87.46 |
| Relative count | CD28- CD25++ CD8br %T cell | rs188216171 | G | A | -0.967 | 0.109 | 1.32e-18 | 0.021 | 78.25 |
| Relative count | CD28- CD25++ CD8br %T cell | rs189799494 | G | T | -0.708 | 0.05 | 7.36e-44 | 0.051 | 198.01 |
| Relative count | CD28- CD25++ CD8br %T cell | rs191363479 | C | T | -0.998 | 0.109 | 1.11e-19 | 0.022 | 83.26 |
| Relative count | CD28- CD25++ CD8br %T cell | rs193039847 | G | A | -1.339 | 0.087 | 2.78e-52 | 0.061 | 238.77 |
| Relative count | CD28- CD25++ CD8br %T cell | rs532866318 | T | G | -1.368 | 0.119 | 7.36e-30 | 0.035 | 131.2 |
| Relative count | CD28- CD25++ CD8br %T cell | rs535059510 | C | T | -1.03 | 0.117 | 2.48e-18 | 0.021 | 77.06 |
| Relative count | CD28- CD25++ CD8br %T cell | rs539241708 | C | T | -1.165 | 0.085 | 1.08e-41 | 0.049 | 187.62 |
| Relative count | CD28- CD25++ CD8br %T cell | rs556260593 | A | C | -0.989 | 0.106 | 1.22e-20 | 0.023 | 87.7 |
| Relative count | CD28- CD25++ CD8br %T cell | rs557577522 | G | A | -0.767 | 0.071 | 4.87e-27 | 0.031 | 117.76 |
| Relative count | CD28- CD25++ CD8br %T cell | rs568374507 | C | T | -0.648 | 0.059 | 5.03e-28 | 0.032 | 122.38 |
| Relative count | CD28- CD25++ CD8br %T cell | rs5758649 | C | T | -0.665 | 0.045 | 7.62e-49 | 0.057 | 222.16 |
| Relative count | CD28- CD25++ CD8br %T cell | rs73165124 | C | T | -1.282 | 0.034 | 1.00e-200 | 0.285 | 1460.2 |
| Relative count | CD28- CD25++ CD8br %T cell | rs73888140 | G | A | -0.645 | 0.06 | 1.06e-26 | 0.031 | 116.16 |
| Relative count | CD28- CD25++ CD8br %T cell | rs78806227 | C | T | -0.958 | 0.12 | 1.96e-15 | 0.017 | 63.66 |
| Relative count | CD28- CD25++ CD8br %T cell | rs79085286 | T | C | -0.442 | 0.053 | 1.69e-16 | 0.018 | 68.53 |
| Relative count | CD28- CD8br %CD8br | rs10774625 | A | G | -0.163 | 0.029 | 1.29e-08 | 0.011 | 32.51 |
| Relative count | CD28- CD8br %CD8br | rs11615628 | G | A | -0.275 | 0.032 | 5.31e-18 | 0.025 | 75.7 |
| Relative count | CD28- CD8br %CD8br | rs11628611 | C | T | 0.17 | 0.028 | 1.47e-09 | 0.012 | 36.77 |
| Relative count | CD28- CD8br %CD8br | rs12298133 | C | T | 0.228 | 0.036 | 4.24e-10 | 0.013 | 39.23 |
| Relative count | CD28- CD8br %CD8br | rs28584364 | C | T | 0.364 | 0.052 | 5.07e-12 | 0.016 | 48.03 |
| Relative count | CD28- CD8dim %CD8dim | rs111797167 | A | G | 0.409 | 0.071 | 7.11e-09 | 0.009 | 33.64 |
| Relative count | CD28- CD8dim %CD8dim | rs144398648 | C | T | 0.75 | 0.119 | 3.20e-10 | 0.011 | 39.71 |
| Relative count | CD28- CD8dim %CD8dim | rs4447195 | C | T | -0.252 | 0.042 | 1.45e-09 | 0.010 | 36.77 |
| Relative count | CD28- CD8dim %CD8dim | rs6459417 | C | T | 0.228 | 0.037 | 7.25e-10 | 0.010 | 38.14 |
| Relative count | CD28- CD8dim %CD8dim | rs6509428 | C | T | -0.24 | 0.029 | 2.09e-16 | 0.018 | 68.12 |
| Relative count | CD28- CD8dim %T cell | rs115832613 | G | A | -0.786 | 0.119 | 4.41e-11 | 0.012 | 43.71 |
| Relative count | CD28- CD8dim %T cell | rs145567900 | C | A | -0.795 | 0.118 | 2.31e-11 | 0.012 | 44.95 |
| Relative count | CD28- CD8dim %T cell | rs574746346 | G | A | -1.028 | 0.082 | 5.25e-35 | 0.041 | 155.63 |
| Relative count | CD28- CD8dim %T cell | rs76354299 | G | A | -0.418 | 0.069 | 1.66e-09 | 0.010 | 36.51 |
| Relative count | CD28- DN (CD4-CD8-) %DN | rs113013837 | A | G | 0.721 | 0.103 | 2.91e-12 | 0.013 | 49.03 |
| Relative count | CD28- DN (CD4-CD8-) %DN | rs137965091 | G | A | 0.374 | 0.068 | 3.16e-08 | 0.008 | 30.72 |
| Relative count | CD28- DN (CD4-CD8-) %DN | rs150239512 | A | G | 0.543 | 0.093 | 6.10e-09 | 0.009 | 33.94 |
| Relative count | CD28- DN (CD4-CD8-) %DN | rs150816467 | T | C | 0.548 | 0.092 | 2.50e-09 | 0.010 | 35.7 |
| Relative count | CD28- DN (CD4-CD8-) %DN | rs28739016 | T | G | 0.804 | 0.102 | 4.48e-15 | 0.017 | 61.93 |
| Relative count | CD33br HLA DR+ CD14- %CD33br HLA DR+ | rs11086984 | C | T | 0.199 | 0.035 | 1.25e-08 | 0.009 | 32.52 |
| Relative count | CD33br HLA DR+ CD14- %CD33br HLA DR+ | rs144642376 | A | G | 0.271 | 0.049 | 3.82e-08 | 0.008 | 30.34 |
| Relative count | CD33br HLA DR+ CD14- %CD33br HLA DR+ | rs147714617 | C | T | 0.248 | 0.038 | 4.48e-11 | 0.012 | 43.64 |
| Relative count | CD33br HLA DR+ CD14- %CD33br HLA DR+ | rs1801274 | A | G | -0.159 | 0.027 | 3.13e-09 | 0.010 | 35.24 |
| Relative count | CD33br HLA DR+ CD14- %CD33br HLA DR+ | rs25680 | G | A | -0.261 | 0.033 | 6.60e-15 | 0.016 | 61.2 |
| Relative count | CD33br HLA DR+ CD14- %CD33br HLA DR+ | rs35574803 | T | C | 0.747 | 0.053 | 8.02e-44 | 0.051 | 197.83 |
| Relative count | CD33br HLA DR+ CD14- %CD33br HLA DR+ | rs529513098 | T | G | 0.611 | 0.1 | 1.07e-09 | 0.010 | 37.37 |
| Relative count | CD33br HLA DR+ CD14- %CD33br HLA DR+ | rs551832918 | A | G | 0.699 | 0.105 | 3.24e-11 | 0.012 | 44.27 |
| Relative count | CD33br HLA DR+ CD14- %CD33br HLA DR+ | rs564347773 | G | A | 0.622 | 0.079 | 5.81e-15 | 0.017 | 61.45 |
| Relative count | CD33br HLA DR+ CD14- %CD33br HLA DR+ | rs569077190 | G | A | 0.33 | 0.055 | 2.67e-09 | 0.010 | 35.57 |
| Relative count | CD33br HLA DR+ CD14- %CD33br HLA DR+ | rs709589 | C | T | -0.176 | 0.032 | 2.78e-08 | 0.008 | 30.98 |
| Relative count | CD33br HLA DR+ CD14- %CD33br HLA DR+ | rs75918255 | G | A | 0.509 | 0.068 | 9.62e-14 | 0.015 | 55.85 |
| Relative count | CD33dim HLA DR+ CD11b- %CD33dim HLA DR+ | rs116007826 | A | G | 0.8 | 0.06 | 3.14e-39 | 0.058 | 176.81 |
| Relative count | CD33dim HLA DR+ CD11b- %CD33dim HLA DR+ | rs11966070 | T | C | 0.275 | 0.042 | 4.82e-11 | 0.015 | 43.55 |
| Relative count | CD33dim HLA DR+ CD11b- %CD33dim HLA DR+ | rs185111 | A | G | 0.179 | 0.031 | 1.15e-08 | 0.011 | 32.75 |
| Relative count | CD33dim HLA DR+ CD11b- %CD33dim HLA DR+ | rs2858885 | C | T | -0.319 | 0.031 | 1.13e-24 | 0.036 | 106.99 |
| Relative count | CD33dim HLA DR+ CD11b- %CD33dim HLA DR+ | rs35525122 | C | A | 0.853 | 0.049 | 1.39e-65 | 0.097 | 307.86 |
| Relative count | CD33dim HLA DR+ CD11b- %CD33dim HLA DR+ | rs362522 | C | T | 0.307 | 0.041 | 5.34e-14 | 0.020 | 57.13 |
| Relative count | CD33dim HLA DR+ CD11b- %CD33dim HLA DR+ | rs55971447 | C | T | -0.48 | 0.031 | 5.94e-51 | 0.076 | 234.46 |
| Relative count | CD33dim HLA DR+ CD11b- %CD33dim HLA DR+ | rs6925683 | T | G | 0.292 | 0.043 | 1.95e-11 | 0.016 | 45.33 |
| Relative count | CD33dim HLA DR+ CD11b- %CD33dim HLA DR+ | rs9468543 | G | A | 0.704 | 0.082 | 1.76e-17 | 0.025 | 73.28 |
| Relative count | CD39+ CD8br %CD8br | rs183053322 | C | T | -0.393 | 0.066 | 3.83e-09 | 0.009 | 34.86 |
| Relative count | CD39+ CD8br %CD8br | rs7216096 | G | A | 0.202 | 0.033 | 6.55e-10 | 0.010 | 38.33 |
| Relative count | CD39+ CD8br %CD8br | rs9916257 | G | T | -0.2 | 0.024 | 8.82e-17 | 0.019 | 69.87 |
| Relative count | CD39+ activated Treg %activated Treg | rs139370814 | C | T | 0.554 | 0.088 | 3.25e-10 | 0.011 | 39.73 |
| Relative count | CD39+ activated Treg %activated Treg | rs45608038 | A | G | 0.353 | 0.062 | 1.71e-08 | 0.009 | 31.93 |
| Relative count | CD39+ activated Treg %activated Treg | rs74053426 | G | A | -0.555 | 0.095 | 5.88e-09 | 0.009 | 34.02 |
| Relative count | CD39+ resting Treg %resting Treg | rs2981595 | G | A | -0.169 | 0.03 | 1.26e-08 | 0.009 | 32.51 |
| Relative count | CD39+ resting Treg %resting Treg | rs3129765 | A | G | -0.231 | 0.033 | 5.45e-12 | 0.014 | 47.83 |
| Relative count | CD39+ resting Treg %resting Treg | rs7766452 | G | A | 0.19 | 0.035 | 4.60e-08 | 0.009 | 29.99 |
| Relative count | CD39+ secreting Treg %CD4 Treg | rs112266065 | C | T | -1.05 | 0.115 | 1.28e-19 | 0.022 | 83.03 |
| Relative count | CD39+ secreting Treg %CD4 Treg | rs114919524 | A | C | -1.132 | 0.107 | 6.38e-26 | 0.030 | 112.49 |
| Relative count | CD39+ secreting Treg %CD4 Treg | rs117119882 | C | A | -0.945 | 0.116 | 3.86e-16 | 0.018 | 66.85 |
| Relative count | CD39+ secreting Treg %CD4 Treg | rs117492772 | G | A | -1.304 | 0.033 | 1.00e-200 | 0.294 | 1523.44 |
| Relative count | CD39+ secreting Treg %CD4 Treg | rs141059303 | G | A | -0.655 | 0.117 | 2.30e-08 | 0.009 | 31.37 |
| Relative count | CD39+ secreting Treg %CD4 Treg | rs144365928 | A | G | -0.7 | 0.078 | 2.88e-19 | 0.022 | 81.38 |
| Relative count | CD39+ secreting Treg %CD4 Treg | rs148516965 | C | T | -0.572 | 0.056 | 4.05e-24 | 0.028 | 104.05 |
| Relative count | CD39+ secreting Treg %CD4 Treg | rs149167072 | A | G | 0.341 | 0.053 | 1.95e-10 | 0.011 | 40.71 |
| Relative count | CD39+ secreting Treg %CD4 Treg | rs149806587 | A | G | -0.529 | 0.057 | 4.63e-20 | 0.023 | 85.07 |
| Relative count | CD39+ secreting Treg %CD4 Treg | rs150288192 | C | T | -1.219 | 0.117 | 3.22e-25 | 0.029 | 109.24 |
| Relative count | CD39+ secreting Treg %CD4 Treg | rs185098316 | G | A | -0.301 | 0.052 | 9.35e-09 | 0.009 | 33.11 |
| Relative count | CD39+ secreting Treg %CD4 Treg | rs185430947 | A | G | -1.01 | 0.108 | 1.82e-20 | 0.023 | 86.93 |
| Relative count | CD39+ secreting Treg %CD4 Treg | rs188216171 | G | A | -0.942 | 0.11 | 1.20e-17 | 0.020 | 73.9 |
| Relative count | CD39+ secreting Treg %CD4 Treg | rs189799494 | G | T | -0.663 | 0.05 | 1.17e-38 | 0.045 | 173.01 |
| Relative count | CD39+ secreting Treg %CD4 Treg | rs191363479 | C | T | -1.014 | 0.109 | 3.08e-20 | 0.023 | 85.86 |
| Relative count | CD39+ secreting Treg %CD4 Treg | rs193039847 | G | A | -1.366 | 0.087 | 2.72e-54 | 0.064 | 248.67 |
| Relative count | CD39+ secreting Treg %CD4 Treg | rs532866318 | T | G | -1.382 | 0.12 | 2.35e-30 | 0.035 | 133.45 |
| Relative count | CD39+ secreting Treg %CD4 Treg | rs535059510 | C | T | -1 | 0.118 | 2.65e-17 | 0.019 | 72.27 |
| Relative count | CD39+ secreting Treg %CD4 Treg | rs539130256 | T | C | -0.581 | 0.105 | 3.87e-08 | 0.008 | 30.33 |
| Relative count | CD39+ secreting Treg %CD4 Treg | rs539241708 | C | T | -1.201 | 0.085 | 3.64e-44 | 0.052 | 199.58 |
| Relative count | CD39+ secreting Treg %CD4 Treg | rs556260593 | A | C | -1.023 | 0.106 | 6.31e-22 | 0.025 | 93.8 |
| Relative count | CD39+ secreting Treg %CD4 Treg | rs557577522 | G | A | -0.777 | 0.071 | 1.31e-27 | 0.032 | 120.45 |
| Relative count | CD39+ secreting Treg %CD4 Treg | rs568374507 | C | T | -0.592 | 0.059 | 1.11e-23 | 0.027 | 101.97 |
| Relative count | CD39+ secreting Treg %CD4 Treg | rs5758649 | C | T | -0.602 | 0.045 | 3.37e-40 | 0.047 | 180.39 |
| Relative count | CD39+ secreting Treg %CD4 Treg | rs73888140 | G | A | -0.595 | 0.06 | 5.73e-23 | 0.026 | 98.65 |
| Relative count | CD39+ secreting Treg %CD4 Treg | rs78806227 | C | T | -0.96 | 0.12 | 1.91e-15 | 0.017 | 63.69 |
| Relative count | CD39+ secreting Treg %CD4 Treg | rs79085286 | T | C | -0.386 | 0.053 | 5.53e-13 | 0.014 | 52.36 |
| Relative count | CD4 Treg %T cell | rs115383270 | G | A | 0.509 | 0.032 | 3.53e-55 | 0.066 | 253.26 |
| Relative count | CD4 Treg %T cell | rs56391246 | C | T | 0.262 | 0.044 | 4.29e-09 | 0.010 | 34.63 |
| Relative count | CD4 Treg %T cell | rs876036 | T | C | 0.166 | 0.028 | 5.07e-09 | 0.009 | 34.33 |
| Relative count | CD4+ %T cell | rs146866569 | C | T | 0.534 | 0.085 | 3.22e-10 | 0.011 | 39.73 |
| Relative count | CD4+ %T cell | rs71632979 | A | G | -0.64 | 0.027 | 1.56e-113 | 0.132 | 550.72 |
| Relative count | CD4+ %T cell | rs71639910 | G | A | -0.306 | 0.048 | 2.25e-10 | 0.011 | 40.44 |
| Relative count | CD4+ %T cell | rs74341264 | A | G | -0.246 | 0.038 | 5.98e-11 | 0.012 | 43.05 |
| Relative count | CD45RA+ CD28- CD8br %T cell | rs115180511 | T | C | -0.713 | 0.127 | 2.10e-08 | 0.011 | 31.57 |
| Relative count | CD45RA+ CD28- CD8br %T cell | rs138284624 | C | T | -0.752 | 0.081 | 3.17e-20 | 0.029 | 86.1 |
| Relative count | CD45RA+ CD28- CD8br %T cell | rs139132726 | C | T | -0.659 | 0.114 | 9.40e-09 | 0.011 | 33.16 |
| Relative count | CD45RA+ CD28- CD8br %T cell | rs142587417 | G | A | -0.81 | 0.117 | 5.08e-12 | 0.016 | 48.01 |
| Relative count | CD45RA+ CD28- CD8br %T cell | rs186498341 | T | C | -1.206 | 0.133 | 2.48e-19 | 0.027 | 81.8 |
| Relative count | CD45RA+ CD28- CD8br %T cell | rs445 | C | T | 0.316 | 0.034 | 4.02e-20 | 0.028 | 85.55 |
| Relative count | CD45RA+ CD28- CD8br %T cell | rs77771777 | C | T | -0.681 | 0.101 | 2.06e-11 | 0.015 | 45.22 |
| Relative count | CD45RA+ CD8br %T cell | rs111608618 | C | T | 0.714 | 0.13 | 4.21e-08 | 0.010 | 30.19 |
| Relative count | CD45RA+ CD8br %T cell | rs115799162 | T | C | 0.581 | 0.072 | 9.35e-16 | 0.022 | 65.25 |
| Relative count | CD45RA+ CD8br %T cell | rs116007826 | A | G | 0.751 | 0.061 | 7.61e-34 | 0.050 | 150.82 |
| Relative count | CD45RA+ CD8br %T cell | rs11644019 | C | T | -0.291 | 0.039 | 1.10e-13 | 0.019 | 55.69 |
| Relative count | CD45RA+ CD8br %T cell | rs145399897 | C | T | 0.58 | 0.09 | 1.17e-10 | 0.014 | 41.8 |
| Relative count | CD45RA+ CD8br %T cell | rs180717063 | G | A | 1.029 | 0.096 | 2.13e-26 | 0.039 | 115.34 |
| Relative count | CD45RA+ CD8br %T cell | rs193047711 | T | C | 1.13 | 0.137 | 2.18e-16 | 0.023 | 68.18 |
| Relative count | CD45RA+ CD8br %T cell | rs2229321 | G | A | -0.384 | 0.066 | 6.20e-09 | 0.012 | 33.96 |
| Relative count | CD45RA+ CD8br %T cell | rs34039593 | T | G | 1.217 | 0.045 | 6.25e-146 | 0.206 | 744.06 |
| Relative count | CD45RA+ CD8br %T cell | rs362522 | C | T | 0.318 | 0.04 | 5.03e-15 | 0.021 | 61.89 |
| Relative count | CD45RA+ CD8br %T cell | rs570992367 | C | T | 0.705 | 0.09 | 4.90e-15 | 0.021 | 61.93 |
| Relative count | CD45RA+ CD8br %T cell | rs62176112 | G | A | -0.659 | 0.119 | 3.40e-08 | 0.011 | 30.59 |
| Relative count | CD45RA+ CD8br %T cell | rs9378177 | A | G | 0.882 | 0.134 | 5.06e-11 | 0.015 | 43.43 |
| Relative count | CD45RA- CD28- CD8br %CD8br | rs187032851 | C | T | 0.593 | 0.108 | 4.32e-08 | 0.008 | 30.11 |
| Relative count | CD45RA- CD28- CD8br %CD8br | rs25680 | G | A | -0.192 | 0.034 | 1.15e-08 | 0.009 | 32.69 |
| Relative count | CD45RA- CD28- CD8br %CD8br | rs35574803 | T | C | 0.746 | 0.053 | 2.35e-43 | 0.051 | 195.58 |
| Relative count | CD45RA- CD28- CD8br %CD8br | rs529513098 | T | G | 0.707 | 0.1 | 2.19e-12 | 0.013 | 49.67 |
| Relative count | CD45RA- CD28- CD8br %CD8br | rs551832918 | A | G | 0.785 | 0.106 | 1.22e-13 | 0.015 | 55.36 |
| Relative count | CD45RA- CD28- CD8br %CD8br | rs564347773 | G | A | 0.646 | 0.08 | 6.64e-16 | 0.018 | 65.81 |
| Relative count | CD45RA- CD28- CD8br %CD8br | rs569077190 | G | A | 0.365 | 0.055 | 5.19e-11 | 0.012 | 43.35 |
| Relative count | CD45RA- CD28- CD8br %CD8br | rs75918255 | G | A | 0.495 | 0.068 | 5.54e-13 | 0.014 | 52.35 |
| Relative count | CD45RA- CD28- CD8br %CD8br | rs76929865 | A | C | 0.574 | 0.087 | 5.04e-11 | 0.012 | 43.4 |
| Relative count | CD45RA- CD4+ %T cell | rs140970775 | A | G | 0.326 | 0.033 | 1.19e-22 | 0.026 | 97.18 |
| Relative count | CD45RA- CD4+ %T cell | rs1800973 | C | A | 0.308 | 0.045 | 6.79e-12 | 0.013 | 47.36 |
| Relative count | CD45RA- CD4+ %T cell | rs3014270 | C | T | -0.261 | 0.046 | 2.04e-08 | 0.009 | 31.59 |
| Relative count | CD45RA- CD4+ %T cell | rs445 | C | T | -0.179 | 0.031 | 6.35e-09 | 0.009 | 33.87 |
| Relative count | CD62L- CD86+ myeloid DC %DC | rs185048341 | G | A | -0.656 | 0.119 | 3.83e-08 | 0.016 | 30.47 |
| Relative count | CD62L- CD86+ myeloid DC %DC | rs553720752 | G | A | -0.693 | 0.122 | 1.62e-08 | 0.017 | 32.18 |
| Relative count | CD62L- CD86+ myeloid DC %DC | rs67760360 | G | A | -0.341 | 0.038 | 2.20e-19 | 0.043 | 82.79 |
| Relative count | CD62L- DC %DC | rs2463509 | C | T | -1.139 | 0.204 | 2.52e-08 | 0.009 | 31.19 |
| Relative count | CD62L- DC %DC | rs6729180 | C | T | 0.262 | 0.022 | 3.19e-32 | 0.040 | 142.45 |
| Relative count | CD62L- DC %DC | rs6751481 | T | C | 0.166 | 0.022 | 2.07e-14 | 0.017 | 58.93 |
| Relative count | CD62L- HLA DR++ monocyte %monocyte | rs1801274 | A | G | -0.463 | 0.026 | 2.62e-69 | 0.083 | 323.34 |
| Relative count | CD62L- HLA DR++ monocyte %monocyte | rs184012084 | G | A | 0.666 | 0.091 | 2.86e-13 | 0.015 | 53.67 |
| Relative count | CD62L- HLA DR++ monocyte %monocyte | rs28655219 | T | C | -0.313 | 0.036 | 7.57e-18 | 0.020 | 74.79 |
| Relative count | CD62L- plasmacytoid DC %DC | rs112044576 | G | A | 0.518 | 0.093 | 2.31e-08 | 0.009 | 31.34 |
| Relative count | CD62L- plasmacytoid DC %DC | rs141485696 | A | G | 0.53 | 0.091 | 5.84e-09 | 0.009 | 34.04 |
| Relative count | CD62L- plasmacytoid DC %DC | rs3087456 | G | A | -0.469 | 0.026 | 7.09e-69 | 0.082 | 321.1 |
| Relative count | CD62L- plasmacytoid DC %DC | rs55724950 | G | A | -0.26 | 0.047 | 3.05e-08 | 0.008 | 30.8 |
| Relative count | CD62L- plasmacytoid DC %DC | rs62029480 | C | T | 0.44 | 0.077 | 1.10e-08 | 0.009 | 32.79 |
| Relative count | CD62L- plasmacytoid DC %DC | rs68174286 | C | T | 0.27 | 0.044 | 1.18e-09 | 0.010 | 37.17 |
| Relative count | CD62L- plasmacytoid DC %DC | rs7032773 | C | T | -0.155 | 0.025 | 5.15e-10 | 0.011 | 38.83 |
| Relative count | CD86+ myeloid DC %DC | rs113519443 | T | C | 0.492 | 0.075 | 5.56e-11 | 0.027 | 43.52 |
| Relative count | CD86+ myeloid DC %DC | rs3865444 | C | A | -1.151 | 0.039 | 1.71e-153 | 0.359 | 877.08 |
| Relative count | CD86+ myeloid DC %DC | rs62114147 | C | T | 0.262 | 0.037 | 2.56e-12 | 0.031 | 49.7 |
| Relative count | CD86+ myeloid DC %DC | rs7351079 | A | G | -0.368 | 0.042 | 9.03e-18 | 0.046 | 75.39 |
| Relative count | CD86+ plasmacytoid DC %DC | rs2226163 | A | G | 0.249 | 0.026 | 9.47e-22 | 0.031 | 93.24 |
| Relative count | CD86+ plasmacytoid DC %DC | rs3116493 | G | T | 0.303 | 0.026 | 8.44e-30 | 0.043 | 131.44 |
| Relative count | CD86+ plasmacytoid DC %DC | rs7423567 | C | T | -0.162 | 0.03 | 4.29e-08 | 0.010 | 30.16 |
| Relative count | CD8br %leukocyte | rs112266065 | C | T | -1.008 | 0.116 | 4.08e-18 | 0.020 | 75.99 |
| Relative count | CD8br %leukocyte | rs114919524 | A | C | -1.137 | 0.107 | 5.45e-26 | 0.030 | 112.85 |
| Relative count | CD8br %leukocyte | rs141059303 | G | A | -0.652 | 0.117 | 2.96e-08 | 0.008 | 30.86 |
| Relative count | CD8br %leukocyte | rs144365928 | A | G | -0.691 | 0.078 | 1.09e-18 | 0.021 | 78.69 |
| Relative count | CD8br %leukocyte | rs148516965 | C | T | -0.551 | 0.056 | 2.24e-22 | 0.026 | 95.9 |
| Relative count | CD8br %leukocyte | rs149167072 | A | G | 0.339 | 0.054 | 2.81e-10 | 0.011 | 40.01 |
| Relative count | CD8br %leukocyte | rs184205740 | C | T | -1.198 | 0.116 | 1.79e-24 | 0.028 | 105.69 |
| Relative count | CD8br %leukocyte | rs185098316 | G | A | -0.311 | 0.052 | 3.36e-09 | 0.010 | 35.11 |
| Relative count | CD8br %leukocyte | rs185430947 | A | G | -0.976 | 0.109 | 4.20e-19 | 0.022 | 80.59 |
| Relative count | CD8br %leukocyte | rs187065953 | T | C | -0.862 | 0.096 | 3.15e-19 | 0.022 | 81.2 |
| Relative count | CD8br %leukocyte | rs188216171 | G | A | -0.907 | 0.11 | 2.04e-16 | 0.018 | 68.13 |
| Relative count | CD8br %leukocyte | rs189799494 | G | T | -0.664 | 0.051 | 1.58e-38 | 0.045 | 172.35 |
| Relative count | CD8br %leukocyte | rs191363479 | C | T | -0.977 | 0.11 | 8.72e-19 | 0.021 | 79.08 |
| Relative count | CD8br %leukocyte | rs193039847 | G | A | -1.362 | 0.087 | 1.03e-53 | 0.063 | 245.74 |
| Relative count | CD8br %leukocyte | rs4657041 | T | C | -0.174 | 0.027 | 1.31e-10 | 0.011 | 41.49 |
| Relative count | CD8br %leukocyte | rs532866318 | T | G | -1.371 | 0.12 | 9.68e-30 | 0.035 | 130.68 |
| Relative count | CD8br %leukocyte | rs535059510 | C | T | -0.98 | 0.118 | 1.42e-16 | 0.018 | 68.87 |
| Relative count | CD8br %leukocyte | rs539130256 | T | C | -0.58 | 0.106 | 4.34e-08 | 0.008 | 30.09 |
| Relative count | CD8br %leukocyte | rs539241708 | C | T | -1.187 | 0.085 | 5.42e-43 | 0.050 | 193.67 |
| Relative count | CD8br %leukocyte | rs556260593 | A | C | -1.009 | 0.106 | 2.81e-21 | 0.024 | 90.73 |
| Relative count | CD8br %leukocyte | rs557577522 | G | A | -0.762 | 0.071 | 1.60e-26 | 0.031 | 115.34 |
| Relative count | CD8br %leukocyte | rs568374507 | C | T | -0.587 | 0.059 | 3.85e-23 | 0.026 | 99.44 |
| Relative count | CD8br %leukocyte | rs5758649 | C | T | -0.602 | 0.045 | 5.24e-40 | 0.047 | 179.45 |
| Relative count | CD8br %leukocyte | rs73888140 | G | A | -0.585 | 0.06 | 4.04e-22 | 0.025 | 94.69 |
| Relative count | CD8br %leukocyte | rs74989989 | T | C | -1.288 | 0.034 | 1.00e-200 | 0.285 | 1459.94 |
| Relative count | CD8br %leukocyte | rs78806227 | C | T | -0.996 | 0.12 | 1.92e-16 | 0.018 | 68.31 |
| Relative count | CD8br %leukocyte | rs79085286 | T | C | -0.394 | 0.054 | 2.41e-13 | 0.015 | 54.02 |
| Relative count | CD8dim NKT %lymphocyte | rs140865820 | A | G | -0.377 | 0.068 | 3.11e-08 | 0.010 | 30.77 |
| Relative count | CD8dim NKT %lymphocyte | rs142456232 | C | T | -0.329 | 0.058 | 1.55e-08 | 0.010 | 32.14 |
| Relative count | CD8dim NKT %lymphocyte | rs188161802 | A | G | -0.417 | 0.075 | 2.96e-08 | 0.010 | 30.86 |
| Relative count | CM CD4+ %CD4+ | rs138297283 | C | T | 0.687 | 0.123 | 2.55e-08 | 0.010 | 31.16 |
| Relative count | CM CD4+ %CD4+ | rs4987360 | A | G | 0.336 | 0.03 | 4.37e-29 | 0.040 | 127.91 |
| Relative count | CM CD4+ %CD4+ | rs7139370 | C | T | 0.295 | 0.054 | 4.96e-08 | 0.010 | 29.87 |
| Relative count | CM CD4+ %CD4+ | rs9859349 | G | A | 0.227 | 0.042 | 4.89e-08 | 0.010 | 29.89 |
| Relative count | CM CD4+ %T cell | rs3087456 | G | A | -0.453 | 0.027 | 1.47e-62 | 0.075 | 289.57 |
| Relative count | CM CD4+ %T cell | rs542160 | C | T | -0.13 | 0.024 | 4.62e-08 | 0.008 | 29.99 |
| Relative count | CM CD4+ %T cell | rs72779785 | A | G | -0.185 | 0.032 | 5.96e-09 | 0.009 | 33.98 |
| Relative count | CM CD4+ %T cell | rs9378213 | G | T | -0.198 | 0.03 | 3.79e-11 | 0.012 | 43.98 |
| Relative count | CM CD8br %T cell | rs113013837 | A | G | 0.669 | 0.104 | 1.20e-10 | 0.011 | 41.71 |
| Relative count | CM CD8br %T cell | rs11643297 | A | G | 0.148 | 0.026 | 7.89e-09 | 0.009 | 33.41 |
| Relative count | CM CD8br %T cell | rs139732336 | C | T | 0.507 | 0.091 | 2.63e-08 | 0.008 | 31.09 |
| Relative count | CM CD8br %T cell | rs28739016 | T | G | 0.792 | 0.103 | 1.49e-14 | 0.016 | 59.55 |
| Relative count | CM CD8br %T cell | rs3934748 | G | T | 0.512 | 0.089 | 1.12e-08 | 0.009 | 32.76 |
| Relative count | CM DN (CD4-CD8-) %T cell | rs2236073 | G | A | 0.211 | 0.035 | 2.92e-09 | 0.019 | 35.56 |
| Relative count | CM DN (CD4-CD8-) %T cell | rs573510881 | A | G | 0.702 | 0.127 | 3.64e-08 | 0.016 | 30.55 |
| Relative count | CM DN (CD4-CD8-) %T cell | rs9400058 | A | G | -0.824 | 0.071 | 3.42e-30 | 0.069 | 135 |
| Relative count | DP (CD4+CD8+) %T cell | rs111758898 | T | C | 0.331 | 0.053 | 5.12e-10 | 0.010 | 38.82 |
| Relative count | DP (CD4+CD8+) %T cell | rs115536607 | C | T | 0.358 | 0.054 | 4.64e-11 | 0.012 | 43.57 |
| Relative count | DP (CD4+CD8+) %T cell | rs117147408 | A | G | 0.675 | 0.076 | 6.50e-19 | 0.021 | 79.74 |
| Relative count | DP (CD4+CD8+) %T cell | rs144837535 | T | C | 0.714 | 0.07 | 3.93e-24 | 0.028 | 104.1 |
| Relative count | DP (CD4+CD8+) %T cell | rs150386792 | G | A | 0.703 | 0.074 | 2.04e-21 | 0.024 | 91.38 |
| Relative count | DP (CD4+CD8+) %T cell | rs181878688 | G | A | 0.872 | 0.067 | 2.66e-38 | 0.045 | 171.29 |
| Relative count | DP (CD4+CD8+) %T cell | rs186978368 | C | T | 0.68 | 0.072 | 4.42e-21 | 0.024 | 89.82 |
| Relative count | DP (CD4+CD8+) %T cell | rs557204100 | A | G | 0.514 | 0.088 | 4.58e-09 | 0.009 | 34.51 |
| Relative count | DP (CD4+CD8+) %T cell | rs57245661 | A | C | 0.616 | 0.092 | 3.07e-11 | 0.012 | 44.38 |
| Relative count | DP (CD4+CD8+) %T cell | rs62432266 | G | T | 0.437 | 0.052 | 7.78e-17 | 0.019 | 70.08 |
| Relative count | DP (CD4+CD8+) %T cell | rs7569868 | C | T | 0.22 | 0.031 | 2.88e-12 | 0.013 | 49.08 |
| Relative count | DP (CD4+CD8+) %T cell | rs77738700 | G | A | -0.377 | 0.039 | 7.84e-22 | 0.025 | 93.35 |
| Relative count | DP (CD4+CD8+) %T cell | rs9916629 | T | C | 0.138 | 0.024 | 1.54e-08 | 0.009 | 32.11 |
| Relative count | DP (CD4+CD8+) %leukocyte | rs113013837 | A | G | 0.956 | 0.136 | 3.23e-12 | 0.026 | 49.15 |
| Relative count | DP (CD4+CD8+) %leukocyte | rs139732336 | C | T | 0.781 | 0.122 | 1.67e-10 | 0.022 | 41.27 |
| Relative count | DP (CD4+CD8+) %leukocyte | rs191007685 | A | G | 0.686 | 0.104 | 6.63e-11 | 0.023 | 43.12 |
| Relative count | DP (CD4+CD8+) %leukocyte | rs28739016 | T | G | 1.072 | 0.131 | 4.60e-16 | 0.035 | 67.1 |
| Relative count | DP (CD4+CD8+) %leukocyte | rs55712453 | A | G | 0.779 | 0.12 | 9.18e-11 | 0.023 | 42.43 |
| Relative count | DP (CD4+CD8+) %leukocyte | rs575687159 | C | T | 0.711 | 0.119 | 2.50e-09 | 0.019 | 35.86 |
| Relative count | EM CD4+ %T cell | rs11653761 | G | T | -0.149 | 0.025 | 2.30e-09 | 0.010 | 35.86 |
| Relative count | EM CD4+ %T cell | rs11777835 | T | C | -0.144 | 0.025 | 1.21e-08 | 0.009 | 32.58 |
| Relative count | EM CD4+ %T cell | rs9520836 | A | G | 0.157 | 0.025 | 2.91e-10 | 0.011 | 39.93 |
| Relative count | EM CD8br %CD8br | rs1037633 | T | G | 0.234 | 0.038 | 1.19e-09 | 0.010 | 37.17 |
| Relative count | EM CD8br %CD8br | rs2018404 | C | T | -0.244 | 0.038 | 9.14e-11 | 0.011 | 42.23 |
| Relative count | EM CD8br %CD8br | rs4721572 | T | C | 0.229 | 0.028 | 2.47e-16 | 0.018 | 67.79 |
| Relative count | EM CD8br %CD8br | rs709589 | C | T | -0.571 | 0.03 | 4.00e-76 | 0.089 | 357.19 |
| Relative count | EM CD8br %T cell | rs12138291 | G | A | -0.232 | 0.038 | 9.94e-10 | 0.013 | 37.56 |
| Relative count | EM CD8br %T cell | rs1723018 | A | G | 0.367 | 0.026 | 7.25e-43 | 0.062 | 194.38 |
| Relative count | EM CD8br %T cell | rs62028293 | T | C | 0.284 | 0.052 | 3.92e-08 | 0.010 | 30.33 |
| Relative count | EM CD8br %T cell | rs858545 | A | C | -0.149 | 0.027 | 4.23e-08 | 0.010 | 30.19 |
| Relative count | EM CD8br %T cell | rs952962 | A | G | 0.231 | 0.039 | 5.49e-09 | 0.012 | 34.2 |
| Relative count | HLA DR+ CD4+ %lymphocyte | rs1801274 | A | G | -0.45 | 0.026 | 2.10e-64 | 0.077 | 298.77 |
| Relative count | HLA DR+ CD4+ %lymphocyte | rs184012084 | G | A | 0.751 | 0.091 | 1.52e-16 | 0.019 | 68.76 |
| Relative count | HLA DR+ CD4+ %lymphocyte | rs28655219 | T | C | -0.398 | 0.036 | 1.03e-27 | 0.033 | 120.98 |
| Relative count | HLA DR+ CD4+ %lymphocyte | rs55994243 | T | G | 0.296 | 0.044 | 2.06e-11 | 0.012 | 45.18 |
| Relative count | HLA DR+ CD8br %T cell | rs115805162 | C | T | 0.239 | 0.034 | 2.03e-12 | 0.017 | 49.83 |
| Relative count | HLA DR+ CD8br %T cell | rs569911 | T | C | -0.155 | 0.028 | 2.70e-08 | 0.010 | 31.06 |
| Relative count | HLA DR+ CD8br %T cell | rs71632979 | A | G | -0.549 | 0.03 | 3.58e-69 | 0.099 | 325.41 |
| Relative count | HLA DR+ NK %NK | rs10494360 | G | A | -0.947 | 0.027 | 1.00e-200 | 0.251 | 1211.62 |
| Relative count | HLA DR+ NK %NK | rs114010589 | T | C | 0.504 | 0.037 | 2.05e-41 | 0.049 | 186.32 |
| Relative count | HLA DR+ NK %NK | rs146866569 | C | T | 0.489 | 0.086 | 1.34e-08 | 0.009 | 32.41 |
| Relative count | HLA DR+ NK %NK | rs148765873 | T | C | 0.353 | 0.063 | 1.94e-08 | 0.009 | 31.68 |
| Relative count | HLA DR+ NK %NK | rs149750765 | G | A | -0.476 | 0.073 | 7.08e-11 | 0.012 | 42.73 |
| Relative count | HLA DR+ NK %NK | rs182914496 | G | A | -0.636 | 0.097 | 5.54e-11 | 0.012 | 43.22 |
| Relative count | HLA DR+ NK %NK | rs2045572 | G | A | -0.779 | 0.137 | 1.51e-08 | 0.009 | 32.15 |
| Relative count | HLA DR+ NK %NK | rs537294345 | C | T | 0.679 | 0.1 | 1.34e-11 | 0.013 | 46.02 |
| Relative count | HLA DR+ NK %NK | rs6656063 | C | T | -0.211 | 0.029 | 2.34e-13 | 0.015 | 54.07 |
| Relative count | HLA DR+ NK %NK | rs71639910 | G | A | -0.561 | 0.048 | 4.63e-31 | 0.036 | 136.82 |
| Relative count | HLA DR+ NK %NK | rs75072970 | T | C | -0.445 | 0.057 | 5.48e-15 | 0.017 | 61.56 |
| Relative count | HLA DR+ NK %NK | rs77418963 | A | G | -0.507 | 0.081 | 4.60e-10 | 0.011 | 39.03 |
| Relative count | HLA DR+ T cell%T cell | rs113075410 | A | G | 0.371 | 0.055 | 2.31e-11 | 0.012 | 44.95 |
| Relative count | HLA DR+ T cell%T cell | rs11544989 | A | G | 0.14 | 0.025 | 3.56e-08 | 0.008 | 30.49 |
| Relative count | HLA DR+ T cell%T cell | rs115536607 | C | T | 0.398 | 0.055 | 3.73e-13 | 0.014 | 53.15 |
| Relative count | HLA DR+ T cell%T cell | rs117147408 | A | G | 0.735 | 0.076 | 6.79e-22 | 0.025 | 93.6 |
| Relative count | HLA DR+ T cell%T cell | rs144837535 | T | C | 0.731 | 0.07 | 6.62e-25 | 0.029 | 107.73 |
| Relative count | HLA DR+ T cell%T cell | rs150386792 | G | A | 0.745 | 0.074 | 1.39e-23 | 0.027 | 101.55 |
| Relative count | HLA DR+ T cell%T cell | rs181878688 | G | A | 0.934 | 0.067 | 3.72e-43 | 0.050 | 194.61 |
| Relative count | HLA DR+ T cell%T cell | rs182663933 | T | C | 0.411 | 0.057 | 6.58e-13 | 0.014 | 52 |
| Relative count | HLA DR+ T cell%T cell | rs186978368 | C | T | 0.698 | 0.072 | 7.21e-22 | 0.025 | 93.49 |
| Relative count | HLA DR+ T cell%T cell | rs57245661 | A | C | 0.626 | 0.093 | 2.00e-11 | 0.012 | 45.23 |
| Relative count | HLA DR+ T cell%T cell | rs62432266 | G | T | 0.457 | 0.053 | 4.82e-18 | 0.020 | 75.72 |
| Relative count | HLA DR+ T cell%T cell | rs77738700 | G | A | -0.326 | 0.04 | 2.06e-16 | 0.018 | 68.15 |
| Relative count | HLA DR+ T cell%lymphocyte | rs25680 | G | A | -0.285 | 0.046 | 9.11e-10 | 0.020 | 37.86 |
| Relative count | HLA DR+ T cell%lymphocyte | rs35574803 | T | C | 0.934 | 0.07 | 1.08e-38 | 0.088 | 177.18 |
| Relative count | HLA DR+ T cell%lymphocyte | rs529513098 | T | G | 0.733 | 0.13 | 1.90e-08 | 0.017 | 31.82 |
| Relative count | HLA DR+ T cell%lymphocyte | rs551832918 | A | G | 0.85 | 0.137 | 7.56e-10 | 0.020 | 38.22 |
| Relative count | HLA DR+ T cell%lymphocyte | rs56237381 | G | A | 0.545 | 0.099 | 4.44e-08 | 0.016 | 30.17 |
| Relative count | HLA DR+ T cell%lymphocyte | rs564347773 | G | A | 0.725 | 0.105 | 7.91e-12 | 0.025 | 47.39 |
| Relative count | HLA DR+ T cell%lymphocyte | rs75918255 | G | A | 0.578 | 0.092 | 3.41e-10 | 0.021 | 39.81 |
| Relative count | IgD+ %B cell | rs111758898 | T | C | 0.342 | 0.053 | 1.71e-10 | 0.011 | 40.98 |
| Relative count | IgD+ %B cell | rs115536607 | C | T | 0.369 | 0.054 | 1.25e-11 | 0.012 | 46.15 |
| Relative count | IgD+ %B cell | rs117055820 | T | C | 0.327 | 0.059 | 3.22e-08 | 0.008 | 30.69 |
| Relative count | IgD+ %B cell | rs117147408 | A | G | 0.704 | 0.076 | 2.75e-20 | 0.023 | 86.12 |
| Relative count | IgD+ %B cell | rs144837535 | T | C | 0.739 | 0.07 | 1.37e-25 | 0.029 | 110.94 |
| Relative count | IgD+ %B cell | rs150386792 | G | A | 0.719 | 0.074 | 3.77e-22 | 0.025 | 94.81 |
| Relative count | IgD+ %B cell | rs181878688 | G | A | 0.906 | 0.067 | 6.47e-41 | 0.048 | 183.85 |
| Relative count | IgD+ %B cell | rs186978368 | C | T | 0.705 | 0.072 | 2.17e-22 | 0.026 | 95.93 |
| Relative count | IgD+ %B cell | rs557204100 | A | G | 0.527 | 0.088 | 2.20e-09 | 0.010 | 35.94 |
| Relative count | IgD+ %B cell | rs57245661 | A | C | 0.628 | 0.093 | 1.44e-11 | 0.012 | 45.88 |
| Relative count | IgD+ %B cell | rs62432266 | G | T | 0.441 | 0.052 | 5.19e-17 | 0.019 | 70.92 |
| Relative count | IgD+ %B cell | rs7569868 | C | T | 0.227 | 0.031 | 6.35e-13 | 0.014 | 52.09 |
| Relative count | IgD+ %B cell | rs77738700 | G | A | -0.383 | 0.039 | 2.68e-22 | 0.025 | 95.51 |
| Relative count | IgD+ %Lymphocyte | rs1800973 | C | A | -0.249 | 0.045 | 2.82e-08 | 0.008 | 30.94 |
| Relative count | IgD+ %Lymphocyte | rs762789 | G | A | -0.186 | 0.025 | 9.48e-14 | 0.015 | 55.84 |
| Relative count | IgD+ %Lymphocyte | rs77465361 | G | T | 0.457 | 0.079 | 8.02e-09 | 0.009 | 33.41 |
| Relative count | IgD+ CD24- %B cell | rs17201560 | T | C | -0.519 | 0.086 | 2.13e-09 | 0.012 | 36.05 |
| Relative count | IgD+ CD24- %B cell | rs2571390 | T | C | 0.364 | 0.038 | 6.16e-22 | 0.031 | 94.11 |
| Relative count | IgD+ CD24- %B cell | rs3020726 | A | G | -0.298 | 0.04 | 7.15e-14 | 0.019 | 56.52 |
| Relative count | IgD+ CD24- %lymphocyte | rs112505169 | C | T | 0.788 | 0.112 | 2.43e-12 | 0.014 | 49.41 |
| Relative count | IgD+ CD24- %lymphocyte | rs142500247 | T | C | 0.47 | 0.078 | 1.76e-09 | 0.010 | 36.41 |
| Relative count | IgD+ CD24- %lymphocyte | rs408686 | C | A | -0.223 | 0.032 | 5.06e-12 | 0.014 | 47.97 |
| Relative count | IgD+ CD24- %lymphocyte | rs6751481 | T | C | 0.209 | 0.025 | 5.19e-17 | 0.020 | 70.94 |
| Relative count | IgD+ CD38- %B cell | rs111411953 | C | T | 0.2 | 0.035 | 1.87e-08 | 0.009 | 31.76 |
| Relative count | IgD+ CD38- %B cell | rs11187166 | C | T | 0.176 | 0.032 | 3.07e-08 | 0.008 | 30.77 |
| Relative count | IgD+ CD38- %B cell | rs12210947 | A | G | 0.197 | 0.035 | 1.62e-08 | 0.009 | 32.03 |
| Relative count | IgD+ CD38- %B cell | rs144398648 | C | T | 0.83 | 0.119 | 3.05e-12 | 0.013 | 48.99 |
| Relative count | IgD+ CD38- %B cell | rs55809481 | T | C | -0.283 | 0.029 | 8.18e-23 | 0.026 | 97.86 |
| Relative count | IgD+ CD38- %B cell | rs6459417 | C | T | 0.214 | 0.037 | 7.67e-09 | 0.009 | 33.5 |
| Relative count | IgD+ CD38- %B cell | rs74621542 | T | C | -0.288 | 0.042 | 1.03e-11 | 0.013 | 46.57 |
| Relative count | IgD+ CD38- %B cell | rs7772894 | C | T | 0.294 | 0.05 | 3.12e-09 | 0.010 | 35.25 |
| Relative count | IgD+ CD38- %lymphocyte | rs71632979 | A | G | 0.809 | 0.028 | 4.21e-165 | 0.198 | 838.89 |
| Relative count | IgD+ CD38- %lymphocyte | rs71639910 | G | A | 0.361 | 0.052 | 4.90e-12 | 0.014 | 48.04 |
| Relative count | IgD+ CD38- %lymphocyte | rs74341264 | A | G | 0.351 | 0.04 | 3.34e-18 | 0.022 | 76.48 |
| Relative count | IgD+ CD38br %lymphocyte | rs10748649 | A | C | 0.416 | 0.026 | 9.56e-57 | 0.083 | 262.96 |
| Relative count | IgD+ CD38br %lymphocyte | rs12778618 | T | C | 0.422 | 0.065 | 8.83e-11 | 0.014 | 42.35 |
| Relative count | IgD+ CD38br %lymphocyte | rs3116494 | G | A | 0.234 | 0.027 | 1.53e-17 | 0.025 | 73.54 |
| Relative count | IgD+ CD38dim %B cell | rs112358299 | G | A | -0.409 | 0.054 | 6.15e-14 | 0.019 | 56.84 |
| Relative count | IgD+ CD38dim %B cell | rs12778618 | T | C | -0.649 | 0.066 | 3.04e-22 | 0.032 | 95.55 |
| Relative count | IgD+ CD38dim %B cell | rs142648862 | C | T | -0.683 | 0.1 | 9.16e-12 | 0.016 | 46.84 |
| Relative count | IgD+ CD38dim %B cell | rs181391987 | G | A | 0.443 | 0.074 | 1.98e-09 | 0.012 | 36.2 |
| Relative count | IgD+ CD38dim %B cell | rs191819784 | G | A | -0.673 | 0.082 | 2.73e-16 | 0.023 | 67.72 |
| Relative count | IgD+ CD38dim %B cell | rs4075310 | C | T | -0.718 | 0.024 | 7.97e-170 | 0.232 | 882.82 |
| Relative count | IgD+ CD38dim %B cell | rs72812685 | G | A | -0.571 | 0.064 | 8.41e-19 | 0.026 | 79.41 |
| Relative count | IgD+ CD38dim %B cell | rs7906684 | G | A | -0.848 | 0.088 | 7.38e-22 | 0.031 | 93.73 |
| Relative count | IgD+ CD38dim %lymphocyte | rs144837535 | T | C | 0.372 | 0.067 | 3.44e-08 | 0.008 | 30.55 |
| Relative count | IgD+ CD38dim %lymphocyte | rs181878688 | G | A | 0.413 | 0.065 | 1.84e-10 | 0.011 | 40.85 |
| Relative count | IgD+ CD38dim %lymphocyte | rs6755452 | T | G | 0.213 | 0.03 | 7.04e-13 | 0.014 | 51.88 |
| Relative count | IgD+ CD38dim %lymphocyte | rs9896155 | A | G | 0.143 | 0.025 | 7.59e-09 | 0.009 | 33.5 |
| Relative count | IgD- CD24- %lymphocyte | rs115805162 | C | T | 0.246 | 0.034 | 2.85e-13 | 0.018 | 53.77 |
| Relative count | IgD- CD24- %lymphocyte | rs569911 | T | C | -0.172 | 0.028 | 4.62e-10 | 0.013 | 39.06 |
| Relative count | IgD- CD24- %lymphocyte | rs71632979 | A | G | -0.565 | 0.03 | 1.98e-74 | 0.106 | 352.35 |
| Relative count | IgD- CD27- %B cell | rs115536607 | C | T | 0.325 | 0.055 | 3.91e-09 | 0.009 | 34.81 |
| Relative count | IgD- CD27- %B cell | rs117147408 | A | G | 0.606 | 0.077 | 5.37e-15 | 0.017 | 61.61 |
| Relative count | IgD- CD27- %B cell | rs144837535 | T | C | 0.617 | 0.072 | 1.05e-17 | 0.020 | 74.13 |
| Relative count | IgD- CD27- %B cell | rs150386792 | G | A | 0.657 | 0.075 | 3.64e-18 | 0.020 | 76.24 |
| Relative count | IgD- CD27- %B cell | rs181878688 | G | A | 0.774 | 0.068 | 2.99e-29 | 0.034 | 128.17 |
| Relative count | IgD- CD27- %B cell | rs186978368 | C | T | 0.613 | 0.073 | 8.78e-17 | 0.019 | 69.86 |
| Relative count | IgD- CD27- %B cell | rs557204100 | A | G | 0.541 | 0.089 | 1.44e-09 | 0.010 | 36.78 |
| Relative count | IgD- CD27- %B cell | rs57245661 | A | C | 0.588 | 0.094 | 5.01e-10 | 0.010 | 38.86 |
| Relative count | IgD- CD27- %B cell | rs62432266 | G | T | 0.403 | 0.053 | 3.71e-14 | 0.016 | 57.75 |
| Relative count | IgD- CD27- %B cell | rs72956154 | A | G | 0.324 | 0.059 | 3.47e-08 | 0.008 | 30.54 |
| Relative count | IgD- CD27- %B cell | rs77738700 | G | A | -0.435 | 0.04 | 1.04e-27 | 0.032 | 120.91 |
| Relative count | IgD- CD27- %lymphocyte | rs184018666 | C | T | -0.412 | 0.067 | 7.00e-10 | 0.011 | 38.21 |
| Relative count | IgD- CD27- %lymphocyte | rs71632979 | A | G | 0.823 | 0.027 | 8.53e-178 | 0.211 | 912.06 |
| Relative count | IgD- CD27- %lymphocyte | rs71639910 | G | A | 0.417 | 0.051 | 5.86e-16 | 0.019 | 66.11 |
| Relative count | IgD- CD27- %lymphocyte | rs74341264 | A | G | 0.426 | 0.039 | 5.29e-27 | 0.033 | 117.72 |
| Relative count | IgD- CD38- %lymphocyte | rs112417310 | G | A | 0.537 | 0.089 | 1.70e-09 | 0.010 | 36.46 |
| Relative count | IgD- CD38- %lymphocyte | rs113013837 | A | G | 0.676 | 0.103 | 6.34e-11 | 0.012 | 42.92 |
| Relative count | IgD- CD38- %lymphocyte | rs28739016 | T | G | 0.791 | 0.102 | 1.18e-14 | 0.016 | 60.06 |
| Relative count | IgD- CD38- %lymphocyte | rs7189927 | T | C | 0.166 | 0.025 | 2.72e-11 | 0.012 | 44.6 |
| Relative count | IgD- CD38br %B cell | rs577535705 | G | T | 0.43 | 0.075 | 1.12e-08 | 0.009 | 32.76 |
| Relative count | IgD- CD38br %B cell | rs9520836 | A | G | 0.136 | 0.024 | 8.45e-09 | 0.009 | 33.31 |
| Relative count | IgD- CD38br %B cell | rs9916257 | G | T | 0.169 | 0.024 | 1.08e-12 | 0.014 | 51 |
| Relative count | IgD- CD38br %lymphocyte | rs11086984 | C | T | 0.211 | 0.035 | 1.67e-09 | 0.010 | 36.48 |
| Relative count | IgD- CD38br %lymphocyte | rs149803677 | A | C | 0.422 | 0.069 | 1.05e-09 | 0.010 | 37.4 |
| Relative count | IgD- CD38br %lymphocyte | rs162076 | G | A | -0.143 | 0.025 | 1.54e-08 | 0.009 | 32.13 |
| Relative count | IgD- CD38br %lymphocyte | rs1801274 | A | G | -0.211 | 0.027 | 2.77e-15 | 0.017 | 62.9 |
| Relative count | IgD- CD38br %lymphocyte | rs2250246 | A | G | -0.206 | 0.025 | 7.42e-17 | 0.019 | 70.25 |
| Relative count | IgD- CD38br %lymphocyte | rs35574803 | T | C | 0.607 | 0.054 | 2.74e-29 | 0.034 | 128.41 |
| Relative count | IgD- CD38br %lymphocyte | rs551832918 | A | G | 0.613 | 0.105 | 6.38e-09 | 0.009 | 33.86 |
| Relative count | IgD- CD38br %lymphocyte | rs564347773 | G | A | 0.542 | 0.079 | 1.05e-11 | 0.013 | 46.5 |
| Relative count | IgD- CD38br %lymphocyte | rs61916429 | G | A | -0.225 | 0.037 | 1.20e-09 | 0.010 | 37.13 |
| Relative count | IgD- CD38dim %B cell | rs111983490 | G | A | -0.301 | 0.038 | 4.36e-15 | 0.020 | 62.12 |
| Relative count | IgD- CD38dim %B cell | rs139462041 | A | G | -0.739 | 0.132 | 2.64e-08 | 0.010 | 31.12 |
| Relative count | IgD- CD38dim %B cell | rs143305120 | T | C | -0.396 | 0.072 | 4.01e-08 | 0.010 | 30.29 |
| Relative count | IgD- CD38dim %B cell | rs147210938 | G | A | -0.43 | 0.075 | 1.06e-08 | 0.011 | 32.88 |
| Relative count | IgD- CD38dim %B cell | rs148434380 | G | T | -0.725 | 0.108 | 2.51e-11 | 0.014 | 44.82 |
| Relative count | IgD- CD38dim %B cell | rs189281718 | G | T | -0.652 | 0.103 | 2.72e-10 | 0.013 | 40.15 |
| Relative count | IgD- CD38dim %B cell | rs2286975 | G | A | 0.174 | 0.03 | 6.18e-09 | 0.011 | 33.95 |
| Relative count | IgD- CD38dim %B cell | rs7192768 | T | C | -0.479 | 0.034 | 2.47e-43 | 0.060 | 196.41 |
| Relative count | IgD- CD38dim %B cell | rs74405933 | G | A | -0.773 | 0.052 | 1.06e-48 | 0.068 | 222.77 |
| Relative count | IgD- CD38dim %B cell | rs9270599 | G | A | 0.557 | 0.032 | 3.05e-65 | 0.091 | 305.2 |
| Relative count | IgD- CD38dim %lymphocyte | rs10736111 | T | C | -0.219 | 0.039 | 1.53e-08 | 0.011 | 32.17 |
| Relative count | IgD- CD38dim %lymphocyte | rs10748647 | T | C | 1.009 | 0.021 | 1.00e-200 | 0.448 | 2369.76 |
| Relative count | IgD- CD38dim %lymphocyte | rs10882701 | A | C | -0.399 | 0.027 | 7.28e-47 | 0.068 | 214.03 |
| Relative count | IgD- CD38dim %lymphocyte | rs12778797 | T | C | 0.285 | 0.028 | 2.24e-23 | 0.033 | 100.85 |
| Relative count | IgD- CD38dim %lymphocyte | rs1410600 | C | T | -0.244 | 0.028 | 6.97e-18 | 0.025 | 75.1 |
| Relative count | Lymphocyte %leukocyte | rs11766311 | G | A | -0.153 | 0.028 | 4.55e-08 | 0.010 | 30.05 |
| Relative count | Lymphocyte %leukocyte | rs445 | C | T | 0.209 | 0.034 | 1.06e-09 | 0.013 | 37.44 |
| Relative count | Lymphocyte %leukocyte | rs7073937 | C | T | -0.204 | 0.034 | 2.45e-09 | 0.012 | 35.79 |
| Relative count | NKT %lymphocyte | rs11249570 | G | A | -0.152 | 0.026 | 7.66e-09 | 0.011 | 33.55 |
| Relative count | NKT %lymphocyte | rs4656708 | A | C | -0.37 | 0.065 | 1.22e-08 | 0.011 | 32.61 |
| Relative count | NKT %lymphocyte | rs4987369 | G | T | 0.238 | 0.03 | 3.61e-15 | 0.020 | 62.54 |
| Relative count | Naive CD4+ %T cell | rs113483143 | T | C | -435.8 | 75.13 | 7.23e-09 | 0.010 | 33.63 |
| Relative count | Naive CD4+ %T cell | rs114166795 | C | T | -565.1 | 71.8 | 4.70e-15 | 0.018 | 61.91 |
| Relative count | Naive CD4+ %T cell | rs116067658 | T | G | 76.45 | 12.55 | 1.23e-09 | 0.011 | 37.09 |
| Relative count | Naive CD4+ %T cell | rs116185462 | A | G | -611.5 | 100.6 | 1.35e-09 | 0.011 | 36.93 |
| Relative count | Naive CD4+ %T cell | rs116536005 | A | C | 66.54 | 12.07 | 3.84e-08 | 0.009 | 30.37 |
| Relative count | Naive CD4+ %T cell | rs117048727 | T | C | -368.8 | 66.09 | 2.58e-08 | 0.009 | 31.12 |
| Relative count | Naive CD4+ %T cell | rs117522565 | G | A | -611.5 | 100.6 | 1.35e-09 | 0.011 | 36.93 |
| Relative count | Naive CD4+ %T cell | rs117535164 | G | A | -425.8 | 60.26 | 1.91e-12 | 0.014 | 49.9 |
| Relative count | Naive CD4+ %T cell | rs118005354 | G | T | 46.79 | 7.075 | 4.36e-11 | 0.013 | 43.71 |
| Relative count | Naive CD4+ %T cell | rs11991846 | T | C | -319.2 | 53.46 | 2.61e-09 | 0.010 | 35.63 |
| Relative count | Naive CD4+ %T cell | rs1199676 | T | C | -1484 | 98.57 | 1.19e-49 | 0.062 | 226.53 |
| Relative count | Naive CD4+ %T cell | rs13004731 | T | G | -408.2 | 66.63 | 1.00e-09 | 0.011 | 37.51 |
| Relative count | Naive CD4+ %T cell | rs130071 | G | A | 22.81 | 3.563 | 1.74e-10 | 0.012 | 40.96 |
| Relative count | Naive CD4+ %T cell | rs13087457 | A | G | -611.5 | 100.6 | 1.35e-09 | 0.011 | 36.93 |
| Relative count | Naive CD4+ %T cell | rs138961453 | T | C | -368.8 | 66.09 | 2.58e-08 | 0.009 | 31.12 |
| Relative count | Naive CD4+ %T cell | rs139457426 | C | T | 71.36 | 10.98 | 9.24e-11 | 0.012 | 42.21 |
| Relative count | Naive CD4+ %T cell | rs141461607 | G | A | 80 | 12.72 | 3.64e-10 | 0.011 | 39.53 |
| Relative count | Naive CD4+ %T cell | rs141618950 | A | C | -431.2 | 64.06 | 1.96e-11 | 0.013 | 45.28 |
| Relative count | Naive CD4+ %T cell | rs142543350 | A | G | -368.8 | 66.09 | 2.58e-08 | 0.009 | 31.12 |
| Relative count | Naive CD4+ %T cell | rs142798669 | T | C | -368.8 | 66.09 | 2.58e-08 | 0.009 | 31.12 |
| Relative count | Naive CD4+ %T cell | rs144377157 | A | G | -685.8 | 101.2 | 1.46e-11 | 0.013 | 45.9 |
| Relative count | Naive CD4+ %T cell | rs144448823 | A | C | -611.5 | 100.6 | 1.35e-09 | 0.011 | 36.93 |
| Relative count | Naive CD4+ %T cell | rs1458573 | C | T | -281.4 | 49.92 | 1.88e-08 | 0.009 | 31.76 |
| Relative count | Naive CD4+ %T cell | rs146550074 | A | G | -557.7 | 102 | 4.88e-08 | 0.009 | 29.88 |
| Relative count | Naive CD4+ %T cell | rs147133204 | A | G | -541.2 | 74.02 | 3.28e-13 | 0.015 | 53.43 |
| Relative count | Naive CD4+ %T cell | rs147602385 | T | C | -506.8 | 58.41 | 6.24e-18 | 0.022 | 75.24 |
| Relative count | Naive CD4+ %T cell | rs148799115 | A | C | 55.39 | 9.959 | 2.87e-08 | 0.009 | 30.92 |
| Relative count | Naive CD4+ %T cell | rs149545885 | G | A | -435.8 | 75.13 | 7.23e-09 | 0.010 | 33.63 |
| Relative count | Naive CD4+ %T cell | rs150382715 | T | C | -500.6 | 65.09 | 1.89e-14 | 0.017 | 59.12 |
| Relative count | Naive CD4+ %T cell | rs150383571 | T | C | 61.29 | 11.2 | 4.77e-08 | 0.009 | 29.93 |
| Relative count | Naive CD4+ %T cell | rs150498189 | C | T | -611.5 | 100.6 | 1.35e-09 | 0.011 | 36.93 |
| Relative count | Naive CD4+ %T cell | rs151061376 | T | C | -740.5 | 101.3 | 3.39e-13 | 0.015 | 53.4 |
| Relative count | Naive CD4+ %T cell | rs17212021 | T | C | -22.92 | 4.094 | 2.31e-08 | 0.009 | 31.32 |
| Relative count | Naive CD4+ %T cell | rs184982628 | G | T | -340.8 | 52.04 | 6.73e-11 | 0.012 | 42.86 |
| Relative count | Naive CD4+ %T cell | rs200838366 | A | G | -435.8 | 75.13 | 7.23e-09 | 0.010 | 33.63 |
| Relative count | Naive CD4+ %T cell | rs201675329 | G | A | -431.2 | 64.06 | 1.96e-11 | 0.013 | 45.28 |
| Relative count | Naive CD4+ %T cell | rs202227187 | A | G | -435.8 | 75.13 | 7.23e-09 | 0.010 | 33.63 |
| Relative count | Naive CD4+ %T cell | rs2615702 | C | T | -397.5 | 42.74 | 2.40e-20 | 0.025 | 86.45 |
| Relative count | Naive CD4+ %T cell | rs2805853 | A | G | -248.4 | 43.41 | 1.14e-08 | 0.010 | 32.72 |
| Relative count | Naive CD4+ %T cell | rs34961617 | A | G | 60.38 | 11.03 | 4.70e-08 | 0.009 | 29.95 |
| Relative count | Naive CD4+ %T cell | rs4744556 | A | G | -351 | 57.21 | 9.48e-10 | 0.011 | 37.62 |
| Relative count | Naive CD4+ %T cell | rs512648 | A | G | -309.7 | 50.57 | 1.02e-09 | 0.011 | 37.48 |
| Relative count | Naive CD4+ %T cell | rs56124337 | T | C | -611.5 | 100.6 | 1.35e-09 | 0.011 | 36.93 |
| Relative count | Naive CD4+ %T cell | rs58905133 | A | G | 20.08 | 2.814 | 1.18e-12 | 0.015 | 50.89 |
| Relative count | Naive CD4+ %T cell | rs6550269 | T | C | -557.7 | 102 | 4.88e-08 | 0.009 | 29.88 |
| Relative count | Naive CD4+ %T cell | rs6725974 | C | T | -292.7 | 51.26 | 1.23e-08 | 0.009 | 32.59 |
| Relative count | Naive CD4+ %T cell | rs6769348 | T | C | -460.9 | 59.66 | 1.46e-14 | 0.017 | 59.65 |
| Relative count | Naive CD4+ %T cell | rs7315940 | A | G | -121.1 | 19.13 | 2.78e-10 | 0.012 | 40.05 |
| Relative count | Naive CD4+ %T cell | rs73511513 | A | G | -557.7 | 102 | 4.88e-08 | 0.009 | 29.88 |
| Relative count | Naive CD4+ %T cell | rs75575438 | C | T | 42.31 | 7.599 | 2.78e-08 | 0.009 | 30.98 |
| Relative count | Naive CD4+ %T cell | rs7799755 | C | T | -465.1 | 65.17 | 1.16e-12 | 0.015 | 50.9 |
| Relative count | Naive CD4+ %T cell | rs78871360 | C | T | -611.5 | 100.6 | 1.35e-09 | 0.011 | 36.93 |
| Relative count | Naive CD4+ %T cell | rs78939697 | A | C | 74.28 | 11.52 | 1.29e-10 | 0.012 | 41.55 |
| Relative count | Naive CD4+ %T cell | rs9688232 | C | A | -559.4 | 102.3 | 4.88e-08 | 0.009 | 29.88 |
| Relative count | Naive CD4+ %T cell | rs9882785 | T | C | -354.6 | 64.63 | 4.41e-08 | 0.009 | 30.09 |
| Relative count | Naive CD4+ %T cell | rs9893938 | G | T | -701.9 | 128.1 | 4.62e-08 | 0.009 | 30.01 |
| Relative count | Naive CD8br %T cell | rs139084170 | C | A | 0.379 | 0.058 | 5.09e-11 | 0.012 | 43.39 |
| Relative count | Naive CD8br %T cell | rs241407 | C | T | -0.201 | 0.034 | 4.39e-09 | 0.009 | 34.58 |
| Relative count | Naive CD8br %T cell | rs28723652 | C | A | 0.411 | 0.073 | 2.15e-08 | 0.009 | 31.49 |
| Relative count | Naive CD8br %T cell | rs709589 | C | T | -0.591 | 0.03 | 4.31e-81 | 0.095 | 382.37 |
| Relative count | Naive CD8br %T cell | rs9267517 | G | A | -0.267 | 0.036 | 1.06e-13 | 0.015 | 55.67 |
| Relative count | Naive CD8br %T cell | rs9276702 | A | G | 0.22 | 0.035 | 4.55e-10 | 0.011 | 39.06 |
| Relative count | Naive DN (CD4-CD8-) %T cell | rs13004842 | C | T | 0.384 | 0.033 | 9.03e-31 | 0.045 | 136.01 |
| Relative count | Naive DN (CD4-CD8-) %T cell | rs142500247 | T | C | 0.942 | 0.078 | 9.45e-33 | 0.048 | 145.49 |
| Relative count | Naive DN (CD4-CD8-) %T cell | rs147667223 | C | T | 0.717 | 0.117 | 1.17e-09 | 0.013 | 37.21 |
| Relative count | Naive DN (CD4-CD8-) %T cell | rs17607399 | C | T | 0.252 | 0.041 | 8.19e-10 | 0.013 | 37.94 |
| Relative count | Naive DN (CD4-CD8-) %T cell | rs186119640 | T | C | 0.685 | 0.07 | 4.25e-22 | 0.032 | 94.88 |
| Relative count | Naive DN (CD4-CD8-) %T cell | rs186694440 | A | G | 0.781 | 0.117 | 2.73e-11 | 0.015 | 44.66 |
| Relative count | Naive DN (CD4-CD8-) %T cell | rs77745909 | A | G | 0.827 | 0.126 | 6.19e-11 | 0.015 | 43.04 |
| Relative count | Naive-mature B cell %B cell | rs1084560 | A | G | -0.133 | 0.023 | 9.71e-09 | 0.009 | 33.06 |
| Relative count | Naive-mature B cell %B cell | rs3087456 | G | A | -0.461 | 0.025 | 3.08e-70 | 0.084 | 327.82 |
| Relative count | Naive-mature B cell %B cell | rs3104414 | G | A | 0.169 | 0.029 | 9.31e-09 | 0.009 | 33.14 |
| Relative count | Naive-mature B cell %B cell | rs7032773 | C | T | -0.147 | 0.024 | 1.46e-09 | 0.010 | 36.75 |
| Relative count | Naive-mature B cell %lymphocyte | rs11653761 | G | T | -0.136 | 0.025 | 3.18e-08 | 0.008 | 30.73 |
| Relative count | Naive-mature B cell %lymphocyte | rs12874404 | A | G | 0.222 | 0.026 | 3.31e-17 | 0.019 | 71.82 |
| Relative count | Naive-mature B cell %lymphocyte | rs139370814 | C | T | 0.515 | 0.087 | 3.07e-09 | 0.010 | 35.29 |
| Relative count | PB/PC %lymphocyte | rs12342539 | C | T | -0.166 | 0.03 | 2.25e-08 | 0.009 | 31.4 |
| Relative count | PB/PC %lymphocyte | rs12722525 | G | A | 0.448 | 0.047 | 3.61e-21 | 0.024 | 90.23 |
| Relative count | PB/PC %lymphocyte | rs706779 | T | C | -0.293 | 0.025 | 6.64e-31 | 0.036 | 136.08 |
| Relative count | Plasmacytoid DC %DC | rs12219630 | C | T | -0.176 | 0.025 | 1.98e-12 | 0.013 | 49.81 |
| Relative count | Plasmacytoid DC %DC | rs1887027 | C | T | -0.405 | 0.027 | 7.76e-48 | 0.056 | 217.24 |
| Relative count | Plasmacytoid DC %DC | rs3793662 | C | T | -0.167 | 0.029 | 8.76e-09 | 0.009 | 33.26 |
| Relative count | T cell %leukocyte | rs112505169 | C | T | 0.852 | 0.102 | 8.67e-17 | 0.020 | 69.99 |
| Relative count | T cell %leukocyte | rs11589644 | C | T | 0.77 | 0.106 | 3.86e-13 | 0.015 | 53.08 |
| Relative count | T cell %leukocyte | rs140308479 | T | G | 0.545 | 0.099 | 3.91e-08 | 0.009 | 30.31 |
| Relative count | T cell %leukocyte | rs141272483 | C | T | -0.41 | 0.071 | 8.60e-09 | 0.010 | 33.28 |
| Relative count | T cell %leukocyte | rs142500247 | T | C | 0.691 | 0.071 | 5.39e-22 | 0.027 | 94.16 |
| Relative count | T cell %leukocyte | rs17607399 | C | T | 0.209 | 0.037 | 2.14e-08 | 0.009 | 31.5 |
| Relative count | T cell %leukocyte | rs413431 | A | G | -0.278 | 0.03 | 1.47e-20 | 0.025 | 87.43 |
| Relative count | T cell %leukocyte | rs555144825 | C | A | 0.571 | 0.104 | 4.10e-08 | 0.009 | 30.24 |
| Relative count | T cell %leukocyte | rs60220946 | C | T | 0.43 | 0.077 | 3.08e-08 | 0.009 | 30.79 |
| Relative count | T cell %leukocyte | rs6751481 | T | C | 0.295 | 0.023 | 3.40e-38 | 0.047 | 171.03 |
| Relative count | T/B | rs1633108 | C | A | -0.228 | 0.034 | 2.85e-11 | 0.015 | 44.57 |
| Relative count | T/B | rs3020726 | A | G | -0.422 | 0.039 | 1.83e-26 | 0.038 | 115.53 |
| Relative count | T/B | rs4081559 | C | T | 0.264 | 0.03 | 5.44e-18 | 0.025 | 75.65 |
| Relative count | TCRgd %T cell | rs12199079 | T | G | 0.226 | 0.028 | 7.54e-16 | 0.022 | 65.72 |
| Relative count | TCRgd %T cell | rs139795227 | A | C | -0.587 | 0.064 | 1.36e-19 | 0.028 | 83.15 |
| Relative count | TCRgd %T cell | rs6445484 | A | G | 0.466 | 0.045 | 1.96e-24 | 0.036 | 105.89 |
| Relative count | TD DN (CD4-CD8-) %T cell | rs148451781 | A | C | -0.391 | 0.059 | 3.04e-11 | 0.012 | 44.4 |
| Relative count | TD DN (CD4-CD8-) %T cell | rs41294937 | T | C | 0.555 | 0.04 | 1.57e-43 | 0.051 | 196.37 |
| Relative count | TD DN (CD4-CD8-) %T cell | rs41295123 | G | A | -0.346 | 0.058 | 2.28e-09 | 0.010 | 35.88 |
| Relative count | TD DN (CD4-CD8-) %T cell | rs41295351 | G | A | -0.531 | 0.089 | 2.48e-09 | 0.010 | 35.71 |
| Relative count | TD DN (CD4-CD8-) %T cell | rs7078614 | G | T | 0.238 | 0.026 | 2.59e-20 | 0.023 | 86.2 |

**Supplemental Table 3.** Effect Estimates of the Immunophenotypes on Abdominal Aortic Aneurysm in the MR Analyses.

| **Taxonomic level** | **Exposure** | **method** | **No. of SNP** | **b** | **or** | **or_lci95** | **or_uci95** | **pval** | **FDR** |
| --- | --- | --- | --- | --- | --- | --- | --- | --- | --- |
| Absolute count | CD14- CD16- AC | Inverse variance weighted | 8 | -0.015 | 0.985 | 0.976 | 0.993 | 3.93e-04 | 0.011 |
| Absolute count | CD14- CD16- AC | MR Egger | 8 | -0.016 | 0.985 | 0.963 | 1.006 | 0.211 | 0.678 |
| Absolute count | CD14- CD16- AC | Simple mode | 8 | -0.018 | 0.983 | 0.98 | 0.985 | 3.02e-06 | 5.96e-05 |
| Absolute count | CD14- CD16- AC | Weighted median | 8 | -0.013 | 0.987 | 0.986 | 0.987 | 0.00e+00 | 0.00e+00 |
| Absolute count | CD14- CD16- AC | Weighted mode | 8 | -0.013 | 0.987 | 0.986 | 0.987 | 4.53e-10 | 3.80e-08 |
| Absolute count | CD20- CD38- AC | Inverse variance weighted | 7 | 0.07 | 1.072 | 1.036 | 1.109 | 5.38e-05 | 0.002 |
| Absolute count | CD20- CD38- AC | MR Egger | 7 | 0.18 | 1.198 | 1.104 | 1.299 | 0.007 | 0.153 |
| Absolute count | CD20- CD38- AC | Simple mode | 7 | 0.072 | 1.075 | 1.057 | 1.092 | 1.32e-04 | 0.001 |
| Absolute count | CD20- CD38- AC | Weighted median | 7 | 0.012 | 1.012 | 1.008 | 1.015 | 2.86e-10 | 4.39e-10 |
| Absolute count | CD20- CD38- AC | Weighted mode | 7 | 0.012 | 1.013 | 1.009 | 1.017 | 0.001 | 0.003 |
| Absolute count | CD62L- CD86+ myeloid DC AC | Inverse variance weighted | 8 | -0.018 | 0.982 | 0.972 | 0.992 | 4.04e-04 | 0.011 |
| Absolute count | CD62L- CD86+ myeloid DC AC | MR Egger | 8 | -0.018 | 0.982 | 0.958 | 1.007 | 0.213 | 0.678 |
| Absolute count | CD62L- CD86+ myeloid DC AC | Simple mode | 8 | -0.025 | 0.975 | 0.971 | 0.98 | 1.18e-05 | 1.65e-04 |
| Absolute count | CD62L- CD86+ myeloid DC AC | Weighted median | 8 | -0.015 | 0.985 | 0.984 | 0.985 | 0.00e+00 | 0.00e+00 |
| Absolute count | CD62L- CD86+ myeloid DC AC | Weighted mode | 8 | -0.015 | 0.985 | 0.984 | 0.986 | 8.67e-10 | 5.81e-08 |
| Absolute count | CD86+ myeloid DC AC | Inverse variance weighted | 4 | 0.03 | 1.03 | 1.021 | 1.039 | 3.01e-11 | 2.04e-09 |
| Absolute count | CD86+ myeloid DC AC | MR Egger | 4 | 0.015 | 1.015 | 0.998 | 1.032 | 0.225 | 0.678 |
| Absolute count | CD86+ myeloid DC AC | Simple mode | 4 | 0.025 | 1.025 | 1.019 | 1.031 | 0.004 | 0.011 |
| Absolute count | CD86+ myeloid DC AC | Weighted median | 4 | 0.025 | 1.025 | 1.021 | 1.03 | 7.82e-30 | 1.90e-29 |
| Absolute count | CD86+ myeloid DC AC | Weighted mode | 4 | 0.025 | 1.025 | 1.02 | 1.03 | 0.002 | 0.006 |
| Absolute count | Naive DN (CD4-CD8-) AC | Inverse variance weighted | 9 | -0.017 | 0.983 | 0.974 | 0.992 | 3.77e-04 | 0.011 |
| Absolute count | Naive DN (CD4-CD8-) AC | MR Egger | 9 | -0.031 | 0.969 | 0.954 | 0.985 | 0.006 | 0.153 |
| Absolute count | Naive DN (CD4-CD8-) AC | Simple mode | 9 | -0.016 | 0.984 | 0.979 | 0.989 | 1.39e-04 | 0.001 |
| Absolute count | Naive DN (CD4-CD8-) AC | Weighted median | 9 | -0.016 | 0.984 | 0.983 | 0.985 | 0.00e+00 | 0.00e+00 |
| Absolute count | Naive DN (CD4-CD8-) AC | Weighted mode | 9 | -0.016 | 0.984 | 0.983 | 0.985 | 1.31e-08 | 3.38e-07 |
| MFI | CCR2 on monocyte | Inverse variance weighted | 8 | -0.022 | 0.978 | 0.971 | 0.986 | 3.52e-08 | 1.99e-06 |
| MFI | CCR2 on monocyte | MR Egger | 8 | -0.035 | 0.965 | 0.955 | 0.976 | 0.001 | 0.042 |
| MFI | CCR2 on monocyte | Simple mode | 8 | -0.022 | 0.978 | 0.976 | 0.981 | 8.76e-07 | 2.84e-05 |
| MFI | CCR2 on monocyte | Weighted median | 8 | -0.025 | 0.975 | 0.973 | 0.978 | 1.20e-83 | 5.08e-83 |
| MFI | CCR2 on monocyte | Weighted mode | 8 | -0.024 | 0.976 | 0.974 | 0.977 | 6.73e-09 | 2.25e-07 |
| MFI | CD127 on CD28- CD8br | Inverse variance weighted | 6 | -0.025 | 0.976 | 0.962 | 0.99 | 0.001 | 0.013 |
| MFI | CD127 on CD28- CD8br | MR Egger | 6 | -0.066 | 0.936 | 0.913 | 0.959 | 0.006 | 0.153 |
| MFI | CD127 on CD28- CD8br | Simple mode | 6 | -0.024 | 0.977 | 0.971 | 0.982 | 3.60e-04 | 0.002 |
| MFI | CD127 on CD28- CD8br | Weighted median | 6 | -0.005 | 0.995 | 0.994 | 0.996 | 2.90e-34 | 7.58e-34 |
| MFI | CD127 on CD28- CD8br | Weighted mode | 6 | -0.005 | 0.995 | 0.994 | 0.996 | 3.53e-04 | 0.001 |
| MFI | CD16-CD56 on NKT | Inverse variance weighted | 8 | -0.022 | 0.978 | 0.969 | 0.986 | 4.84e-07 | 2.35e-05 |
| MFI | CD16-CD56 on NKT | MR Egger | 8 | -0.031 | 0.97 | 0.948 | 0.992 | 0.039 | 0.321 |
| MFI | CD16-CD56 on NKT | Simple mode | 8 | -0.022 | 0.979 | 0.976 | 0.981 | 1.20e-06 | 3.34e-05 |
| MFI | CD16-CD56 on NKT | Weighted median | 8 | -0.019 | 0.981 | 0.979 | 0.983 | 5.40e-111 | 2.83e-110 |
| MFI | CD16-CD56 on NKT | Weighted mode | 8 | -0.018 | 0.982 | 0.981 | 0.983 | 1.39e-09 | 6.64e-08 |
| MFI | CD24 on sw mem | Inverse variance weighted | 4 | -0.082 | 0.922 | 0.914 | 0.929 | 1.54e-81 | 2.62e-79 |
| MFI | CD24 on sw mem | MR Egger | 4 | -0.003 | 0.997 | 0.945 | 1.052 | 0.916 | 0.982 |
| MFI | CD24 on sw mem | Simple mode | 4 | -0.079 | 0.924 | 0.91 | 0.939 | 0.002 | 0.008 |
| MFI | CD24 on sw mem | Weighted median | 4 | -0.083 | 0.921 | 0.909 | 0.933 | 4.88e-35 | 1.30e-34 |
| MFI | CD24 on sw mem | Weighted mode | 4 | -0.079 | 0.924 | 0.913 | 0.936 | 0.001 | 0.003 |
| MFI | CD25 on activated Treg | Inverse variance weighted | 7 | -0.009 | 0.991 | 0.985 | 0.997 | 0.002 | 0.035 |
| MFI | CD25 on activated Treg | MR Egger | 7 | -0.022 | 0.979 | 0.976 | 0.982 | 2.97e-05 | 0.005 |
| MFI | CD25 on activated Treg | Simple mode | 7 | -0.007 | 0.993 | 0.992 | 0.994 | 8.57e-06 | 1.37e-04 |
| MFI | CD25 on activated Treg | Weighted median | 7 | -0.009 | 0.991 | 0.989 | 0.992 | 1.99e-45 | 6.34e-45 |
| MFI | CD25 on activated Treg | Weighted mode | 7 | -0.01 | 0.99 | 0.99 | 0.991 | 4.60e-08 | 1.03e-06 |
| MFI | CD25 on CD39+ activated Treg | Inverse variance weighted | 5 | -0.028 | 0.973 | 0.956 | 0.989 | 0.001 | 0.026 |
| MFI | CD25 on CD39+ activated Treg | MR Egger | 5 | -0.027 | 0.973 | 0.922 | 1.027 | 0.392 | 0.75 |
| MFI | CD25 on CD39+ activated Treg | Simple mode | 5 | -0.006 | 0.994 | 0.989 | 0.999 | 0.075 | 0.091 |
| MFI | CD25 on CD39+ activated Treg | Weighted median | 5 | -0.005 | 0.996 | 0.995 | 0.996 | 9.18e-20 | 1.91e-19 |
| MFI | CD25 on CD39+ activated Treg | Weighted mode | 5 | -0.003 | 0.997 | 0.995 | 0.998 | 0.005 | 0.01 |
| MFI | CD45 on CD8br | Inverse variance weighted | 3 | 0.045 | 1.047 | 1.037 | 1.056 | 1.59e-23 | 1.80e-21 |
| MFI | CD45 on CD8br | MR Egger | 3 | 0.078 | 1.081 | 0.894 | 1.306 | 0.57 | 0.826 |
| MFI | CD45 on CD8br | Simple mode | 3 | 0.051 | 1.052 | 1.036 | 1.069 | 0.024 | 0.034 |
| MFI | CD45 on CD8br | Weighted median | 3 | 0.045 | 1.046 | 1.036 | 1.057 | 1.38e-19 | 2.85e-19 |
| MFI | CD45 on CD8br | Weighted mode | 3 | 0.037 | 1.038 | 1.027 | 1.049 | 0.021 | 0.029 |
| MFI | CD62L on monocyte | Inverse variance weighted | 12 | -0.02 | 0.981 | 0.971 | 0.99 | 7.10e-05 | 0.003 |
| MFI | CD62L on monocyte | MR Egger | 12 | -0.039 | 0.962 | 0.943 | 0.981 | 0.003 | 0.13 |
| MFI | CD62L on monocyte | Simple mode | 12 | -0.023 | 0.977 | 0.974 | 0.981 | 4.81e-08 | 4.62e-06 |
| MFI | CD62L on monocyte | Weighted median | 12 | -0.02 | 0.98 | 0.979 | 0.982 | 3.89e-180 | 3.62e-179 |
| MFI | CD62L on monocyte | Weighted mode | 12 | -0.018 | 0.982 | 0.981 | 0.983 | 5.68e-12 | 1.90e-09 |
| Morphological parameter | SSC-A on HLA DR+ NK | Inverse variance weighted | 3 | -0.135 | 0.873 | 0.861 | 0.885 | 2.64e-83 | 8.96e-81 |
| Morphological parameter | SSC-A on HLA DR+ NK | MR Egger | 3 | -0.304 | 0.738 | 0.685 | 0.795 | 0.079 | 0.488 |
| Morphological parameter | SSC-A on HLA DR+ NK | Simple mode | 3 | -0.127 | 0.881 | 0.85 | 0.913 | 0.02 | 0.03 |
| Morphological parameter | SSC-A on HLA DR+ NK | Weighted median | 3 | -0.133 | 0.875 | 0.853 | 0.898 | 7.79e-24 | 1.76e-23 |
| Morphological parameter | SSC-A on HLA DR+ NK | Weighted mode | 3 | -0.132 | 0.876 | 0.85 | 0.904 | 0.014 | 0.022 |
| Morphological parameter | SSC-A on HLA DR+ T cell | Inverse variance weighted | 10 | -0.018 | 0.982 | 0.973 | 0.992 | 4.41e-04 | 0.011 |
| Morphological parameter | SSC-A on HLA DR+ T cell | MR Egger | 10 | -0.016 | 0.984 | 0.96 | 1.008 | 0.231 | 0.678 |
| Morphological parameter | SSC-A on HLA DR+ T cell | Simple mode | 10 | -0.029 | 0.972 | 0.967 | 0.977 | 2.01e-06 | 4.80e-05 |
| Morphological parameter | SSC-A on HLA DR+ T cell | Weighted median | 10 | -0.015 | 0.985 | 0.984 | 0.986 | 0.00e+00 | 0.00e+00 |
| Morphological parameter | SSC-A on HLA DR+ T cell | Weighted mode | 10 | -0.015 | 0.985 | 0.984 | 0.986 | 3.12e-11 | 5.23e-09 |
| Relative count | IgD+ CD38br %lymphocyte | Inverse variance weighted | 3 | -0.037 | 0.964 | 0.944 | 0.984 | 0.001 | 0.011 |
| Relative count | IgD+ CD38br %lymphocyte | MR Egger | 3 | -0.046 | 0.955 | 0.848 | 1.074 | 0.581 | 0.828 |
| Relative count | IgD+ CD38br %lymphocyte | Simple mode | 3 | -0.036 | 0.965 | 0.961 | 0.969 | 0.004 | 0.011 |
| Relative count | IgD+ CD38br %lymphocyte | Weighted median | 3 | -0.036 | 0.964 | 0.96 | 0.969 | 1.23e-60 | 4.32e-60 |
| Relative count | IgD+ CD38br %lymphocyte | Weighted mode | 3 | -0.038 | 0.963 | 0.959 | 0.967 | 0.003 | 0.008 |

**Supplemental Table 4.** Test Results for Pleiotropy and Heterogeneity - Causal Effects of the Immunophenotypes on Abdominal Aortic Aneurysm.

| **Trait type** | **Exposure** | **Cochran's Q test** | | **MR-Egger** | | **MR-PRESSO** | |
| --- | --- | --- | --- | --- | --- | --- | --- |
|  |  | **Q** | ***P* value** | **Egger_ intercept** | ***P* value** | ***global test*** | ***Outlier-corrected*** |
| Absolute count | CD14- CD16- AC | 2853419.415 | 0 | 0 | 0.981 | <0.001 | NA |
| Absolute count | CD20- CD38- AC | 1209128.553 | 0 | -2.646e-02 | 0.039 | 0.199 | NA |
| Absolute count | CD62L- CD86+ myeloid DC AC | 3669518.909 | 0 | -3.139e-04 | 0.975 | <0.001 | NA |
| Absolute count | CD86+ myeloid DC AC | 159193.170 | 0 | 0.006 | 0.205 | 0.053 | NA |
| Absolute count | Naive DN (CD4-CD8-) AC | 3638404.105 | 0 | 0.01 | 0.082 | <0.001 | NA |
| MFI | CCR2 on monocyte | 1712835.452 | 0 | 0.009 | 0.034 | 0.003 | 0.138 |
| MFI | CD127 on CD28- CD8br | 2904688.456 | 0 | 0.024 | 0.025 | 0.003 | NA |
| MFI | CD16-CD56 on NKT | 1887741.776 | 0 | 0.006 | 0.469 | <0.001 | NA |
| MFI | CD24 on sw mem | 104555.214 | 0 | -2.884e-02 | 0.103 | 0.484 | NA |
| MFI | CD25 on CD39+ activated Treg | 2910678.918 | 0 | -2.075e-04 | 0.988 | <0.001 | NA |
| MFI | CD25 on activated Treg | 1188962.774 | 0 | 0.011 | 0 | 0.001 | NA |
| MFI | CD45 on CD8br | 38237.462 | 0 | -6.290e-03 | 0.796 | NA | NA |
| MFI | CD62L on monocyte | 5747866.753 | 0 | 0.016 | 0.065 | <0.001 | NA |
| Morphological parameter | SSC-A on HLA DR+ NK | 76402.808 | 0 | 0.03 | 0.14 | NA | NA |
| Morphological parameter | SSC-A on HLA DR+ T cell | 4720660.823 | 0 | -1.328e-03 | 0.892 | <0.001 | NA |
| Relative count | IgD+ CD38br %lymphocyte | 753404.542 | 0 | 0.004 | 0.893 | NA | NA |

**Supplemental Table 5.** Details of Instrumental Variables Used for MR Analysis on the Effect Estimates of Abdominal Aortic Aneurysm on the Immunophenotypes.

| **Exposure** | **SNP** | **Other allele** | **Effect allele** | **Beta** | **SE** | ***P* value** | **R square** | ***F*** |
| --- | --- | --- | --- | --- | --- | --- | --- | --- |
| AAA | rs11038871 | C | T | -0.0607 | 8.21e-06 | 2.58e-12 | 0.980 | 54705013.78 |
| AAA | rs11149831 | G | A | -0.0571 | 8.28e-06 | 4.73e-11 | 0.977 | 47574159.01 |
| AAA | rs1115866 | C | T | -0.0479 | 8.09e-06 | 1.98e-08 | 0.969 | 35048041.63 |
| AAA | rs11172113 | C | T | 0.0905 | 7.64e-06 | 4.30e-29 | 0.992 | 140255128.9 |
| AAA | rs112403212 | C | T | 0.0828 | 1.19e-05 | 3.14e-11 | 0.978 | 48405872.22 |
| AAA | rs11546525 | C | T | -0.055 | 7.74e-06 | 1.90e-11 | 0.978 | 50557539.41 |
| AAA | rs11548482 | G | A | -0.158 | 2.14e-05 | 2.55e-12 | 0.980 | 54424022.46 |
| AAA | rs11612171 | G | A | 0.0867 | 1.49e-05 | 1.21e-09 | 0.974 | 33859492.64 |
| AAA | rs11856657 | C | T | 0.0694 | 9.13e-06 | 5.36e-13 | 0.981 | 57718346.81 |
| AAA | rs1192524 | G | A | 0.0587 | 8.11e-06 | 1.10e-11 | 0.979 | 52338428.45 |
| AAA | rs12126142 | G | A | -0.0939 | 7.74e-06 | 3.58e-30 | 0.992 | 147364113.1 |
| AAA | rs12264252 | G | A | -0.0657 | 1.01e-05 | 4.97e-10 | 0.975 | 42428376.8 |
| AAA | rs1230345 | G | T | 0.0613 | 8.21e-06 | 1.83e-12 | 0.980 | 55753118.09 |
| AAA | rs12408895 | C | T | -0.0691 | 8.18e-06 | 7.67e-16 | 0.985 | 71301160.88 |
| AAA | rs12436072 | G | A | -0.0489 | 7.64e-06 | 1.73e-09 | 0.973 | 40954819.67 |
| AAA | rs12477249 | G | A | 0.0455 | 7.83e-06 | 4.64e-08 | 0.968 | 33771550.4 |
| AAA | rs12578371 | C | A | 0.079 | 1.18e-05 | 3.35e-13 | 0.982 | 44804483.58 |
| AAA | rs1260400 | C | T | -0.0567 | 9.94e-06 | 5.29e-11 | 0.978 | 32522115.87 |
| AAA | rs12638862 | G | A | -0.0585 | 8.49e-06 | 7.74e-11 | 0.977 | 47466100.5 |
| AAA | rs12661910 | C | T | -0.069 | 1.00e-05 | 9.10e-11 | 0.977 | 47611610.16 |
| AAA | rs13384676 | G | A | -0.0532 | 8.19e-06 | 6.07e-10 | 0.975 | 42233487.23 |
| AAA | rs13417446 | G | A | -0.0597 | 9.62e-06 | 4.22e-09 | 0.972 | 38492377.54 |
| AAA | rs140570886 | C | T | -0.3988 | 3.37e-05 | 1.36e-34 | 0.993 | 139716305.2 |
| AAA | rs142344547 | G | A | -0.1008 | 1.75e-05 | 4.62e-08 | 0.968 | 33085343.91 |
| AAA | rs1423561 | G | A | 0.0532 | 8.28e-06 | 7.94e-10 | 0.974 | 41297348.43 |
| AAA | rs1469101 | C | T | 0.0479 | 8.18e-06 | 2.73e-08 | 0.969 | 34261907.08 |
| AAA | rs1660364 | C | A | 0.0828 | 8.11e-06 | 3.94e-22 | 0.989 | 104162975.9 |
| AAA | rs17053797 | C | T | 0.049 | 8.30e-06 | 2.72e-08 | 0.969 | 34837691.48 |
| AAA | rs17688414 | C | T | 0.0864 | 8.02e-06 | 1.92e-24 | 0.990 | 116085345.7 |
| AAA | rs17710437 | C | T | 0.0592 | 9.81e-06 | 1.12e-08 | 0.970 | 36410798.86 |
| AAA | rs1892971 | G | A | 0.0946 | 9.52e-06 | 2.27e-21 | 0.989 | 98837028.8 |
| AAA | rs2111485 | G | A | -0.0543 | 7.74e-06 | 3.02e-11 | 0.978 | 49277494.2 |
| AAA | rs2125213 | G | A | 0.0487 | 7.83e-06 | 3.88e-09 | 0.972 | 38685778.92 |
| AAA | rs2150879 | G | A | 0.0658 | 7.64e-06 | 2.89e-16 | 0.985 | 74148829.96 |
| AAA | rs2223598 | C | T | 0.0514 | 8.18e-06 | 2.10e-09 | 0.973 | 39451792.85 |
| AAA | rs2227200 | G | A | 0.0618 | 7.74e-06 | 3.98e-14 | 0.983 | 63744955.92 |
| AAA | rs2227564 | C | T | 0.0653 | 8.77e-06 | 2.18e-12 | 0.980 | 55403983.49 |
| AAA | rs2245157 | G | T | -0.0741 | 1.13e-05 | 1.44e-12 | 0.980 | 42665311.91 |
| AAA | rs2246942 | G | A | -0.0728 | 7.92e-06 | 3.01e-18 | 0.987 | 84403707.56 |
| AAA | rs2289123 | G | T | -0.0589 | 9.90e-06 | 1.47e-08 | 0.970 | 35424303.46 |
| AAA | rs2736100 | C | A | 0.0467 | 7.45e-06 | 3.16e-09 | 0.972 | 39260143.46 |
| AAA | rs2784251 | G | A | 0.0502 | 1.04e-05 | 3.58e-09 | 0.972 | 23477494.87 |
| AAA | rs2836411 | C | T | 0.0833 | 8.75e-06 | 9.56e-20 | 0.988 | 90609573.43 |
| AAA | rs3011545 | G | T | 0.0653 | 8.85e-06 | 2.22e-12 | 0.980 | 54449998.73 |
| AAA | rs335183 | G | A | -0.0539 | 7.99e-06 | 1.30e-10 | 0.976 | 45473288.97 |
| AAA | rs34402154 | C | T | -0.0714 | 9.33e-06 | 2.84e-13 | 0.982 | 58624841.39 |
| AAA | rs35254673 | G | A | -0.0667 | 9.33e-06 | 8.88e-12 | 0.979 | 51160752.65 |
| AAA | rs35934224 | C | T | 0.0675 | 1.24e-05 | 1.50e-08 | 0.970 | 29636570.69 |
| AAA | rs363820 | C | T | -0.0856 | 1.25e-05 | 8.40e-11 | 0.977 | 47204700.1 |
| AAA | rs3761849 | C | T | 0.0476 | 8.28e-06 | 3.83e-08 | 0.968 | 33060758.16 |
| AAA | rs3827066 | C | T | 0.1586 | 1.02e-05 | 1.45e-48 | 0.995 | 242335027.6 |
| AAA | rs3850306 | C | A | -0.0497 | 8.09e-06 | 5.45e-09 | 0.972 | 37758291.47 |
| AAA | rs402072 | C | T | -0.0647 | 1.12e-05 | 3.70e-08 | 0.968 | 33203353.97 |
| AAA | rs42235 | C | T | 0.0628 | 7.92e-06 | 9.31e-14 | 0.982 | 62805093.07 |
| AAA | rs4252324 | C | T | 0.097 | 1.62e-05 | 1.12e-08 | 0.970 | 35956965.58 |
| AAA | rs4323539 | C | T | 0.0494 | 8.21e-06 | 1.47e-08 | 0.970 | 36203283.87 |
| AAA | rs434182 | C | T | -0.1021 | 8.11e-06 | 1.01e-32 | 0.993 | 158374357.1 |
| AAA | rs4394764 | G | A | -0.0953 | 9.62e-06 | 1.21e-20 | 0.989 | 98099316.96 |
| AAA | rs464605 | C | T | 0.0523 | 8.58e-06 | 8.54e-09 | 0.971 | 37120075.25 |
| AAA | rs4698161 | G | A | -0.057 | 8.18e-06 | 3.39e-11 | 0.978 | 48516584.26 |
| AAA | rs4936098 | G | A | -0.1108 | 8.47e-06 | 7.03e-36 | 0.994 | 171173651.8 |
| AAA | rs4977574 | G | A | -0.182 | 7.55e-06 | 1.67e-114 | 0.998 | 580999100.5 |
| AAA | rs56288724 | G | A | 0.0549 | 8.18e-06 | 1.92e-10 | 0.976 | 45007531.59 |
| AAA | rs56853305 | G | A | -0.0813 | 1.41e-05 | 1.53e-09 | 0.973 | 33406261.81 |
| AAA | rs59755145 | G | A | 0.0755 | 1.60e-05 | 3.12e-11 | 0.978 | 22280494.72 |
| AAA | rs6066802 | C | T | -0.0606 | 8.40e-06 | 8.01e-12 | 0.979 | 52101891.47 |
| AAA | rs6564889 | C | T | -0.0485 | 8.18e-06 | 1.77e-08 | 0.970 | 35125618.75 |
| AAA | rs7025486 | G | A | 0.1018 | 8.30e-06 | 9.53e-31 | 0.993 | 150372432.4 |
| AAA | rs724016 | G | A | 0.0503 | 7.55e-06 | 3.14e-10 | 0.975 | 44425929.38 |
| AAA | rs72745473 | G | A | -0.0612 | 8.47e-06 | 4.79e-12 | 0.979 | 52222810.36 |
| AAA | rs72800535 | C | A | 0.0765 | 1.27e-05 | 1.65e-08 | 0.970 | 36081585.78 |
| AAA | rs72801778 | C | T | -0.1025 | 1.66e-05 | 6.39e-09 | 0.971 | 38115274.89 |
| AAA | rs72878024 | G | A | 0.0921 | 1.57e-05 | 2.78e-08 | 0.969 | 34316821.57 |
| AAA | rs72975824 | G | A | 0.1037 | 1.73e-05 | 1.24e-08 | 0.970 | 35831824.91 |
| AAA | rs729761 | G | T | -0.0589 | 9.23e-06 | 1.52e-09 | 0.974 | 40692811.97 |
| AAA | rs73015011 | C | T | 0.1654 | 1.21e-05 | 2.68e-38 | 0.994 | 187640997.2 |
| AAA | rs73107993 | C | T | 0.0528 | 8.96e-06 | 2.36e-08 | 0.969 | 34705997.78 |
| AAA | rs731141 | G | A | 0.0923 | 7.64e-06 | 3.39e-30 | 0.992 | 145914493.3 |
| AAA | rs73143503 | G | A | -0.0575 | 8.56e-06 | 2.03e-10 | 0.976 | 45080433.02 |
| AAA | rs750607 | C | T | 0.0492 | 7.74e-06 | 1.67e-09 | 0.973 | 40454928 |
| AAA | rs76060974 | G | A | -0.0895 | 1.51e-05 | 2.37e-08 | 0.969 | 35155860.81 |
| AAA | rs761422 | G | A | -0.0451 | 7.64e-06 | 2.17e-08 | 0.969 | 34829530.36 |
| AAA | rs7662069 | G | T | -0.0462 | 7.55e-06 | 9.26e-09 | 0.971 | 37430673.76 |
| AAA | rs76631336 | G | T | -0.0925 | 1.59e-05 | 4.16e-08 | 0.968 | 33659636.22 |
| AAA | rs77925345 | G | A | 0.1428 | 1.61e-05 | 2.53e-20 | 0.988 | 78953979.13 |
| AAA | rs7903146 | C | T | -0.0481 | 8.21e-06 | 3.58e-08 | 0.968 | 34350123.96 |
| AAA | rs7928656 | G | A | -0.0632 | 1.09e-05 | 3.90e-08 | 0.968 | 33356147.41 |
| AAA | rs79486579 | C | A | -0.1199 | 1.55e-05 | 3.58e-15 | 0.984 | 59507310.61 |
| AAA | rs7994761 | G | A | 0.1355 | 9.80e-06 | 1.17e-39 | 0.994 | 191135584.8 |
| AAA | rs8134664 | C | T | -0.066 | 9.15e-06 | 1.07e-11 | 0.979 | 52064963.84 |
| AAA | rs8142788 | G | A | 0.08 | 1.08e-05 | 1.86e-12 | 0.980 | 55355464.01 |
| AAA | rs890746 | G | A | 0.0584 | 7.55e-06 | 3.74e-13 | 0.982 | 59867478.12 |
| AAA | rs9306894 | G | A | -0.0835 | 7.83e-06 | 6.33e-24 | 0.990 | 113718622.1 |
| AAA | rs991967 | C | A | -0.0623 | 8.40e-06 | 2.64e-12 | 0.980 | 55051402 |

**Supplemental Table 6.** Effect Estimates of Abdominal Aortic Aneurysm on the Immunophenotypes in the MR Analyses.

| **Exposure** | **Outcome** | **Outcome type** | **method** | **No. of SNP** | **b** | **or** | **or_lci95** | **or_uci95** | **pval** | **FDR** |
| --- | --- | --- | --- | --- | --- | --- | --- | --- | --- | --- |
| AAA | CD33br HLA DR+ CD14- AC | Absolute count | Inverse variance weighted | 94 | -0.089 | 0.915 | 0.839 | 0.996 | 0.041 | 0.9999 |
| AAA | CD14 on CD33dim HLA DR+ CD11b+ | MFI | Inverse variance weighted | 94 | 0.071 | 1.073 | 1.005 | 1.146 | 0.034 | 0.9999 |
| AAA | CD14 on CD33br HLA DR+ CD14dim | MFI | Inverse variance weighted | 94 | 0.075 | 1.078 | 1.007 | 1.153 | 0.03 | 0.9999 |
| AAA | CD24 on IgD- CD38- | MFI | Inverse variance weighted | 94 | -0.1 | 0.905 | 0.829 | 0.988 | 0.026 | 0.9999 |
| AAA | CD3 on TD CD8br | MFI | Inverse variance weighted | 94 | -0.099 | 0.906 | 0.836 | 0.981 | 0.015 | 0.9999 |
| AAA | CD38 on PB/PC | MFI | Inverse variance weighted | 94 | -0.099 | 0.906 | 0.823 | 0.998 | 0.045 | 0.9999 |
| AAA | CD4 on activated & secreting Treg | MFI | Inverse variance weighted | 94 | -0.095 | 0.91 | 0.833 | 0.993 | 0.034 | 0.9999 |
| AAA | CD24+ CD27+ %B cell | Relative count | Inverse variance weighted | 94 | 0.135 | 1.145 | 1.017 | 1.289 | 0.025 | 0.9999 |
| AAA | CD45RA+ CD28- CD8br %T cell | Relative count | Inverse variance weighted | 94 | -0.107 | 0.899 | 0.82 | 0.986 | 0.024 | 0.9999 |
| AAA | Naive DN (CD4-CD8-) %T cell | Relative count | Inverse variance weighted | 94 | 0.094 | 1.098 | 1.008 | 1.196 | 0.031 | 0.9999 |
